# Proteolytic Surface-Shaving and Serotype-Dependent Expression of SPI-1 Invasion Proteins in *Salmonella enterica* Subspecies *enterica*

**DOI:** 10.3389/fnut.2018.00124

**Published:** 2018-12-10

**Authors:** Clifton K. Fagerquist, William J. Zaragoza

**Affiliations:** Produce Safety & Microbiology Research Unit, Western Regional Research Center, Agricultural Research Service, U.S. Department of Agriculture, Albany, CA, United States

**Keywords:** *Salmonella enterica enterica*, surface-shaving, proteolysis, trypsin, pathogenicity island 1, nano-electrospray ionization, Orbitrap mass spectrometry, flagella

## Abstract

We performed proteolytic surface-shaving with trypsin on three strains/sevovars of *Salmonella enterica enterica* (SEE): Newport, Kentucky, and Thompson. Surfaced-exposed proteins of live bacterial cells were digested for 15 min. A separate 20 h re-digestion was also performed on the supernatant of each shaving experiment to more completely digest protein fragments into detectable peptides for proteomic analysis by nano-liquid chromatography-electrospray ionization-Orbitrap mass spectrometry. Control samples (i.e., no trypsin during surface-shaving step) were also performed in parallel. We detected peptides of flagella proteins: FliC (filament), FliD (cap), and FlgL (hook-filament junction) as well as peptides of FlgM (anti-σ^28^ factor), i.e., the negative regulator of flagella synthesis. For SEE Newport and Thompson, we detected *Salmonella* pathogenicity island 1 (SPI-1) secreted effector/invasion proteins: SipA, SipB, SipC, and SipD, whereas no Sip proteins were detected in control samples. No Sip proteins were detected for SEE Kentucky (or its control) although *sip* genes were confirmed to be present. Our results may suggest a biological response (<15 min) to proteolysis of live cells for these SEE strains and, in the case of Newport and Thompson, a possible invasion response.

## Introduction

Bacterial *surface-shaving* is a technique by which surface-exposed biomolecules (usually proteins) are cleaved from the surface of live cells with proteolytic enzymes, e.g., trypsin, followed by detection by liquid chromatography tandem mass spectrometry (LC/MS/MS) (1–3). A majority of the surface-shaving experiments have been performed on Gram-positive bacteria (1–15). It was reasoned that the peptidoglycan cell wall of Gram-positive bacteria, having greater structural rigidity, would be less likely to rupture during proteolysis than the outer (and inner) membranes of Gram-negative bacteria. Cellular rupture contaminates the sample with cytoplasmic proteins complicating data analysis making it more difficult to assess which proteins are truly surface-exposed. As a certain amount of cell lysis is unavoidable during a shaving experiment, attempts to minimize its occurrence involved primarily reducing the proteolysis time as much as possible, e.g., 15 min (2). Despite the lack of a cell wall, surface-shaving has been performed on a number of Gram-negative bacteria with mixed success (16–21).

In an early work, Grandi and co-workers demonstrated the surface-shaving technique on group A *Streptococcus* (a Gram-positive microorganism) in order to identify new vaccine targets (1). In addition to proteolytic surface-shaving, this influential paper used liquid chromatography tandem mass spectrometry (LC/MS/MS) to detect and identify peptides and their respective proteins by comparison to a proteomic database derived from a genomically sequenced *S. pyogenes* strain (SF370). In addition, proteins identified as surface or surface-associated were analyzed with *in silico* prediction software [e.g., PSORT(22, 23)] to confirm whether the peptides identified by LC/MS/MS were predicted to be surface-exposed. By this approach, new potential vaccine targets were identified.

Trypsin has been the proteolytic enzyme of choice for bottom-up proteomic experiments because it cleaves on the C-terminal side of basic residues: arginine (R) and lysine (K). It has been used to digest proteins in solution as well as in-gel. When ionized by electrospray ionization (ESI) (24) or nano-ESI, (25) tryptic-generated peptides will sequester an ionizing proton at the C-terminal basic residue which, for all practical purposes, is immobilized. Additional ionizing protons will occupy other basic residues (if present due to a missed cleavage) or at the N-terminus or along peptide backbone. During vibrational excitation, e.g., collision-induced dissociation (CID), (26) these additional protons “hop” along the peptide backbone causing fragmentation and resulting in an easily interpretable MS/MS spectrum (27).

Trypsin has been used in many (although not all) surface-shaving experiments primarily because the analysis is LC-ESI/MS/MS. However, trypsin has drawbacks for surface-shaving primarily because the target proteins are often embedded in the outer membrane or cell wall and may not have cleavage sites that are easily accessible even for the protein region that is exposed on the bacterial surface. In consequence, the number of peptides identified from trypsin surface-shaving may be quite limited. To address this issue, other proteolytic enzymes have been utilized that cleave at sites other basic residues, e.g., proteinase K (cleavage at aliphatic and aromatic residues), chymotrypsin, etc. However, the difficulty of cleaving at sites other than at basic residues is that the peptides generated may not fragment efficiently by CID and generate MS/MS spectra that are as easily interpretable compared to MS/MS of tryptic-generated peptides.

Another issue that was noted in early surface-shaving experiments is that the short digestion time (~15–30 min) used in order to minimize cell lysis and contamination with cytoplasmic proteins may result in large protein fragments that may not fragment efficiently by MS/MS. In consequence, a re-digestion step was incorporated in which the supernatant containing peptides and protein fragments from a surface-shaving experiment were digested for a much longer period of time (e.g., 20 h). Implementation of this insight increased the number of identifiable peptides and proteins (3, 12).

*Salmonella enterica* subspecies *enterica* (SEE) is a Gram-negative human pathogen often associated with outbreaks of foodborne illness. There are over 2,500 different serovars of SEE, and pathogenicity and virulence across serovars (and even strains) can vary considerably. There have been very few experiments analyzing the surface-exposed biomolecules of SEE (19). In the current study, we examined three serovars/strains of SEE (Newport, Kentucky, and Thompson) by surface-shaving with trypsin. We chose these particular serovars/strains from our strain collection because each has some relevance to food safety or were associated with a foodborne outbreak. Although a number of surface-associated proteins were identified, we also observed significant proteolytic cleavage of flagella proteins as well as a secreted protein that is a negative regulator of flagella biosynthesis as well as SPI-1 invasion/effector proteins in the case of Newport and Thompson serovars. Our results may suggest an unusually rapid response (<15 min) of these pathogens to proteolytic damage of their flagella perhaps triggering a virulence response.

## Experimental Section

### Culture Conditions

Strains utilized in this study are shown in Table 1 (28, 29). Strains were inoculated from glycerol stocks into LB broth and incubated overnight at 37°C with 200 rpm agitation. The following morning, 5 μL of overnight culture was sub-cultured into 5 mL of fresh LB broth and incubated until mid-log phase (OD_600_ ≈ 0.4). Cells were then harvested for the surface-shaving experiment.

**Table 1:** Strains used in this study.

**Strain**	**Description**	**Source**
RM1655	*Salmonella enterica* subspecies *enterica serovar* Newport	Greg Inami (CA State Health Lab, Berkeley). Strain isolated from alfalfa seeds responsible for an outbreak of S. Newport (28, 29).
RM7890	*Salmonella enterica* subspecies *enterica serovar* Kentucky	Isolated from ground chicken by Food Safety & Inspection Service, USDA (Alameda, CA)
RM1987	*Salmonella enterica* subspecies *enterica serovar* Thompson	Sharon Abbott (CADHS). Human isolate putatively part of an outbreak due to contaminated cilantro, epidemiologically linked to Cilantro.

### Cell Preparation

Cells were removed from the incubator at mid-log phase and quenched on ice for 5 min. A 1 mL aliquot of cells was transferred to sterile 1.5 mL snap-cap tubes and centrifuged at 1,400 rpm for 15 min at 4°C. The broth media was discarded and the cells were suspended in 1 mL of sterile, ice-cold 1x phosphate buffered saline (PBS) and centrifuged at 1,400 rpm for 15 min. The PBS was discarded and the cells were suspended in 1 mL of 1x PBS to which was added 2 μg of modified, sequencing grade porcine trypsin (Product # V5111, Promega, Madison,WI). As a control, a 1 mL aliquot of cells were similarly pelleted by centrifugation, washed and re-suspended in 1 mL of 1x PBS but *without* trypsin.

### Proteolytic Surface-Shaving

Cell samples with trypsin and *without* trypsin (control) were incubated for 15 min at 37°C and 75 rpm. Cells were then centrifuged at 13,000 rpm for 5 min. The resulting supernatant of both samples were collected separately, filtered through a 0.2 μm filter (Millipore) to remove cells and partitioned into two equal 0.5 mL aliquots. One trypsin surface-shaving aliquot was diluted with 0.5 mL of 1x PBS, filtered through a 10 kDa MWCO spin filter (VWA) with centrifugation at 14,000 g for 10 min to remove trypsin. The eluent was transferred to an HPLC vial and stored at −20°C for subsequent analysis. The other trypsin surface-shaving aliquot was diluted with 0.5 mL in 1x PBS to which was added 2 μg of trypsin, and the sample was incubated for 20 h at 37°C at 75 rpm. This re-digested sample was then filtered with a 10 kDa MWCO spin filter to remove trypsin and transferred to an HPLC vial and stored at −20°C for subsequent analysis.

The 0.2 μm filtered supernatants of cell samples that underwent “surface-shaving” in the *absence* of trypsin were also separated into two equal 0.5 mL aliquots. One aliquot was diluted with 0.5 mL of 1x PBS to which was added 2 μg of trypsin. The sample was incubated for 15 min at 37°C at 75 rpm and filtered through a 10 kDa MWCO spin filter with centrifugation at 14,000 g for 10 min. The eluent was transferred to an HPLC vial and stored at −20°C for subsequent analysis. The other aliquot was diluted with 0.5 mL of 1x PBS to which was added 2 μg of trypsin. This sample was incubated for 20 h at 37°C and 75 rpm and was filtered through a 10 kDa MWCO spin filter with centrifugation at 14,000 g for 10 min. The eluent was transferred to an HPLC vial and stored at −20°C for subsequent analysis.

### Nano-Liquid Chromatography-Tandem Mass Spectrometry (nano-LC-MS/MS)

Samples were analyzed using a nano-LC system (Tempo™, nano MDLC, Applied Biosystems/Eksigent) with a PicoSlide nano-electrospray (nano-ESI, 3 column set-up) ion source (New Objective, Woburn, MA) coupled to a hybrid LTQ-Orbitrap Elite mass spectrometer (Thermo Fisher Scientific, San Jose, CA). An 8–10 μL aliquot of sample was loaded onto a 20 μL stainless steel loop using an Ultra-Plus II autosampler (Micro-Tech Scientific). The sample slug was then transferred to a one of the three PicoChip columns (C18-AQ, 3 μm, 120 Å, 105 mm, New Objective) at a flow rate of 400 nL/min using a NanoEasy n-LC II (Thermo Scientific) HPLC. The loading solution was 5% acetonitrile, 95% water, and 0.1% formic acid. Sample was eluted from the column at flow rate of 400 nL/min using the following gradient: 0.0 to 58.0 min, A: 98 to 70%, respectively, followed by 58.0 to 58.5 min, A: 70 to 98%, respectively, followed by 58.5 to 60.0 min, A: 98 to 98%. Buffer A was 0.1% formic acid in HPLC grade water (Optima® LC/MS grade, Fisher Chemical). Buffer B was 0.1% formic acid in HPLC grade acetonitrile (Optima® LC/MS grade, Fisher Chemical). After column elution, the next loaded column was automatically moved in-line for elution and mass spectrometry analysis. The recently eluted column was automatically moved out of alignment with the mass spectrometer and was subjected to a series of four fast ramping sawtooth washing cycles from high-to-low organic (90 to 10%).

ESI voltage was 2.5 kV. A heated metal capillary at 250°C was used for ESI desolvation. No sheath or auxiliary gas was used. A data dependent analysis was performed using a FTMS scan range of *m/z* 400–2,000 at a resolution 60,000 in profile mode using the Orbitrap mass analyzer. The top 10 putative peptide ions were selected from the MS survey scan on the basis of charge state (+2, +3, +4) and signal intensity for collision-induced dissociation (CID) tandem mass spectrometry (MS/MS) in the linear trap. MS/MS (Data type: centroid) was performed with a minimum signal threshold: 30,000; isolation width (*m/z*): 2.0; normalized collision energy: 35.0; activation Q: 0.250 and activation time (ms): 30.

Prior to and after analysis of *Salmonella* surface-shaving samples, the retention time of the LC and the mass spectrometry calibration of the instrument system were tested with a 200 fmol injection of a bovine serum albumin (BSA) digest. BSA tryptic peptides started eluting ~12 min. The root-mean-square (rms) error of the precursor ion *m/z* was below 10 ppm as calculated by the search engine. Three technical replicates were performed on all surface-shaving samples and control samples, and two biological replicates were performed on different days.

### Bioinformatics and Proteomic Analysis

Three databases were constructed for proteomic searches. The SEE Newport database consists of 347,185 protein sequences from 148 genomes downloaded from NCBI non-redundant protein database. The SEE Kentucky database was consists 452,644 protein sequences from 14 genomes. The SEE Thompson database is comprised 63,971 protein sequences from 8 genomes.

Raw MS and MS/MS data files (Xcalibur) were extracted and converted to.mgf files using the MSConvert (ProteoWizard). Database searches were performed with Mascot v2.2.04 (Matrix Science, London, UK). Searches were conducted using a fragment mass tolerance of 0.40 Da and peptide mass tolerance of 20.0 ppm. Trypsin was specified as the enzyme. Searches allowed a maximum of 3 missed cleavages and methionine oxidation was set as a variable modification.

### Supplementary Materials

Raw Mascot proteomic identifications are provided in the Supplementary Materials file. This data is organized by SEE serovar, biological and technical replicates of both samples and their corresponding control samples. For each analysis, protein identifications that are highlighted in yellow are summarized in Tables 2–4 of the manuscript (excluding cytoplasmic proteins which are the result of cell lysis during surface shaving).

**Table 2:** Summary of surface-shaving of SEE Newport.

				**Day 1**						**Day 2**							
				**Mascot scores**			**Number of peptides**			**Mascot scores**			**Number of peptides**				
**Accession number**	**Protein description**	**MW (Da)**	**AA**	**1st**	**2nd**	**3rd**	**1st**	**2nd**	**3rd**	**1st**	**2nd**	**3rd**	**1st**	**2nd**	**3rd**		
**NEWPORT 15 MIN**																	
gi|50830890|gb|AAT81610.1|	Phase 1 flagellin, **FliC** [Salmonella enterica subsp. enterica serovar Newport]	52,223	502	136	78		3	2		388	267	429	3	3	3		
gi|459466347|gb|EMG61125.1|	Flagellar biosynthesis protein **FliC**, partial [Salmonella enterica subsp. enterica serovar Newport str. SH111077]	8447	79	136			3										
gi|194401173|gb|ACF61395.1|	Flagellar hook-associated protein 2 (HAP2), **FliD** [Salmonella enterica subsp. enterica serovar Newport str. SL254]	49,778	467							159	280	358	1	2	2		
gi|194404219|gb|ACF64441.1|	Flagellar hook-associated protein 3 (HAP3), **FlgL** [Salmonella enterica subsp. enterica serovar Newport str. SL254]	34,155	317	88		25	2		1	47			1				
gi|194403331|gb|ACF63553.1|	Negative regulator of flagellin synthesis, **FlgM** [Salmonella enterica subsp. enterica serovar Newport str. SL254]	10,561	97	188		127	1		2	209	244	337	3	2	3		
gi|194402702|gb|ACF62924.1|	Cell invasion protein **SipA** [Salmonella enterica subsp. enterica serovar Newport str. SL254]	72,333	670	89	67	62	2	2	2	299	242	254	5	3	4		
gi|194403640|gb|ACF63862.1|	Cell invasion protein **SipB** [Salmonella enterica subsp. enterica serovar Newport str. SL254]	62,382	593	66	103	64	2	5	2	112	59	105	3	2	2		
gi|392616945|gb|EIW99373.1|	Pathogenicity island 1 effector protein **SipC** [Salmonella enterica subsp. enterica serovar Newport str. Levine 15]	42,957	409	332	196	219	6	4	4	289	256	308	5	2	4		
gi|392616944|gb|EIW99372.1|	Cell invasion protein **SipD** [Salmonella enterica subsp. enterica serovar Newport str. Levine 15]	37,081	343	192		85	1		1	225	195	236	2	2	1		
gi|392765192|gb|EJA21981.1|	Phage immunity repressor protein [Salmonella enterica subsp. enterica serovar Newport str. CVM 19449]	21,776	196	20			1										
							21	13	12				23	16	19	104	Total
**NEWPORT 20 H**																	
gi|50830890|gb|AAT81610.1|	Phase 1 flagellin, **FliC** [Salmonella enterica subsp. enterica serovar Newport]	52,223	502	499	613	222	6	9	5	331	170	419	3	2	3		
gi|874404664|gb|KMU13862.1|	Flagellin **FliC** [Salmonella enterica subsp. enterica serovar Newport str. DC_10-446]	34,557	337		117			5									
gi|194401173|gb|ACF61395.1|	Flagellar hook-associated protein 2 (HAP2), **FliD** [Salmonella enterica subsp. enterica serovar Newport str. SL254]	49,778	467							89	191		1	1			
gi|194404219|gb|ACF64441.1|	Flagellar hook-associated protein 3 (HAP3), **FlgL** [Salmonella enterica subsp. enterica serovar Newport str. SL254]	34,155	317	112	48		2	1		41	32		1	1			
gi|194403331|gb|ACF63553.1|	Negative regulator of flagellin synthesis, **FlgM** [Salmonella enterica subsp. enterica serovar Newport str. SL254]	10,561	97	314	198	305	3	1	4	145	262	347	3	4	4		
gi|194402702|gb|ACF62924.1|	Cell invasion protein **SipA** [Salmonella enterica subsp. enterica serovar Newport str. SL254]	72,333	670	173	221	123	3	4	3	476	531	237	7	5	4		
gi|194403640|gb|ACF63862.1|	Cell invasion protein **SipB** [Salmonella enterica subsp. enterica serovar Newport str. SL254]	62,382	593	55	101	53	3	3	2	54			2				
gi|392616945|gb|EIW99373.1|	Pathogenicity island 1 effector protein **SipC** [Salmonella enterica subsp. enterica serovar Newport str. Levine 15]	42,957	409	662	587	354	9	6	5	262	216	380	5	2	3		
gi|392616944|gb|EIW99372.1|	Cell invasion protein SipD [Salmonella enterica subsp. enterica serovar Newport str. Levine 15]	37,081	343			111			1	139	214	189	1	1	1		
gi|392742504|gb|EIZ99592.1|	Phage regulatory protein [Salmonella enterica subsp. enterica serovar Newport str. CVM 35199]	9236	80								21			1			
							26	29	20				23	18	15	131	Total
**CONTROL NEWPORT 15 MIN**																	
																	Total
**CONTROL NEWPORT 20 H**																	
gi|392765192|gb|EJA21981.1|	Phage immunity repressor protein [Salmonella enterica subsp. enterica serovar Newport str. CVM 19449]	21,776	196		21			1									
								1								1	Total

**Table 3:** Summary of surface-shaving of SEE Kentucky.

				**Day 1**						**Day 2**							
				**Mascot scores**			**Number of peptides**			**Mascot scores**			**Number of peptides**				
**Accession number**	**Protein description**	**MW (Da)**	**AA**	**1st**	**2nd**	**3rd**	**1st**	**2nd**	**3rd**	**1st**	**2nd**	**3rd**	**1st**	**2nd**	**3rd**		
**KENTUCKY 15 MIN**																	
gi|969071361|gb|KUB03511.1|	Flagellin **FliC** [Salmonella enterica subsp. enterica serovar Kentucky]	52,225	502	127	103		3	3		154	105	183	4	3	4		
gi|983253333|gb|KWU90600.1|	Flagellin **FliC** [Salmonella enterica subsp. enterica serovar Kentucky]	51,683	495									158			3		
gi|148644991|gb|ABR01027.1|	Phase 2 flagellin **FljB**, partial [Salmonella enterica subsp. enterica serovar Kentucky]	52,459	500		103			3									
gi|194455642|gb|EDX44481.1|	Flagellar hook-associated protein 3 (HAP3) **FlgL** [Salmonella enterica subsp. enterica serovar Kentucky str. CVM29188]	34,155	317		15			1		42	52	45	2	3	3		
gi|194456320|gb|EDX45159.1|	Negative regulator of flagellin synthesis, **FlgM** [Salmonella enterica subsp. enterica serovar Kentucky str. CVM29188]	10,561	97	183	100	116	2	2	2	69	302	123	3	5	5		
gi|194455511|gb|EDX44350.1|	Major outer membrane lipoprotein [Salmonella enterica subsp. enterica serovar Kentucky str. CVM29188]	8386	78								96			2			
gi|969072264|gb|KUB04403.1|	Major outer membrane lipoprotein 2 [Salmonella enterica subsp. enterica serovar Kentucky]	8531	80							39			3				
							5	9	2				12	13	15	56	Total
**KENTUCKY 20 H**																	
gi|148644993|gb|ABR01028.1|	Phase 1 flagellin **FliC**, partial [Salmonella enterica subsp. enterica serovar Kentucky]	51,551	495								456			7			
gi|969071361|gb|KUB03511.1|	Flagellin **FliC** [Salmonella enterica subsp. enterica serovar Kentucky]	52,225	502	333	353		6	6		200	430	121	5	7	3		
gi|983253333|gb|KWU90600.1|	Flagellin **FliC** [Salmonella enterica subsp. enterica serovar Kentucky]	51,683	495								326			7			
gi|115381392|gb|ABI96378.1|	Phase 2 flagellin **FljB**, partial [Salmonella enterica subsp. enterica serovar Kentucky]	48,340	462			49			1								
gi|194455957|gb|EDX44796.1|	Flagellar hook-associated protein 2 (HAP2), **FliD** [Salmonella enterica subsp. enterica serovar Kentucky str. CVM29188]	49,778	467								176			3			
gi|194455642|gb|EDX44481.1|	Flagellar hook-associated protein 3 (HAP3), **FlgL** [Salmonella enterica subsp. enterica serovar Kentucky str. CVM29188]	34,155	317	36	91		1	3		63	45	25	3	2	1		
gi|194456320|gb|EDX45159.1|	Negative regulator of flagellin synthesis **FlgM** [Salmonella enterica subsp. enterica serovar Kentucky str. CVM29188]	10,561	97	299	355	190	4	5	3	77	546	59	2	4	2		
gi|553491170|gb|ESC15085.1|	Anti-sigma28 factor **FlgM** [Salmonella enterica subsp. enterica serovar Kentucky str. 0253]	10,577	97	235	277	175	4	5	3								
gi|194459234|gb|EDX48073.1|	Outer membrane protein A [Salmonella enterica subsp. enterica serovar Kentucky str. CVM29188]	40,058	371								27			1			
gi|444820129|gb|ELX47576.1|	Tail fiber domain protein [Salmonella enterica subsp. enterica serovar Kentucky str. 29439]	35,445	332	21	18		1	1									
							16	20	7				10	31	6	90	Total
**CONTROL KENTUCKY 15 MIN**																	
gi|554231997|gb|ESG69366.1|	Outer membrane protein [Salmonella enterica subsp. enterica serovar Kentucky str. ATCC 9263]	27,757	252		15			1									
								**1**								1	Total
**CONTROL KENTUCKY 20 H**																	
gi|444820129|gb|ELX47576.1|	Tail fiber domain protein [Salmonella enterica subsp. enterica serovar Kentucky str. 29439]	35,445	332	18	21	18	1	1	1								
gi|115381392|gb|ABI96378.1|	Phase 2 flagellin **FljB**, partial [Salmonella enterica subsp. enterica serovar Kentucky]	48,340	462								21	39		1	2		
gi|969071361|gb|KUB03511.1|	Flagellin **FliC** [Salmonella enterica subsp. enterica serovar Kentucky]	52,225	502									39			2		
gi|969072435|gb|KUB04565.1|	Flagellin **FliC** [Salmonella enterica subsp. enterica serovar Kentucky]	52,690	506									39			2		
							1	1	1					1	6	10	Total

**Table 4:** Summary of surface-shaving of SEE Thompson.

				**Day 1**						**Day 2**							
				**Mascot scores**			**Number of peptides**			**Mascot scores**			**Number of peptides**				
**Accession number**	**Protein description**	**MW (Da)**	**AA**	**1st**	**2nd**	**3rd**	**1st**	**2nd**	**3rd**	**1st**	**2nd**	**3rd**	**1st**	**2nd**	**3rd**		
**THOMPSON 15 MIN**																	
gi|50830928|gb|AAT81629.1|	Phase 1 flagellin **FliC** [Salmonella enterica subsp. enterica serovar Thompson]	51,467	495	181	150		2	2									
gi|548714623|gb|AGX10263.1|	Flagellar hook-associated protein **FlgL** [Salmonella enterica subsp. enterica serovar Thompson str. RM6836]	34,155	317	68	51	33	2	1	1								
gi|548714611|gb|AGX10251.1|	Anti-sigma28 factor **FlgM** [Salmonella enterica subsp. enterica serovar Thompson str. RM6836]	10,561	97			77			1								
gi|548715882|gb|AGX11522.1|	Pathogenicity island 1 effector protein **SipA** [Salmonella enterica subsp. enterica serovar Thompson str. RM6836]	73,927	685	84	63		1	1		59			1				
gi|548715885|gb|AGX11525.1|	Pathogenicity island 1 effector protein **SipB** [Salmonella enterica subsp. enterica serovar Thompson str. RM6836]	62,412	593	117	239	151	5	6	4								
gi|548715884|gb|AGX11524.1|	Pathogenicity island 1 effector protein **SipC** [Salmonella enterica subsp. enterica serovar Thompson str. RM6836]	42,957	409	256	278	232	4	3	4	46	54	108	1	1	2		
gi|548715883|gb|AGX11523.1|	Cell invasion protein **SipD** [Salmonella enterica subsp. enterica serovar Thompson str. RM6836]	37,081	343							28			1				
gi|548714481|gb|AGX10121.1|	Enterohemolysin [Salmonella enterica subsp. enterica serovar Thompson str. RM6836]	40,698	369	134	119		2	2		39	84		2	1			
gi|548714507|gb|AGX10147.1|	Tail protein [Salmonella enterica subsp. enterica serovar Thompson str. RM6836]	112,007	1031	71	61	59	1	1	1								
gi|808222093|gb|KKD68276.1|	Tail protein [Salmonella enterica subsp. enterica serovar Thompson]	84,119	834			115			2								
gi|548714496|gb|AGX10136.1|	Head-tail joining protein [Salmonella enterica subsp. enterica serovar Thompson str. RM6836]	7380	67							99	48		1	1			
gi|548714499|gb|AGX10139.1|	Head decoration protein [Salmonella enterica subsp. enterica serovar Thompson str. RM6836]	11,916	115	19			1			76	115	91	2	2	2		
							18	16	13				8	5	4	64	Total
**THOMPSON 20 H**																	
gi|50830928|gb|AAT81629.1|	Phase 1 flagellin **FliC** [Salmonella enterica subsp. enterica serovar Thompson]	51,467	495			505			7	79	83	123	2	1	1		
gi|548715793|gb|AGX11433.1|	Flagellin **FliC** [Salmonella enterica subsp. enterica serovar Thompson str. RM6836]	52,487	506	1034	966		9	8									
gi|548714623|gb|AGX10263.1|	Flagellar hook-associated protein **FlgL** [Salmonella enterica subsp. enterica serovar Thompson str. RM6836]	34,155	317	62		34	1		1								
gi|548714611|gb|AGX10251.1|	Anti-sigma28 factor **FlgM** [Salmonella enterica subsp. enterica serovar Thompson str. RM6836]	10,561	97	60			1										
gi|548715882|gb|AGX11522.1|	Pathogenicity island 1 effector protein **SipA** [Salmonella enterica subsp. enterica serovar Thompson str. RM6836]	73,927	685	136	112	78	2	3	1		200			3			
gi|548715885|gb|AGX11525.1|	Pathogenicity island 1 effector protein **SipB** [Salmonella enterica subsp. enterica serovar Thompson str. RM6836]	62,412	593	72	107	107	3	3	2								
gi|548715884|gb|AGX11524.1|	Pathogenicity island 1 effector protein **SipC** [Salmonella enterica subsp. enterica serovar Thompson str. RM6836]	42,957	409	567	480	350	6	5	6	48	178	281	1	3	3		
gi|548714481|gb|AGX10121.1|	Enterohemolysin [Salmonella enterica subsp. enterica serovar Thompson str. RM6836]	40,698	369	262	236	69	3	5	3	54	47		2	2			
gi|808222093|gb|KKD68276.1|	Tail protein [Salmonella enterica subsp. enterica serovar Thompson]	84,119	834	201	249	255	1	2	4	49	35		1	1			
gi|548714507|gb|AGX10147.1|	Tail protein [Salmonella enterica subsp. enterica serovar Thompson]	112,007	1031		43	40		1	1								
gi|548714105|gb|AGX09745.1|	Trigger factor [Salmonella enterica subsp. enterica serovar Thompson str. RM6836]																
gi|548714505|gb|AGX10145.1|	Minor tail protein [Salmonella enterica subsp. enterica serovar Thompson str. RM6836]	14,833	131	84	54	43	1	1	1	64	142	128	2	2	2		
gi|548716337|gb|AGX11977.1|	Integration host factor subunit alpha [Salmonella enterica subsp. enterica serovar Thompson str. RM6836]																
gi|548714525|gb|AGX10165.1	Membrane protein [Salmonella enterica subsp. enterica serovar Thompson str. RM6836]	39,940	369			18			1		54			1			
gi|548714500|gb|AGX10140.1|	Head protein [Salmonella enterica subsp. enterica serovar Thompson str. RM6836]	38,058	342	60	143	72	2	1	1								
gi|548714499|gb|AGX10139.1|	Head decoration protein [Salmonella enterica subsp. enterica serovar Thompson str. RM6836]	11,916	115	16	14	14	1	1	1		44	43		2	1		
gi|911477197|gb|KNN22204.1|	Phage tail protein [Salmonella enterica subsp. enterica serovar Thompson]	?	656								28			1			
							30	30	29				8	16	7	120	Total
**CONTROL THOMPSON 15 MIN**																	
gi|548714499|gb|AGX10139.1|	Head decoration protein [Salmonella enterica subsp. enterica serovar Thompson str. RM6836]	11,916	115							18	55		1	1			
													1	1		2	Total
**CONTROL THOMPSON 20 H**																	
gi|548714505|gb|AGX10145.1|	Minor tail protein [Salmonella enterica subsp. enterica serovar Thompson str. RM6836]	14,833	131								46	21		1	1		
														1	1	2	Total

### Polymerase Chain Reaction (PCR)

PCR was used to verify the presence of the *sip* operon and *hilA* in *SEE* Kentucky. PCR was carried out on a Tetrad 2 (Bio-Rad, Hercules, CA) using colonies of *SEE* Kentucky with the primers listed in Data Sheet 1 (Supplementary Materials Kentucky, page 65) under the following conditions: an initial denaturation step was at 94°C for 10 min followed by 29 cycles at 94°C for 30 s, 53°C for 30 s, and 72°C for 2 min with a final elongation cycle at 72°C for 7 min. PCR products were analyzed by gel electrophoresis and imaged using a GelDoc XR (Biorad, Hercules, CA).

## Results and Discussion

### *Salmonella enterica* Subspecies *enterica* (SEE) Serovar Newport

Table 2 summarizes the results of 15 min surface-shaving experiment (and 20 h re-digestion) of SEE Newport strain RM1655 and their controls. Both the MASCOT identification scores and the corresponding number of peptides identified are reported for three technical replicates. In addition, two biological replicates were performed on different days. More detailed proteomic information on peptide/protein identifications (including any cytoplasmic proteins detected) is provided in Supplementary Materials Newport (pages 1-64). Table 2 shows a number of tryptic peptide identifications corresponding to cleavage of flagella proteins (FliC, FliD, FlgL) in both the 15 min experiment as well as the 20 h re-digestion. We observe an overall increase in the number of peptides detected in the 20 h re-digestion (131) compared with the 15 min surface-shaving (104) as one might expect given the fact that the 15 min experiment may produce protein fragments too large to be detected by MS/MS, whereas the 20 h re-digestion allows greater time for large protein fragments to be enzymatically cleaved into smaller, more detectable peptides.

**Figure 1:**
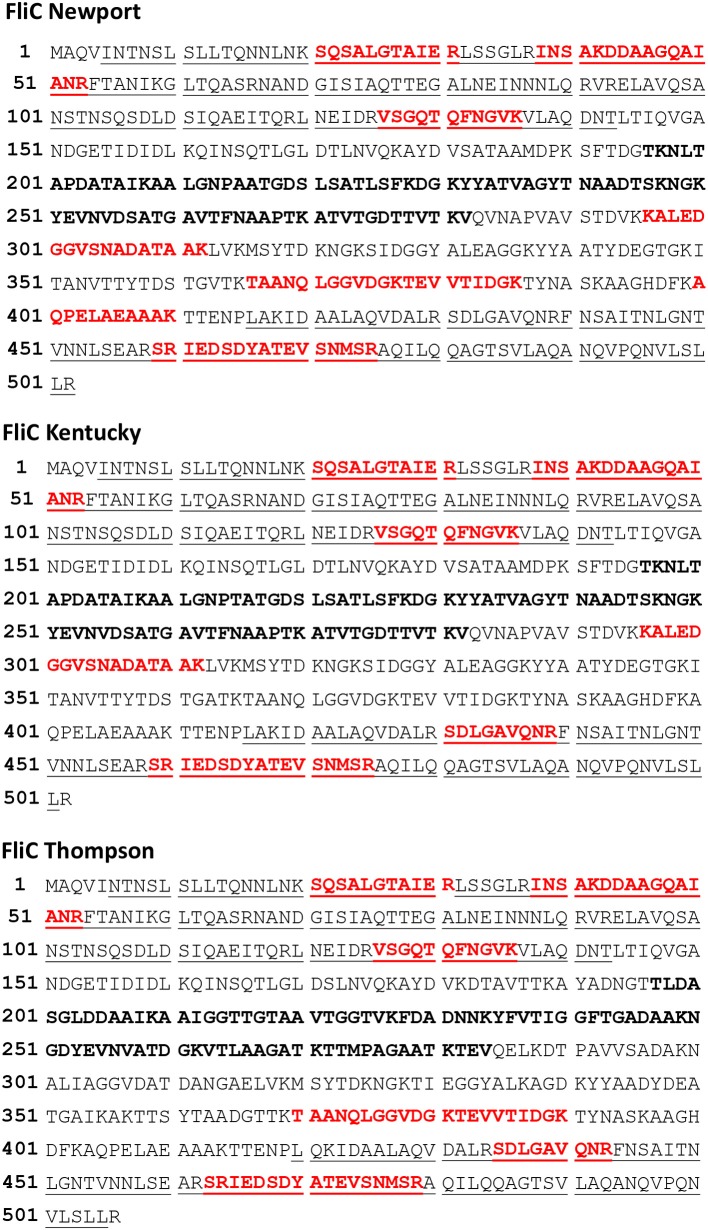
Top panel. Phase 1 flagellin (FliC) sequence of *Salmonella enterica* subsp. *enterica* (SEE) serovar Newport strain SGSC2493. Bold red denotes the highest sequence coverage obtained for a single analysis of SEE Newport strain RM1655 (Table 2, 20 h, Day 1, 2nd analysis). Underlined residues are the N-terminal(5–143) and C-terminal (416–501) helical regions, respectively. Bold black residues denotes the D3 domain (196–282). Middle Panel. Flagellin (FliC) sequence from SEE serovar Kentucky strain CVM N38870. Bold red denotes highest sequence coverage obtained for a single analysis of SEE Kentucky strain RM7890 (Table 3, 20 h, Day 2, 2nd analysis). Underlined residues are the N-terminal (5–143) and C-terminal (416–501) helical regions, respectively. Bold black residues denotes the D3 domain (196–282). Bottom panel. Flagellin (FliC) sequence for SEE serovar Thompson str. RM6836. Bold red denotes highest sequence coverage obtained for a single analysis SEE Thompson strain RM1987 (Table 4, 20 h, Day 1, 1st analysis). Underlined residues are the N-terminal (5–143) and C-terminal (420–505) helical regions, respectively. Bold black residues denotes the D3 domain (197–284).

**Figure 2:**
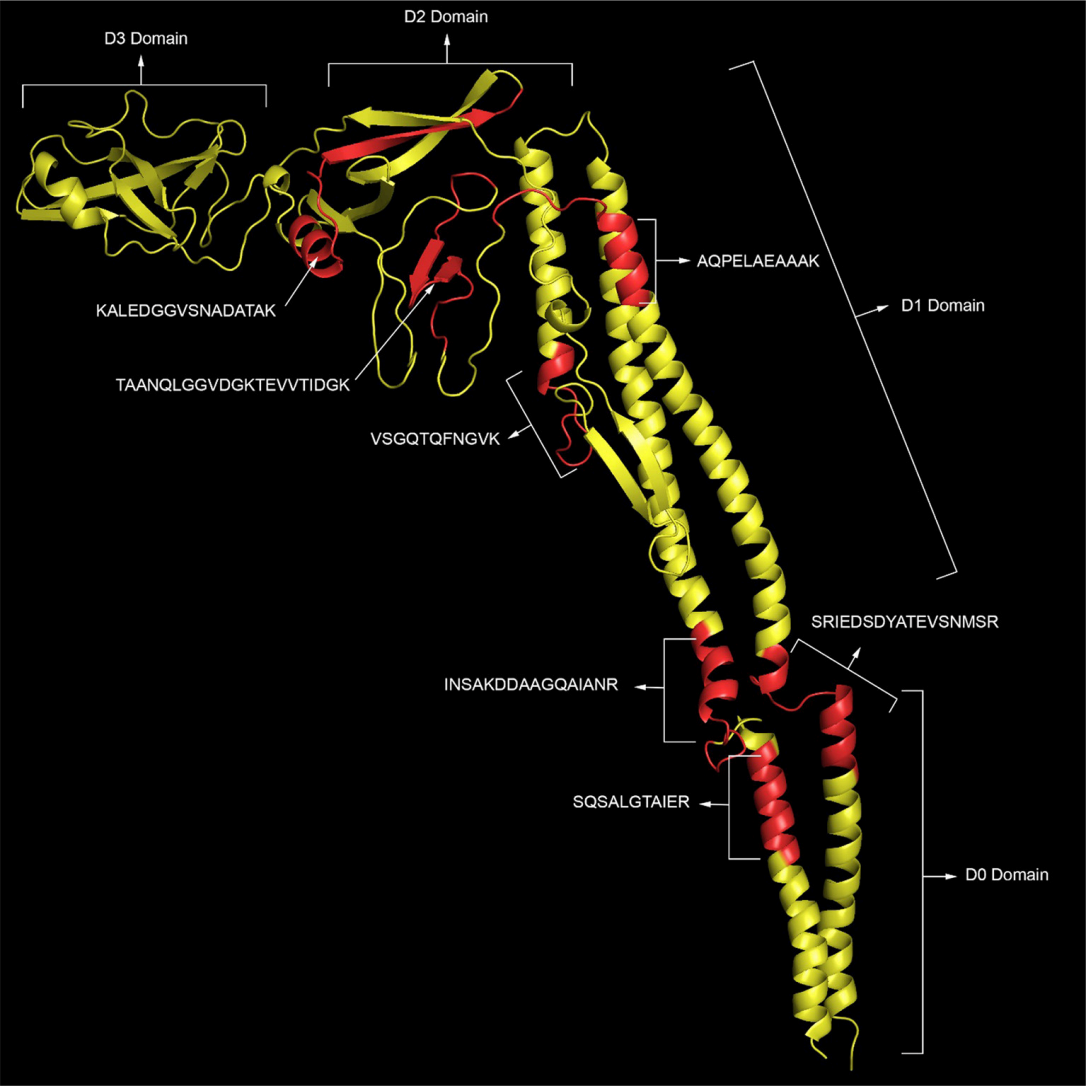
The 3-D image of FliC of *S. enterica* based on X-ray crystallographic structure from Protein Data Bank (Entry: 3A5X) (32) and viewed in PyMOL. The corresponding peptide sequence coverage of SEE Newport is highlighted in red and specific peptides highlighted in white. Specific domains (D0, D1, D2, D3) are also indicated.

Phase 1 flagellin (FliC) is the most abundant of the flagella proteins with approximately 30,000 proteins per flagella (and 5-10 flagella per cell), and it is the primary structural constituent of the filament that extends into the extracellular space (30, 31). As such, this protein is not only abundant but also highly accessible to proteolytic degradation. Not surprising, we detect the highest number of peptides for this protein. Figure 1 (**top panel**) shows the peptide sequence coverage for FliC. Peptide sequence coverage is highlighted in bold red. The N-terminal (5-143) and C-terminal (416-501) helical domains are underlined and the D3 (196-282) domain is in bold, black. It is interesting that the D3 domain has a total of eight basic residues (eight lysines), but no peptides were detected in this domain. Figure 2 shows the 3-D image of FliC of *S. enterica* based on X-ray crystallographic structure from Protein Data Bank (Entry: 3A5X) (32) and viewed in PyMOL. The corresponding peptide sequence coverage is highlighted in red and specific peptides highlighted in white. Specific domains (D0, D1, D2, D3) are also indicated. Although the D3 domain is probably the most accessible of all the domains of FliC, it has a somewhat globular tertiary structure which may inhibit proteolysis even as part of a larger protein fragment. Interestingly, many (although not all) of the peptides detected appear to be located within secondary helical structures in the D2, D1, and D0 domains. This may suggest that, in the absence of denaturants, trypsin may favor cleavage of the polypeptide backbone at basic residues within alpha-helices. The toll-like receptor 5 region (TLR5) responsible for the innate immune response in eukaryotic cells (33, 34) is present in the upper half of the D1 domain which has three, nearly parallel alpha-helices (shown in Figure 2). The fact that we detect peptides in two of the three helices is consistent with the accessibility of this region.

We also observe a few peptides of FliD (also called HAP2) which functions as the “cap” of the filament as well as FlgL (also called HAP3) which is critical at the junction between the filament and the hook. From a stoichiometric point-of-view, these proteins are significantly less abundant than FliC, so it is not surprising that the number of peptides identified are fewer. However, it is not simply the abundance of the protein but the accessibility of its trypsin cleavable sites that is critical.

The facile detection of flagellin tryptic peptides from this strain of SEE Newport suggest a robust number of flagellin filament structures. Interestingly, we also detected peptides of the negative regulator of flagellin synthesis or FlgM an anti-σ^28^ factor. FlgM is a secreted protein and its secretion is concomitant with up-regulation of flagellin biosynthesis noted by other researchers in *Salmonella typhimurium, Escherichia coli* and *Bacillus subtilis* (35–39). The very strong identification of FlgM (nearly 50% coverage in several analyses) suggests that this protein is highly abundant.

We also identified a number of effector proteins associated with pathogen virulence whose genes are located on pathogenicity island 1 (SPI-1): SipA, SipB, SipC, and SipD. SipA-D are also secreted proteins, and their abundance is high and reproducible as evidenced by the number of tryptic peptides identified in both the 15 min and the 20 h re-digestion analyses. This result is striking as the secretion of effector proteins is to facilitate the invasion of eukaryotic cells even though no eukaryotic cells were present in the sample. The fact that SipA-D appear to be strongly expressed as a result of trypsin proteolysis may suggest that, along with secretion of FlgM, genes related to virulence and invasion may also be activated and their protein products secreted.

Surface-shaving experiments are often accompanied with a certain amount of cell lysis caused by degradation of surface structures that weaken the cell membrane resulting in cell rupture and contamination of the sample with cytoplasmic proteins, e.g., ribosomal proteins. We detect some ribosomal and other cytoplasmic proteins, including one of the most abundant cytoplasmic proteins, i.e., elongation factor Tu (40) (shown in Supplementary Materials Newport, pages 1-65) which suggests a small amount of cell lysis in these experiments.

In parallel, analyses were also performed on control samples, i.e., no trypsin during the 15 min surface-shaving step (Table 2 and Supplementary Materials Newport). In these samples, we did *not* observe any flagella proteins or FlgM or SPI-1 proteins. In addition, we observed very little evidence of cell lysis based upon detection of only a few peptides of cytoplasmic proteins.

The type III secretion system is responsible for flagellin biosynthesis, (36) but it is not clear the mechanism by which SEE Newport would “sense” damage to its flagella. It is possible that tryptic peptides of the flagellin filament are detected by receptors on its surface that signal to the pathogen the presence of damaging proteases in the extracellular milieu. Alternatively, proteolytic damage of the hook-filament junction (a critical structural junction) may result in impaired flagellin movement/operation leading to release FlgM that may re-activate flagellin biosynthesis. It is also possible that damage to the hook-filament junction may lead to detachment of the filament altogether allowing secretion of FlgM.

### SEE Serovar Kentucky

Table 3 summarizes the results of the 15 min surface-shaving experiment and 20 h re-digestion of SEE Kentucky strain RM7890 and their controls. As with the SEE Newport strain, the number of peptides detected/identified is significantly increased for the 20 h re-digest compared to the 15 min shaving experiment which supports the usefulness of this secondary digestion step. Once again, peptides from proteins of the filament and hook/filament junction are detected: FliC, FljB, and FlgL. Figure 1 (**middle panel)** shows sequence coverage obtained for FliC. Peptides from the D0, D1, and D2 domains are detected but not the D3 domain which suggests that this domain appears resistant to proteolysis under the experimental conditions of our experiment. This is not entirely surprising as most bottom-up proteomic analyses incorporate denaturation of the protein prior to digestion to facilitate access to cleavable sites (basic residues). However, denaturation is contrary to the objective of a surface-shaving experiment which is to sample only the most surface-exposed protein structures.

The negative regulator of flagellin synthesis, FlgM, was once again detected and, given the number of peptides detected, appears to be highly abundant. The appearance of FlgM under conditions of very brief exposure to trypsin (15 min) suggests a very rapid biological response to flagellin damage by the SEE Kentucky strain. Interestingly, we detected no peptides of the secreted effector/invasion proteins: SipA, SipB, SipC or SipD. The absence of detection suggested that perhaps their genes may not be present in this strain. In consequence, PCR was performed on the *sip* operon and *hilA* [a transcriptional activator of SPI1 regulation (41, 42)], and both were found to be present in this strain. The absence of *sip* expression may contribute to a lack of pathogenicity in this strain. This finding may be consistent with an assessment by the USDA in 2002 that, although the Kentucky serovar is prevalent in the food supply environment, it is not generally considered a successful human pathogen (43).

We detected only fleeting evidence of peptides of a few outer membrane proteins as shown in Table 3. However, reproducibility was an issue as these peptides were not detected in both biological replicates or with every analysis of a triplicate. This is probably due to their relatively low abundance as well as a portion of the protein being embedded in the membrane.

Control samples for SEE Kentucky showed detection of a few cytoplasmic proteins (Supplementary Materials Kentucky, pages 65–107). However, unlike the first biological replicate, the second biological replicate revealed detection of several cytoplasmic proteins (ribosomal, ef-Tu, etc.) and even flagellin (Table 3 and Supplementary Materials Kentucky). This is likely the result of cell lysis releasing of cytoplasmic proteins as well as breakage of flagellin during processing.

### SEE Serovar Thompson

Table 4 summarizes the results of the 15 min surface-shaving experiment and the 20 h re-digestion on SEE Thompson strain RM1987 and their controls (excluding cytoplasmic proteins detected which are shown in Supplementary Materials Thompson, pages 108–185). Consistent with SEE Newport and SEE Kentucky strains, we observe a significant increase in the number of peptides detected for the 20 h re-digestion (120) vs. that obtained for the 15 min surface-shaving experiment (64). Once again, peptides from FliC filament digestion were detected as well as FlgL of the hook-filament junction. The Figure 1 (**bottom panel)** shows the sequence coverage obtained for FliC sequence of SEE Thompson. Peptides from the D0, D1, and D2 domains (but not the D3) are detected consistent with results from the other two SEE serovars. In addition to this apparent flagellin proteolysis, we observe weak detection of the anti-σ^28^ factor: FlgM.

SEE Thompson shows significant expression of SipA, SipB, SipC and SipD in both the 15 min and the 20 h re-digested samples which suggests, like the SEE Newport strain, a possible response to proteolysis including secretion of effector/invasion proteins. In addition, we detect enterohemolysin which was not detected in the SEE Newport and SEE Kentucky strains. A number of bacteriophage proteins were also detected. SEE Thompson (Supplementary Materials) shows a large number of cytoplasmic proteins detected in the surface-shaving samples (and even in the control samples) suggesting extensive lysis of the inner and outer membranes. It would seem that trypsin significantly weakens the integrity of the SEE Thompson envelope far more than for SEE Newport and SEE Kentucky strains. Another explanation could be that cell lysis is caused by activation of a lytic cycle of a bacteriophage in the host genome resulting in expression of bacteriophage-encoded proteins. Activation of the bacteriophage lytic cycle may be triggered by proteolytic surface-shaving.

The SEE Thompson control samples revealed a significant amount of cell lysis as evident from detection of cytoplasmic proteins in both the 15 min surface-shaving sample as well as the 20 h re-digested of the control samples. As no trypsin was used during the surface-shaving step of control samples, we can only conclude that the cellular membranes of this SEE Thompson strain were more susceptible to rupture. Cell lysis may be due to inability to respond to rapid changes in osmolarity, i.e., from broth to PBS, or membrane fragility during washing with PBS and centrifugation. In any case, the control samples showed no tryptic peptides of flagellin-associated proteins or Sip proteins (and other virulence factors) or bacteriophage proteins.

For this SEE Thompson strain, we conclude the following. The inner and outer membranes of this strain appear to be unusually susceptible to rupture and surface-shaving may exacerbate this tendency resulting in the release of a large number of cytoplasmic proteins. Surface-shaving with trypsin results in proteolytic cleavage of flagellin-associated proteins (FliC and FlgL) and the secreted FlgM and SPI-1 proteins. In addition, peptides of enterohemolysin and bacteriophage-encoded proteins were also detected. The latter may contribute to host cell lysis. Control samples were comprised of almost entirely cytoplasmic proteins.

Although sample contamination by cytoplasmic proteins is a common problem associated with proteolytic surface-shaving experiments, the amount of cell lysis observed for each strain in our study varied significantly and appeared to be strain dependent. SEE Newport was the most resistant to lysis followed by SEE Kentucky and lastly SEE Thompson which showed extensive cell lysis as evident from the number of cytoplasmic proteins detected. The use of PBS as the medium to perform all microbiological manipulations reduced the likelihood of cell lysis by maintaining mild osmotic conditions although it may have reduced the efficiency of trypsin digestion.

## Conclusions

Proteolytic surface-shaving with trypsin of live SEE bacterial cells resulted in significant cleavage of flagella filament and hook-associated proteins and secretion of the negative regulator of flagellin biosynthesis: FlgM which may suggest up-regulation of flagellin biosynthesis. In addition, invasion/effector Sip proteins were also expressed in the Newport and Thompson strains. In the *absence* of trypsin during the shaving-shaving step, no significant flagella proteolysis occurred and FlgM and Sip were not detected. The Kentucky serovar/strain, although possessing *sip* genes, did not express Sip proteins (at least not at levels detectable by our measurement).

For all three SEE strains/serovars, tryptic-generated peptides from proteolytic cleavage of the flagellin filament, FliC, were detected. Interestingly, no peptides were detected in the most accessible domain of FliC (i.e., D3) though the domain possessed seven or eight lysine residues. It is possible that the tertiary structure of the D3 domain (globular) may thwart efficient proteolysis in contrast to peptides that possess alpha-helical secondary structures.

Cell lysis can be a confounding problem of proteolytic surface-shaving experiments as it contaminates the sample with non-surface-exposed proteins (i.e., cytoplasmic proteins). In our experiments, cell lysis appeared to be serovar/strain dependent that may reflect the general robustness of the outer and inner membranes during sample processing. The greatest amount of lysis occurred with SEE Thompson which was accompanied by detection of many bacteriophage and cytoplasmic proteins that may suggest activation of a bacteriophage lytic cycle and that cell rupture may not have been entirely due to the intrinsic stability of the cell membrane. Our results suggest that brief proteolytic surface-shaving may be a useful technique to assess the potential virulence and robustness of SEE strains/serovars *in vitro*. Other techniques for assessing potential SEE virulence would be mice model, mammalian cells *in vitro* invasion assay (e.g., Caco2 cell line) or perhaps whole genome sequencing (44). Advantages of surface-shaving in assessing potential virulence would be that it does not require the use of live animals or preparation of mammalian cells *in vitro*. As surface-shaving is a mass spectrometry-based proteomic technique, we only detect expressed proteins (and by implication their genes) under specific experimental conditions.

## Author Contributions

CF conceptualized experiment, analyzed data and drafted and finalized manuscript. WZ performed microbiological experiments and collected mass spectrometry data and preliminary data analysis, reviewed drafts, and final manuscript.

### Conflict of Interest Statement

The authors declare that the research was conducted in the absence of any commercial or financial relationships that could be construed as a potential conflict of interest.

## Supplementary Material

Data Sheet 1 Mascot proteomic identifications of surface-shaving (and controls) for SEE Newport, Kentucky and Thompson.
