# Supplementary material for: Proteolytic Surface-Shaving and Serotype-Dependent Expression of SPI-1 Invasion Proteins in Salmonella enterica Subspecies enterica
Source: Front Nutr. 2018 Dec 10;5:124. doi: 10.3389/fnut.2018.00124 (PMC6295468; doi:10.3389/fnut.2018.00124)
Supplement: Data Sheet 1 — Mascot proteomic identifications of surface-shaving (and controls) for SEE Newport, Kentucky and Thompson. [file Data_Sheet_1.docx]

SEE Newport

The proteins summarized in **Table 2** (in the manuscript) are highlighted in yellow below with detailed MASCOT identifications. Cytoplasmic proteins are not highlighted or detailed.

******************************************************************DAY 1***********************************************************************

**********************************************************15 minute SS****************************************************************

**1^st^ technical replicate**

**Enzyme : Trypsin**

**Variable modifications :** [**Oxidation (M)**](http://10.200.41.11/mascot/cgi/client.pl?modification&mod_name=Oxidation%20%28M%29&file=..%2Fdata%2F20160418%2FF080005.dat)

**Mass values : Monoisotopic**

**Protein Mass : Unrestricted**

**Peptide Mass Tolerance : ± 20 ppm**

**Fragment Mass Tolerance: ± 0.4 Da**

**Max Missed Cleavages : 3**

**Instrument type : Default**

**Number of queries : 3110**

| **Protein hits           :** | [**gi|392616945|gb|EIW99373.1|**](http://10.200.41.11/mascot/cgi/master_results.pl?file=..%2Fdata%2F20160418%2FF080005.dat#Hit1) | pathogenicity island 1 effector protein SipC [Salmonella enterica subsp. enterica serovar Newport str. Levine 15] |
| --- | --- | --- |
|  | [**gi|392616944|gb|EIW99372.1|**](http://10.200.41.11/mascot/cgi/master_results.pl?file=..%2Fdata%2F20160418%2FF080005.dat#Hit2) | cell invasion protein SipD [Salmonella enterica subsp. enterica serovar Newport str. Levine 15] |
|  | [**gi|194403331|gb|ACF63553.1|**](http://10.200.41.11/mascot/cgi/master_results.pl?file=..%2Fdata%2F20160418%2FF080005.dat#Hit3) | negative regulator of flagellin synthesis [Salmonella enterica subsp. enterica serovar Newport str. SL254] |
|  | [**gi|50830890|gb|AAT81610.1|**](http://10.200.41.11/mascot/cgi/master_results.pl?file=..%2Fdata%2F20160418%2FF080005.dat#Hit4) | phase 1 flagellin [Salmonella enterica subsp. enterica serovar Newport] |
|  | [**gi|459466347|gb|EMG61125.1|**](http://10.200.41.11/mascot/cgi/master_results.pl?file=..%2Fdata%2F20160418%2FF080005.dat#Hit5) | Flagellar biosynthesis protein FliC, partial [Salmonella enterica subsp. enterica serovar Newport str. SH111077] |
|  | [**gi|194401698|gb|ACF61920.1|**](http://10.200.41.11/mascot/cgi/master_results.pl?file=..%2Fdata%2F20160418%2FF080005.dat#Hit6) | translation elongation factor Tu [Salmonella enterica subsp. enterica serovar Newport str. SL254] |
|  | [**gi|692117421|ref|WP_032074328.1|**](http://10.200.41.11/mascot/cgi/master_results.pl?file=..%2Fdata%2F20160418%2FF080005.dat#Hit7) | hypothetical protein [Salmonella enterica] |
|  | [**gi|194402702|gb|ACF62924.1|**](http://10.200.41.11/mascot/cgi/master_results.pl?file=..%2Fdata%2F20160418%2FF080005.dat#Hit8) | cell invasion protein SipA [Salmonella enterica subsp. enterica serovar Newport str. SL254] |
|  | [**gi|194404219|gb|ACF64441.1|**](http://10.200.41.11/mascot/cgi/master_results.pl?file=..%2Fdata%2F20160418%2FF080005.dat#Hit9) | flagellar hook-associated protein 3 [Salmonella enterica subsp. enterica serovar Newport str. SL254] |
|  | [**gi|194401878|gb|ACF62100.1|**](http://10.200.41.11/mascot/cgi/master_results.pl?file=..%2Fdata%2F20160418%2FF080005.dat#Hit10) | ribosomal protein L11 [Salmonella enterica subsp. enterica serovar Newport str. SL254] |
|  | [**gi|194403640|gb|ACF63862.1|**](http://10.200.41.11/mascot/cgi/master_results.pl?file=..%2Fdata%2F20160418%2FF080005.dat#Hit11) | cell invasion protein SipB [Salmonella enterica subsp. enterica serovar Newport str. SL254] |
|  | [**gi|194404381|gb|ACF64603.1|**](http://10.200.41.11/mascot/cgi/master_results.pl?file=..%2Fdata%2F20160418%2FF080005.dat#Hit12) | DNA-binding protein HU-alpha [Salmonella enterica subsp. enterica serovar Newport str. SL254] |
|  | [**gi|194401103|gb|ACF61325.1|**](http://10.200.41.11/mascot/cgi/master_results.pl?file=..%2Fdata%2F20160418%2FF080005.dat#Hit13) | ribosomal protein L6 [Salmonella enterica subsp. enterica serovar Newport str. SL254] |
|  | [**gi|194403829|gb|ACF64051.1|**](http://10.200.41.11/mascot/cgi/master_results.pl?file=..%2Fdata%2F20160418%2FF080005.dat#Hit14) | ribosomal protein L3 [Salmonella enterica subsp. enterica serovar Newport str. SL254] |
|  | [**gi|194403359|gb|ACF63581.1|**](http://10.200.41.11/mascot/cgi/master_results.pl?file=..%2Fdata%2F20160418%2FF080005.dat#Hit15) | ribosomal protein L10 [Salmonella enterica subsp. enterica serovar Newport str. SL254] |
|  | **[gi|392765192|gb|EJA21981.1|](http://10.200.41.11/mascot/cgi/master_results.pl?file=..%2Fdata%2F20160418%2FF080005.dat#Hit16)** | phage immunity repressor protein [Salmonella enterica subsp. enterica serovar Newport str. CVM 19449] |

| **1.** | [gi|392616945|gb|EIW99373.1|](http://10.200.41.11/mascot/cgi/protein_view.pl?file=..%2Fdata%2F20160418%2FF080005.dat&hit=gi%7C392616945%7Cgb%7CEIW99373.1%7C&db_idx=1&px=1&ave_thresh=33&_ignoreionsscorebelow=0&report=0&_sigthreshold=0.05&_msresflags=1089&_msresflags2=2&percolate=-1&percolate_rt=0&_minpeplen=5&sessionID=all_secdisabledsession)    **Mass:** 42957    **Score:** 332    **Matches:** 19(11)  **Sequences:** 6(4)  **emPAI:** 0.74 |
| --- | --- |
|  | pathogenicity island 1 effector protein SipC [Salmonella enterica subsp. enterica serovar Newport str. Levine 15] |

|  | **Query** | **Observed** | **Mr(expt)** | **Mr(calc)** | **ppm** | **Miss** | **Score** | **Expect** | **Rank** | **Unique** | **Peptide** |
| --- | --- | --- | --- | --- | --- | --- | --- | --- | --- | --- | --- |
|  | [1075](http://10.200.41.11/mascot/cgi/peptide_view.pl?file=..%2Fdata%2F20160418%2FF080005.dat&query=1075&hit=1&index=gi%7C392616945%7Cgb%7CEIW99373.1%7C&db_idx=1&px=1&section=5&ave_thresh=33&_ignoreionsscorebelow=0&report=0&_sigthreshold=0.05&_msresflags=1089&_msresflags2=2&percolate=-1&percolate_rt=0&_minpeplen=5&sessionID=all_secdisabledsession) | **494.7728** | **987.5310** | **987.5237** | **7.41** | **0** | **39** | **0.033** | **1** | **U** | **K.LGAEGVDSLK.S** [1074](http://10.200.41.11/mascot/cgi/peptide_view.pl?file=..%2Fdata%2F20160418%2FF080005.dat&query=1074&hit=1&index=gi%7C392616945%7Cgb%7CEIW99373.1%7C&db_idx=1&px=1&section=5&ave_thresh=33&_ignoreionsscorebelow=0&report=0&_sigthreshold=0.05&_msresflags=1089&_msresflags2=2&percolate=-1&percolate_rt=0&_minpeplen=5&sessionID=all_secdisabledsession) [1076](http://10.200.41.11/mascot/cgi/peptide_view.pl?file=..%2Fdata%2F20160418%2FF080005.dat&query=1076&hit=1&index=gi%7C392616945%7Cgb%7CEIW99373.1%7C&db_idx=1&px=1&section=5&ave_thresh=33&_ignoreionsscorebelow=0&report=0&_sigthreshold=0.05&_msresflags=1089&_msresflags2=2&percolate=-1&percolate_rt=0&_minpeplen=5&sessionID=all_secdisabledsession) [1077](http://10.200.41.11/mascot/cgi/peptide_view.pl?file=..%2Fdata%2F20160418%2FF080005.dat&query=1077&hit=1&index=gi%7C392616945%7Cgb%7CEIW99373.1%7C&db_idx=1&px=1&section=5&ave_thresh=33&_ignoreionsscorebelow=0&report=0&_sigthreshold=0.05&_msresflags=1089&_msresflags2=2&percolate=-1&percolate_rt=0&_minpeplen=5&sessionID=all_secdisabledsession) |
|  | [1342](http://10.200.41.11/mascot/cgi/peptide_view.pl?file=..%2Fdata%2F20160418%2FF080005.dat&query=1342&hit=1&index=gi%7C392616945%7Cgb%7CEIW99373.1%7C&db_idx=1&px=1&section=5&ave_thresh=33&_ignoreionsscorebelow=0&report=0&_sigthreshold=0.05&_msresflags=1089&_msresflags2=2&percolate=-1&percolate_rt=0&_minpeplen=5&sessionID=all_secdisabledsession) | **519.2539** | **1036.4933** | **1036.4859** | **7.11** | **0** | **41** | **0.0085** | **1** | **U** | **K.TMESINQSK.A** [1341](http://10.200.41.11/mascot/cgi/peptide_view.pl?file=..%2Fdata%2F20160418%2FF080005.dat&query=1341&hit=1&index=gi%7C392616945%7Cgb%7CEIW99373.1%7C&db_idx=1&px=1&section=5&ave_thresh=33&_ignoreionsscorebelow=0&report=0&_sigthreshold=0.05&_msresflags=1089&_msresflags2=2&percolate=-1&percolate_rt=0&_minpeplen=5&sessionID=all_secdisabledsession) [1343](http://10.200.41.11/mascot/cgi/peptide_view.pl?file=..%2Fdata%2F20160418%2FF080005.dat&query=1343&hit=1&index=gi%7C392616945%7Cgb%7CEIW99373.1%7C&db_idx=1&px=1&section=5&ave_thresh=33&_ignoreionsscorebelow=0&report=0&_sigthreshold=0.05&_msresflags=1089&_msresflags2=2&percolate=-1&percolate_rt=0&_minpeplen=5&sessionID=all_secdisabledsession) |
|  | [2519](http://10.200.41.11/mascot/cgi/peptide_view.pl?file=..%2Fdata%2F20160418%2FF080005.dat&query=2519&hit=1&index=gi%7C392616945%7Cgb%7CEIW99373.1%7C&db_idx=1&px=1&section=5&ave_thresh=33&_ignoreionsscorebelow=0&report=0&_sigthreshold=0.05&_msresflags=1089&_msresflags2=2&percolate=-1&percolate_rt=0&_minpeplen=5&sessionID=all_secdisabledsession) | **651.8276** | **1301.6407** | **1301.6324** | **6.42** | **0** | **62** | **8.6e-005** | **1** | **U** | **R.SEQQISQVNNR.V** [2520](http://10.200.41.11/mascot/cgi/peptide_view.pl?file=..%2Fdata%2F20160418%2FF080005.dat&query=2520&hit=1&index=gi%7C392616945%7Cgb%7CEIW99373.1%7C&db_idx=1&px=1&section=5&ave_thresh=33&_ignoreionsscorebelow=0&report=0&_sigthreshold=0.05&_msresflags=1089&_msresflags2=2&percolate=-1&percolate_rt=0&_minpeplen=5&sessionID=all_secdisabledsession) [2521](http://10.200.41.11/mascot/cgi/peptide_view.pl?file=..%2Fdata%2F20160418%2FF080005.dat&query=2521&hit=1&index=gi%7C392616945%7Cgb%7CEIW99373.1%7C&db_idx=1&px=1&section=5&ave_thresh=33&_ignoreionsscorebelow=0&report=0&_sigthreshold=0.05&_msresflags=1089&_msresflags2=2&percolate=-1&percolate_rt=0&_minpeplen=5&sessionID=all_secdisabledsession) [2522](http://10.200.41.11/mascot/cgi/peptide_view.pl?file=..%2Fdata%2F20160418%2FF080005.dat&query=2522&hit=1&index=gi%7C392616945%7Cgb%7CEIW99373.1%7C&db_idx=1&px=1&section=5&ave_thresh=33&_ignoreionsscorebelow=0&report=0&_sigthreshold=0.05&_msresflags=1089&_msresflags2=2&percolate=-1&percolate_rt=0&_minpeplen=5&sessionID=all_secdisabledsession) [2523](http://10.200.41.11/mascot/cgi/peptide_view.pl?file=..%2Fdata%2F20160418%2FF080005.dat&query=2523&hit=1&index=gi%7C392616945%7Cgb%7CEIW99373.1%7C&db_idx=1&px=1&section=5&ave_thresh=33&_ignoreionsscorebelow=0&report=0&_sigthreshold=0.05&_msresflags=1089&_msresflags2=2&percolate=-1&percolate_rt=0&_minpeplen=5&sessionID=all_secdisabledsession) |
|  | [2923](http://10.200.41.11/mascot/cgi/peptide_view.pl?file=..%2Fdata%2F20160418%2FF080005.dat&query=2923&hit=1&index=gi%7C392616945%7Cgb%7CEIW99373.1%7C&db_idx=1&px=1&section=5&ave_thresh=33&_ignoreionsscorebelow=0&report=0&_sigthreshold=0.05&_msresflags=1089&_msresflags2=2&percolate=-1&percolate_rt=0&_minpeplen=5&sessionID=all_secdisabledsession) | **733.3522** | **1464.6899** | **1464.6804** | **6.47** | **1** | **41** | **0.01** | **1** | **U** | **R.VASTASDEARESSR.K** |
|  | [2925](http://10.200.41.11/mascot/cgi/peptide_view.pl?file=..%2Fdata%2F20160418%2FF080005.dat&query=2925&hit=1&index=gi%7C392616945%7Cgb%7CEIW99373.1%7C&db_idx=1&px=1&section=5&ave_thresh=33&_ignoreionsscorebelow=0&report=0&_sigthreshold=0.05&_msresflags=1089&_msresflags2=2&percolate=-1&percolate_rt=0&_minpeplen=5&sessionID=all_secdisabledsession) | **489.2379** | **1464.6919** | **1464.6804** | **7.84** | **1** | **(10)** | **14** | **1** | **U** | **R.VASTASDEARESSR.K** [2924](http://10.200.41.11/mascot/cgi/peptide_view.pl?file=..%2Fdata%2F20160418%2FF080005.dat&query=2924&hit=1&index=gi%7C392616945%7Cgb%7CEIW99373.1%7C&db_idx=1&px=1&section=5&ave_thresh=33&_ignoreionsscorebelow=0&report=0&_sigthreshold=0.05&_msresflags=1089&_msresflags2=2&percolate=-1&percolate_rt=0&_minpeplen=5&sessionID=all_secdisabledsession) |
|  | [3030](http://10.200.41.11/mascot/cgi/peptide_view.pl?file=..%2Fdata%2F20160418%2FF080005.dat&query=3030&hit=1&index=gi%7C392616945%7Cgb%7CEIW99373.1%7C&db_idx=1&px=1&section=5&ave_thresh=33&_ignoreionsscorebelow=0&report=0&_sigthreshold=0.05&_msresflags=1089&_msresflags2=2&percolate=-1&percolate_rt=0&_minpeplen=5&sessionID=all_secdisabledsession) | **521.6090** | **1561.8052** | **1561.7948** | **6.69** | **1** | **14** | **10** | **1** | **U** | **K.TGTDATKNLNDATLK.S** [3031](http://10.200.41.11/mascot/cgi/peptide_view.pl?file=..%2Fdata%2F20160418%2FF080005.dat&query=3031&hit=1&index=gi%7C392616945%7Cgb%7CEIW99373.1%7C&db_idx=1&px=1&section=5&ave_thresh=33&_ignoreionsscorebelow=0&report=0&_sigthreshold=0.05&_msresflags=1089&_msresflags2=2&percolate=-1&percolate_rt=0&_minpeplen=5&sessionID=all_secdisabledsession) |
|  | [3060](http://10.200.41.11/mascot/cgi/peptide_view.pl?file=..%2Fdata%2F20160418%2FF080005.dat&query=3060&hit=1&index=gi%7C392616945%7Cgb%7CEIW99373.1%7C&db_idx=1&px=1&section=5&ave_thresh=33&_ignoreionsscorebelow=0&report=0&_sigthreshold=0.05&_msresflags=1089&_msresflags2=2&percolate=-1&percolate_rt=0&_minpeplen=5&sessionID=all_secdisabledsession) | **531.9362** | **1592.7868** | **1592.7754** | **7.18** | **2** | **20** | **1.7** | **1** | **U** | **R.VASTASDEARESSRK.S** [3059](http://10.200.41.11/mascot/cgi/peptide_view.pl?file=..%2Fdata%2F20160418%2FF080005.dat&query=3059&hit=1&index=gi%7C392616945%7Cgb%7CEIW99373.1%7C&db_idx=1&px=1&section=5&ave_thresh=33&_ignoreionsscorebelow=0&report=0&_sigthreshold=0.05&_msresflags=1089&_msresflags2=2&percolate=-1&percolate_rt=0&_minpeplen=5&sessionID=all_secdisabledsession) |

**Protein sequence coverage: 14%**

Matched peptides shown in ***bold red***.

| **1** | MLISNVGINP | AAYLNNHSVE | NSSQTASQSV | SAKDILNSIG | ISSSKVSDLG |
| --- | --- | --- | --- | --- | --- |
| **51** | LSPTLSAPAP | GVLTQTPGTI | TSFLKASIQN | TDMNQDLNAL | ANNVTTKANE |
| **101** | VVQTQLREQQ | AEVGKFFDIS | GMSSSAVALL | AAANTLMLTL | NQADSKLSGK |
| **151** | LSLVSFDAAK | TTASSMMREG | MNALSGSISQ | SALQLGITGV | GAKLEYKGLQ |
| **201** | NERGALKHNA | AKIDKLTTES | HSIKNVLNGQ | NSVK**LGAEGV** | **DSLK**SLNMKK |
| **251** | **TGTDATKNLN** | **DATLK**SNAGT | SATESLGIKD | SNKQISPEHQ | AILSKRLESV |
| **301** | ESDIRLEQNT | MDMTRIDARK | MQMTGDLIMK | NSVTVGGIAG | ASGQYAATQE |
| **351** | R**SEQQISQVN** | **NRVASTASDE** | **ARESSRK**STS | LIQEMLK**TME** | **SINQSK**ASAL |
| **401** | AAIAGNIRA |  |  |  |  |

| **2.** | [gi|392616944|gb|EIW99372.1|](http://10.200.41.11/mascot/cgi/protein_view.pl?file=..%2Fdata%2F20160418%2FF080005.dat&hit=gi%7C392616944%7Cgb%7CEIW99372.1%7C&db_idx=1&px=1&ave_thresh=33&_ignoreionsscorebelow=0&report=0&_sigthreshold=0.05&_msresflags=1089&_msresflags2=2&percolate=-1&percolate_rt=0&_minpeplen=5&sessionID=all_secdisabledsession)    **Mass:** 37081    **Score:** 192    **Matches:** 4(4)  **Sequences:** 1(1)  **emPAI:** 0.17 |
| --- | --- |
|  | cell invasion protein SipD [Salmonella enterica subsp. enterica serovar Newport str. Levine 15] |

|  | **Query** | **Observed** | **Mr(expt)** | **Mr(calc)** | **ppm** | **Miss** | **Score** | **Expect** | **Rank** | **Unique** | **Peptide** |
| --- | --- | --- | --- | --- | --- | --- | --- | --- | --- | --- | --- |
|  | [2940](http://10.200.41.11/mascot/cgi/peptide_view.pl?file=..%2Fdata%2F20160418%2FF080005.dat&query=2940&hit=1&index=gi%7C392616944%7Cgb%7CEIW99372.1%7C&db_idx=1&px=1&section=5&ave_thresh=33&_ignoreionsscorebelow=0&report=0&_sigthreshold=0.05&_msresflags=1089&_msresflags2=2&percolate=-1&percolate_rt=0&_minpeplen=5&sessionID=all_secdisabledsession) | **752.3784** | **1502.7422** | **1502.7325** | **6.45** | **0** | **64** | **5.8e-005** | **1** | **U** | **K.SGVSLSAEQNENLR.S** [2938](http://10.200.41.11/mascot/cgi/peptide_view.pl?file=..%2Fdata%2F20160418%2FF080005.dat&query=2938&hit=1&index=gi%7C392616944%7Cgb%7CEIW99372.1%7C&db_idx=1&px=1&section=5&ave_thresh=33&_ignoreionsscorebelow=0&report=0&_sigthreshold=0.05&_msresflags=1089&_msresflags2=2&percolate=-1&percolate_rt=0&_minpeplen=5&sessionID=all_secdisabledsession) [2939](http://10.200.41.11/mascot/cgi/peptide_view.pl?file=..%2Fdata%2F20160418%2FF080005.dat&query=2939&hit=1&index=gi%7C392616944%7Cgb%7CEIW99372.1%7C&db_idx=1&px=1&section=5&ave_thresh=33&_ignoreionsscorebelow=0&report=0&_sigthreshold=0.05&_msresflags=1089&_msresflags2=2&percolate=-1&percolate_rt=0&_minpeplen=5&sessionID=all_secdisabledsession) [2941](http://10.200.41.11/mascot/cgi/peptide_view.pl?file=..%2Fdata%2F20160418%2FF080005.dat&query=2941&hit=1&index=gi%7C392616944%7Cgb%7CEIW99372.1%7C&db_idx=1&px=1&section=5&ave_thresh=33&_ignoreionsscorebelow=0&report=0&_sigthreshold=0.05&_msresflags=1089&_msresflags2=2&percolate=-1&percolate_rt=0&_minpeplen=5&sessionID=all_secdisabledsession) |

**Protein sequence coverage: 4%**

Matched peptides shown in ***bold red***.

| **1** | MLNIQNYSAS | PHPGIVAERP | QTPSASEHVE | TAVVPSTTEH | RGTDIISLSQ |
| --- | --- | --- | --- | --- | --- |
| **51** | AATKIQQAQQ | TLQSTPPISE | ENNDERTLAR | QQLTSSLNAL | AK**SGVSLSAE** |
| **101** | **QNENLR**SAFS | APTSALFSAS | PMAQPRTTIS | DAEIWDMVSQ | NISAIGDSYL |
| **151** | GVYENVVAVY | TDFYQAFSDI | LSKMGGWLLP | GKDGNTVKLD | VTSLKNDLNS |
| **201** | LVNKYNQINS | NTVLFPAQSG | SGVKVATEAE | ARQWLSELNL | PNSCLKSYGS |
| **251** | GYVVTVDLTP | LQKMVQDIDG | LGAPGKDSKL | EMDNAKYQAW | QSGFKAQEEN |
| **301** | MKTTLQTLTQ | KYSNANSLYD | NLVKVLSSTI | SSSLETAKSF | LQG |

| **3.** | [gi|194403331|gb|ACF63553.1|](http://10.200.41.11/mascot/cgi/protein_view.pl?file=..%2Fdata%2F20160418%2FF080005.dat&hit=gi%7C194403331%7Cgb%7CACF63553.1%7C&db_idx=1&px=1&ave_thresh=33&_ignoreionsscorebelow=0&report=0&_sigthreshold=0.05&_msresflags=1089&_msresflags2=2&percolate=-1&percolate_rt=0&_minpeplen=5&sessionID=all_secdisabledsession)    **Mass:** 10561    **Score:** 188    **Matches:** 3(3)  **Sequences:** 1(1)  **emPAI:** 0.71 |
| --- | --- |
|  | negative regulator of flagellin synthesis [Salmonella enterica subsp. enterica serovar Newport str. SL254] |

|  | **Query** | **Observed** | **Mr(expt)** | **Mr(calc)** | **ppm** | **Miss** | **Score** | **Expect** | **Rank** | **Unique** | **Peptide** |
| --- | --- | --- | --- | --- | --- | --- | --- | --- | --- | --- | --- |
|  | [3063](http://10.200.41.11/mascot/cgi/peptide_view.pl?file=..%2Fdata%2F20160418%2FF080005.dat&query=3063&hit=1&index=gi%7C194403331%7Cgb%7CACF63553.1%7C&db_idx=1&px=1&section=5&ave_thresh=33&_ignoreionsscorebelow=0&report=0&_sigthreshold=0.05&_msresflags=1089&_msresflags2=2&percolate=-1&percolate_rt=0&_minpeplen=5&sessionID=all_secdisabledsession) | **804.9117** | **1607.8088** | **1607.8002** | **5.34** | **0** | **98** | **2.6e-008** | **1** | **U** | **K.TSAATSASVTLSDAQAK.L** [3064](http://10.200.41.11/mascot/cgi/peptide_view.pl?file=..%2Fdata%2F20160418%2FF080005.dat&query=3064&hit=1&index=gi%7C194403331%7Cgb%7CACF63553.1%7C&db_idx=1&px=1&section=5&ave_thresh=33&_ignoreionsscorebelow=0&report=0&_sigthreshold=0.05&_msresflags=1089&_msresflags2=2&percolate=-1&percolate_rt=0&_minpeplen=5&sessionID=all_secdisabledsession) [3065](http://10.200.41.11/mascot/cgi/peptide_view.pl?file=..%2Fdata%2F20160418%2FF080005.dat&query=3065&hit=1&index=gi%7C194403331%7Cgb%7CACF63553.1%7C&db_idx=1&px=1&section=5&ave_thresh=33&_ignoreionsscorebelow=0&report=0&_sigthreshold=0.05&_msresflags=1089&_msresflags2=2&percolate=-1&percolate_rt=0&_minpeplen=5&sessionID=all_secdisabledsession) |

**Protein sequence coverage: 17%**

Matched peptides shown in ***bold red***.

| **1** | MSIDRTSPLK | PVSTVQTRET | SDTPVQKTRQ | EK**TSAATSAS** | **VTLSDAQAK**L |
| --- | --- | --- | --- | --- | --- |
| **51** | MQPGVSDINM | ERVEALKTAI | RNGELKMDTG | KIADSLIREA | QSYLQSK |

| **4.** | [gi|50830890|gb|AAT81610.1|](http://10.200.41.11/mascot/cgi/protein_view.pl?file=..%2Fdata%2F20160418%2FF080005.dat&hit=gi%7C50830890%7Cgb%7CAAT81610.1%7C&db_idx=1&px=1&ave_thresh=33&_ignoreionsscorebelow=0&report=0&_sigthreshold=0.05&_msresflags=1089&_msresflags2=2&percolate=-1&percolate_rt=0&_minpeplen=5&sessionID=all_secdisabledsession)    **Mass:** 52223    **Score:** 136    **Matches:** 12(4)  **Sequences:** 3(1)  **emPAI:** 0.12 |
| --- | --- |
|  | phase 1 flagellin [Salmonella enterica subsp. enterica serovar Newport] |

|  | **Query** | **Observed** | **Mr(expt)** | **Mr(calc)** | **ppm** | **Miss** | **Score** | **Expect** | **Rank** | **Unique** | **Peptide** |
| --- | --- | --- | --- | --- | --- | --- | --- | --- | --- | --- | --- |
|  | [1739](http://10.200.41.11/mascot/cgi/peptide_view.pl?file=..%2Fdata%2F20160418%2FF080005.dat&query=1739&hit=1&index=gi%7C50830890%7Cgb%7CAAT81610.1%7C&db_idx=1&px=1&section=5&ave_thresh=33&_ignoreionsscorebelow=0&report=0&_sigthreshold=0.05&_msresflags=1089&_msresflags2=2&percolate=-1&percolate_rt=0&_minpeplen=5&sessionID=all_secdisabledsession) | **566.8055** | **1131.5965** | **1131.5884** | **7.20** | **0** | **48** | **0.003** | **1** |  | **K.SQSALGTAIER.L** [1737](http://10.200.41.11/mascot/cgi/peptide_view.pl?file=..%2Fdata%2F20160418%2FF080005.dat&query=1737&hit=1&index=gi%7C50830890%7Cgb%7CAAT81610.1%7C&db_idx=1&px=1&section=5&ave_thresh=33&_ignoreionsscorebelow=0&report=0&_sigthreshold=0.05&_msresflags=1089&_msresflags2=2&percolate=-1&percolate_rt=0&_minpeplen=5&sessionID=all_secdisabledsession) [1738](http://10.200.41.11/mascot/cgi/peptide_view.pl?file=..%2Fdata%2F20160418%2FF080005.dat&query=1738&hit=1&index=gi%7C50830890%7Cgb%7CAAT81610.1%7C&db_idx=1&px=1&section=5&ave_thresh=33&_ignoreionsscorebelow=0&report=0&_sigthreshold=0.05&_msresflags=1089&_msresflags2=2&percolate=-1&percolate_rt=0&_minpeplen=5&sessionID=all_secdisabledsession) [1740](http://10.200.41.11/mascot/cgi/peptide_view.pl?file=..%2Fdata%2F20160418%2FF080005.dat&query=1740&hit=1&index=gi%7C50830890%7Cgb%7CAAT81610.1%7C&db_idx=1&px=1&section=5&ave_thresh=33&_ignoreionsscorebelow=0&report=0&_sigthreshold=0.05&_msresflags=1089&_msresflags2=2&percolate=-1&percolate_rt=0&_minpeplen=5&sessionID=all_secdisabledsession) |
|  | [3066](http://10.200.41.11/mascot/cgi/peptide_view.pl?file=..%2Fdata%2F20160418%2FF080005.dat&query=3066&hit=1&index=gi%7C50830890%7Cgb%7CAAT81610.1%7C&db_idx=1&px=1&section=5&ave_thresh=33&_ignoreionsscorebelow=0&report=0&_sigthreshold=0.05&_msresflags=1089&_msresflags2=2&percolate=-1&percolate_rt=0&_minpeplen=5&sessionID=all_secdisabledsession) | **538.9487** | **1613.8244** | **1613.8121** | **7.60** | **1** | **34** | **0.085** | **1** |  | **R.INSAKDDAAGQAIANR.F** [3067](http://10.200.41.11/mascot/cgi/peptide_view.pl?file=..%2Fdata%2F20160418%2FF080005.dat&query=3067&hit=1&index=gi%7C50830890%7Cgb%7CAAT81610.1%7C&db_idx=1&px=1&section=5&ave_thresh=33&_ignoreionsscorebelow=0&report=0&_sigthreshold=0.05&_msresflags=1089&_msresflags2=2&percolate=-1&percolate_rt=0&_minpeplen=5&sessionID=all_secdisabledsession) |
|  | [3072](http://10.200.41.11/mascot/cgi/peptide_view.pl?file=..%2Fdata%2F20160418%2FF080005.dat&query=3072&hit=1&index=gi%7C50830890%7Cgb%7CAAT81610.1%7C&db_idx=1&px=1&section=5&ave_thresh=33&_ignoreionsscorebelow=0&report=0&_sigthreshold=0.05&_msresflags=1089&_msresflags2=2&percolate=-1&percolate_rt=0&_minpeplen=5&sessionID=all_secdisabledsession) | **539.9374** | **1616.7905** | **1616.8006** | **-6.22** | **1** | **2** | **95** | **1** | **U** | **K.KALEDGGVSNADATAAK.L** [3068](http://10.200.41.11/mascot/cgi/peptide_view.pl?file=..%2Fdata%2F20160418%2FF080005.dat&query=3068&hit=1&index=gi%7C50830890%7Cgb%7CAAT81610.1%7C&db_idx=1&px=1&section=5&ave_thresh=33&_ignoreionsscorebelow=0&report=0&_sigthreshold=0.05&_msresflags=1089&_msresflags2=2&percolate=-1&percolate_rt=0&_minpeplen=5&sessionID=all_secdisabledsession) [3070](http://10.200.41.11/mascot/cgi/peptide_view.pl?file=..%2Fdata%2F20160418%2FF080005.dat&query=3070&hit=1&index=gi%7C50830890%7Cgb%7CAAT81610.1%7C&db_idx=1&px=1&section=5&ave_thresh=33&_ignoreionsscorebelow=0&report=0&_sigthreshold=0.05&_msresflags=1089&_msresflags2=2&percolate=-1&percolate_rt=0&_minpeplen=5&sessionID=all_secdisabledsession) [3071](http://10.200.41.11/mascot/cgi/peptide_view.pl?file=..%2Fdata%2F20160418%2FF080005.dat&query=3071&hit=1&index=gi%7C50830890%7Cgb%7CAAT81610.1%7C&db_idx=1&px=1&section=5&ave_thresh=33&_ignoreionsscorebelow=0&report=0&_sigthreshold=0.05&_msresflags=1089&_msresflags2=2&percolate=-1&percolate_rt=0&_minpeplen=5&sessionID=all_secdisabledsession) [3074](http://10.200.41.11/mascot/cgi/peptide_view.pl?file=..%2Fdata%2F20160418%2FF080005.dat&query=3074&hit=1&index=gi%7C50830890%7Cgb%7CAAT81610.1%7C&db_idx=1&px=1&section=5&ave_thresh=33&_ignoreionsscorebelow=0&report=0&_sigthreshold=0.05&_msresflags=1089&_msresflags2=2&percolate=-1&percolate_rt=0&_minpeplen=5&sessionID=all_secdisabledsession) [3076](http://10.200.41.11/mascot/cgi/peptide_view.pl?file=..%2Fdata%2F20160418%2FF080005.dat&query=3076&hit=1&index=gi%7C50830890%7Cgb%7CAAT81610.1%7C&db_idx=1&px=1&section=5&ave_thresh=33&_ignoreionsscorebelow=0&report=0&_sigthreshold=0.05&_msresflags=1089&_msresflags2=2&percolate=-1&percolate_rt=0&_minpeplen=5&sessionID=all_secdisabledsession) |

### Protein sequence coverage: 8%

Matched peptides shown in ***bold red***.

| **1** | MAQVINTNSL | SLLTQNNLNK | **SQSALGTAIE** | **R**LSSGLR**INS** | **AKDDAAGQAI** |
| --- | --- | --- | --- | --- | --- |
| **51** | **ANR**FTANIKG | LTQASRNAND | GISIAQTTEG | ALNEINNNLQ | RVRELAVQSA |
| **101** | NSTNSQSDLD | SIQAEITQRL | NEIDRVSGQT | QFNGVKVLAQ | DNTLTIQVGA |
| **151** | NDGETIDIDL | KQINSQTLGL | DTLNVQKAYD | VSATAAMDPK | SFTDGTKNLT |
| **201** | APDATAIKAA | LGNPAATGDS | LSATLSFKDG | KYYATVAGYT | NAADTSKNGK |
| **251** | YEVNVDSATG | AVTFNAAPTK | ATVTGDTTVT | KVQVNAPVAV | STDVK**KALED** |
| **301** | **GGVSNADATA** | **AK**LVKMSYTD | KNGKSIDGGY | ALEAGGKYYA | ATYDEGTGKI |
| **351** | TANVTTYTDS | TGVTKTAANQ | LGGVDGKTEV | VTIDGKTYNA | SKAAGHDFKA |
| **401** | QPELAEAAAK | TTENPLAKID | AALAQVDALR | SDLGAVQNRF | NSAITNLGNT |
| **451** | VNNLSEARSR | IEDSDYATEV | SNMSRAQILQ | QAGTSVLAQA | NQVPQNVLSL |
| **501** | LR |  |  |  |  |

| **5.** | [gi|459466347|gb|EMG61125.1|](http://10.200.41.11/mascot/cgi/protein_view.pl?file=..%2Fdata%2F20160418%2FF080005.dat&hit=gi%7C459466347%7Cgb%7CEMG61125.1%7C&db_idx=1&px=1&ave_thresh=33&_ignoreionsscorebelow=0&report=0&_sigthreshold=0.05&_msresflags=1089&_msresflags2=2&percolate=-1&percolate_rt=0&_minpeplen=5&sessionID=all_secdisabledsession)    **Mass:** 8447     **Score:** 136    **Matches:** 7(4)  **Sequences:** 3(1)  **emPAI:** 0.93 |
| --- | --- |
|  | Flagellar biosynthesis protein FliC, partial [Salmonella enterica subsp. enterica serovar Newport str. SH111077] |

|  | **Query** | **Observed** | **Mr(expt)** | **Mr(calc)** | **ppm** | **Miss** | **Score** | **Expect** | **Rank** | **Unique** | **Peptide** |
| --- | --- | --- | --- | --- | --- | --- | --- | --- | --- | --- | --- |
|  | [1309](http://10.200.41.11/mascot/cgi/peptide_view.pl?file=..%2Fdata%2F20160418%2FF080005.dat&query=1309&hit=1&index=gi%7C459466347%7Cgb%7CEMG61125.1%7C&db_idx=1&px=1&section=5&ave_thresh=33&_ignoreionsscorebelow=0&report=0&_sigthreshold=0.05&_msresflags=1089&_msresflags2=2&percolate=-1&percolate_rt=0&_minpeplen=5&sessionID=all_secdisabledsession) | **516.2648** | **1030.5151** | **1030.5155** | **-0.42** | **1** | **0** | **1.5e+002** | **1** | **U** | **K.GLTQASRNAN.-** |
|  | [1739](http://10.200.41.11/mascot/cgi/peptide_view.pl?file=..%2Fdata%2F20160418%2FF080005.dat&query=1739&hit=1&index=gi%7C459466347%7Cgb%7CEMG61125.1%7C&db_idx=1&px=1&section=5&ave_thresh=33&_ignoreionsscorebelow=0&report=0&_sigthreshold=0.05&_msresflags=1089&_msresflags2=2&percolate=-1&percolate_rt=0&_minpeplen=5&sessionID=all_secdisabledsession) | 566.8055 | 1131.5965 | 1131.5884 | 7.20 | 0 | 48 | 0.003 | 1 |  | K.SQSALGTAIER.L [1737](http://10.200.41.11/mascot/cgi/peptide_view.pl?file=..%2Fdata%2F20160418%2FF080005.dat&query=1737&hit=1&index=gi%7C459466347%7Cgb%7CEMG61125.1%7C&db_idx=1&px=1&section=5&ave_thresh=33&_ignoreionsscorebelow=0&report=0&_sigthreshold=0.05&_msresflags=1089&_msresflags2=2&percolate=-1&percolate_rt=0&_minpeplen=5&sessionID=all_secdisabledsession) [1738](http://10.200.41.11/mascot/cgi/peptide_view.pl?file=..%2Fdata%2F20160418%2FF080005.dat&query=1738&hit=1&index=gi%7C459466347%7Cgb%7CEMG61125.1%7C&db_idx=1&px=1&section=5&ave_thresh=33&_ignoreionsscorebelow=0&report=0&_sigthreshold=0.05&_msresflags=1089&_msresflags2=2&percolate=-1&percolate_rt=0&_minpeplen=5&sessionID=all_secdisabledsession) [1740](http://10.200.41.11/mascot/cgi/peptide_view.pl?file=..%2Fdata%2F20160418%2FF080005.dat&query=1740&hit=1&index=gi%7C459466347%7Cgb%7CEMG61125.1%7C&db_idx=1&px=1&section=5&ave_thresh=33&_ignoreionsscorebelow=0&report=0&_sigthreshold=0.05&_msresflags=1089&_msresflags2=2&percolate=-1&percolate_rt=0&_minpeplen=5&sessionID=all_secdisabledsession) |
|  | [3066](http://10.200.41.11/mascot/cgi/peptide_view.pl?file=..%2Fdata%2F20160418%2FF080005.dat&query=3066&hit=1&index=gi%7C459466347%7Cgb%7CEMG61125.1%7C&db_idx=1&px=1&section=5&ave_thresh=33&_ignoreionsscorebelow=0&report=0&_sigthreshold=0.05&_msresflags=1089&_msresflags2=2&percolate=-1&percolate_rt=0&_minpeplen=5&sessionID=all_secdisabledsession) | 538.9487 | 1613.8244 | 1613.8121 | 7.60 | 1 | 34 | 0.085 | 1 |  | R.INSAKDDAAGQAIANR.F [3067](http://10.200.41.11/mascot/cgi/peptide_view.pl?file=..%2Fdata%2F20160418%2FF080005.dat&query=3067&hit=1&index=gi%7C459466347%7Cgb%7CEMG61125.1%7C&db_idx=1&px=1&section=5&ave_thresh=33&_ignoreionsscorebelow=0&report=0&_sigthreshold=0.05&_msresflags=1089&_msresflags2=2&percolate=-1&percolate_rt=0&_minpeplen=5&sessionID=all_secdisabledsession) |

### Protein sequence coverage: 46%

Matched peptides shown in ***bold red***.

| **1** | LSKTFQKENF | MAQVINTNSL | SLLTQNNLNK | **SQSALGTAIE** | **R**LSSGLR**INS** |
| --- | --- | --- | --- | --- | --- |
| **51** | **AKDDAAGQAI** | **ANR**FTANIK**G** | **LTQASRNAN** |  |  |

| **8.** | [gi|194402702|gb|ACF62924.1|](http://10.200.41.11/mascot/cgi/protein_view.pl?file=..%2Fdata%2F20160418%2FF080005.dat&hit=gi%7C194402702%7Cgb%7CACF62924.1%7C&db_idx=1&px=1&ave_thresh=33&_ignoreionsscorebelow=0&report=0&_sigthreshold=0.05&_msresflags=1089&_msresflags2=2&percolate=-1&percolate_rt=0&_minpeplen=5&sessionID=all_secdisabledsession)    **Mass:** 72333    **Score:** 89     **Matches:** 7(5)  **Sequences:** 2(2)  **emPAI:** 0.18 |
| --- | --- |
|  | cell invasion protein SipA [Salmonella enterica subsp. enterica serovar Newport str. SL254] |

|  | **Query** | **Observed** | **Mr(expt)** | **Mr(calc)** | **ppm** | **Miss** | **Score** | **Expect** | **Rank** | **Unique** | **Peptide** |
| --- | --- | --- | --- | --- | --- | --- | --- | --- | --- | --- | --- |
|  | [998](http://10.200.41.11/mascot/cgi/peptide_view.pl?file=..%2Fdata%2F20160418%2FF080005.dat&query=998&hit=1&index=gi%7C194402702%7Cgb%7CACF62924.1%7C&db_idx=1&px=1&section=5&ave_thresh=33&_ignoreionsscorebelow=0&report=0&_sigthreshold=0.05&_msresflags=1089&_msresflags2=2&percolate=-1&percolate_rt=0&_minpeplen=5&sessionID=all_secdisabledsession) | **490.7471** | **979.4797** | **979.4723** | **7.56** | **0** | **37** | **0.034** | **1** | **U** | **R.TFIDNSQR.N** [996](http://10.200.41.11/mascot/cgi/peptide_view.pl?file=..%2Fdata%2F20160418%2FF080005.dat&query=996&hit=1&index=gi%7C194402702%7Cgb%7CACF62924.1%7C&db_idx=1&px=1&section=5&ave_thresh=33&_ignoreionsscorebelow=0&report=0&_sigthreshold=0.05&_msresflags=1089&_msresflags2=2&percolate=-1&percolate_rt=0&_minpeplen=5&sessionID=all_secdisabledsession) [997](http://10.200.41.11/mascot/cgi/peptide_view.pl?file=..%2Fdata%2F20160418%2FF080005.dat&query=997&hit=1&index=gi%7C194402702%7Cgb%7CACF62924.1%7C&db_idx=1&px=1&section=5&ave_thresh=33&_ignoreionsscorebelow=0&report=0&_sigthreshold=0.05&_msresflags=1089&_msresflags2=2&percolate=-1&percolate_rt=0&_minpeplen=5&sessionID=all_secdisabledsession) [999](http://10.200.41.11/mascot/cgi/peptide_view.pl?file=..%2Fdata%2F20160418%2FF080005.dat&query=999&hit=1&index=gi%7C194402702%7Cgb%7CACF62924.1%7C&db_idx=1&px=1&section=5&ave_thresh=33&_ignoreionsscorebelow=0&report=0&_sigthreshold=0.05&_msresflags=1089&_msresflags2=2&percolate=-1&percolate_rt=0&_minpeplen=5&sessionID=all_secdisabledsession) [1000](http://10.200.41.11/mascot/cgi/peptide_view.pl?file=..%2Fdata%2F20160418%2FF080005.dat&query=1000&hit=1&index=gi%7C194402702%7Cgb%7CACF62924.1%7C&db_idx=1&px=1&section=5&ave_thresh=33&_ignoreionsscorebelow=0&report=0&_sigthreshold=0.05&_msresflags=1089&_msresflags2=2&percolate=-1&percolate_rt=0&_minpeplen=5&sessionID=all_secdisabledsession) |
|  | [2031](http://10.200.41.11/mascot/cgi/peptide_view.pl?file=..%2Fdata%2F20160418%2FF080005.dat&query=2031&hit=1&index=gi%7C194402702%7Cgb%7CACF62924.1%7C&db_idx=1&px=1&section=5&ave_thresh=33&_ignoreionsscorebelow=0&report=0&_sigthreshold=0.05&_msresflags=1089&_msresflags2=2&percolate=-1&percolate_rt=0&_minpeplen=5&sessionID=all_secdisabledsession) | **588.3083** | **1174.6020** | **1174.5942** | **6.64** | **0** | **44** | **0.0089** | **1** | **U** | **K.LTQEQGTSVGR.E** [2032](http://10.200.41.11/mascot/cgi/peptide_view.pl?file=..%2Fdata%2F20160418%2FF080005.dat&query=2032&hit=1&index=gi%7C194402702%7Cgb%7CACF62924.1%7C&db_idx=1&px=1&section=5&ave_thresh=33&_ignoreionsscorebelow=0&report=0&_sigthreshold=0.05&_msresflags=1089&_msresflags2=2&percolate=-1&percolate_rt=0&_minpeplen=5&sessionID=all_secdisabledsession) |

### Protein sequence coverage: 2%

Matched peptides shown in ***bold red***.

| **1** | MQTEIKTQAT | NLAANLSAVR | ESATTTLSGE | IKGPQLEDFP | ALIKQASLDA |
| --- | --- | --- | --- | --- | --- |
| **51** | LFKCGKDAEA | LKEVFTNSNN | VAGKKAIMEF | AGLFRSALNA | TSDSPEAKTL |
| **101** | LMKVGAEYTA | QIIKDGLKEK | SAFGPWLPET | KKAEAKLENL | EKQLLDIIKN |
| **151** | NTGGELSKLS | TNLVMQEVMP | YIASCIEHNF | GCTLDPLTRS | NLTHLVDKAA |
| **201** | AKAVEALDMC | HQK**LTQEQGT** | **SVGR**EARHLE | MQTLIPLLLR | NVFAQIPADK |
| **251** | LPDPKIPEPA | AGPVPDGGKK | AEPTGININI | NIDSSNHSVD | NSKHINNSRS |
| **301** | HVDNSQRHID | NSNHDNSRKT | IDNSR**TFIDN** | **SQR**NGESHHS | TNSSNVSHSH |
| **351** | SRVDSTTHQT | ETAHSASTGA | IDHGIAGKID | VTAHATAEAV | TNASSESKDG |
| **401** | KVVTSEKGTT | GETTSFDEVD | GVTSKSIIGK | PVQATVHGVD | DNKQQSQTAE |
| **451** | IVNVKPLASQ | LAGVENVKTD | TLQSDTTVIT | GNKAGTTDND | NSQTDKTGPF |
| **501** | SGLKFKQNSF | LSTVPSVTNM | HSMHFDARET | FLGVIRKALE | PDTSTPFPVR |
| **551** | RAFDGLRAEI | LPNDTIKSAA | LKAQCSDIDK | HPELKAKMET | LKEVITHHPQ |
| **601** | KEKLAEIALQ | FAREAGLTRL | KGETDYVLSN | VLDGLIGDGS | WRAGPAYESY |
| **651** | LNKPGVDRVI | TTVDGLHMQR |  |  |  |

| **9.** | [gi|194404219|gb|ACF64441.1|](http://10.200.41.11/mascot/cgi/protein_view.pl?file=..%2Fdata%2F20160418%2FF080005.dat&hit=gi%7C194404219%7Cgb%7CACF64441.1%7C&db_idx=1&px=1&ave_thresh=33&_ignoreionsscorebelow=0&report=0&_sigthreshold=0.05&_msresflags=1089&_msresflags2=2&percolate=-1&percolate_rt=0&_minpeplen=5&sessionID=all_secdisabledsession)    **Mass:** 34155    **Score:** 88     **Matches:** 5(5)  **Sequences:** 2(2)  **emPAI:** 0.41 |
| --- | --- |
|  | flagellar hook-associated protein 3 [Salmonella enterica subsp. enterica serovar Newport str. SL254] |

|  | **Query** | **Observed** | **Mr(expt)** | **Mr(calc)** | **ppm** | **Miss** | **Score** | **Expect** | **Rank** | **Unique** | **Peptide** |
| --- | --- | --- | --- | --- | --- | --- | --- | --- | --- | --- | --- |
|  | [1553](http://10.200.41.11/mascot/cgi/peptide_view.pl?file=..%2Fdata%2F20160418%2FF080005.dat&query=1553&hit=1&index=gi%7C194404219%7Cgb%7CACF64441.1%7C&db_idx=1&px=1&section=5&ave_thresh=33&_ignoreionsscorebelow=0&report=0&_sigthreshold=0.05&_msresflags=1089&_msresflags2=2&percolate=-1&percolate_rt=0&_minpeplen=5&sessionID=all_secdisabledsession) | **545.7817** | **1089.5488** | **1089.5415** | **6.74** | **0** | **41** | **0.013** | **1** | **U** | **K.SVTQQVDSAR.T** [1554](http://10.200.41.11/mascot/cgi/peptide_view.pl?file=..%2Fdata%2F20160418%2FF080005.dat&query=1554&hit=1&index=gi%7C194404219%7Cgb%7CACF64441.1%7C&db_idx=1&px=1&section=5&ave_thresh=33&_ignoreionsscorebelow=0&report=0&_sigthreshold=0.05&_msresflags=1089&_msresflags2=2&percolate=-1&percolate_rt=0&_minpeplen=5&sessionID=all_secdisabledsession) [1555](http://10.200.41.11/mascot/cgi/peptide_view.pl?file=..%2Fdata%2F20160418%2FF080005.dat&query=1555&hit=1&index=gi%7C194404219%7Cgb%7CACF64441.1%7C&db_idx=1&px=1&section=5&ave_thresh=33&_ignoreionsscorebelow=0&report=0&_sigthreshold=0.05&_msresflags=1089&_msresflags2=2&percolate=-1&percolate_rt=0&_minpeplen=5&sessionID=all_secdisabledsession) |
|  | [1664](http://10.200.41.11/mascot/cgi/peptide_view.pl?file=..%2Fdata%2F20160418%2FF080005.dat&query=1664&hit=1&index=gi%7C194404219%7Cgb%7CACF64441.1%7C&db_idx=1&px=1&section=5&ave_thresh=33&_ignoreionsscorebelow=0&report=0&_sigthreshold=0.05&_msresflags=1089&_msresflags2=2&percolate=-1&percolate_rt=0&_minpeplen=5&sessionID=all_secdisabledsession) | **553.7888** | **1105.5631** | **1105.5550** | **7.33** | **1** | **36** | **0.042** | **1** | **U** | **K.LGEQMSTGKR.V** [1663](http://10.200.41.11/mascot/cgi/peptide_view.pl?file=..%2Fdata%2F20160418%2FF080005.dat&query=1663&hit=1&index=gi%7C194404219%7Cgb%7CACF64441.1%7C&db_idx=1&px=1&section=5&ave_thresh=33&_ignoreionsscorebelow=0&report=0&_sigthreshold=0.05&_msresflags=1089&_msresflags2=2&percolate=-1&percolate_rt=0&_minpeplen=5&sessionID=all_secdisabledsession) |

### Protein sequence coverage: 6%

Matched peptides shown in ***bold red***.

| **1** | MRISTQMMYE | QNMSGITNSQ | AEWMK**LGEQM** | **STGKR**VTNPS | DDPIAASQAV |
| --- | --- | --- | --- | --- | --- |
| **51** | VLSQAQAQNS | QYALARTFAT | QKVSLEESVL | SQVTTAIQTA | QEKIVYAGNG |
| **101** | TLSDDDRASL | ATDLQGIRDQ | LMNLANSTDG | NGRYIFAGYK | TEAAPFDQAT |
| **151** | GGYHGGEK**SV** | **TQQVDSAR**TM | VIGHTGAQIF | NSITSNAVPE | PDGSDSEKNL |
| **201** | FVMLDTAIAA | LKTPVEGNDV | EKEKAAAAID | KTNRGLKNSL | NNVLTVRAEL |
| **251** | GTQLSELSTL | DSLGSDRALG | QKLQMSNLVD | VDWNSVISSY | VMQQAALQAS |
| **301** | YKTFTDMQGM | SLFQLNR |  |  |  |

| **11.** | [gi|194403640|gb|ACF63862.1|](http://10.200.41.11/mascot/cgi/protein_view.pl?file=..%2Fdata%2F20160418%2FF080005.dat&hit=gi%7C194403640%7Cgb%7CACF63862.1%7C&db_idx=1&px=1&ave_thresh=33&_ignoreionsscorebelow=0&report=0&_sigthreshold=0.05&_msresflags=1089&_msresflags2=2&percolate=-1&percolate_rt=0&_minpeplen=5&sessionID=all_secdisabledsession)    **Mass:** 62382    **Score:** 66     **Matches:** 5(2)  **Sequences:** 2(1)  **emPAI:** 0.10 |
| --- | --- |
|  | cell invasion protein SipB [Salmonella enterica subsp. enterica serovar Newport str. SL254] |

|  | **Query** | **Observed** | **Mr(expt)** | **Mr(calc)** | **ppm** | **Miss** | **Score** | **Expect** | **Rank** | **Unique** | **Peptide** |
| --- | --- | --- | --- | --- | --- | --- | --- | --- | --- | --- | --- |
|  | [997](http://10.200.41.11/mascot/cgi/peptide_view.pl?file=..%2Fdata%2F20160418%2FF080005.dat&query=997&hit=6&index=gi%7C194403640%7Cgb%7CACF63862.1%7C&db_idx=1&px=1&section=5&ave_thresh=33&_ignoreionsscorebelow=0&report=0&_sigthreshold=0.05&_msresflags=1089&_msresflags2=2&percolate=-1&percolate_rt=0&_minpeplen=5&sessionID=all_secdisabledsession) | 490.7469 | 979.4792 | 979.4909 | -11.96 | 0 | 3 | 89 | 6 | U | K.LFTQGMQR.I [998](http://10.200.41.11/mascot/cgi/peptide_view.pl?file=..%2Fdata%2F20160418%2FF080005.dat&query=998&hit=7&index=gi%7C194403640%7Cgb%7CACF63862.1%7C&db_idx=1&px=1&section=5&ave_thresh=33&_ignoreionsscorebelow=0&report=0&_sigthreshold=0.05&_msresflags=1089&_msresflags2=2&percolate=-1&percolate_rt=0&_minpeplen=5&sessionID=all_secdisabledsession) [1000](http://10.200.41.11/mascot/cgi/peptide_view.pl?file=..%2Fdata%2F20160418%2FF080005.dat&query=1000&hit=7&index=gi%7C194403640%7Cgb%7CACF63862.1%7C&db_idx=1&px=1&section=5&ave_thresh=33&_ignoreionsscorebelow=0&report=0&_sigthreshold=0.05&_msresflags=1089&_msresflags2=2&percolate=-1&percolate_rt=0&_minpeplen=5&sessionID=all_secdisabledsession) |
|  | [1467](http://10.200.41.11/mascot/cgi/peptide_view.pl?file=..%2Fdata%2F20160418%2FF080005.dat&query=1467&hit=1&index=gi%7C194403640%7Cgb%7CACF63862.1%7C&db_idx=1&px=1&section=5&ave_thresh=33&_ignoreionsscorebelow=0&report=0&_sigthreshold=0.05&_msresflags=1089&_msresflags2=2&percolate=-1&percolate_rt=0&_minpeplen=5&sessionID=all_secdisabledsession) | **531.7867** | **1061.5588** | **1061.5505** | **7.80** | **0** | **54** | **0.00077** | **1** | **U** | **R.LAEAAFEGVR.K** [1466](http://10.200.41.11/mascot/cgi/peptide_view.pl?file=..%2Fdata%2F20160418%2FF080005.dat&query=1466&hit=1&index=gi%7C194403640%7Cgb%7CACF63862.1%7C&db_idx=1&px=1&section=5&ave_thresh=33&_ignoreionsscorebelow=0&report=0&_sigthreshold=0.05&_msresflags=1089&_msresflags2=2&percolate=-1&percolate_rt=0&_minpeplen=5&sessionID=all_secdisabledsession) |

### Protein sequence coverage: 3%

Matched peptides shown in ***bold red***.

| **1** | MVNDASSISR | SGYTQNPR**LA** | **EAAFEGVR**KN | TDFLKAADKA | FKDVVATKAG |
| --- | --- | --- | --- | --- | --- |
| **51** | DLKAGTKSGE | SAINTVGLKP | PTDAAREKLS | SEGQLTLLLG | KLMTLLGDVS |
| **101** | LSQLESRLAV | WQAMIESQKE | MGIQVSKEFQ | TALGEAQEAT | DLYEASIKKT |
| **151** | DTAKSVYDAA | AKKLTQAQNK | LQSLDPADPG | YAQAEAAVEQ | AGKEATEAKE |
| **201** | ALDKATDATV | KAGTDAKAKA | EKADNILTKF | QGTANAASQN | QVSQGEQDNL |
| **251** | SNVARLTMLM | AMFIEIVGKN | TEESLQNDLA | LFNALQEGRQ | AEMEKKSAEF |
| **301** | QEETRKAEET | NRIMGCIGKV | LGALLTIVSV | VAAVFTGGAS | LALAAVGLAV |
| **351** | MVADEIVKAA | TGVSFIQQAL | NPIMEHVLKP | LMELIGKAIT | KALEGLGVDK |
| **401** | KTAEMAGSIV | GAIVAAIAMV | AVIVVVAVVG | KGAAAKLGNA | LSKMMGETIK |
| **451** | KLVPNVLKQL | AQNGSK**LFTQ** | **GMQR**ITSGLG | NVGSKMGLQT | NALSKELVGN |
| **501** | TLNKVALGME | VTNTAAQSAG | GVAEGVFIKN | ASEALADFML | ARFAMDQIQQ |
| **551** | WLKQSVEIFG | ENQKVTAELQ | KAMSSAVQQN | ADASRFILRQ | SRA |

| **16.** | [gi|392765192|gb|EJA21981.1|](http://10.200.41.11/mascot/cgi/protein_view.pl?file=..%2Fdata%2F20160418%2FF080005.dat&hit=gi%7C392765192%7Cgb%7CEJA21981.1%7C&db_idx=1&px=1&ave_thresh=33&_ignoreionsscorebelow=0&report=0&_sigthreshold=0.05&_msresflags=1089&_msresflags2=2&percolate=-1&percolate_rt=0&_minpeplen=5&sessionID=all_secdisabledsession)    **Mass:** 21776    **Score:** 20     **Matches:** 12(0)  **Sequences:** 1(0) |
| --- | --- |
|  | phage immunity repressor protein [Salmonella enterica subsp. enterica serovar Newport str. CVM 19449] |

|  | **Query** | **Observed** | **Mr(expt)** | **Mr(calc)** | **ppm** | **Miss** | **Score** | **Expect** | **Rank** | **Unique** | **Peptide** |
| --- | --- | --- | --- | --- | --- | --- | --- | --- | --- | --- | --- |
|  | [87](http://10.200.41.11/mascot/cgi/peptide_view.pl?file=..%2Fdata%2F20160418%2FF080005.dat&query=87&hit=1&index=gi%7C392765192%7Cgb%7CEJA21981.1%7C&db_idx=1&px=1&section=5&ave_thresh=33&_ignoreionsscorebelow=0&report=0&_sigthreshold=0.05&_msresflags=1089&_msresflags2=2&percolate=-1&percolate_rt=0&_minpeplen=5&sessionID=all_secdisabledsession) | **415.7458** | **829.4771** | **829.4770** | **0.14** | **1** | **20** | **4.3** | **1** | **U** | **K.VAAKSAAGR.R** [82](http://10.200.41.11/mascot/cgi/peptide_view.pl?file=..%2Fdata%2F20160418%2FF080005.dat&query=82&hit=4&index=gi%7C392765192%7Cgb%7CEJA21981.1%7C&db_idx=1&px=1&section=5&ave_thresh=33&_ignoreionsscorebelow=0&report=0&_sigthreshold=0.05&_msresflags=1089&_msresflags2=2&percolate=-1&percolate_rt=0&_minpeplen=5&sessionID=all_secdisabledsession) [83](http://10.200.41.11/mascot/cgi/peptide_view.pl?file=..%2Fdata%2F20160418%2FF080005.dat&query=83&hit=1&index=gi%7C392765192%7Cgb%7CEJA21981.1%7C&db_idx=1&px=1&section=5&ave_thresh=33&_ignoreionsscorebelow=0&report=0&_sigthreshold=0.05&_msresflags=1089&_msresflags2=2&percolate=-1&percolate_rt=0&_minpeplen=5&sessionID=all_secdisabledsession) [84](http://10.200.41.11/mascot/cgi/peptide_view.pl?file=..%2Fdata%2F20160418%2FF080005.dat&query=84&hit=4&index=gi%7C392765192%7Cgb%7CEJA21981.1%7C&db_idx=1&px=1&section=5&ave_thresh=33&_ignoreionsscorebelow=0&report=0&_sigthreshold=0.05&_msresflags=1089&_msresflags2=2&percolate=-1&percolate_rt=0&_minpeplen=5&sessionID=all_secdisabledsession) [86](http://10.200.41.11/mascot/cgi/peptide_view.pl?file=..%2Fdata%2F20160418%2FF080005.dat&query=86&hit=5&index=gi%7C392765192%7Cgb%7CEJA21981.1%7C&db_idx=1&px=1&section=5&ave_thresh=33&_ignoreionsscorebelow=0&report=0&_sigthreshold=0.05&_msresflags=1089&_msresflags2=2&percolate=-1&percolate_rt=0&_minpeplen=5&sessionID=all_secdisabledsession) [89](http://10.200.41.11/mascot/cgi/peptide_view.pl?file=..%2Fdata%2F20160418%2FF080005.dat&query=89&hit=2&index=gi%7C392765192%7Cgb%7CEJA21981.1%7C&db_idx=1&px=1&section=5&ave_thresh=33&_ignoreionsscorebelow=0&report=0&_sigthreshold=0.05&_msresflags=1089&_msresflags2=2&percolate=-1&percolate_rt=0&_minpeplen=5&sessionID=all_secdisabledsession) [90](http://10.200.41.11/mascot/cgi/peptide_view.pl?file=..%2Fdata%2F20160418%2FF080005.dat&query=90&hit=4&index=gi%7C392765192%7Cgb%7CEJA21981.1%7C&db_idx=1&px=1&section=5&ave_thresh=33&_ignoreionsscorebelow=0&report=0&_sigthreshold=0.05&_msresflags=1089&_msresflags2=2&percolate=-1&percolate_rt=0&_minpeplen=5&sessionID=all_secdisabledsession) [91](http://10.200.41.11/mascot/cgi/peptide_view.pl?file=..%2Fdata%2F20160418%2FF080005.dat&query=91&hit=5&index=gi%7C392765192%7Cgb%7CEJA21981.1%7C&db_idx=1&px=1&section=5&ave_thresh=33&_ignoreionsscorebelow=0&report=0&_sigthreshold=0.05&_msresflags=1089&_msresflags2=2&percolate=-1&percolate_rt=0&_minpeplen=5&sessionID=all_secdisabledsession) [92](http://10.200.41.11/mascot/cgi/peptide_view.pl?file=..%2Fdata%2F20160418%2FF080005.dat&query=92&hit=1&index=gi%7C392765192%7Cgb%7CEJA21981.1%7C&db_idx=1&px=1&section=5&ave_thresh=33&_ignoreionsscorebelow=0&report=0&_sigthreshold=0.05&_msresflags=1089&_msresflags2=2&percolate=-1&percolate_rt=0&_minpeplen=5&sessionID=all_secdisabledsession) [93](http://10.200.41.11/mascot/cgi/peptide_view.pl?file=..%2Fdata%2F20160418%2FF080005.dat&query=93&hit=1&index=gi%7C392765192%7Cgb%7CEJA21981.1%7C&db_idx=1&px=1&section=5&ave_thresh=33&_ignoreionsscorebelow=0&report=0&_sigthreshold=0.05&_msresflags=1089&_msresflags2=2&percolate=-1&percolate_rt=0&_minpeplen=5&sessionID=all_secdisabledsession) [94](http://10.200.41.11/mascot/cgi/peptide_view.pl?file=..%2Fdata%2F20160418%2FF080005.dat&query=94&hit=5&index=gi%7C392765192%7Cgb%7CEJA21981.1%7C&db_idx=1&px=1&section=5&ave_thresh=33&_ignoreionsscorebelow=0&report=0&_sigthreshold=0.05&_msresflags=1089&_msresflags2=2&percolate=-1&percolate_rt=0&_minpeplen=5&sessionID=all_secdisabledsession) [95](http://10.200.41.11/mascot/cgi/peptide_view.pl?file=..%2Fdata%2F20160418%2FF080005.dat&query=95&hit=2&index=gi%7C392765192%7Cgb%7CEJA21981.1%7C&db_idx=1&px=1&section=5&ave_thresh=33&_ignoreionsscorebelow=0&report=0&_sigthreshold=0.05&_msresflags=1089&_msresflags2=2&percolate=-1&percolate_rt=0&_minpeplen=5&sessionID=all_secdisabledsession) |

### Protein sequence coverage: 4%

Matched peptides shown in ***bold red***.

| **1** | MPALINPGNH | QFSVINFLPH | QEYAGYSLK**V** | **AAKSAAGR**RN | PCNLTATQHA |
| --- | --- | --- | --- | --- | --- |
| **51** | PGVFFCVVAL | THLRFMVWWL | TACFYFLRHI | IRIMVVQAGQ | LSGWPVSVRA |
| **101** | GIPTPVWATT | HERRNSGGSV | TRYLTEVAIM | ATVPVLSHPE | FTFVFLAVRR |
| **151** | TDRDARPRPV | RVIADCEHAA | RLKLATEFIL | SFAARIPVKN | AGEVVA |

**********************************************************************************************************************

**2^nd^ technical replicate**

**Enzyme : Trypsin**

**Variable modifications :** [**Oxidation (M)**](http://10.200.41.11/mascot/cgi/client.pl?modification&mod_name=Oxidation%20%28M%29&file=..%2Fdata%2F20160418%2FF080006.dat)

**Mass values : Monoisotopic**

**Protein Mass : Unrestricted**

**Peptide Mass Tolerance : ± 20 ppm**

**Fragment Mass Tolerance: ± 0.4 Da**

**Max Missed Cleavages : 3**

**Instrument type : Default**

**Number of queries : 2949**

| **Protein hits           :** | [**gi|392616945|gb|EIW99373.1|**](http://10.200.41.11/mascot/cgi/master_results.pl?file=..%2Fdata%2F20160418%2FF080006.dat#Hit1) | pathogenicity island 1 effector protein SipC [Salmonella enterica subsp. enterica serovar Newport str. Levine 15] |
| --- | --- | --- |
|  | [**gi|194403640|gb|ACF63862.1|**](http://10.200.41.11/mascot/cgi/master_results.pl?file=..%2Fdata%2F20160418%2FF080006.dat#Hit2) | cell invasion protein SipB [Salmonella enterica subsp. enterica serovar Newport str. SL254] |
|  | [**gi|50830890|gb|AAT81610.1|**](http://10.200.41.11/mascot/cgi/master_results.pl?file=..%2Fdata%2F20160418%2FF080006.dat#Hit3) | phase 1 flagellin [Salmonella enterica subsp. enterica serovar Newport] |
|  | [**gi|194402702|gb|ACF62924.1|**](http://10.200.41.11/mascot/cgi/master_results.pl?file=..%2Fdata%2F20160418%2FF080006.dat#Hit4) | cell invasion protein SipA [Salmonella enterica subsp. enterica serovar Newport str. SL254] |
|  | [**gi|194401878|gb|ACF62100.1|**](http://10.200.41.11/mascot/cgi/master_results.pl?file=..%2Fdata%2F20160418%2FF080006.dat#Hit5) | ribosomal protein L11 [Salmonella enterica subsp. enterica serovar Newport str. SL254] |
|  | [**gi|194401698|gb|ACF61920.1|**](http://10.200.41.11/mascot/cgi/master_results.pl?file=..%2Fdata%2F20160418%2FF080006.dat#Hit6) | translation elongation factor Tu [Salmonella enterica subsp. enterica serovar Newport str. SL254] |
|  | [**gi|194401103|gb|ACF61325.1|**](http://10.200.41.11/mascot/cgi/master_results.pl?file=..%2Fdata%2F20160418%2FF080006.dat#Hit7) | ribosomal protein L6 [Salmonella enterica subsp. enterica serovar Newport str. SL254] |
|  | [**gi|194403829|gb|ACF64051.1|**](http://10.200.41.11/mascot/cgi/master_results.pl?file=..%2Fdata%2F20160418%2FF080006.dat#Hit8) | ribosomal protein L3 [Salmonella enterica subsp. enterica serovar Newport str. SL254] |
|  | [**gi|553467807|gb|ESB92309.1|**](http://10.200.41.11/mascot/cgi/master_results.pl?file=..%2Fdata%2F20160418%2FF080006.dat#Hit9) | trifunctional transcriptional regulator/proline dehydrogenase/pyrroline-5-carboxylate dehydrogenase, partial [Salmonella enterica subsp. enterica serovar Newport str. 637564_17] |

| **1.** | [gi|392616945|gb|EIW99373.1|](http://10.200.41.11/mascot/cgi/protein_view.pl?file=..%2Fdata%2F20160418%2FF080006.dat&hit=gi%7C392616945%7Cgb%7CEIW99373.1%7C&db_idx=1&px=1&ave_thresh=32&_ignoreionsscorebelow=0&report=0&_sigthreshold=0.05&_msresflags=1089&_msresflags2=2&percolate=-1&percolate_rt=0&_minpeplen=5&sessionID=all_secdisabledsession)    **Mass:** 42957    **Score:** 196    **Matches:** 9(5)  **Sequences:** 4(2)  **emPAI:** 0.31 |
| --- | --- |
|  | pathogenicity island 1 effector protein SipC [Salmonella enterica subsp. enterica serovar Newport str. Levine 15] |

|  | **Query** | **Observed** | **Mr(expt)** | **Mr(calc)** | **ppm** | **Miss** | **Score** | **Expect** | **Rank** | **Unique** | **Peptide** |
| --- | --- | --- | --- | --- | --- | --- | --- | --- | --- | --- | --- |
|  | [1128](http://10.200.41.11/mascot/cgi/peptide_view.pl?file=..%2Fdata%2F20160418%2FF080006.dat&query=1128&hit=1&index=gi%7C392616945%7Cgb%7CEIW99373.1%7C&db_idx=1&px=1&section=5&ave_thresh=32&_ignoreionsscorebelow=0&report=0&_sigthreshold=0.05&_msresflags=1089&_msresflags2=2&percolate=-1&percolate_rt=0&_minpeplen=5&sessionID=all_secdisabledsession) | **494.7728** | **987.5310** | **987.5237** | **7.41** | **0** | **37** | **0.048** | **1** | **U** | **K.LGAEGVDSLK.S** [1127](http://10.200.41.11/mascot/cgi/peptide_view.pl?file=..%2Fdata%2F20160418%2FF080006.dat&query=1127&hit=1&index=gi%7C392616945%7Cgb%7CEIW99373.1%7C&db_idx=1&px=1&section=5&ave_thresh=32&_ignoreionsscorebelow=0&report=0&_sigthreshold=0.05&_msresflags=1089&_msresflags2=2&percolate=-1&percolate_rt=0&_minpeplen=5&sessionID=all_secdisabledsession) |
|  | [2088](http://10.200.41.11/mascot/cgi/peptide_view.pl?file=..%2Fdata%2F20160418%2FF080006.dat&query=2088&hit=1&index=gi%7C392616945%7Cgb%7CEIW99373.1%7C&db_idx=1&px=1&section=5&ave_thresh=32&_ignoreionsscorebelow=0&report=0&_sigthreshold=0.05&_msresflags=1089&_msresflags2=2&percolate=-1&percolate_rt=0&_minpeplen=5&sessionID=all_secdisabledsession) | **617.3500** | **1232.6854** | **1232.6612** | **19.6** | **0** | **0** | **1.5e+002** | **1** | **U** | **K.DILNSIGISSSK.V** |
|  | [2331](http://10.200.41.11/mascot/cgi/peptide_view.pl?file=..%2Fdata%2F20160418%2FF080006.dat&query=2331&hit=1&index=gi%7C392616945%7Cgb%7CEIW99373.1%7C&db_idx=1&px=1&section=5&ave_thresh=32&_ignoreionsscorebelow=0&report=0&_sigthreshold=0.05&_msresflags=1089&_msresflags2=2&percolate=-1&percolate_rt=0&_minpeplen=5&sessionID=all_secdisabledsession) | **651.8284** | **1301.6423** | **1301.6324** | **7.64** | **0** | **66** | **3.3e-005** | **1** | **U** | **R.SEQQISQVNNR.V** [2328](http://10.200.41.11/mascot/cgi/peptide_view.pl?file=..%2Fdata%2F20160418%2FF080006.dat&query=2328&hit=1&index=gi%7C392616945%7Cgb%7CEIW99373.1%7C&db_idx=1&px=1&section=5&ave_thresh=32&_ignoreionsscorebelow=0&report=0&_sigthreshold=0.05&_msresflags=1089&_msresflags2=2&percolate=-1&percolate_rt=0&_minpeplen=5&sessionID=all_secdisabledsession) [2329](http://10.200.41.11/mascot/cgi/peptide_view.pl?file=..%2Fdata%2F20160418%2FF080006.dat&query=2329&hit=1&index=gi%7C392616945%7Cgb%7CEIW99373.1%7C&db_idx=1&px=1&section=5&ave_thresh=32&_ignoreionsscorebelow=0&report=0&_sigthreshold=0.05&_msresflags=1089&_msresflags2=2&percolate=-1&percolate_rt=0&_minpeplen=5&sessionID=all_secdisabledsession) [2330](http://10.200.41.11/mascot/cgi/peptide_view.pl?file=..%2Fdata%2F20160418%2FF080006.dat&query=2330&hit=1&index=gi%7C392616945%7Cgb%7CEIW99373.1%7C&db_idx=1&px=1&section=5&ave_thresh=32&_ignoreionsscorebelow=0&report=0&_sigthreshold=0.05&_msresflags=1089&_msresflags2=2&percolate=-1&percolate_rt=0&_minpeplen=5&sessionID=all_secdisabledsession) |
|  | [2709](http://10.200.41.11/mascot/cgi/peptide_view.pl?file=..%2Fdata%2F20160418%2FF080006.dat&query=2709&hit=1&index=gi%7C392616945%7Cgb%7CEIW99373.1%7C&db_idx=1&px=1&section=5&ave_thresh=32&_ignoreionsscorebelow=0&report=0&_sigthreshold=0.05&_msresflags=1089&_msresflags2=2&percolate=-1&percolate_rt=0&_minpeplen=5&sessionID=all_secdisabledsession) | **489.2382** | **1464.6928** | **1464.6804** | **8.46** | **1** | **10** | **17** | **1** | **U** | **R.VASTASDEARESSR.K** [2708](http://10.200.41.11/mascot/cgi/peptide_view.pl?file=..%2Fdata%2F20160418%2FF080006.dat&query=2708&hit=1&index=gi%7C392616945%7Cgb%7CEIW99373.1%7C&db_idx=1&px=1&section=5&ave_thresh=32&_ignoreionsscorebelow=0&report=0&_sigthreshold=0.05&_msresflags=1089&_msresflags2=2&percolate=-1&percolate_rt=0&_minpeplen=5&sessionID=all_secdisabledsession) |

### Protein sequence coverage: 11%

Matched peptides shown in ***bold red***.

| **1** | MLISNVGINP | AAYLNNHSVE | NSSQTASQSV | SAK**DILNSIG** | **ISSSK**VSDLG |
| --- | --- | --- | --- | --- | --- |
| **51** | LSPTLSAPAP | GVLTQTPGTI | TSFLKASIQN | TDMNQDLNAL | ANNVTTKANE |
| **101** | VVQTQLREQQ | AEVGKFFDIS | GMSSSAVALL | AAANTLMLTL | NQADSKLSGK |
| **151** | LSLVSFDAAK | TTASSMMREG | MNALSGSISQ | SALQLGITGV | GAKLEYKGLQ |
| **201** | NERGALKHNA | AKIDKLTTES | HSIKNVLNGQ | NSVK**LGAEGV** | **DSLK**SLNMKK |
| **251** | TGTDATKNLN | DATLKSNAGT | SATESLGIKD | SNKQISPEHQ | AILSKRLESV |
| **301** | ESDIRLEQNT | MDMTRIDARK | MQMTGDLIMK | NSVTVGGIAG | ASGQYAATQE |
| **351** | R**SEQQISQVN** | **NRVASTASDE** | **ARESSR**KSTS | LIQEMLKTME | SINQSKASAL |
| **401** | AAIAGNIRA |  |  |  |  |

| **2.** | [gi|194403640|gb|ACF63862.1|](http://10.200.41.11/mascot/cgi/protein_view.pl?file=..%2Fdata%2F20160418%2FF080006.dat&hit=gi%7C194403640%7Cgb%7CACF63862.1%7C&db_idx=1&px=1&ave_thresh=32&_ignoreionsscorebelow=0&report=0&_sigthreshold=0.05&_msresflags=1089&_msresflags2=2&percolate=-1&percolate_rt=0&_minpeplen=5&sessionID=all_secdisabledsession)    **Mass:** 62382    **Score:** 103    **Matches:** 7(4)  **Sequences:** 5(3)  **emPAI:** 0.32 |
| --- | --- |
|  | cell invasion protein SipB [Salmonella enterica subsp. enterica serovar Newport str. SL254] |

|  | **Query** | **Observed** | **Mr(expt)** | **Mr(calc)** | **ppm** | **Miss** | **Score** | **Expect** | **Rank** | **Unique** | **Peptide** |
| --- | --- | --- | --- | --- | --- | --- | --- | --- | --- | --- | --- |
|  | [799](http://10.200.41.11/mascot/cgi/peptide_view.pl?file=..%2Fdata%2F20160418%2FF080006.dat&query=799&hit=1&index=gi%7C194403640%7Cgb%7CACF63862.1%7C&db_idx=1&px=1&section=5&ave_thresh=32&_ignoreionsscorebelow=0&report=0&_sigthreshold=0.05&_msresflags=1089&_msresflags2=2&percolate=-1&percolate_rt=0&_minpeplen=5&sessionID=all_secdisabledsession) | **461.7263** | **921.4380** | **921.4304** | **8.24** | **0** | **40** | **0.011** | **1** | **U** | **R.SGYTQNPR.L** [798](http://10.200.41.11/mascot/cgi/peptide_view.pl?file=..%2Fdata%2F20160418%2FF080006.dat&query=798&hit=1&index=gi%7C194403640%7Cgb%7CACF63862.1%7C&db_idx=1&px=1&section=5&ave_thresh=32&_ignoreionsscorebelow=0&report=0&_sigthreshold=0.05&_msresflags=1089&_msresflags2=2&percolate=-1&percolate_rt=0&_minpeplen=5&sessionID=all_secdisabledsession) |
|  | [1071](http://10.200.41.11/mascot/cgi/peptide_view.pl?file=..%2Fdata%2F20160418%2FF080006.dat&query=1071&hit=6&index=gi%7C194403640%7Cgb%7CACF63862.1%7C&db_idx=1&px=1&section=5&ave_thresh=32&_ignoreionsscorebelow=0&report=0&_sigthreshold=0.05&_msresflags=1089&_msresflags2=2&percolate=-1&percolate_rt=0&_minpeplen=5&sessionID=all_secdisabledsession) | **490.7472** | **979.4799** | **979.4909** | **-11.27** | **0** | **2** | **1.2e+002** | **6** | **U** | **K.LFTQGMQR.I** |
|  | [1118](http://10.200.41.11/mascot/cgi/peptide_view.pl?file=..%2Fdata%2F20160418%2FF080006.dat&query=1118&hit=4&index=gi%7C194403640%7Cgb%7CACF63862.1%7C&db_idx=1&px=1&section=5&ave_thresh=32&_ignoreionsscorebelow=0&report=0&_sigthreshold=0.05&_msresflags=1089&_msresflags2=2&percolate=-1&percolate_rt=0&_minpeplen=5&sessionID=all_secdisabledsession) | **494.2830** | **986.5514** | **986.5396** | **11.9** | **0** | **1** | **1.3e+002** | **4** | **U** | **K.ELVGNTLNK.V** [1123](http://10.200.41.11/mascot/cgi/peptide_view.pl?file=..%2Fdata%2F20160418%2FF080006.dat&query=1123&hit=8&index=gi%7C194403640%7Cgb%7CACF63862.1%7C&db_idx=1&px=1&section=5&ave_thresh=32&_ignoreionsscorebelow=0&report=0&_sigthreshold=0.05&_msresflags=1089&_msresflags2=2&percolate=-1&percolate_rt=0&_minpeplen=5&sessionID=all_secdisabledsession) |
|  | [1519](http://10.200.41.11/mascot/cgi/peptide_view.pl?file=..%2Fdata%2F20160418%2FF080006.dat&query=1519&hit=1&index=gi%7C194403640%7Cgb%7CACF63862.1%7C&db_idx=1&px=1&section=5&ave_thresh=32&_ignoreionsscorebelow=0&report=0&_sigthreshold=0.05&_msresflags=1089&_msresflags2=2&percolate=-1&percolate_rt=0&_minpeplen=5&sessionID=all_secdisabledsession) | **531.7866** | **1061.5586** | **1061.5505** | **7.57** | **0** | **41** | **0.014** | **1** | **U** | **R.LAEAAFEGVR.K** |
|  | [1562](http://10.200.41.11/mascot/cgi/peptide_view.pl?file=..%2Fdata%2F20160418%2FF080006.dat&query=1562&hit=1&index=gi%7C194403640%7Cgb%7CACF63862.1%7C&db_idx=1&px=1&section=5&ave_thresh=32&_ignoreionsscorebelow=0&report=0&_sigthreshold=0.05&_msresflags=1089&_msresflags2=2&percolate=-1&percolate_rt=0&_minpeplen=5&sessionID=all_secdisabledsession) | **548.2626** | **1094.5106** | **1094.5026** | **7.30** | **0** | **38** | **0.012** | **1** | **U** | **-.MVNDASSISR.S** |

### Protein sequence coverage: 7%

Matched peptides shown in ***bold red***.

| **1** | **MVNDASSISR** | **SGYTQNPRLA** | **EAAFEGVR**KN | TDFLKAADKA | FKDVVATKAG |
| --- | --- | --- | --- | --- | --- |
| **51** | DLKAGTKSGE | SAINTVGLKP | PTDAAREKLS | SEGQLTLLLG | KLMTLLGDVS |
| **101** | LSQLESRLAV | WQAMIESQKE | MGIQVSKEFQ | TALGEAQEAT | DLYEASIKKT |
| **151** | DTAKSVYDAA | AKKLTQAQNK | LQSLDPADPG | YAQAEAAVEQ | AGKEATEAKE |
| **201** | ALDKATDATV | KAGTDAKAKA | EKADNILTKF | QGTANAASQN | QVSQGEQDNL |
| **251** | SNVARLTMLM | AMFIEIVGKN | TEESLQNDLA | LFNALQEGRQ | AEMEKKSAEF |
| **301** | QEETRKAEET | NRIMGCIGKV | LGALLTIVSV | VAAVFTGGAS | LALAAVGLAV |
| **351** | MVADEIVKAA | TGVSFIQQAL | NPIMEHVLKP | LMELIGKAIT | KALEGLGVDK |
| **401** | KTAEMAGSIV | GAIVAAIAMV | AVIVVVAVVG | KGAAAKLGNA | LSKMMGETIK |
| **451** | KLVPNVLKQL | AQNGSK**LFTQ** | **GMQR**ITSGLG | NVGSKMGLQT | NALSK**ELVGN** |
| **501** | **TLNK**VALGME | VTNTAAQSAG | GVAEGVFIKN | ASEALADFML | ARFAMDQIQQ |
| **551** | WLKQSVEIFG | ENQKVTAELQ | KAMSSAVQQN | ADASRFILRQ | SRA |

| **3.** | [gi|50830890|gb|AAT81610.1|](http://10.200.41.11/mascot/cgi/protein_view.pl?file=..%2Fdata%2F20160418%2FF080006.dat&hit=gi%7C50830890%7Cgb%7CAAT81610.1%7C&db_idx=1&px=1&ave_thresh=32&_ignoreionsscorebelow=0&report=0&_sigthreshold=0.05&_msresflags=1089&_msresflags2=2&percolate=-1&percolate_rt=0&_minpeplen=5&sessionID=all_secdisabledsession)    **Mass:** 52223    **Score:** 78     **Matches:** 9(3)  **Sequences:** 2(1)  **emPAI:** 0.12 |
| --- | --- |
|  | phase 1 flagellin [Salmonella enterica subsp. enterica serovar Newport] |

|  | **Query** | **Observed** | **Mr(expt)** | **Mr(calc)** | **ppm** | **Miss** | **Score** | **Expect** | **Rank** | **Unique** | **Peptide** |
| --- | --- | --- | --- | --- | --- | --- | --- | --- | --- | --- | --- |
|  | [1682](http://10.200.41.11/mascot/cgi/peptide_view.pl?file=..%2Fdata%2F20160418%2FF080006.dat&query=1682&hit=1&index=gi%7C50830890%7Cgb%7CAAT81610.1%7C&db_idx=1&px=1&section=5&ave_thresh=32&_ignoreionsscorebelow=0&report=0&_sigthreshold=0.05&_msresflags=1089&_msresflags2=2&percolate=-1&percolate_rt=0&_minpeplen=5&sessionID=all_secdisabledsession) | **566.8057** | **1131.5969** | **1131.5884** | **7.53** | **0** | **39** | **0.028** | **1** | **U** | **K.SQSALGTAIER.L** [1681](http://10.200.41.11/mascot/cgi/peptide_view.pl?file=..%2Fdata%2F20160418%2FF080006.dat&query=1681&hit=1&index=gi%7C50830890%7Cgb%7CAAT81610.1%7C&db_idx=1&px=1&section=5&ave_thresh=32&_ignoreionsscorebelow=0&report=0&_sigthreshold=0.05&_msresflags=1089&_msresflags2=2&percolate=-1&percolate_rt=0&_minpeplen=5&sessionID=all_secdisabledsession) [1683](http://10.200.41.11/mascot/cgi/peptide_view.pl?file=..%2Fdata%2F20160418%2FF080006.dat&query=1683&hit=1&index=gi%7C50830890%7Cgb%7CAAT81610.1%7C&db_idx=1&px=1&section=5&ave_thresh=32&_ignoreionsscorebelow=0&report=0&_sigthreshold=0.05&_msresflags=1089&_msresflags2=2&percolate=-1&percolate_rt=0&_minpeplen=5&sessionID=all_secdisabledsession) |
|  | [2870](http://10.200.41.11/mascot/cgi/peptide_view.pl?file=..%2Fdata%2F20160418%2FF080006.dat&query=2870&hit=1&index=gi%7C50830890%7Cgb%7CAAT81610.1%7C&db_idx=1&px=1&section=5&ave_thresh=32&_ignoreionsscorebelow=0&report=0&_sigthreshold=0.05&_msresflags=1089&_msresflags2=2&percolate=-1&percolate_rt=0&_minpeplen=5&sessionID=all_secdisabledsession) | **539.9373** | **1616.7901** | **1616.8006** | **-6.45** | **1** | **6** | **38** | **1** | **U** | **K.KALEDGGVSNADATAAK.L** [2868](http://10.200.41.11/mascot/cgi/peptide_view.pl?file=..%2Fdata%2F20160418%2FF080006.dat&query=2868&hit=1&index=gi%7C50830890%7Cgb%7CAAT81610.1%7C&db_idx=1&px=1&section=5&ave_thresh=32&_ignoreionsscorebelow=0&report=0&_sigthreshold=0.05&_msresflags=1089&_msresflags2=2&percolate=-1&percolate_rt=0&_minpeplen=5&sessionID=all_secdisabledsession) [2869](http://10.200.41.11/mascot/cgi/peptide_view.pl?file=..%2Fdata%2F20160418%2FF080006.dat&query=2869&hit=1&index=gi%7C50830890%7Cgb%7CAAT81610.1%7C&db_idx=1&px=1&section=5&ave_thresh=32&_ignoreionsscorebelow=0&report=0&_sigthreshold=0.05&_msresflags=1089&_msresflags2=2&percolate=-1&percolate_rt=0&_minpeplen=5&sessionID=all_secdisabledsession) [2871](http://10.200.41.11/mascot/cgi/peptide_view.pl?file=..%2Fdata%2F20160418%2FF080006.dat&query=2871&hit=1&index=gi%7C50830890%7Cgb%7CAAT81610.1%7C&db_idx=1&px=1&section=5&ave_thresh=32&_ignoreionsscorebelow=0&report=0&_sigthreshold=0.05&_msresflags=1089&_msresflags2=2&percolate=-1&percolate_rt=0&_minpeplen=5&sessionID=all_secdisabledsession) [2872](http://10.200.41.11/mascot/cgi/peptide_view.pl?file=..%2Fdata%2F20160418%2FF080006.dat&query=2872&hit=2&index=gi%7C50830890%7Cgb%7CAAT81610.1%7C&db_idx=1&px=1&section=5&ave_thresh=32&_ignoreionsscorebelow=0&report=0&_sigthreshold=0.05&_msresflags=1089&_msresflags2=2&percolate=-1&percolate_rt=0&_minpeplen=5&sessionID=all_secdisabledsession) [2873](http://10.200.41.11/mascot/cgi/peptide_view.pl?file=..%2Fdata%2F20160418%2FF080006.dat&query=2873&hit=1&index=gi%7C50830890%7Cgb%7CAAT81610.1%7C&db_idx=1&px=1&section=5&ave_thresh=32&_ignoreionsscorebelow=0&report=0&_sigthreshold=0.05&_msresflags=1089&_msresflags2=2&percolate=-1&percolate_rt=0&_minpeplen=5&sessionID=all_secdisabledsession) |

### Protein sequence coverage: 5%

Matched peptides shown in ***bold red***.

| **1** | MAQVINTNSL | SLLTQNNLNK | **SQSALGTAIE** | **R**LSSGLRINS | AKDDAAGQAI |
| --- | --- | --- | --- | --- | --- |
| **51** | ANRFTANIKG | LTQASRNAND | GISIAQTTEG | ALNEINNNLQ | RVRELAVQSA |
| **101** | NSTNSQSDLD | SIQAEITQRL | NEIDRVSGQT | QFNGVKVLAQ | DNTLTIQVGA |
| **151** | NDGETIDIDL | KQINSQTLGL | DTLNVQKAYD | VSATAAMDPK | SFTDGTKNLT |
| **201** | APDATAIKAA | LGNPAATGDS | LSATLSFKDG | KYYATVAGYT | NAADTSKNGK |
| **251** | YEVNVDSATG | AVTFNAAPTK | ATVTGDTTVT | KVQVNAPVAV | STDVK**KALED** |
| **301** | **GGVSNADATA** | **AK**LVKMSYTD | KNGKSIDGGY | ALEAGGKYYA | ATYDEGTGKI |
| **351** | TANVTTYTDS | TGVTKTAANQ | LGGVDGKTEV | VTIDGKTYNA | SKAAGHDFKA |
| **401** | QPELAEAAAK | TTENPLAKID | AALAQVDALR | SDLGAVQNRF | NSAITNLGNT |
| **451** | VNNLSEARSR | IEDSDYATEV | SNMSRAQILQ | QAGTSVLAQA | NQVPQNVLSL |
| **501** | LR |  |  |  |  |

| **4.** | [gi|194402702|gb|ACF62924.1|](http://10.200.41.11/mascot/cgi/protein_view.pl?file=..%2Fdata%2F20160418%2FF080006.dat&hit=gi%7C194402702%7Cgb%7CACF62924.1%7C&db_idx=1&px=1&ave_thresh=32&_ignoreionsscorebelow=0&report=0&_sigthreshold=0.05&_msresflags=1089&_msresflags2=2&percolate=-1&percolate_rt=0&_minpeplen=5&sessionID=all_secdisabledsession)    **Mass:** 72333    **Score:** 67     **Matches:** 7(2)  **Sequences:** 2(1)  **emPAI:** 0.08 |
| --- | --- |
|  | cell invasion protein SipA [Salmonella enterica subsp. enterica serovar Newport str. SL254] |

|  | **Query** | **Observed** | **Mr(expt)** | **Mr(calc)** | **ppm** | **Miss** | **Score** | **Expect** | **Rank** | **Unique** | **Peptide** |
| --- | --- | --- | --- | --- | --- | --- | --- | --- | --- | --- | --- |
|  | [1071](http://10.200.41.11/mascot/cgi/peptide_view.pl?file=..%2Fdata%2F20160418%2FF080006.dat&query=1071&hit=1&index=gi%7C194402702%7Cgb%7CACF62924.1%7C&db_idx=1&px=1&section=5&ave_thresh=32&_ignoreionsscorebelow=0&report=0&_sigthreshold=0.05&_msresflags=1089&_msresflags2=2&percolate=-1&percolate_rt=0&_minpeplen=5&sessionID=all_secdisabledsession) | 490.7472 | 979.4799 | 979.4723 | 7.75 | 0 | 27 | 0.4 | 1 | U | R.TFIDNSQR.N [1068](http://10.200.41.11/mascot/cgi/peptide_view.pl?file=..%2Fdata%2F20160418%2FF080006.dat&query=1068&hit=1&index=gi%7C194402702%7Cgb%7CACF62924.1%7C&db_idx=1&px=1&section=5&ave_thresh=32&_ignoreionsscorebelow=0&report=0&_sigthreshold=0.05&_msresflags=1089&_msresflags2=2&percolate=-1&percolate_rt=0&_minpeplen=5&sessionID=all_secdisabledsession) [1069](http://10.200.41.11/mascot/cgi/peptide_view.pl?file=..%2Fdata%2F20160418%2FF080006.dat&query=1069&hit=1&index=gi%7C194402702%7Cgb%7CACF62924.1%7C&db_idx=1&px=1&section=5&ave_thresh=32&_ignoreionsscorebelow=0&report=0&_sigthreshold=0.05&_msresflags=1089&_msresflags2=2&percolate=-1&percolate_rt=0&_minpeplen=5&sessionID=all_secdisabledsession) [1070](http://10.200.41.11/mascot/cgi/peptide_view.pl?file=..%2Fdata%2F20160418%2FF080006.dat&query=1070&hit=1&index=gi%7C194402702%7Cgb%7CACF62924.1%7C&db_idx=1&px=1&section=5&ave_thresh=32&_ignoreionsscorebelow=0&report=0&_sigthreshold=0.05&_msresflags=1089&_msresflags2=2&percolate=-1&percolate_rt=0&_minpeplen=5&sessionID=all_secdisabledsession) [1072](http://10.200.41.11/mascot/cgi/peptide_view.pl?file=..%2Fdata%2F20160418%2FF080006.dat&query=1072&hit=1&index=gi%7C194402702%7Cgb%7CACF62924.1%7C&db_idx=1&px=1&section=5&ave_thresh=32&_ignoreionsscorebelow=0&report=0&_sigthreshold=0.05&_msresflags=1089&_msresflags2=2&percolate=-1&percolate_rt=0&_minpeplen=5&sessionID=all_secdisabledsession) |
|  | [1915](http://10.200.41.11/mascot/cgi/peptide_view.pl?file=..%2Fdata%2F20160418%2FF080006.dat&query=1915&hit=1&index=gi%7C194402702%7Cgb%7CACF62924.1%7C&db_idx=1&px=1&section=5&ave_thresh=32&_ignoreionsscorebelow=0&report=0&_sigthreshold=0.05&_msresflags=1089&_msresflags2=2&percolate=-1&percolate_rt=0&_minpeplen=5&sessionID=all_secdisabledsession) | **588.3084** | **1174.6023** | **1174.5942** | **6.85** | **0** | **51** | **0.0016** | **1** | **U** | **K.LTQEQGTSVGR.E** [1914](http://10.200.41.11/mascot/cgi/peptide_view.pl?file=..%2Fdata%2F20160418%2FF080006.dat&query=1914&hit=1&index=gi%7C194402702%7Cgb%7CACF62924.1%7C&db_idx=1&px=1&section=5&ave_thresh=32&_ignoreionsscorebelow=0&report=0&_sigthreshold=0.05&_msresflags=1089&_msresflags2=2&percolate=-1&percolate_rt=0&_minpeplen=5&sessionID=all_secdisabledsession) |

### Protein sequence coverage: 2%

Matched peptides shown in ***bold red***.

| **1** | MQTEIKTQAT | NLAANLSAVR | ESATTTLSGE | IKGPQLEDFP | ALIKQASLDA |
| --- | --- | --- | --- | --- | --- |
| **51** | LFKCGKDAEA | LKEVFTNSNN | VAGKKAIMEF | AGLFRSALNA | TSDSPEAKTL |
| **101** | LMKVGAEYTA | QIIKDGLKEK | SAFGPWLPET | KKAEAKLENL | EKQLLDIIKN |
| **151** | NTGGELSKLS | TNLVMQEVMP | YIASCIEHNF | GCTLDPLTRS | NLTHLVDKAA |
| **201** | AKAVEALDMC | HQK**LTQEQGT** | **SVGR**EARHLE | MQTLIPLLLR | NVFAQIPADK |
| **251** | LPDPKIPEPA | AGPVPDGGKK | AEPTGININI | NIDSSNHSVD | NSKHINNSRS |
| **301** | HVDNSQRHID | NSNHDNSRKT | IDNSR**TFIDN** | **SQR**NGESHHS | TNSSNVSHSH |
| **351** | SRVDSTTHQT | ETAHSASTGA | IDHGIAGKID | VTAHATAEAV | TNASSESKDG |
| **401** | KVVTSEKGTT | GETTSFDEVD | GVTSKSIIGK | PVQATVHGVD | DNKQQSQTAE |
| **451** | IVNVKPLASQ | LAGVENVKTD | TLQSDTTVIT | GNKAGTTDND | NSQTDKTGPF |
| **501** | SGLKFKQNSF | LSTVPSVTNM | HSMHFDARET | FLGVIRKALE | PDTSTPFPVR |
| **551** | RAFDGLRAEI | LPNDTIKSAA | LKAQCSDIDK | HPELKAKMET | LKEVITHHPQ |
| **601** | KEKLAEIALQ | FAREAGLTRL | KGETDYVLSN | VLDGLIGDGS | WRAGPAYESY |
| **651** | LNKPGVDRVI | TTVDGLHMQR |  |  |  |

**********************************************************************************************************************

**3^rd^ technical replicate**

**Enzyme : Trypsin**

**Variable modifications :** [**Oxidation (M)**](http://10.200.41.11/mascot/cgi/client.pl?modification&mod_name=Oxidation%20%28M%29&file=..%2Fdata%2F20160418%2FF080008.dat)

**Mass values : Monoisotopic**

**Protein Mass : Unrestricted**

**Peptide Mass Tolerance : ± 20 ppm**

**Fragment Mass Tolerance: ± 0.4 Da**

**Max Missed Cleavages : 3**

**Instrument type : Default**

**Number of queries : 3055**

| **Protein hits           :** | [**gi|392616945|gb|EIW99373.1|**](http://10.200.41.11/mascot/cgi/master_results.pl?file=..%2Fdata%2F20160418%2FF080008.dat#Hit1) | pathogenicity island 1 effector protein SipC [Salmonella enterica subsp. enterica serovar Newport str. Levine 15] |
| --- | --- | --- |
|  | [**gi|194403331|gb|ACF63553.1|**](http://10.200.41.11/mascot/cgi/master_results.pl?file=..%2Fdata%2F20160418%2FF080008.dat#Hit2) | negative regulator of flagellin synthesis [Salmonella enterica subsp. enterica serovar Newport str. SL254] |
|  | [**gi|392616944|gb|EIW99372.1|**](http://10.200.41.11/mascot/cgi/master_results.pl?file=..%2Fdata%2F20160418%2FF080008.dat#Hit3) | cell invasion protein SipD [Salmonella enterica subsp. enterica serovar Newport str. Levine 15] |
|  | [**gi|194403640|gb|ACF63862.1|**](http://10.200.41.11/mascot/cgi/master_results.pl?file=..%2Fdata%2F20160418%2FF080008.dat#Hit4) | cell invasion protein SipB [Salmonella enterica subsp. enterica serovar Newport str. SL254] |
|  | [**gi|194402702|gb|ACF62924.1|**](http://10.200.41.11/mascot/cgi/master_results.pl?file=..%2Fdata%2F20160418%2FF080008.dat#Hit5) | cell invasion protein SipA [Salmonella enterica subsp. enterica serovar Newport str. SL254] |
|  | [**gi|194403829|gb|ACF64051.1|**](http://10.200.41.11/mascot/cgi/master_results.pl?file=..%2Fdata%2F20160418%2FF080008.dat#Hit6) | ribosomal protein L3 [Salmonella enterica subsp. enterica serovar Newport str. SL254] |
|  | [**gi|194404219|gb|ACF64441.1|**](http://10.200.41.11/mascot/cgi/master_results.pl?file=..%2Fdata%2F20160418%2FF080008.dat#Hit7) | flagellar hook-associated protein 3 [Salmonella enterica subsp. enterica serovar Newport str. SL254] |
|  | [**gi|194405039|gb|ACF65261.1|**](http://10.200.41.11/mascot/cgi/master_results.pl?file=..%2Fdata%2F20160418%2FF080008.dat#Hit8) | putative amidohydrolase family [Salmonella enterica subsp. enterica serovar Newport str. SL254] |

| **1.** | [gi|392616945|gb|EIW99373.1|](http://10.200.41.11/mascot/cgi/protein_view.pl?file=..%2Fdata%2F20160418%2FF080008.dat&hit=gi%7C392616945%7Cgb%7CEIW99373.1%7C&db_idx=1&px=1&ave_thresh=33&_ignoreionsscorebelow=0&report=0&_sigthreshold=0.05&_msresflags=1089&_msresflags2=2&percolate=-1&percolate_rt=0&_minpeplen=5&sessionID=all_secdisabledsession)    **Mass:** 42957    **Score:** 219    **Matches:** 11(8)  **Sequences:** 4(3)  **emPAI:** 0.47 |
| --- | --- |
|  | pathogenicity island 1 effector protein SipC [Salmonella enterica subsp. enterica serovar Newport str. Levine 15] |

|  | **Query** | **Observed** | **Mr(expt)** | **Mr(calc)** | **ppm** | **Miss** | **Score** | **Expect** | **Rank** | **Unique** | **Peptide** |
| --- | --- | --- | --- | --- | --- | --- | --- | --- | --- | --- | --- |
|  | [810](http://10.200.41.11/mascot/cgi/peptide_view.pl?file=..%2Fdata%2F20160418%2FF080008.dat&query=810&hit=1&index=gi%7C392616945%7Cgb%7CEIW99373.1%7C&db_idx=1&px=1&section=5&ave_thresh=33&_ignoreionsscorebelow=0&report=0&_sigthreshold=0.05&_msresflags=1089&_msresflags2=2&percolate=-1&percolate_rt=0&_minpeplen=5&sessionID=all_secdisabledsession) | **494.7730** | **987.5314** | **987.5237** | **7.84** | **0** | **39** | **0.033** | **1** | **U** | **K.LGAEGVDSLK.S** [809](http://10.200.41.11/mascot/cgi/peptide_view.pl?file=..%2Fdata%2F20160418%2FF080008.dat&query=809&hit=1&index=gi%7C392616945%7Cgb%7CEIW99373.1%7C&db_idx=1&px=1&section=5&ave_thresh=33&_ignoreionsscorebelow=0&report=0&_sigthreshold=0.05&_msresflags=1089&_msresflags2=2&percolate=-1&percolate_rt=0&_minpeplen=5&sessionID=all_secdisabledsession) |
|  | [1088](http://10.200.41.11/mascot/cgi/peptide_view.pl?file=..%2Fdata%2F20160418%2FF080008.dat&query=1088&hit=1&index=gi%7C392616945%7Cgb%7CEIW99373.1%7C&db_idx=1&px=1&section=5&ave_thresh=33&_ignoreionsscorebelow=0&report=0&_sigthreshold=0.05&_msresflags=1089&_msresflags2=2&percolate=-1&percolate_rt=0&_minpeplen=5&sessionID=all_secdisabledsession) | **527.2509** | **1052.4873** | **1052.4808** | **6.15** | **0** | **36** | **0.019** | **1** | **U** | **K.TMESINQSK.A** [1089](http://10.200.41.11/mascot/cgi/peptide_view.pl?file=..%2Fdata%2F20160418%2FF080008.dat&query=1089&hit=1&index=gi%7C392616945%7Cgb%7CEIW99373.1%7C&db_idx=1&px=1&section=5&ave_thresh=33&_ignoreionsscorebelow=0&report=0&_sigthreshold=0.05&_msresflags=1089&_msresflags2=2&percolate=-1&percolate_rt=0&_minpeplen=5&sessionID=all_secdisabledsession) [1090](http://10.200.41.11/mascot/cgi/peptide_view.pl?file=..%2Fdata%2F20160418%2FF080008.dat&query=1090&hit=1&index=gi%7C392616945%7Cgb%7CEIW99373.1%7C&db_idx=1&px=1&section=5&ave_thresh=33&_ignoreionsscorebelow=0&report=0&_sigthreshold=0.05&_msresflags=1089&_msresflags2=2&percolate=-1&percolate_rt=0&_minpeplen=5&sessionID=all_secdisabledsession) |
|  | [1885](http://10.200.41.11/mascot/cgi/peptide_view.pl?file=..%2Fdata%2F20160418%2FF080008.dat&query=1885&hit=1&index=gi%7C392616945%7Cgb%7CEIW99373.1%7C&db_idx=1&px=1&section=5&ave_thresh=33&_ignoreionsscorebelow=0&report=0&_sigthreshold=0.05&_msresflags=1089&_msresflags2=2&percolate=-1&percolate_rt=0&_minpeplen=5&sessionID=all_secdisabledsession) | **651.8279** | **1301.6412** | **1301.6324** | **6.79** | **0** | **57** | **0.00029** | **1** | **U** | **R.SEQQISQVNNR.V** [1884](http://10.200.41.11/mascot/cgi/peptide_view.pl?file=..%2Fdata%2F20160418%2FF080008.dat&query=1884&hit=1&index=gi%7C392616945%7Cgb%7CEIW99373.1%7C&db_idx=1&px=1&section=5&ave_thresh=33&_ignoreionsscorebelow=0&report=0&_sigthreshold=0.05&_msresflags=1089&_msresflags2=2&percolate=-1&percolate_rt=0&_minpeplen=5&sessionID=all_secdisabledsession) [1886](http://10.200.41.11/mascot/cgi/peptide_view.pl?file=..%2Fdata%2F20160418%2FF080008.dat&query=1886&hit=1&index=gi%7C392616945%7Cgb%7CEIW99373.1%7C&db_idx=1&px=1&section=5&ave_thresh=33&_ignoreionsscorebelow=0&report=0&_sigthreshold=0.05&_msresflags=1089&_msresflags2=2&percolate=-1&percolate_rt=0&_minpeplen=5&sessionID=all_secdisabledsession) [1887](http://10.200.41.11/mascot/cgi/peptide_view.pl?file=..%2Fdata%2F20160418%2FF080008.dat&query=1887&hit=1&index=gi%7C392616945%7Cgb%7CEIW99373.1%7C&db_idx=1&px=1&section=5&ave_thresh=33&_ignoreionsscorebelow=0&report=0&_sigthreshold=0.05&_msresflags=1089&_msresflags2=2&percolate=-1&percolate_rt=0&_minpeplen=5&sessionID=all_secdisabledsession) |
|  | [2248](http://10.200.41.11/mascot/cgi/peptide_view.pl?file=..%2Fdata%2F20160418%2FF080008.dat&query=2248&hit=1&index=gi%7C392616945%7Cgb%7CEIW99373.1%7C&db_idx=1&px=1&section=5&ave_thresh=33&_ignoreionsscorebelow=0&report=0&_sigthreshold=0.05&_msresflags=1089&_msresflags2=2&percolate=-1&percolate_rt=0&_minpeplen=5&sessionID=all_secdisabledsession) | **489.2379** | **1464.6917** | **1464.6804** | **7.71** | **1** | **18** | **2** | **1** | **U** | **R.VASTASDEARESSR.K** [2249](http://10.200.41.11/mascot/cgi/peptide_view.pl?file=..%2Fdata%2F20160418%2FF080008.dat&query=2249&hit=1&index=gi%7C392616945%7Cgb%7CEIW99373.1%7C&db_idx=1&px=1&section=5&ave_thresh=33&_ignoreionsscorebelow=0&report=0&_sigthreshold=0.05&_msresflags=1089&_msresflags2=2&percolate=-1&percolate_rt=0&_minpeplen=5&sessionID=all_secdisabledsession) |

### Protein sequence coverage: 10%

Matched peptides shown in ***bold red***.

| **1** | MLISNVGINP | AAYLNNHSVE | NSSQTASQSV | SAKDILNSIG | ISSSKVSDLG |
| --- | --- | --- | --- | --- | --- |
| **51** | LSPTLSAPAP | GVLTQTPGTI | TSFLKASIQN | TDMNQDLNAL | ANNVTTKANE |
| **101** | VVQTQLREQQ | AEVGKFFDIS | GMSSSAVALL | AAANTLMLTL | NQADSKLSGK |
| **151** | LSLVSFDAAK | TTASSMMREG | MNALSGSISQ | SALQLGITGV | GAKLEYKGLQ |
| **201** | NERGALKHNA | AKIDKLTTES | HSIKNVLNGQ | NSVK**LGAEGV** | **DSLK**SLNMKK |
| **251** | TGTDATKNLN | DATLKSNAGT | SATESLGIKD | SNKQISPEHQ | AILSKRLESV |
| **301** | ESDIRLEQNT | MDMTRIDARK | MQMTGDLIMK | NSVTVGGIAG | ASGQYAATQE |
| **351** | R**SEQQISQVN** | **NRVASTASDE** | **ARESSR**KSTS | LIQEMLK**TME** | **SINQSK**ASAL |
| **401** | AAIAGNIRA |  |  |  |  |

| **2.** | [gi|194403331|gb|ACF63553.1|](http://10.200.41.11/mascot/cgi/protein_view.pl?file=..%2Fdata%2F20160418%2FF080008.dat&hit=gi%7C194403331%7Cgb%7CACF63553.1%7C&db_idx=1&px=1&ave_thresh=33&_ignoreionsscorebelow=0&report=0&_sigthreshold=0.05&_msresflags=1089&_msresflags2=2&percolate=-1&percolate_rt=0&_minpeplen=5&sessionID=all_secdisabledsession)    **Mass:** 10561    **Score:** 127    **Matches:** 8(1)  **Sequences:** 2(1)  **emPAI:** 0.65 |
| --- | --- |
|  | negative regulator of flagellin synthesis [Salmonella enterica subsp. enterica serovar Newport str. SL254] |

|  | **Query** | **Observed** | **Mr(expt)** | **Mr(calc)** | **ppm** | **Miss** | **Score** | **Expect** | **Rank** | **Unique** | **Peptide** |
| --- | --- | --- | --- | --- | --- | --- | --- | --- | --- | --- | --- |
|  | [869](http://10.200.41.11/mascot/cgi/peptide_view.pl?file=..%2Fdata%2F20160418%2FF080008.dat&query=869&hit=1&index=gi%7C194403331%7Cgb%7CACF63553.1%7C&db_idx=1&px=1&section=5&ave_thresh=33&_ignoreionsscorebelow=0&report=0&_sigthreshold=0.05&_msresflags=1089&_msresflags2=2&percolate=-1&percolate_rt=0&_minpeplen=5&sessionID=all_secdisabledsession) | **502.7521** | **1003.4897** | **1003.4822** | **7.49** | **0** | **32** | **0.076** | **1** | **U** | **R.ETSDTPVQK.T** [867](http://10.200.41.11/mascot/cgi/peptide_view.pl?file=..%2Fdata%2F20160418%2FF080008.dat&query=867&hit=1&index=gi%7C194403331%7Cgb%7CACF63553.1%7C&db_idx=1&px=1&section=5&ave_thresh=33&_ignoreionsscorebelow=0&report=0&_sigthreshold=0.05&_msresflags=1089&_msresflags2=2&percolate=-1&percolate_rt=0&_minpeplen=5&sessionID=all_secdisabledsession) [868](http://10.200.41.11/mascot/cgi/peptide_view.pl?file=..%2Fdata%2F20160418%2FF080008.dat&query=868&hit=1&index=gi%7C194403331%7Cgb%7CACF63553.1%7C&db_idx=1&px=1&section=5&ave_thresh=33&_ignoreionsscorebelow=0&report=0&_sigthreshold=0.05&_msresflags=1089&_msresflags2=2&percolate=-1&percolate_rt=0&_minpeplen=5&sessionID=all_secdisabledsession) [870](http://10.200.41.11/mascot/cgi/peptide_view.pl?file=..%2Fdata%2F20160418%2FF080008.dat&query=870&hit=1&index=gi%7C194403331%7Cgb%7CACF63553.1%7C&db_idx=1&px=1&section=5&ave_thresh=33&_ignoreionsscorebelow=0&report=0&_sigthreshold=0.05&_msresflags=1089&_msresflags2=2&percolate=-1&percolate_rt=0&_minpeplen=5&sessionID=all_secdisabledsession) [871](http://10.200.41.11/mascot/cgi/peptide_view.pl?file=..%2Fdata%2F20160418%2FF080008.dat&query=871&hit=1&index=gi%7C194403331%7Cgb%7CACF63553.1%7C&db_idx=1&px=1&section=5&ave_thresh=33&_ignoreionsscorebelow=0&report=0&_sigthreshold=0.05&_msresflags=1089&_msresflags2=2&percolate=-1&percolate_rt=0&_minpeplen=5&sessionID=all_secdisabledsession) [872](http://10.200.41.11/mascot/cgi/peptide_view.pl?file=..%2Fdata%2F20160418%2FF080008.dat&query=872&hit=1&index=gi%7C194403331%7Cgb%7CACF63553.1%7C&db_idx=1&px=1&section=5&ave_thresh=33&_ignoreionsscorebelow=0&report=0&_sigthreshold=0.05&_msresflags=1089&_msresflags2=2&percolate=-1&percolate_rt=0&_minpeplen=5&sessionID=all_secdisabledsession) [873](http://10.200.41.11/mascot/cgi/peptide_view.pl?file=..%2Fdata%2F20160418%2FF080008.dat&query=873&hit=1&index=gi%7C194403331%7Cgb%7CACF63553.1%7C&db_idx=1&px=1&section=5&ave_thresh=33&_ignoreionsscorebelow=0&report=0&_sigthreshold=0.05&_msresflags=1089&_msresflags2=2&percolate=-1&percolate_rt=0&_minpeplen=5&sessionID=all_secdisabledsession) |
|  | [2396](http://10.200.41.11/mascot/cgi/peptide_view.pl?file=..%2Fdata%2F20160418%2FF080008.dat&query=2396&hit=1&index=gi%7C194403331%7Cgb%7CACF63553.1%7C&db_idx=1&px=1&section=5&ave_thresh=33&_ignoreionsscorebelow=0&report=0&_sigthreshold=0.05&_msresflags=1089&_msresflags2=2&percolate=-1&percolate_rt=0&_minpeplen=5&sessionID=all_secdisabledsession) | **804.9129** | **1607.8113** | **1607.8002** | **6.86** | **0** | **61** | **0.00014** | **1** | **U** | **K.TSAATSASVTLSDAQAK.L** |

### Protein sequence coverage: 26%

Matched peptides shown in ***bold red***.

| **1** | MSIDRTSPLK | PVSTVQTR**ET** | **SDTPVQK**TRQ | EK**TSAATSAS** | **VTLSDAQAK**L |
| --- | --- | --- | --- | --- | --- |
| **51** | MQPGVSDINM | ERVEALKTAI | RNGELKMDTG | KIADSLIREA | QSYLQSK |

| **3.** | [gi|392616944|gb|EIW99372.1|](http://10.200.41.11/mascot/cgi/protein_view.pl?file=..%2Fdata%2F20160418%2FF080008.dat&hit=gi%7C392616944%7Cgb%7CEIW99372.1%7C&db_idx=1&px=1&ave_thresh=33&_ignoreionsscorebelow=0&report=0&_sigthreshold=0.05&_msresflags=1089&_msresflags2=2&percolate=-1&percolate_rt=0&_minpeplen=5&sessionID=all_secdisabledsession)    **Mass:** 37081    **Score:** 85     **Matches:** 2(2)  **Sequences:** 1(1)  **emPAI:** 0.16 |
| --- | --- |
|  | cell invasion protein SipD [Salmonella enterica subsp. enterica serovar Newport str. Levine 15] |

|  | **Query** | **Observed** | **Mr(expt)** | **Mr(calc)** | **ppm** | **Miss** | **Score** | **Expect** | **Rank** | **Unique** | **Peptide** |
| --- | --- | --- | --- | --- | --- | --- | --- | --- | --- | --- | --- |
|  | [2267](http://10.200.41.11/mascot/cgi/peptide_view.pl?file=..%2Fdata%2F20160418%2FF080008.dat&query=2267&hit=1&index=gi%7C392616944%7Cgb%7CEIW99372.1%7C&db_idx=1&px=1&section=5&ave_thresh=33&_ignoreionsscorebelow=0&report=0&_sigthreshold=0.05&_msresflags=1089&_msresflags2=2&percolate=-1&percolate_rt=0&_minpeplen=5&sessionID=all_secdisabledsession) | **752.3783** | **1502.7420** | **1502.7325** | **6.37** | **0** | **59** | **0.00019** | **1** | **U** | **K.SGVSLSAEQNENLR.S** [2266](http://10.200.41.11/mascot/cgi/peptide_view.pl?file=..%2Fdata%2F20160418%2FF080008.dat&query=2266&hit=1&index=gi%7C392616944%7Cgb%7CEIW99372.1%7C&db_idx=1&px=1&section=5&ave_thresh=33&_ignoreionsscorebelow=0&report=0&_sigthreshold=0.05&_msresflags=1089&_msresflags2=2&percolate=-1&percolate_rt=0&_minpeplen=5&sessionID=all_secdisabledsession) |

### Protein sequence coverage: 4%

Matched peptides shown in ***bold red***.

| **1** | MLNIQNYSAS | PHPGIVAERP | QTPSASEHVE | TAVVPSTTEH | RGTDIISLSQ |
| --- | --- | --- | --- | --- | --- |
| **51** | AATKIQQAQQ | TLQSTPPISE | ENNDERTLAR | QQLTSSLNAL | AK**SGVSLSAE** |
| **101** | **QNENLR**SAFS | APTSALFSAS | PMAQPRTTIS | DAEIWDMVSQ | NISAIGDSYL |
| **151** | GVYENVVAVY | TDFYQAFSDI | LSKMGGWLLP | GKDGNTVKLD | VTSLKNDLNS |
| **201** | LVNKYNQINS | NTVLFPAQSG | SGVKVATEAE | ARQWLSELNL | PNSCLKSYGS |
| **251** | GYVVTVDLTP | LQKMVQDIDG | LGAPGKDSKL | EMDNAKYQAW | QSGFKAQEEN |
| **301** | MKTTLQTLTQ | KYSNANSLYD | NLVKVLSSTI | SSSLETAKSF | LQG |

| **4.** | [gi|194403640|gb|ACF63862.1|](http://10.200.41.11/mascot/cgi/protein_view.pl?file=..%2Fdata%2F20160418%2FF080008.dat&hit=gi%7C194403640%7Cgb%7CACF63862.1%7C&db_idx=1&px=1&ave_thresh=33&_ignoreionsscorebelow=0&report=0&_sigthreshold=0.05&_msresflags=1089&_msresflags2=2&percolate=-1&percolate_rt=0&_minpeplen=5&sessionID=all_secdisabledsession)    **Mass:** 62382    **Score:** 64     **Matches:** 3(1)  **Sequences:** 2(1)  **emPAI:** 0.09 |
| --- | --- |
|  | cell invasion protein SipB [Salmonella enterica subsp. enterica serovar Newport str. SL254] |

|  | **Query** | **Observed** | **Mr(expt)** | **Mr(calc)** | **ppm** | **Miss** | **Score** | **Expect** | **Rank** | **Unique** | **Peptide** |
| --- | --- | --- | --- | --- | --- | --- | --- | --- | --- | --- | --- |
|  | [582](http://10.200.41.11/mascot/cgi/peptide_view.pl?file=..%2Fdata%2F20160418%2FF080008.dat&query=582&hit=1&index=gi%7C194403640%7Cgb%7CACF63862.1%7C&db_idx=1&px=1&section=5&ave_thresh=33&_ignoreionsscorebelow=0&report=0&_sigthreshold=0.05&_msresflags=1089&_msresflags2=2&percolate=-1&percolate_rt=0&_minpeplen=5&sessionID=all_secdisabledsession) | **461.7259** | **921.4373** | **921.4304** | **7.44** | **0** | **47** | **0.002** | **1** | **U** | **R.SGYTQNPR.L** [581](http://10.200.41.11/mascot/cgi/peptide_view.pl?file=..%2Fdata%2F20160418%2FF080008.dat&query=581&hit=1&index=gi%7C194403640%7Cgb%7CACF63862.1%7C&db_idx=1&px=1&section=5&ave_thresh=33&_ignoreionsscorebelow=0&report=0&_sigthreshold=0.05&_msresflags=1089&_msresflags2=2&percolate=-1&percolate_rt=0&_minpeplen=5&sessionID=all_secdisabledsession) |
|  | [755](http://10.200.41.11/mascot/cgi/peptide_view.pl?file=..%2Fdata%2F20160418%2FF080008.dat&query=755&hit=4&index=gi%7C194403640%7Cgb%7CACF63862.1%7C&db_idx=1&px=1&section=5&ave_thresh=33&_ignoreionsscorebelow=0&report=0&_sigthreshold=0.05&_msresflags=1089&_msresflags2=2&percolate=-1&percolate_rt=0&_minpeplen=5&sessionID=all_secdisabledsession) | **490.7468** | **979.4790** | **979.4909** | **-12.15** | **0** | **2** | **1.2e+002** | **4** | **U** | **K.LFTQGMQR.I** |

### Protein sequence coverage: 2%

Matched peptides shown in ***bold red***.

| **1** | MVNDASSISR | **SGYTQNPR**LA | EAAFEGVRKN | TDFLKAADKA | FKDVVATKAG |
| --- | --- | --- | --- | --- | --- |
| **51** | DLKAGTKSGE | SAINTVGLKP | PTDAAREKLS | SEGQLTLLLG | KLMTLLGDVS |
| **101** | LSQLESRLAV | WQAMIESQKE | MGIQVSKEFQ | TALGEAQEAT | DLYEASIKKT |
| **151** | DTAKSVYDAA | AKKLTQAQNK | LQSLDPADPG | YAQAEAAVEQ | AGKEATEAKE |
| **201** | ALDKATDATV | KAGTDAKAKA | EKADNILTKF | QGTANAASQN | QVSQGEQDNL |
| **251** | SNVARLTMLM | AMFIEIVGKN | TEESLQNDLA | LFNALQEGRQ | AEMEKKSAEF |
| **301** | QEETRKAEET | NRIMGCIGKV | LGALLTIVSV | VAAVFTGGAS | LALAAVGLAV |
| **351** | MVADEIVKAA | TGVSFIQQAL | NPIMEHVLKP | LMELIGKAIT | KALEGLGVDK |
| **401** | KTAEMAGSIV | GAIVAAIAMV | AVIVVVAVVG | KGAAAKLGNA | LSKMMGETIK |
| **451** | KLVPNVLKQL | AQNGSK**LFTQ** | **GMQR**ITSGLG | NVGSKMGLQT | NALSKELVGN |
| **501** | TLNKVALGME | VTNTAAQSAG | GVAEGVFIKN | ASEALADFML | ARFAMDQIQQ |
| **551** | WLKQSVEIFG | ENQKVTAELQ | KAMSSAVQQN | ADASRFILRQ | SRA |

| **5.** | [gi|194402702|gb|ACF62924.1|](http://10.200.41.11/mascot/cgi/protein_view.pl?file=..%2Fdata%2F20160418%2FF080008.dat&hit=gi%7C194402702%7Cgb%7CACF62924.1%7C&db_idx=1&px=1&ave_thresh=33&_ignoreionsscorebelow=0&report=0&_sigthreshold=0.05&_msresflags=1089&_msresflags2=2&percolate=-1&percolate_rt=0&_minpeplen=5&sessionID=all_secdisabledsession)    **Mass:** 72333    **Score:** 62     **Matches:** 3(2)  **Sequences:** 2(1)  **emPAI:** 0.08 |
| --- | --- |
|  | cell invasion protein SipA [Salmonella enterica subsp. enterica serovar Newport str. SL254] |

|  | **Query** | **Observed** | **Mr(expt)** | **Mr(calc)** | **ppm** | **Miss** | **Score** | **Expect** | **Rank** | **Unique** | **Peptide** |
| --- | --- | --- | --- | --- | --- | --- | --- | --- | --- | --- | --- |
|  | [755](http://10.200.41.11/mascot/cgi/peptide_view.pl?file=..%2Fdata%2F20160418%2FF080008.dat&query=755&hit=1&index=gi%7C194402702%7Cgb%7CACF62924.1%7C&db_idx=1&px=1&section=5&ave_thresh=33&_ignoreionsscorebelow=0&report=0&_sigthreshold=0.05&_msresflags=1089&_msresflags2=2&percolate=-1&percolate_rt=0&_minpeplen=5&sessionID=all_secdisabledsession) | 490.7468 | 979.4790 | 979.4723 | 6.88 | 0 | 23 | 0.9 | 1 | U | R.TFIDNSQR.N |
|  | [1531](http://10.200.41.11/mascot/cgi/peptide_view.pl?file=..%2Fdata%2F20160418%2FF080008.dat&query=1531&hit=1&index=gi%7C194402702%7Cgb%7CACF62924.1%7C&db_idx=1&px=1&section=5&ave_thresh=33&_ignoreionsscorebelow=0&report=0&_sigthreshold=0.05&_msresflags=1089&_msresflags2=2&percolate=-1&percolate_rt=0&_minpeplen=5&sessionID=all_secdisabledsession) | **588.3081** | **1174.6017** | **1174.5942** | **6.33** | **0** | **42** | **0.013** | **1** | **U** | **K.LTQEQGTSVGR.E** [1532](http://10.200.41.11/mascot/cgi/peptide_view.pl?file=..%2Fdata%2F20160418%2FF080008.dat&query=1532&hit=1&index=gi%7C194402702%7Cgb%7CACF62924.1%7C&db_idx=1&px=1&section=5&ave_thresh=33&_ignoreionsscorebelow=0&report=0&_sigthreshold=0.05&_msresflags=1089&_msresflags2=2&percolate=-1&percolate_rt=0&_minpeplen=5&sessionID=all_secdisabledsession) |

### Protein sequence coverage: 2%

Matched peptides shown in ***bold red***.

| **1** | MQTEIKTQAT | NLAANLSAVR | ESATTTLSGE | IKGPQLEDFP | ALIKQASLDA |
| --- | --- | --- | --- | --- | --- |
| **51** | LFKCGKDAEA | LKEVFTNSNN | VAGKKAIMEF | AGLFRSALNA | TSDSPEAKTL |
| **101** | LMKVGAEYTA | QIIKDGLKEK | SAFGPWLPET | KKAEAKLENL | EKQLLDIIKN |
| **151** | NTGGELSKLS | TNLVMQEVMP | YIASCIEHNF | GCTLDPLTRS | NLTHLVDKAA |
| **201** | AKAVEALDMC | HQK**LTQEQGT** | **SVGR**EARHLE | MQTLIPLLLR | NVFAQIPADK |
| **251** | LPDPKIPEPA | AGPVPDGGKK | AEPTGININI | NIDSSNHSVD | NSKHINNSRS |
| **301** | HVDNSQRHID | NSNHDNSRKT | IDNSR**TFIDN** | **SQR**NGESHHS | TNSSNVSHSH |
| **351** | SRVDSTTHQT | ETAHSASTGA | IDHGIAGKID | VTAHATAEAV | TNASSESKDG |
| **401** | KVVTSEKGTT | GETTSFDEVD | GVTSKSIIGK | PVQATVHGVD | DNKQQSQTAE |
| **451** | IVNVKPLASQ | LAGVENVKTD | TLQSDTTVIT | GNKAGTTDND | NSQTDKTGPF |
| **501** | SGLKFKQNSF | LSTVPSVTNM | HSMHFDARET | FLGVIRKALE | PDTSTPFPVR |
| **551** | RAFDGLRAEI | LPNDTIKSAA | LKAQCSDIDK | HPELKAKMET | LKEVITHHPQ |
| **601** | KEKLAEIALQ | FAREAGLTRL | KGETDYVLSN | VLDGLIGDGS | WRAGPAYESY |
| **651** | LNKPGVDRVI | TTVDGLHMQR |  |  |  |

| **7.** | [gi|194404219|gb|ACF64441.1|](http://10.200.41.11/mascot/cgi/protein_view.pl?file=..%2Fdata%2F20160418%2FF080008.dat&hit=gi%7C194404219%7Cgb%7CACF64441.1%7C&db_idx=1&px=1&ave_thresh=33&_ignoreionsscorebelow=0&report=0&_sigthreshold=0.05&_msresflags=1089&_msresflags2=2&percolate=-1&percolate_rt=0&_minpeplen=5&sessionID=all_secdisabledsession)    **Mass:** 34155    **Score:** 25     **Matches:** 1(0)  **Sequences:** 1(0) |
| --- | --- |
|  | flagellar hook-associated protein 3 [Salmonella enterica subsp. enterica serovar Newport str. SL254] |

|  | **Query** | **Observed** | **Mr(expt)** | **Mr(calc)** | **ppm** | **Miss** | **Score** | **Expect** | **Rank** | **Unique** | **Peptide** |
| --- | --- | --- | --- | --- | --- | --- | --- | --- | --- | --- | --- |
|  | [1322](http://10.200.41.11/mascot/cgi/peptide_view.pl?file=..%2Fdata%2F20160418%2FF080008.dat&query=1322&hit=1&index=gi%7C194404219%7Cgb%7CACF64441.1%7C&db_idx=1&px=1&section=5&ave_thresh=33&_ignoreionsscorebelow=0&report=0&_sigthreshold=0.05&_msresflags=1089&_msresflags2=2&percolate=-1&percolate_rt=0&_minpeplen=5&sessionID=all_secdisabledsession) | **561.7860** | **1121.5575** | **1121.5499** | **6.75** | **1** | **25** | **0.54** | **1** | **U** | **K.LGEQMSTGKR.V** |

### Protein sequence coverage: 3%

Matched peptides shown in ***bold red***.

| **1** | MRISTQMMYE | QNMSGITNSQ | AEWMK**LGEQM** | **STGKR**VTNPS | DDPIAASQAV |
| --- | --- | --- | --- | --- | --- |
| **51** | VLSQAQAQNS | QYALARTFAT | QKVSLEESVL | SQVTTAIQTA | QEKIVYAGNG |
| **101** | TLSDDDRASL | ATDLQGIRDQ | LMNLANSTDG | NGRYIFAGYK | TEAAPFDQAT |
| **151** | GGYHGGEKSV | TQQVDSARTM | VIGHTGAQIF | NSITSNAVPE | PDGSDSEKNL |
| **201** | FVMLDTAIAA | LKTPVEGNDV | EKEKAAAAID | KTNRGLKNSL | NNVLTVRAEL |
| **251** | GTQLSELSTL | DSLGSDRALG | QKLQMSNLVD | VDWNSVISSY | VMQQAALQAS |
| **301** | YKTFTDMQGM | SLFQLNR |  |  |  |

****************************************************15 minute SS Control*****************************************************

**1^st^ technical replicate**

**Enzyme : Trypsin**

**Variable modifications :** [**Oxidation (M)**](http://10.200.41.11/mascot/cgi/client.pl?modification&mod_name=Oxidation%20%28M%29&file=..%2Fdata%2F20160414%2FF079938.dat)

**Mass values : Monoisotopic**

**Protein Mass : Unrestricted**

**Peptide Mass Tolerance : ± 20 ppm**

**Fragment Mass Tolerance: ± 0.4 Da**

**Max Missed Cleavages : 3**

**Instrument type : Default**

**Number of queries : 2919**

| **Protein hits           :** | [**gi|764048859|gb|AJQ67632.1|**](http://10.200.41.11/mascot/cgi/master_results.pl?file=..%2Fdata%2F20160414%2FF079938.dat#Hit1) | Cof-like hydrolase [Salmonella enterica subsp. enterica serovar Newport str. USDA-ARS-USMARC-1927] |
| --- | --- | --- |

**************************************************************************************************************************

**2^nd^ technical replicate**

**Enzyme : Trypsin**

**Variable modifications :** [**Oxidation (M)**](http://10.200.41.11/mascot/cgi/client.pl?modification&mod_name=Oxidation%20%28M%29&file=..%2Fdata%2F20160414%2FF079940.dat)

**Mass values : Monoisotopic**

**Protein Mass : Unrestricted**

**Peptide Mass Tolerance : ± 20 ppm**

**Fragment Mass Tolerance: ± 0.4 Da**

**Max Missed Cleavages : 3**

**Instrument type : Default**

**Number of queries : 2808**

| **Protein hits           :** | [**gi|764048859|gb|AJQ67632.1|**](http://10.200.41.11/mascot/cgi/master_results.pl?file=..%2Fdata%2F20160414%2FF079940.dat#Hit1) | Cof-like hydrolase [Salmonella enterica subsp. enterica serovar Newport str. USDA-ARS-USMARC-1927] |
| --- | --- | --- |
|  | [**gi|195630380|gb|EDX49006.1|**](http://10.200.41.11/mascot/cgi/master_results.pl?file=..%2Fdata%2F20160414%2FF079940.dat#Hit2) | protein RecT [Salmonella enterica subsp. enterica serovar Newport str. SL317] |

**************************************************************************************************************************

**3^rd^ technical replicate**

**Enzyme : Trypsin**

**Variable modifications :** [**Oxidation (M)**](http://10.200.41.11/mascot/cgi/client.pl?modification&mod_name=Oxidation%20%28M%29&file=..%2Fdata%2F20160414%2FF079942.dat)

**Mass values : Monoisotopic**

**Protein Mass : Unrestricted**

**Peptide Mass Tolerance : ± 20 ppm**

**Fragment Mass Tolerance: ± 0.4 Da**

**Max Missed Cleavages : 3**

**Instrument type : Default**

**Number of queries : 3043**

**No identifications.**

*********************************************************20 Hour Re-Digest*****************************************************

**1^st^ technical replicate**

**Enzyme : Trypsin**

**Variable modifications :** [**Oxidation (M)**](http://10.200.41.11/mascot/cgi/client.pl?modification&mod_name=Oxidation%20%28M%29&file=..%2Fdata%2F20160418%2FF080011.dat)

**Mass values : Monoisotopic**

**Protein Mass : Unrestricted**

**Peptide Mass Tolerance : ± 20 ppm**

**Fragment Mass Tolerance: ± 0.4 Da**

**Max Missed Cleavages : 3**

**Instrument type : Default**

**Number of queries : 3237**

| **Protein hits           :** | [**gi|392616945|gb|EIW99373.1|**](http://10.200.41.11/mascot/cgi/master_results.pl?file=..%2Fdata%2F20160418%2FF080011.dat#Hit1) | pathogenicity island 1 effector protein SipC [Salmonella enterica subsp. enterica serovar Newport str. Levine 15] |
| --- | --- | --- |
|  | [**gi|50830890|gb|AAT81610.1|**](http://10.200.41.11/mascot/cgi/master_results.pl?file=..%2Fdata%2F20160418%2FF080011.dat#Hit2) | phase 1 flagellin [Salmonella enterica subsp. enterica serovar Newport] |
|  | [**gi|194401698|gb|ACF61920.1|**](http://10.200.41.11/mascot/cgi/master_results.pl?file=..%2Fdata%2F20160418%2FF080011.dat#Hit3) | translation elongation factor Tu [Salmonella enterica subsp. enterica serovar Newport str. SL254] |
|  | [**gi|194403331|gb|ACF63553.1|**](http://10.200.41.11/mascot/cgi/master_results.pl?file=..%2Fdata%2F20160418%2FF080011.dat#Hit4) | negative regulator of flagellin synthesis [Salmonella enterica subsp. enterica serovar Newport str. SL254] |
|  | [**gi|194402702|gb|ACF62924.1|**](http://10.200.41.11/mascot/cgi/master_results.pl?file=..%2Fdata%2F20160418%2FF080011.dat#Hit5) | cell invasion protein SipA [Salmonella enterica subsp. enterica serovar Newport str. SL254] |
|  | [**gi|194401103|gb|ACF61325.1|**](http://10.200.41.11/mascot/cgi/master_results.pl?file=..%2Fdata%2F20160418%2FF080011.dat#Hit6) | ribosomal protein L6 [Salmonella enterica subsp. enterica serovar Newport str. SL254] |
|  | [**gi|194401176|gb|ACF61398.1|**](http://10.200.41.11/mascot/cgi/master_results.pl?file=..%2Fdata%2F20160418%2FF080011.dat#Hit7) | ribosomal protein L7/L12 [Salmonella enterica subsp. enterica serovar Newport str. SL254] |
|  | [**gi|194404219|gb|ACF64441.1|**](http://10.200.41.11/mascot/cgi/master_results.pl?file=..%2Fdata%2F20160418%2FF080011.dat#Hit8) | flagellar hook-associated protein 3 [Salmonella enterica subsp. enterica serovar Newport str. SL254] |
|  | [**gi|392616944|gb|EIW99372.1|**](http://10.200.41.11/mascot/cgi/master_results.pl?file=..%2Fdata%2F20160418%2FF080011.dat#Hit9) | cell invasion protein SipD [Salmonella enterica subsp. enterica serovar Newport str. Levine 15] |
|  | [**gi|194401878|gb|ACF62100.1|**](http://10.200.41.11/mascot/cgi/master_results.pl?file=..%2Fdata%2F20160418%2FF080011.dat#Hit10) | ribosomal protein L11 [Salmonella enterica subsp. enterica serovar Newport str. SL254] |
|  | [**gi|194402309|gb|ACF62531.1|**](http://10.200.41.11/mascot/cgi/master_results.pl?file=..%2Fdata%2F20160418%2FF080011.dat#Hit11) | ribosomal protein S10 [Salmonella enterica subsp. enterica serovar Newport str. SL254] |
|  | [**gi|194404381|gb|ACF64603.1|**](http://10.200.41.11/mascot/cgi/master_results.pl?file=..%2Fdata%2F20160418%2FF080011.dat#Hit12) | DNA-binding protein HU-alpha [Salmonella enterica subsp. enterica serovar Newport str. SL254] |
|  | [**gi|194403640|gb|ACF63862.1|**](http://10.200.41.11/mascot/cgi/master_results.pl?file=..%2Fdata%2F20160418%2FF080011.dat#Hit13) | cell invasion protein SipB [Salmonella enterica subsp. enterica serovar Newport str. SL254] |
|  | [**gi|194403829|gb|ACF64051.1|**](http://10.200.41.11/mascot/cgi/master_results.pl?file=..%2Fdata%2F20160418%2FF080011.dat#Hit14) | ribosomal protein L3 [Salmonella enterica subsp. enterica serovar Newport str. SL254] |
|  | [**gi|194405415|gb|ACF65637.1|**](http://10.200.41.11/mascot/cgi/master_results.pl?file=..%2Fdata%2F20160418%2FF080011.dat#Hit15) | ribosomal protein L15 [Salmonella enterica subsp. enterica serovar Newport str. SL254] |
|  | [**gi|194405308|gb|ACF65530.1|**](http://10.200.41.11/mascot/cgi/master_results.pl?file=..%2Fdata%2F20160418%2FF080011.dat#Hit16) | DNA-binding protein HU-beta [Salmonella enterica subsp. enterica serovar Newport str. SL254] |
|  | [**gi|194404930|gb|ACF65152.1|**](http://10.200.41.11/mascot/cgi/master_results.pl?file=..%2Fdata%2F20160418%2FF080011.dat#Hit17) | H-NS histone family protein [Salmonella enterica subsp. enterica serovar Newport str. SL254] |

| **1.** | [gi|392616945|gb|EIW99373.1|](http://10.200.41.11/mascot/cgi/protein_view.pl?file=..%2Fdata%2F20160418%2FF080011.dat&hit=gi%7C392616945%7Cgb%7CEIW99373.1%7C&db_idx=1&px=1&ave_thresh=33&_ignoreionsscorebelow=0&report=0&_sigthreshold=0.05&_msresflags=1089&_msresflags2=2&percolate=-1&percolate_rt=0&_minpeplen=5&sessionID=all_secdisabledsession)    **Mass:** 42957    **Score:** 662    **Matches:** 34(19)  **Sequences:** 9(7)  **emPAI:** 1.42 |
| --- | --- |
|  | pathogenicity island 1 effector protein SipC [Salmonella enterica subsp. enterica serovar Newport str. Levine 15] |

|  | **Query** | **Observed** | **Mr(expt)** | **Mr(calc)** | **ppm** | **Miss** | **Score** | **Expect** | **Rank** | **Unique** | **Peptide** |
| --- | --- | --- | --- | --- | --- | --- | --- | --- | --- | --- | --- |
|  | [1321](http://10.200.41.11/mascot/cgi/peptide_view.pl?file=..%2Fdata%2F20160418%2FF080011.dat&query=1321&hit=1&index=gi%7C392616945%7Cgb%7CEIW99373.1%7C&db_idx=1&px=1&section=5&ave_thresh=33&_ignoreionsscorebelow=0&report=0&_sigthreshold=0.05&_msresflags=1089&_msresflags2=2&percolate=-1&percolate_rt=0&_minpeplen=5&sessionID=all_secdisabledsession) | **494.7726** | **987.5307** | **987.5237** | **7.10** | **0** | **57** | **0.00046** | **1** | **U** | **K.LGAEGVDSLK.S** [1320](http://10.200.41.11/mascot/cgi/peptide_view.pl?file=..%2Fdata%2F20160418%2FF080011.dat&query=1320&hit=1&index=gi%7C392616945%7Cgb%7CEIW99373.1%7C&db_idx=1&px=1&section=5&ave_thresh=33&_ignoreionsscorebelow=0&report=0&_sigthreshold=0.05&_msresflags=1089&_msresflags2=2&percolate=-1&percolate_rt=0&_minpeplen=5&sessionID=all_secdisabledsession) [1322](http://10.200.41.11/mascot/cgi/peptide_view.pl?file=..%2Fdata%2F20160418%2FF080011.dat&query=1322&hit=1&index=gi%7C392616945%7Cgb%7CEIW99373.1%7C&db_idx=1&px=1&section=5&ave_thresh=33&_ignoreionsscorebelow=0&report=0&_sigthreshold=0.05&_msresflags=1089&_msresflags2=2&percolate=-1&percolate_rt=0&_minpeplen=5&sessionID=all_secdisabledsession) [1323](http://10.200.41.11/mascot/cgi/peptide_view.pl?file=..%2Fdata%2F20160418%2FF080011.dat&query=1323&hit=1&index=gi%7C392616945%7Cgb%7CEIW99373.1%7C&db_idx=1&px=1&section=5&ave_thresh=33&_ignoreionsscorebelow=0&report=0&_sigthreshold=0.05&_msresflags=1089&_msresflags2=2&percolate=-1&percolate_rt=0&_minpeplen=5&sessionID=all_secdisabledsession) [1325](http://10.200.41.11/mascot/cgi/peptide_view.pl?file=..%2Fdata%2F20160418%2FF080011.dat&query=1325&hit=1&index=gi%7C392616945%7Cgb%7CEIW99373.1%7C&db_idx=1&px=1&section=5&ave_thresh=33&_ignoreionsscorebelow=0&report=0&_sigthreshold=0.05&_msresflags=1089&_msresflags2=2&percolate=-1&percolate_rt=0&_minpeplen=5&sessionID=all_secdisabledsession) [1326](http://10.200.41.11/mascot/cgi/peptide_view.pl?file=..%2Fdata%2F20160418%2FF080011.dat&query=1326&hit=1&index=gi%7C392616945%7Cgb%7CEIW99373.1%7C&db_idx=1&px=1&section=5&ave_thresh=33&_ignoreionsscorebelow=0&report=0&_sigthreshold=0.05&_msresflags=1089&_msresflags2=2&percolate=-1&percolate_rt=0&_minpeplen=5&sessionID=all_secdisabledsession) |
|  | [1580](http://10.200.41.11/mascot/cgi/peptide_view.pl?file=..%2Fdata%2F20160418%2FF080011.dat&query=1580&hit=1&index=gi%7C392616945%7Cgb%7CEIW99373.1%7C&db_idx=1&px=1&section=5&ave_thresh=33&_ignoreionsscorebelow=0&report=0&_sigthreshold=0.05&_msresflags=1089&_msresflags2=2&percolate=-1&percolate_rt=0&_minpeplen=5&sessionID=all_secdisabledsession) | **519.2541** | **1036.4937** | **1036.4859** | **7.59** | **0** | **37** | **0.02** | **1** | **U** | **K.TMESINQSK.A** [1581](http://10.200.41.11/mascot/cgi/peptide_view.pl?file=..%2Fdata%2F20160418%2FF080011.dat&query=1581&hit=1&index=gi%7C392616945%7Cgb%7CEIW99373.1%7C&db_idx=1&px=1&section=5&ave_thresh=33&_ignoreionsscorebelow=0&report=0&_sigthreshold=0.05&_msresflags=1089&_msresflags2=2&percolate=-1&percolate_rt=0&_minpeplen=5&sessionID=all_secdisabledsession) [1582](http://10.200.41.11/mascot/cgi/peptide_view.pl?file=..%2Fdata%2F20160418%2FF080011.dat&query=1582&hit=1&index=gi%7C392616945%7Cgb%7CEIW99373.1%7C&db_idx=1&px=1&section=5&ave_thresh=33&_ignoreionsscorebelow=0&report=0&_sigthreshold=0.05&_msresflags=1089&_msresflags2=2&percolate=-1&percolate_rt=0&_minpeplen=5&sessionID=all_secdisabledsession) |
|  | [1759](http://10.200.41.11/mascot/cgi/peptide_view.pl?file=..%2Fdata%2F20160418%2FF080011.dat&query=1759&hit=1&index=gi%7C392616945%7Cgb%7CEIW99373.1%7C&db_idx=1&px=1&section=5&ave_thresh=33&_ignoreionsscorebelow=0&report=0&_sigthreshold=0.05&_msresflags=1089&_msresflags2=2&percolate=-1&percolate_rt=0&_minpeplen=5&sessionID=all_secdisabledsession) | **536.7950** | **1071.5754** | **1071.5673** | **7.61** | **0** | **45** | **0.0052** | **1** | **U** | **K.NVLNGQNSVK.L** [1757](http://10.200.41.11/mascot/cgi/peptide_view.pl?file=..%2Fdata%2F20160418%2FF080011.dat&query=1757&hit=1&index=gi%7C392616945%7Cgb%7CEIW99373.1%7C&db_idx=1&px=1&section=5&ave_thresh=33&_ignoreionsscorebelow=0&report=0&_sigthreshold=0.05&_msresflags=1089&_msresflags2=2&percolate=-1&percolate_rt=0&_minpeplen=5&sessionID=all_secdisabledsession) [1758](http://10.200.41.11/mascot/cgi/peptide_view.pl?file=..%2Fdata%2F20160418%2FF080011.dat&query=1758&hit=1&index=gi%7C392616945%7Cgb%7CEIW99373.1%7C&db_idx=1&px=1&section=5&ave_thresh=33&_ignoreionsscorebelow=0&report=0&_sigthreshold=0.05&_msresflags=1089&_msresflags2=2&percolate=-1&percolate_rt=0&_minpeplen=5&sessionID=all_secdisabledsession) |
|  | [2330](http://10.200.41.11/mascot/cgi/peptide_view.pl?file=..%2Fdata%2F20160418%2FF080011.dat&query=2330&hit=1&index=gi%7C392616945%7Cgb%7CEIW99373.1%7C&db_idx=1&px=1&section=5&ave_thresh=33&_ignoreionsscorebelow=0&report=0&_sigthreshold=0.05&_msresflags=1089&_msresflags2=2&percolate=-1&percolate_rt=0&_minpeplen=5&sessionID=all_secdisabledsession) | **602.3245** | **1202.6345** | **1202.6255** | **7.49** | **1** | **43** | **0.0096** | **1** | **U** | **K.RLESVESDIR.L** [2327](http://10.200.41.11/mascot/cgi/peptide_view.pl?file=..%2Fdata%2F20160418%2FF080011.dat&query=2327&hit=1&index=gi%7C392616945%7Cgb%7CEIW99373.1%7C&db_idx=1&px=1&section=5&ave_thresh=33&_ignoreionsscorebelow=0&report=0&_sigthreshold=0.05&_msresflags=1089&_msresflags2=2&percolate=-1&percolate_rt=0&_minpeplen=5&sessionID=all_secdisabledsession) [2328](http://10.200.41.11/mascot/cgi/peptide_view.pl?file=..%2Fdata%2F20160418%2FF080011.dat&query=2328&hit=1&index=gi%7C392616945%7Cgb%7CEIW99373.1%7C&db_idx=1&px=1&section=5&ave_thresh=33&_ignoreionsscorebelow=0&report=0&_sigthreshold=0.05&_msresflags=1089&_msresflags2=2&percolate=-1&percolate_rt=0&_minpeplen=5&sessionID=all_secdisabledsession) [2329](http://10.200.41.11/mascot/cgi/peptide_view.pl?file=..%2Fdata%2F20160418%2FF080011.dat&query=2329&hit=1&index=gi%7C392616945%7Cgb%7CEIW99373.1%7C&db_idx=1&px=1&section=5&ave_thresh=33&_ignoreionsscorebelow=0&report=0&_sigthreshold=0.05&_msresflags=1089&_msresflags2=2&percolate=-1&percolate_rt=0&_minpeplen=5&sessionID=all_secdisabledsession) |
|  | [2694](http://10.200.41.11/mascot/cgi/peptide_view.pl?file=..%2Fdata%2F20160418%2FF080011.dat&query=2694&hit=1&index=gi%7C392616945%7Cgb%7CEIW99373.1%7C&db_idx=1&px=1&section=5&ave_thresh=33&_ignoreionsscorebelow=0&report=0&_sigthreshold=0.05&_msresflags=1089&_msresflags2=2&percolate=-1&percolate_rt=0&_minpeplen=5&sessionID=all_secdisabledsession) | **651.8282** | **1301.6419** | **1301.6324** | **7.35** | **0** | **69** | **1.7e-005** | **1** | **U** | **R.SEQQISQVNNR.V** [2692](http://10.200.41.11/mascot/cgi/peptide_view.pl?file=..%2Fdata%2F20160418%2FF080011.dat&query=2692&hit=1&index=gi%7C392616945%7Cgb%7CEIW99373.1%7C&db_idx=1&px=1&section=5&ave_thresh=33&_ignoreionsscorebelow=0&report=0&_sigthreshold=0.05&_msresflags=1089&_msresflags2=2&percolate=-1&percolate_rt=0&_minpeplen=5&sessionID=all_secdisabledsession) [2693](http://10.200.41.11/mascot/cgi/peptide_view.pl?file=..%2Fdata%2F20160418%2FF080011.dat&query=2693&hit=1&index=gi%7C392616945%7Cgb%7CEIW99373.1%7C&db_idx=1&px=1&section=5&ave_thresh=33&_ignoreionsscorebelow=0&report=0&_sigthreshold=0.05&_msresflags=1089&_msresflags2=2&percolate=-1&percolate_rt=0&_minpeplen=5&sessionID=all_secdisabledsession) [2695](http://10.200.41.11/mascot/cgi/peptide_view.pl?file=..%2Fdata%2F20160418%2FF080011.dat&query=2695&hit=1&index=gi%7C392616945%7Cgb%7CEIW99373.1%7C&db_idx=1&px=1&section=5&ave_thresh=33&_ignoreionsscorebelow=0&report=0&_sigthreshold=0.05&_msresflags=1089&_msresflags2=2&percolate=-1&percolate_rt=0&_minpeplen=5&sessionID=all_secdisabledsession) [2696](http://10.200.41.11/mascot/cgi/peptide_view.pl?file=..%2Fdata%2F20160418%2FF080011.dat&query=2696&hit=1&index=gi%7C392616945%7Cgb%7CEIW99373.1%7C&db_idx=1&px=1&section=5&ave_thresh=33&_ignoreionsscorebelow=0&report=0&_sigthreshold=0.05&_msresflags=1089&_msresflags2=2&percolate=-1&percolate_rt=0&_minpeplen=5&sessionID=all_secdisabledsession) |
|  | [2797](http://10.200.41.11/mascot/cgi/peptide_view.pl?file=..%2Fdata%2F20160418%2FF080011.dat&query=2797&hit=1&index=gi%7C392616945%7Cgb%7CEIW99373.1%7C&db_idx=1&px=1&section=5&ave_thresh=33&_ignoreionsscorebelow=0&report=0&_sigthreshold=0.05&_msresflags=1089&_msresflags2=2&percolate=-1&percolate_rt=0&_minpeplen=5&sessionID=all_secdisabledsession) | **668.3461** | **1334.6777** | **1334.6678** | **7.46** | **0** | **62** | **0.00012** | **1** | **U** | **K.SNAGTSATESLGIK.D** [2795](http://10.200.41.11/mascot/cgi/peptide_view.pl?file=..%2Fdata%2F20160418%2FF080011.dat&query=2795&hit=1&index=gi%7C392616945%7Cgb%7CEIW99373.1%7C&db_idx=1&px=1&section=5&ave_thresh=33&_ignoreionsscorebelow=0&report=0&_sigthreshold=0.05&_msresflags=1089&_msresflags2=2&percolate=-1&percolate_rt=0&_minpeplen=5&sessionID=all_secdisabledsession) [2796](http://10.200.41.11/mascot/cgi/peptide_view.pl?file=..%2Fdata%2F20160418%2FF080011.dat&query=2796&hit=1&index=gi%7C392616945%7Cgb%7CEIW99373.1%7C&db_idx=1&px=1&section=5&ave_thresh=33&_ignoreionsscorebelow=0&report=0&_sigthreshold=0.05&_msresflags=1089&_msresflags2=2&percolate=-1&percolate_rt=0&_minpeplen=5&sessionID=all_secdisabledsession) [2798](http://10.200.41.11/mascot/cgi/peptide_view.pl?file=..%2Fdata%2F20160418%2FF080011.dat&query=2798&hit=1&index=gi%7C392616945%7Cgb%7CEIW99373.1%7C&db_idx=1&px=1&section=5&ave_thresh=33&_ignoreionsscorebelow=0&report=0&_sigthreshold=0.05&_msresflags=1089&_msresflags2=2&percolate=-1&percolate_rt=0&_minpeplen=5&sessionID=all_secdisabledsession) |
|  | [2832](http://10.200.41.11/mascot/cgi/peptide_view.pl?file=..%2Fdata%2F20160418%2FF080011.dat&query=2832&hit=1&index=gi%7C392616945%7Cgb%7CEIW99373.1%7C&db_idx=1&px=1&section=5&ave_thresh=33&_ignoreionsscorebelow=0&report=0&_sigthreshold=0.05&_msresflags=1089&_msresflags2=2&percolate=-1&percolate_rt=0&_minpeplen=5&sessionID=all_secdisabledsession) | **675.8774** | **1349.7403** | **1349.7303** | **7.44** | **0** | **37** | **0.04** | **1** | **U** | **K.QISPEHQAILSK.R** |
|  | [2854](http://10.200.41.11/mascot/cgi/peptide_view.pl?file=..%2Fdata%2F20160418%2FF080011.dat&query=2854&hit=1&index=gi%7C392616945%7Cgb%7CEIW99373.1%7C&db_idx=1&px=1&section=5&ave_thresh=33&_ignoreionsscorebelow=0&report=0&_sigthreshold=0.05&_msresflags=1089&_msresflags2=2&percolate=-1&percolate_rt=0&_minpeplen=5&sessionID=all_secdisabledsession) | **457.9247** | **1370.7523** | **1370.7405** | **8.60** | **1** | **26** | **0.5** | **1** | **U** | **K.IDKLTTESHSIK.N** [2850](http://10.200.41.11/mascot/cgi/peptide_view.pl?file=..%2Fdata%2F20160418%2FF080011.dat&query=2850&hit=1&index=gi%7C392616945%7Cgb%7CEIW99373.1%7C&db_idx=1&px=1&section=5&ave_thresh=33&_ignoreionsscorebelow=0&report=0&_sigthreshold=0.05&_msresflags=1089&_msresflags2=2&percolate=-1&percolate_rt=0&_minpeplen=5&sessionID=all_secdisabledsession) [2851](http://10.200.41.11/mascot/cgi/peptide_view.pl?file=..%2Fdata%2F20160418%2FF080011.dat&query=2851&hit=1&index=gi%7C392616945%7Cgb%7CEIW99373.1%7C&db_idx=1&px=1&section=5&ave_thresh=33&_ignoreionsscorebelow=0&report=0&_sigthreshold=0.05&_msresflags=1089&_msresflags2=2&percolate=-1&percolate_rt=0&_minpeplen=5&sessionID=all_secdisabledsession) [2852](http://10.200.41.11/mascot/cgi/peptide_view.pl?file=..%2Fdata%2F20160418%2FF080011.dat&query=2852&hit=1&index=gi%7C392616945%7Cgb%7CEIW99373.1%7C&db_idx=1&px=1&section=5&ave_thresh=33&_ignoreionsscorebelow=0&report=0&_sigthreshold=0.05&_msresflags=1089&_msresflags2=2&percolate=-1&percolate_rt=0&_minpeplen=5&sessionID=all_secdisabledsession) [2853](http://10.200.41.11/mascot/cgi/peptide_view.pl?file=..%2Fdata%2F20160418%2FF080011.dat&query=2853&hit=1&index=gi%7C392616945%7Cgb%7CEIW99373.1%7C&db_idx=1&px=1&section=5&ave_thresh=33&_ignoreionsscorebelow=0&report=0&_sigthreshold=0.05&_msresflags=1089&_msresflags2=2&percolate=-1&percolate_rt=0&_minpeplen=5&sessionID=all_secdisabledsession) |
|  | [3209](http://10.200.41.11/mascot/cgi/peptide_view.pl?file=..%2Fdata%2F20160418%2FF080011.dat&query=3209&hit=1&index=gi%7C392616945%7Cgb%7CEIW99373.1%7C&db_idx=1&px=1&section=5&ave_thresh=33&_ignoreionsscorebelow=0&report=0&_sigthreshold=0.05&_msresflags=1089&_msresflags2=2&percolate=-1&percolate_rt=0&_minpeplen=5&sessionID=all_secdisabledsession) | **593.9666** | **1778.8780** | **1778.8646** | **7.54** | **1** | **24** | **0.72** | **1** | **U** | **K.SNAGTSATESLGIKDSNK.Q** [3208](http://10.200.41.11/mascot/cgi/peptide_view.pl?file=..%2Fdata%2F20160418%2FF080011.dat&query=3208&hit=1&index=gi%7C392616945%7Cgb%7CEIW99373.1%7C&db_idx=1&px=1&section=5&ave_thresh=33&_ignoreionsscorebelow=0&report=0&_sigthreshold=0.05&_msresflags=1089&_msresflags2=2&percolate=-1&percolate_rt=0&_minpeplen=5&sessionID=all_secdisabledsession) [3210](http://10.200.41.11/mascot/cgi/peptide_view.pl?file=..%2Fdata%2F20160418%2FF080011.dat&query=3210&hit=1&index=gi%7C392616945%7Cgb%7CEIW99373.1%7C&db_idx=1&px=1&section=5&ave_thresh=33&_ignoreionsscorebelow=0&report=0&_sigthreshold=0.05&_msresflags=1089&_msresflags2=2&percolate=-1&percolate_rt=0&_minpeplen=5&sessionID=all_secdisabledsession) |

### Protein sequence coverage: 22%

Matched peptides shown in ***bold red***.

| **1** | MLISNVGINP | AAYLNNHSVE | NSSQTASQSV | SAKDILNSIG | ISSSKVSDLG |
| --- | --- | --- | --- | --- | --- |
| **51** | LSPTLSAPAP | GVLTQTPGTI | TSFLKASIQN | TDMNQDLNAL | ANNVTTKANE |
| **101** | VVQTQLREQQ | AEVGKFFDIS | GMSSSAVALL | AAANTLMLTL | NQADSKLSGK |
| **151** | LSLVSFDAAK | TTASSMMREG | MNALSGSISQ | SALQLGITGV | GAKLEYKGLQ |
| **201** | NERGALKHNA | AK**IDKLTTES** | **HSIKNVLNGQ** | **NSVKLGAEGV** | **DSLK**SLNMKK |
| **251** | TGTDATKNLN | DATLK**SNAGT** | **SATESLGIKD** | **SNKQISPEHQ** | **AILSKRLESV** |
| **301** | **ESDIR**LEQNT | MDMTRIDARK | MQMTGDLIMK | NSVTVGGIAG | ASGQYAATQE |
| **351** | R**SEQQISQVN** | **NR**VASTASDE | ARESSRKSTS | LIQEMLK**TME** | **SINQSK**ASAL |
| **401** | AAIAGNIRA |  |  |  |  |

| **2.** | [gi|50830890|gb|AAT81610.1|](http://10.200.41.11/mascot/cgi/protein_view.pl?file=..%2Fdata%2F20160418%2FF080011.dat&hit=gi%7C50830890%7Cgb%7CAAT81610.1%7C&db_idx=1&px=1&ave_thresh=33&_ignoreionsscorebelow=0&report=0&_sigthreshold=0.05&_msresflags=1089&_msresflags2=2&percolate=-1&percolate_rt=0&_minpeplen=5&sessionID=all_secdisabledsession)    **Mass:** 52223    **Score:** 499    **Matches:** 21(15)  **Sequences:** 6(5)  **emPAI:** 0.68 |
| --- | --- |
|  | phase 1 flagellin [Salmonella enterica subsp. enterica serovar Newport] |

|  | **Query** | **Observed** | **Mr(expt)** | **Mr(calc)** | **ppm** | **Miss** | **Score** | **Expect** | **Rank** | **Unique** | **Peptide** |
| --- | --- | --- | --- | --- | --- | --- | --- | --- | --- | --- | --- |
|  | [1076](http://10.200.41.11/mascot/cgi/peptide_view.pl?file=..%2Fdata%2F20160418%2FF080011.dat&query=1076&hit=1&index=gi%7C50830890%7Cgb%7CAAT81610.1%7C&db_idx=1&px=1&section=5&ave_thresh=33&_ignoreionsscorebelow=0&report=0&_sigthreshold=0.05&_msresflags=1089&_msresflags2=2&percolate=-1&percolate_rt=0&_minpeplen=5&sessionID=all_secdisabledsession) | **481.2675** | **960.5205** | **960.5128** | **8.08** | **0** | **48** | **0.0029** | **1** | **U** | **K.TEVVTIDGK.T** [1074](http://10.200.41.11/mascot/cgi/peptide_view.pl?file=..%2Fdata%2F20160418%2FF080011.dat&query=1074&hit=1&index=gi%7C50830890%7Cgb%7CAAT81610.1%7C&db_idx=1&px=1&section=5&ave_thresh=33&_ignoreionsscorebelow=0&report=0&_sigthreshold=0.05&_msresflags=1089&_msresflags2=2&percolate=-1&percolate_rt=0&_minpeplen=5&sessionID=all_secdisabledsession) [1075](http://10.200.41.11/mascot/cgi/peptide_view.pl?file=..%2Fdata%2F20160418%2FF080011.dat&query=1075&hit=1&index=gi%7C50830890%7Cgb%7CAAT81610.1%7C&db_idx=1&px=1&section=5&ave_thresh=33&_ignoreionsscorebelow=0&report=0&_sigthreshold=0.05&_msresflags=1089&_msresflags2=2&percolate=-1&percolate_rt=0&_minpeplen=5&sessionID=all_secdisabledsession) |
|  | [1853](http://10.200.41.11/mascot/cgi/peptide_view.pl?file=..%2Fdata%2F20160418%2FF080011.dat&query=1853&hit=1&index=gi%7C50830890%7Cgb%7CAAT81610.1%7C&db_idx=1&px=1&section=5&ave_thresh=33&_ignoreionsscorebelow=0&report=0&_sigthreshold=0.05&_msresflags=1089&_msresflags2=2&percolate=-1&percolate_rt=0&_minpeplen=5&sessionID=all_secdisabledsession) | **551.2717** | **1100.5288** | **1100.5210** | **7.05** | **0** | **58** | **0.00014** | **1** | **U** | **K.DDAAGQAIANR.F** [1852](http://10.200.41.11/mascot/cgi/peptide_view.pl?file=..%2Fdata%2F20160418%2FF080011.dat&query=1852&hit=1&index=gi%7C50830890%7Cgb%7CAAT81610.1%7C&db_idx=1&px=1&section=5&ave_thresh=33&_ignoreionsscorebelow=0&report=0&_sigthreshold=0.05&_msresflags=1089&_msresflags2=2&percolate=-1&percolate_rt=0&_minpeplen=5&sessionID=all_secdisabledsession) [1854](http://10.200.41.11/mascot/cgi/peptide_view.pl?file=..%2Fdata%2F20160418%2FF080011.dat&query=1854&hit=1&index=gi%7C50830890%7Cgb%7CAAT81610.1%7C&db_idx=1&px=1&section=5&ave_thresh=33&_ignoreionsscorebelow=0&report=0&_sigthreshold=0.05&_msresflags=1089&_msresflags2=2&percolate=-1&percolate_rt=0&_minpeplen=5&sessionID=all_secdisabledsession) [1855](http://10.200.41.11/mascot/cgi/peptide_view.pl?file=..%2Fdata%2F20160418%2FF080011.dat&query=1855&hit=1&index=gi%7C50830890%7Cgb%7CAAT81610.1%7C&db_idx=1&px=1&section=5&ave_thresh=33&_ignoreionsscorebelow=0&report=0&_sigthreshold=0.05&_msresflags=1089&_msresflags2=2&percolate=-1&percolate_rt=0&_minpeplen=5&sessionID=all_secdisabledsession) |
|  | [1946](http://10.200.41.11/mascot/cgi/peptide_view.pl?file=..%2Fdata%2F20160418%2FF080011.dat&query=1946&hit=1&index=gi%7C50830890%7Cgb%7CAAT81610.1%7C&db_idx=1&px=1&section=5&ave_thresh=33&_ignoreionsscorebelow=0&report=0&_sigthreshold=0.05&_msresflags=1089&_msresflags2=2&percolate=-1&percolate_rt=0&_minpeplen=5&sessionID=all_secdisabledsession) | **565.7978** | **1129.5810** | **1129.5728** | **7.33** | **0** | **43** | **0.0098** | **1** | **U** | **K.TAANQLGGVDGK.T** |
|  | [1947](http://10.200.41.11/mascot/cgi/peptide_view.pl?file=..%2Fdata%2F20160418%2FF080011.dat&query=1947&hit=1&index=gi%7C50830890%7Cgb%7CAAT81610.1%7C&db_idx=1&px=1&section=5&ave_thresh=33&_ignoreionsscorebelow=0&report=0&_sigthreshold=0.05&_msresflags=1089&_msresflags2=2&percolate=-1&percolate_rt=0&_minpeplen=5&sessionID=all_secdisabledsession) | **566.8052** | **1131.5958** | **1131.5884** | **6.56** | **0** | **56** | **0.00051** | **1** | **U** | **K.SQSALGTAIER.L** [1948](http://10.200.41.11/mascot/cgi/peptide_view.pl?file=..%2Fdata%2F20160418%2FF080011.dat&query=1948&hit=1&index=gi%7C50830890%7Cgb%7CAAT81610.1%7C&db_idx=1&px=1&section=5&ave_thresh=33&_ignoreionsscorebelow=0&report=0&_sigthreshold=0.05&_msresflags=1089&_msresflags2=2&percolate=-1&percolate_rt=0&_minpeplen=5&sessionID=all_secdisabledsession) [1949](http://10.200.41.11/mascot/cgi/peptide_view.pl?file=..%2Fdata%2F20160418%2FF080011.dat&query=1949&hit=1&index=gi%7C50830890%7Cgb%7CAAT81610.1%7C&db_idx=1&px=1&section=5&ave_thresh=33&_ignoreionsscorebelow=0&report=0&_sigthreshold=0.05&_msresflags=1089&_msresflags2=2&percolate=-1&percolate_rt=0&_minpeplen=5&sessionID=all_secdisabledsession) [1950](http://10.200.41.11/mascot/cgi/peptide_view.pl?file=..%2Fdata%2F20160418%2FF080011.dat&query=1950&hit=1&index=gi%7C50830890%7Cgb%7CAAT81610.1%7C&db_idx=1&px=1&section=5&ave_thresh=33&_ignoreionsscorebelow=0&report=0&_sigthreshold=0.05&_msresflags=1089&_msresflags2=2&percolate=-1&percolate_rt=0&_minpeplen=5&sessionID=all_secdisabledsession) [1951](http://10.200.41.11/mascot/cgi/peptide_view.pl?file=..%2Fdata%2F20160418%2FF080011.dat&query=1951&hit=1&index=gi%7C50830890%7Cgb%7CAAT81610.1%7C&db_idx=1&px=1&section=5&ave_thresh=33&_ignoreionsscorebelow=0&report=0&_sigthreshold=0.05&_msresflags=1089&_msresflags2=2&percolate=-1&percolate_rt=0&_minpeplen=5&sessionID=all_secdisabledsession) [1952](http://10.200.41.11/mascot/cgi/peptide_view.pl?file=..%2Fdata%2F20160418%2FF080011.dat&query=1952&hit=1&index=gi%7C50830890%7Cgb%7CAAT81610.1%7C&db_idx=1&px=1&section=5&ave_thresh=33&_ignoreionsscorebelow=0&report=0&_sigthreshold=0.05&_msresflags=1089&_msresflags2=2&percolate=-1&percolate_rt=0&_minpeplen=5&sessionID=all_secdisabledsession) |
|  | [2091](http://10.200.41.11/mascot/cgi/peptide_view.pl?file=..%2Fdata%2F20160418%2FF080011.dat&query=2091&hit=1&index=gi%7C50830890%7Cgb%7CAAT81610.1%7C&db_idx=1&px=1&section=5&ave_thresh=33&_ignoreionsscorebelow=0&report=0&_sigthreshold=0.05&_msresflags=1089&_msresflags2=2&percolate=-1&percolate_rt=0&_minpeplen=5&sessionID=all_secdisabledsession) | **582.8082** | **1163.6018** | **1163.5935** | **7.11** | **0** | **56** | **0.0005** | **1** | **U** | **R.VSGQTQFNGVK.V** [2092](http://10.200.41.11/mascot/cgi/peptide_view.pl?file=..%2Fdata%2F20160418%2FF080011.dat&query=2092&hit=1&index=gi%7C50830890%7Cgb%7CAAT81610.1%7C&db_idx=1&px=1&section=5&ave_thresh=33&_ignoreionsscorebelow=0&report=0&_sigthreshold=0.05&_msresflags=1089&_msresflags2=2&percolate=-1&percolate_rt=0&_minpeplen=5&sessionID=all_secdisabledsession) [2093](http://10.200.41.11/mascot/cgi/peptide_view.pl?file=..%2Fdata%2F20160418%2FF080011.dat&query=2093&hit=1&index=gi%7C50830890%7Cgb%7CAAT81610.1%7C&db_idx=1&px=1&section=5&ave_thresh=33&_ignoreionsscorebelow=0&report=0&_sigthreshold=0.05&_msresflags=1089&_msresflags2=2&percolate=-1&percolate_rt=0&_minpeplen=5&sessionID=all_secdisabledsession) |
|  | [3198](http://10.200.41.11/mascot/cgi/peptide_view.pl?file=..%2Fdata%2F20160418%2FF080011.dat&query=3198&hit=1&index=gi%7C50830890%7Cgb%7CAAT81610.1%7C&db_idx=1&px=1&section=5&ave_thresh=33&_ignoreionsscorebelow=0&report=0&_sigthreshold=0.05&_msresflags=1089&_msresflags2=2&percolate=-1&percolate_rt=0&_minpeplen=5&sessionID=all_secdisabledsession) | **539.9375** | **1616.7907** | **1616.8006** | **-6.11** | **1** | **2** | **89** | **1** | **U** | **K.KALEDGGVSNADATAAK.L** [3197](http://10.200.41.11/mascot/cgi/peptide_view.pl?file=..%2Fdata%2F20160418%2FF080011.dat&query=3197&hit=1&index=gi%7C50830890%7Cgb%7CAAT81610.1%7C&db_idx=1&px=1&section=5&ave_thresh=33&_ignoreionsscorebelow=0&report=0&_sigthreshold=0.05&_msresflags=1089&_msresflags2=2&percolate=-1&percolate_rt=0&_minpeplen=5&sessionID=all_secdisabledsession) [3199](http://10.200.41.11/mascot/cgi/peptide_view.pl?file=..%2Fdata%2F20160418%2FF080011.dat&query=3199&hit=1&index=gi%7C50830890%7Cgb%7CAAT81610.1%7C&db_idx=1&px=1&section=5&ave_thresh=33&_ignoreionsscorebelow=0&report=0&_sigthreshold=0.05&_msresflags=1089&_msresflags2=2&percolate=-1&percolate_rt=0&_minpeplen=5&sessionID=all_secdisabledsession) [3200](http://10.200.41.11/mascot/cgi/peptide_view.pl?file=..%2Fdata%2F20160418%2FF080011.dat&query=3200&hit=1&index=gi%7C50830890%7Cgb%7CAAT81610.1%7C&db_idx=1&px=1&section=5&ave_thresh=33&_ignoreionsscorebelow=0&report=0&_sigthreshold=0.05&_msresflags=1089&_msresflags2=2&percolate=-1&percolate_rt=0&_minpeplen=5&sessionID=all_secdisabledsession) |

### Protein sequence coverage: 14%

Matched peptides shown in ***bold red***.

| **1** | MAQVINTNSL | SLLTQNNLNK | **SQSALGTAIE** | **R**LSSGLRINS | AK**DDAAGQAI** |
| --- | --- | --- | --- | --- | --- |
| **51** | **ANR**FTANIKG | LTQASRNAND | GISIAQTTEG | ALNEINNNLQ | RVRELAVQSA |
| **101** | NSTNSQSDLD | SIQAEITQRL | NEIDR**VSGQT** | **QFNGVK**VLAQ | DNTLTIQVGA |
| **151** | NDGETIDIDL | KQINSQTLGL | DTLNVQKAYD | VSATAAMDPK | SFTDGTKNLT |
| **201** | APDATAIKAA | LGNPAATGDS | LSATLSFKDG | KYYATVAGYT | NAADTSKNGK |
| **251** | YEVNVDSATG | AVTFNAAPTK | ATVTGDTTVT | KVQVNAPVAV | STDVK**KALED** |
| **301** | **GGVSNADATA** | **AK**LVKMSYTD | KNGKSIDGGY | ALEAGGKYYA | ATYDEGTGKI |
| **351** | TANVTTYTDS | TGVTK**TAANQ** | **LGGVDGKTEV** | **VTIDGK**TYNA | SKAAGHDFKA |
| **401** | QPELAEAAAK | TTENPLAKID | AALAQVDALR | SDLGAVQNRF | NSAITNLGNT |
| **451** | VNNLSEARSR | IEDSDYATEV | SNMSRAQILQ | QAGTSVLAQA | NQVPQNVLSL |
| **501** | LR |  |  |  |  |

| **4.** | [gi|194403331|gb|ACF63553.1|](http://10.200.41.11/mascot/cgi/protein_view.pl?file=..%2Fdata%2F20160418%2FF080011.dat&hit=gi%7C194403331%7Cgb%7CACF63553.1%7C&db_idx=1&px=1&ave_thresh=33&_ignoreionsscorebelow=0&report=0&_sigthreshold=0.05&_msresflags=1089&_msresflags2=2&percolate=-1&percolate_rt=0&_minpeplen=5&sessionID=all_secdisabledsession)    **Mass:** 10561    **Score:** 314    **Matches:** 9(5)  **Sequences:** 3(2)  **emPAI:** 1.68 |
| --- | --- |
|  | negative regulator of flagellin synthesis [Salmonella enterica subsp. enterica serovar Newport str. SL254] |

|  | **Query** | **Observed** | **Mr(expt)** | **Mr(calc)** | **ppm** | **Miss** | **Score** | **Expect** | **Rank** | **Unique** | **Peptide** |
| --- | --- | --- | --- | --- | --- | --- | --- | --- | --- | --- | --- |
|  | [2933](http://10.200.41.11/mascot/cgi/peptide_view.pl?file=..%2Fdata%2F20160418%2FF080011.dat&query=2933&hit=1&index=gi%7C194403331%7Cgb%7CACF63553.1%7C&db_idx=1&px=1&section=5&ave_thresh=33&_ignoreionsscorebelow=0&report=0&_sigthreshold=0.05&_msresflags=1089&_msresflags2=2&percolate=-1&percolate_rt=0&_minpeplen=5&sessionID=all_secdisabledsession) | **707.4117** | **1412.8088** | **1412.7987** | **7.13** | **0** | **33** | **0.069** | **1** | **U** | **R.TSPLKPVSTVQTR.E** [2934](http://10.200.41.11/mascot/cgi/peptide_view.pl?file=..%2Fdata%2F20160418%2FF080011.dat&query=2934&hit=1&index=gi%7C194403331%7Cgb%7CACF63553.1%7C&db_idx=1&px=1&section=5&ave_thresh=33&_ignoreionsscorebelow=0&report=0&_sigthreshold=0.05&_msresflags=1089&_msresflags2=2&percolate=-1&percolate_rt=0&_minpeplen=5&sessionID=all_secdisabledsession) |
|  | [2936](http://10.200.41.11/mascot/cgi/peptide_view.pl?file=..%2Fdata%2F20160418%2FF080011.dat&query=2936&hit=1&index=gi%7C194403331%7Cgb%7CACF63553.1%7C&db_idx=1&px=1&section=5&ave_thresh=33&_ignoreionsscorebelow=0&report=0&_sigthreshold=0.05&_msresflags=1089&_msresflags2=2&percolate=-1&percolate_rt=0&_minpeplen=5&sessionID=all_secdisabledsession) | **471.9441** | **1412.8104** | **1412.7987** | **8.29** | **0** | **(29)** | **0.15** | **1** | **U** | **R.TSPLKPVSTVQTR.E** [2935](http://10.200.41.11/mascot/cgi/peptide_view.pl?file=..%2Fdata%2F20160418%2FF080011.dat&query=2935&hit=1&index=gi%7C194403331%7Cgb%7CACF63553.1%7C&db_idx=1&px=1&section=5&ave_thresh=33&_ignoreionsscorebelow=0&report=0&_sigthreshold=0.05&_msresflags=1089&_msresflags2=2&percolate=-1&percolate_rt=0&_minpeplen=5&sessionID=all_secdisabledsession) |
|  | [3121](http://10.200.41.11/mascot/cgi/peptide_view.pl?file=..%2Fdata%2F20160418%2FF080011.dat&query=3121&hit=1&index=gi%7C194403331%7Cgb%7CACF63553.1%7C&db_idx=1&px=1&section=5&ave_thresh=33&_ignoreionsscorebelow=0&report=0&_sigthreshold=0.05&_msresflags=1089&_msresflags2=2&percolate=-1&percolate_rt=0&_minpeplen=5&sessionID=all_secdisabledsession) | **761.3599** | **1520.7052** | **1520.6963** | **5.83** | **0** | **58** | **0.00016** | **1** | **U** | **K.LMQPGVSDINMER.V** [3122](http://10.200.41.11/mascot/cgi/peptide_view.pl?file=..%2Fdata%2F20160418%2FF080011.dat&query=3122&hit=1&index=gi%7C194403331%7Cgb%7CACF63553.1%7C&db_idx=1&px=1&section=5&ave_thresh=33&_ignoreionsscorebelow=0&report=0&_sigthreshold=0.05&_msresflags=1089&_msresflags2=2&percolate=-1&percolate_rt=0&_minpeplen=5&sessionID=all_secdisabledsession) |
|  | [3194](http://10.200.41.11/mascot/cgi/peptide_view.pl?file=..%2Fdata%2F20160418%2FF080011.dat&query=3194&hit=1&index=gi%7C194403331%7Cgb%7CACF63553.1%7C&db_idx=1&px=1&section=5&ave_thresh=33&_ignoreionsscorebelow=0&report=0&_sigthreshold=0.05&_msresflags=1089&_msresflags2=2&percolate=-1&percolate_rt=0&_minpeplen=5&sessionID=all_secdisabledsession) | **804.9117** | **1607.8089** | **1607.8002** | **5.41** | **0** | **98** | **2.6e-008** | **1** | **U** | **K.TSAATSASVTLSDAQAK.L** [3195](http://10.200.41.11/mascot/cgi/peptide_view.pl?file=..%2Fdata%2F20160418%2FF080011.dat&query=3195&hit=1&index=gi%7C194403331%7Cgb%7CACF63553.1%7C&db_idx=1&px=1&section=5&ave_thresh=33&_ignoreionsscorebelow=0&report=0&_sigthreshold=0.05&_msresflags=1089&_msresflags2=2&percolate=-1&percolate_rt=0&_minpeplen=5&sessionID=all_secdisabledsession) [3196](http://10.200.41.11/mascot/cgi/peptide_view.pl?file=..%2Fdata%2F20160418%2FF080011.dat&query=3196&hit=1&index=gi%7C194403331%7Cgb%7CACF63553.1%7C&db_idx=1&px=1&section=5&ave_thresh=33&_ignoreionsscorebelow=0&report=0&_sigthreshold=0.05&_msresflags=1089&_msresflags2=2&percolate=-1&percolate_rt=0&_minpeplen=5&sessionID=all_secdisabledsession) |

### Protein sequence coverage: 44%

Matched peptides shown in ***bold red***.

| **1** | MSIDR**TSPLK** | **PVSTVQTR**ET | SDTPVQKTRQ | EK**TSAATSAS** | **VTLSDAQAKL** |
| --- | --- | --- | --- | --- | --- |
| **51** | **MQPGVSDINM** | **ER**VEALKTAI | RNGELKMDTG | KIADSLIREA | QSYLQSK |

| **5.** | [gi|194402702|gb|ACF62924.1|](http://10.200.41.11/mascot/cgi/protein_view.pl?file=..%2Fdata%2F20160418%2FF080011.dat&hit=gi%7C194402702%7Cgb%7CACF62924.1%7C&db_idx=1&px=1&ave_thresh=33&_ignoreionsscorebelow=0&report=0&_sigthreshold=0.05&_msresflags=1089&_msresflags2=2&percolate=-1&percolate_rt=0&_minpeplen=5&sessionID=all_secdisabledsession)    **Mass:** 72333    **Score:** 173    **Matches:** 8(6)  **Sequences:** 3(2)  **emPAI:** 0.16 |
| --- | --- |
|  | cell invasion protein SipA [Salmonella enterica subsp. enterica serovar Newport str. SL254] |

|  | **Query** | **Observed** | **Mr(expt)** | **Mr(calc)** | **ppm** | **Miss** | **Score** | **Expect** | **Rank** | **Unique** | **Peptide** |
| --- | --- | --- | --- | --- | --- | --- | --- | --- | --- | --- | --- |
|  | [1254](http://10.200.41.11/mascot/cgi/peptide_view.pl?file=..%2Fdata%2F20160418%2FF080011.dat&query=1254&hit=1&index=gi%7C194402702%7Cgb%7CACF62924.1%7C&db_idx=1&px=1&section=5&ave_thresh=33&_ignoreionsscorebelow=0&report=0&_sigthreshold=0.05&_msresflags=1089&_msresflags2=2&percolate=-1&percolate_rt=0&_minpeplen=5&sessionID=all_secdisabledsession) | **490.7471** | **979.4796** | **979.4723** | **7.50** | **0** | **40** | **0.019** | **1** | **U** | **R.TFIDNSQR.N** [1255](http://10.200.41.11/mascot/cgi/peptide_view.pl?file=..%2Fdata%2F20160418%2FF080011.dat&query=1255&hit=1&index=gi%7C194402702%7Cgb%7CACF62924.1%7C&db_idx=1&px=1&section=5&ave_thresh=33&_ignoreionsscorebelow=0&report=0&_sigthreshold=0.05&_msresflags=1089&_msresflags2=2&percolate=-1&percolate_rt=0&_minpeplen=5&sessionID=all_secdisabledsession) [1256](http://10.200.41.11/mascot/cgi/peptide_view.pl?file=..%2Fdata%2F20160418%2FF080011.dat&query=1256&hit=1&index=gi%7C194402702%7Cgb%7CACF62924.1%7C&db_idx=1&px=1&section=5&ave_thresh=33&_ignoreionsscorebelow=0&report=0&_sigthreshold=0.05&_msresflags=1089&_msresflags2=2&percolate=-1&percolate_rt=0&_minpeplen=5&sessionID=all_secdisabledsession) [1257](http://10.200.41.11/mascot/cgi/peptide_view.pl?file=..%2Fdata%2F20160418%2FF080011.dat&query=1257&hit=1&index=gi%7C194402702%7Cgb%7CACF62924.1%7C&db_idx=1&px=1&section=5&ave_thresh=33&_ignoreionsscorebelow=0&report=0&_sigthreshold=0.05&_msresflags=1089&_msresflags2=2&percolate=-1&percolate_rt=0&_minpeplen=5&sessionID=all_secdisabledsession) |
|  | [2182](http://10.200.41.11/mascot/cgi/peptide_view.pl?file=..%2Fdata%2F20160418%2FF080011.dat&query=2182&hit=1&index=gi%7C194402702%7Cgb%7CACF62924.1%7C&db_idx=1&px=1&section=5&ave_thresh=33&_ignoreionsscorebelow=0&report=0&_sigthreshold=0.05&_msresflags=1089&_msresflags2=2&percolate=-1&percolate_rt=0&_minpeplen=5&sessionID=all_secdisabledsession) | **588.3085** | **1174.6025** | **1174.5942** | **7.06** | **0** | **59** | **0.00027** | **1** | **U** | **K.LTQEQGTSVGR.E** [2183](http://10.200.41.11/mascot/cgi/peptide_view.pl?file=..%2Fdata%2F20160418%2FF080011.dat&query=2183&hit=1&index=gi%7C194402702%7Cgb%7CACF62924.1%7C&db_idx=1&px=1&section=5&ave_thresh=33&_ignoreionsscorebelow=0&report=0&_sigthreshold=0.05&_msresflags=1089&_msresflags2=2&percolate=-1&percolate_rt=0&_minpeplen=5&sessionID=all_secdisabledsession) [2184](http://10.200.41.11/mascot/cgi/peptide_view.pl?file=..%2Fdata%2F20160418%2FF080011.dat&query=2184&hit=1&index=gi%7C194402702%7Cgb%7CACF62924.1%7C&db_idx=1&px=1&section=5&ave_thresh=33&_ignoreionsscorebelow=0&report=0&_sigthreshold=0.05&_msresflags=1089&_msresflags2=2&percolate=-1&percolate_rt=0&_minpeplen=5&sessionID=all_secdisabledsession) |
|  | [3236](http://10.200.41.11/mascot/cgi/peptide_view.pl?file=..%2Fdata%2F20160418%2FF080011.dat&query=3236&hit=1&index=gi%7C194402702%7Cgb%7CACF62924.1%7C&db_idx=1&px=1&section=5&ave_thresh=33&_ignoreionsscorebelow=0&report=0&_sigthreshold=0.05&_msresflags=1089&_msresflags2=2&percolate=-1&percolate_rt=0&_minpeplen=5&sessionID=all_secdisabledsession) | **648.8174** | **2591.2404** | **2591.2212** | **7.42** | **0** | **26** | **0.46** | **1** | **U** | **R.VDSTTHQTETAHSASTGAIDHGIAGK.I** |

### Protein sequence coverage: 6%

Matched peptides shown in ***bold red***.

| **1** | MQTEIKTQAT | NLAANLSAVR | ESATTTLSGE | IKGPQLEDFP | ALIKQASLDA |
| --- | --- | --- | --- | --- | --- |
| **51** | LFKCGKDAEA | LKEVFTNSNN | VAGKKAIMEF | AGLFRSALNA | TSDSPEAKTL |
| **101** | LMKVGAEYTA | QIIKDGLKEK | SAFGPWLPET | KKAEAKLENL | EKQLLDIIKN |
| **151** | NTGGELSKLS | TNLVMQEVMP | YIASCIEHNF | GCTLDPLTRS | NLTHLVDKAA |
| **201** | AKAVEALDMC | HQK**LTQEQGT** | **SVGR**EARHLE | MQTLIPLLLR | NVFAQIPADK |
| **251** | LPDPKIPEPA | AGPVPDGGKK | AEPTGININI | NIDSSNHSVD | NSKHINNSRS |
| **301** | HVDNSQRHID | NSNHDNSRKT | IDNSR**TFIDN** | **SQR**NGESHHS | TNSSNVSHSH |
| **351** | SR**VDSTTHQT** | **ETAHSASTGA** | **IDHGIAGK**ID | VTAHATAEAV | TNASSESKDG |
| **401** | KVVTSEKGTT | GETTSFDEVD | GVTSKSIIGK | PVQATVHGVD | DNKQQSQTAE |
| **451** | IVNVKPLASQ | LAGVENVKTD | TLQSDTTVIT | GNKAGTTDND | NSQTDKTGPF |
| **501** | SGLKFKQNSF | LSTVPSVTNM | HSMHFDARET | FLGVIRKALE | PDTSTPFPVR |
| **551** | RAFDGLRAEI | LPNDTIKSAA | LKAQCSDIDK | HPELKAKMET | LKEVITHHPQ |
| **601** | KEKLAEIALQ | FAREAGLTRL | KGETDYVLSN | VLDGLIGDGS | WRAGPAYESY |
| **651** | LNKPGVDRVI | TTVDGLHMQR |  |  |  |

| **8.** | [gi|194404219|gb|ACF64441.1|](http://10.200.41.11/mascot/cgi/protein_view.pl?file=..%2Fdata%2F20160418%2FF080011.dat&hit=gi%7C194404219%7Cgb%7CACF64441.1%7C&db_idx=1&px=1&ave_thresh=33&_ignoreionsscorebelow=0&report=0&_sigthreshold=0.05&_msresflags=1089&_msresflags2=2&percolate=-1&percolate_rt=0&_minpeplen=5&sessionID=all_secdisabledsession)    **Mass:** 34155    **Score:** 112    **Matches:** 5(5)  **Sequences:** 2(2)  **emPAI:** 0.37 |
| --- | --- |
|  | flagellar hook-associated protein 3 [Salmonella enterica subsp. enterica serovar Newport str. SL254] |

|  | **Query** | **Observed** | **Mr(expt)** | **Mr(calc)** | **ppm** | **Miss** | **Score** | **Expect** | **Rank** | **Unique** | **Peptide** |
| --- | --- | --- | --- | --- | --- | --- | --- | --- | --- | --- | --- |
|  | [993](http://10.200.41.11/mascot/cgi/peptide_view.pl?file=..%2Fdata%2F20160418%2FF080011.dat&query=993&hit=1&index=gi%7C194404219%7Cgb%7CACF64441.1%7C&db_idx=1&px=1&section=5&ave_thresh=33&_ignoreionsscorebelow=0&report=0&_sigthreshold=0.05&_msresflags=1089&_msresflags2=2&percolate=-1&percolate_rt=0&_minpeplen=5&sessionID=all_secdisabledsession) | **475.7378** | **949.4611** | **949.4539** | **7.61** | **0** | **45** | **0.0053** | **1** | **U** | **K.LGEQMSTGK.R** [994](http://10.200.41.11/mascot/cgi/peptide_view.pl?file=..%2Fdata%2F20160418%2FF080011.dat&query=994&hit=1&index=gi%7C194404219%7Cgb%7CACF64441.1%7C&db_idx=1&px=1&section=5&ave_thresh=33&_ignoreionsscorebelow=0&report=0&_sigthreshold=0.05&_msresflags=1089&_msresflags2=2&percolate=-1&percolate_rt=0&_minpeplen=5&sessionID=all_secdisabledsession) |
|  | [1796](http://10.200.41.11/mascot/cgi/peptide_view.pl?file=..%2Fdata%2F20160418%2FF080011.dat&query=1796&hit=1&index=gi%7C194404219%7Cgb%7CACF64441.1%7C&db_idx=1&px=1&section=5&ave_thresh=33&_ignoreionsscorebelow=0&report=0&_sigthreshold=0.05&_msresflags=1089&_msresflags2=2&percolate=-1&percolate_rt=0&_minpeplen=5&sessionID=all_secdisabledsession) | **545.7818** | **1089.5490** | **1089.5415** | **6.96** | **0** | **42** | **0.01** | **1** | **U** | **K.SVTQQVDSAR.T** [1797](http://10.200.41.11/mascot/cgi/peptide_view.pl?file=..%2Fdata%2F20160418%2FF080011.dat&query=1797&hit=1&index=gi%7C194404219%7Cgb%7CACF64441.1%7C&db_idx=1&px=1&section=5&ave_thresh=33&_ignoreionsscorebelow=0&report=0&_sigthreshold=0.05&_msresflags=1089&_msresflags2=2&percolate=-1&percolate_rt=0&_minpeplen=5&sessionID=all_secdisabledsession) [1798](http://10.200.41.11/mascot/cgi/peptide_view.pl?file=..%2Fdata%2F20160418%2FF080011.dat&query=1798&hit=1&index=gi%7C194404219%7Cgb%7CACF64441.1%7C&db_idx=1&px=1&section=5&ave_thresh=33&_ignoreionsscorebelow=0&report=0&_sigthreshold=0.05&_msresflags=1089&_msresflags2=2&percolate=-1&percolate_rt=0&_minpeplen=5&sessionID=all_secdisabledsession) |

### Protein sequence coverage: 5%

Matched peptides shown in ***bold red***.

| **1** | MRISTQMMYE | QNMSGITNSQ | AEWMK**LGEQM** | **STGK**RVTNPS | DDPIAASQAV |
| --- | --- | --- | --- | --- | --- |
| **51** | VLSQAQAQNS | QYALARTFAT | QKVSLEESVL | SQVTTAIQTA | QEKIVYAGNG |
| **101** | TLSDDDRASL | ATDLQGIRDQ | LMNLANSTDG | NGRYIFAGYK | TEAAPFDQAT |
| **151** | GGYHGGEK**SV** | **TQQVDSAR**TM | VIGHTGAQIF | NSITSNAVPE | PDGSDSEKNL |
| **201** | FVMLDTAIAA | LKTPVEGNDV | EKEKAAAAID | KTNRGLKNSL | NNVLTVRAEL |
| **251** | GTQLSELSTL | DSLGSDRALG | QKLQMSNLVD | VDWNSVISSY | VMQQAALQAS |
| **301** | YKTFTDMQGM | SLFQLNR |  |  |  |

| **9.** | [gi|392616944|gb|EIW99372.1|](http://10.200.41.11/mascot/cgi/protein_view.pl?file=..%2Fdata%2F20160418%2FF080011.dat&hit=gi%7C392616944%7Cgb%7CEIW99372.1%7C&db_idx=1&px=1&ave_thresh=33&_ignoreionsscorebelow=0&report=0&_sigthreshold=0.05&_msresflags=1089&_msresflags2=2&percolate=-1&percolate_rt=0&_minpeplen=5&sessionID=all_secdisabledsession)    **Mass:** 37081    **Score:** 111    **Matches:** 2(2)  **Sequences:** 1(1)  **emPAI:** 0.16 |
| --- | --- |
|  | cell invasion protein SipD [Salmonella enterica subsp. enterica serovar Newport str. Levine 15] |

|  | **Query** | **Observed** | **Mr(expt)** | **Mr(calc)** | **ppm** | **Miss** | **Score** | **Expect** | **Rank** | **Unique** | **Peptide** |
| --- | --- | --- | --- | --- | --- | --- | --- | --- | --- | --- | --- |
|  | [3092](http://10.200.41.11/mascot/cgi/peptide_view.pl?file=..%2Fdata%2F20160418%2FF080011.dat&query=3092&hit=1&index=gi%7C392616944%7Cgb%7CEIW99372.1%7C&db_idx=1&px=1&section=5&ave_thresh=33&_ignoreionsscorebelow=0&report=0&_sigthreshold=0.05&_msresflags=1089&_msresflags2=2&percolate=-1&percolate_rt=0&_minpeplen=5&sessionID=all_secdisabledsession) | **752.3784** | **1502.7422** | **1502.7325** | **6.45** | **0** | **64** | **5.7e-005** | **1** | **U** | **K.SGVSLSAEQNENLR.S** [3093](http://10.200.41.11/mascot/cgi/peptide_view.pl?file=..%2Fdata%2F20160418%2FF080011.dat&query=3093&hit=1&index=gi%7C392616944%7Cgb%7CEIW99372.1%7C&db_idx=1&px=1&section=5&ave_thresh=33&_ignoreionsscorebelow=0&report=0&_sigthreshold=0.05&_msresflags=1089&_msresflags2=2&percolate=-1&percolate_rt=0&_minpeplen=5&sessionID=all_secdisabledsession) |

### Protein sequence coverage: 4%

Matched peptides shown in ***bold red***.

| **1** | MLNIQNYSAS | PHPGIVAERP | QTPSASEHVE | TAVVPSTTEH | RGTDIISLSQ |
| --- | --- | --- | --- | --- | --- |
| **51** | AATKIQQAQQ | TLQSTPPISE | ENNDERTLAR | QQLTSSLNAL | AK**SGVSLSAE** |
| **101** | **QNENLR**SAFS | APTSALFSAS | PMAQPRTTIS | DAEIWDMVSQ | NISAIGDSYL |
| **151** | GVYENVVAVY | TDFYQAFSDI | LSKMGGWLLP | GKDGNTVKLD | VTSLKNDLNS |
| **201** | LVNKYNQINS | NTVLFPAQSG | SGVKVATEAE | ARQWLSELNL | PNSCLKSYGS |
| **251** | GYVVTVDLTP | LQKMVQDIDG | LGAPGKDSKL | EMDNAKYQAW | QSGFKAQEEN |
| **301** | MKTTLQTLTQ | KYSNANSLYD | NLVKVLSSTI | SSSLETAKSF | LQG |

| **13.** | [gi|194403640|gb|ACF63862.1|](http://10.200.41.11/mascot/cgi/protein_view.pl?file=..%2Fdata%2F20160418%2FF080011.dat&hit=gi%7C194403640%7Cgb%7CACF63862.1%7C&db_idx=1&px=1&ave_thresh=33&_ignoreionsscorebelow=0&report=0&_sigthreshold=0.05&_msresflags=1089&_msresflags2=2&percolate=-1&percolate_rt=0&_minpeplen=5&sessionID=all_secdisabledsession)    **Mass:** 62382    **Score:** 55     **Matches:** 5(1)  **Sequences:** 3(1)  **emPAI:** 0.09 |
| --- | --- |
|  | cell invasion protein SipB [Salmonella enterica subsp. enterica serovar Newport str. SL254] |

|  | **Query** | **Observed** | **Mr(expt)** | **Mr(calc)** | **ppm** | **Miss** | **Score** | **Expect** | **Rank** | **Unique** | **Peptide** |
| --- | --- | --- | --- | --- | --- | --- | --- | --- | --- | --- | --- |
|  | [273](http://10.200.41.11/mascot/cgi/peptide_view.pl?file=..%2Fdata%2F20160418%2FF080011.dat&query=273&hit=5&index=gi%7C194403640%7Cgb%7CACF63862.1%7C&db_idx=1&px=1&section=5&ave_thresh=33&_ignoreionsscorebelow=0&report=0&_sigthreshold=0.05&_msresflags=1089&_msresflags2=2&percolate=-1&percolate_rt=0&_minpeplen=5&sessionID=all_secdisabledsession) | **423.2306** | **844.4466** | **844.4403** | **7.47** | **0** | **2** | **1.2e+002** | **5** | **U** | **K.QLAQNGSK.L** |
|  | [1257](http://10.200.41.11/mascot/cgi/peptide_view.pl?file=..%2Fdata%2F20160418%2FF080011.dat&query=1257&hit=6&index=gi%7C194403640%7Cgb%7CACF63862.1%7C&db_idx=1&px=1&section=5&ave_thresh=33&_ignoreionsscorebelow=0&report=0&_sigthreshold=0.05&_msresflags=1089&_msresflags2=2&percolate=-1&percolate_rt=0&_minpeplen=5&sessionID=all_secdisabledsession) | 490.7472 | 979.4798 | 979.4909 | -11.34 | 0 | 3 | 82 | 6 | U | K.LFTQGMQR.I [1254](http://10.200.41.11/mascot/cgi/peptide_view.pl?file=..%2Fdata%2F20160418%2FF080011.dat&query=1254&hit=5&index=gi%7C194403640%7Cgb%7CACF63862.1%7C&db_idx=1&px=1&section=5&ave_thresh=33&_ignoreionsscorebelow=0&report=0&_sigthreshold=0.05&_msresflags=1089&_msresflags2=2&percolate=-1&percolate_rt=0&_minpeplen=5&sessionID=all_secdisabledsession) [1256](http://10.200.41.11/mascot/cgi/peptide_view.pl?file=..%2Fdata%2F20160418%2FF080011.dat&query=1256&hit=7&index=gi%7C194403640%7Cgb%7CACF63862.1%7C&db_idx=1&px=1&section=5&ave_thresh=33&_ignoreionsscorebelow=0&report=0&_sigthreshold=0.05&_msresflags=1089&_msresflags2=2&percolate=-1&percolate_rt=0&_minpeplen=5&sessionID=all_secdisabledsession) |
|  | [1728](http://10.200.41.11/mascot/cgi/peptide_view.pl?file=..%2Fdata%2F20160418%2FF080011.dat&query=1728&hit=1&index=gi%7C194403640%7Cgb%7CACF63862.1%7C&db_idx=1&px=1&section=5&ave_thresh=33&_ignoreionsscorebelow=0&report=0&_sigthreshold=0.05&_msresflags=1089&_msresflags2=2&percolate=-1&percolate_rt=0&_minpeplen=5&sessionID=all_secdisabledsession) | **531.7864** | **1061.5583** | **1061.5505** | **7.34** | **0** | **55** | **0.0006** | **1** | **U** | **R.LAEAAFEGVR.K** |

### Protein sequence coverage: 4%

Matched peptides shown in ***bold red***.

| **1** | MVNDASSISR | SGYTQNPR**LA** | **EAAFEGVR**KN | TDFLKAADKA | FKDVVATKAG |
| --- | --- | --- | --- | --- | --- |
| **51** | DLKAGTKSGE | SAINTVGLKP | PTDAAREKLS | SEGQLTLLLG | KLMTLLGDVS |
| **101** | LSQLESRLAV | WQAMIESQKE | MGIQVSKEFQ | TALGEAQEAT | DLYEASIKKT |
| **151** | DTAKSVYDAA | AKKLTQAQNK | LQSLDPADPG | YAQAEAAVEQ | AGKEATEAKE |
| **201** | ALDKATDATV | KAGTDAKAKA | EKADNILTKF | QGTANAASQN | QVSQGEQDNL |
| **251** | SNVARLTMLM | AMFIEIVGKN | TEESLQNDLA | LFNALQEGRQ | AEMEKKSAEF |
| **301** | QEETRKAEET | NRIMGCIGKV | LGALLTIVSV | VAAVFTGGAS | LALAAVGLAV |
| **351** | MVADEIVKAA | TGVSFIQQAL | NPIMEHVLKP | LMELIGKAIT | KALEGLGVDK |
| **401** | KTAEMAGSIV | GAIVAAIAMV | AVIVVVAVVG | KGAAAKLGNA | LSKMMGETIK |
| **451** | KLVPNVLK**QL** | **AQNGSKLFTQ** | **GMQR**ITSGLG | NVGSKMGLQT | NALSKELVGN |
| **501** | TLNKVALGME | VTNTAAQSAG | GVAEGVFIKN | ASEALADFML | ARFAMDQIQQ |
| **551** | WLKQSVEIFG | ENQKVTAELQ | KAMSSAVQQN | ADASRFILRQ | SRA |

*******************************************************************************************************************************************************

**2^nd^ technical replicate**

**Enzyme : Trypsin**

**Variable modifications :** [**Oxidation (M)**](http://10.200.41.11/mascot/cgi/client.pl?modification&mod_name=Oxidation%20%28M%29&file=..%2Fdata%2F20160418%2FF080015.dat)

**Mass values : Monoisotopic**

**Protein Mass : Unrestricted**

**Peptide Mass Tolerance : ± 20 ppm**

**Fragment Mass Tolerance: ± 0.4 Da**

**Max Missed Cleavages : 3**

**Instrument type : Default**

**Number of queries : 3244**

| **Protein hits           :** | [**gi|50830890|gb|AAT81610.1|**](http://10.200.41.11/mascot/cgi/master_results.pl?file=..%2Fdata%2F20160418%2FF080015.dat#Hit1) | phase 1 flagellin [Salmonella enterica subsp. enterica serovar Newport] |
| --- | --- | --- |
|  | [**gi|392616945|gb|EIW99373.1|**](http://10.200.41.11/mascot/cgi/master_results.pl?file=..%2Fdata%2F20160418%2FF080015.dat#Hit2) | pathogenicity island 1 effector protein SipC [Salmonella enterica subsp. enterica serovar Newport str. Levine 15] |
|  | [**gi|194401698|gb|ACF61920.1|**](http://10.200.41.11/mascot/cgi/master_results.pl?file=..%2Fdata%2F20160418%2FF080015.dat#Hit3) | translation elongation factor Tu [Salmonella enterica subsp. enterica serovar Newport str. SL254] |
|  | [**gi|194402702|gb|ACF62924.1|**](http://10.200.41.11/mascot/cgi/master_results.pl?file=..%2Fdata%2F20160418%2FF080015.dat#Hit4) | cell invasion protein SipA [Salmonella enterica subsp. enterica serovar Newport str. SL254] |
|  | [**gi|194403331|gb|ACF63553.1|**](http://10.200.41.11/mascot/cgi/master_results.pl?file=..%2Fdata%2F20160418%2FF080015.dat#Hit5) | negative regulator of flagellin synthesis [Salmonella enterica subsp. enterica serovar Newport str. SL254] |
|  | [**gi|194404381|gb|ACF64603.1|**](http://10.200.41.11/mascot/cgi/master_results.pl?file=..%2Fdata%2F20160418%2FF080015.dat#Hit6) | DNA-binding protein HU-alpha [Salmonella enterica subsp. enterica serovar Newport str. SL254] |
|  | [**gi|874404664|gb|KMU13862.1|**](http://10.200.41.11/mascot/cgi/master_results.pl?file=..%2Fdata%2F20160418%2FF080015.dat#Hit7) | flagellin [Salmonella enterica subsp. enterica serovar Newport str. DC_10-446] |
|  | [**gi|194401103|gb|ACF61325.1|**](http://10.200.41.11/mascot/cgi/master_results.pl?file=..%2Fdata%2F20160418%2FF080015.dat#Hit8) | ribosomal protein L6 [Salmonella enterica subsp. enterica serovar Newport str. SL254] |
|  | [**gi|194403640|gb|ACF63862.1|**](http://10.200.41.11/mascot/cgi/master_results.pl?file=..%2Fdata%2F20160418%2FF080015.dat#Hit9) | cell invasion protein SipB [Salmonella enterica subsp. enterica serovar Newport str. SL254] |
|  | [**gi|194401878|gb|ACF62100.1|**](http://10.200.41.11/mascot/cgi/master_results.pl?file=..%2Fdata%2F20160418%2FF080015.dat#Hit10) | ribosomal protein L11 [Salmonella enterica subsp. enterica serovar Newport str. SL254] |
|  | [**gi|194401176|gb|ACF61398.1|**](http://10.200.41.11/mascot/cgi/master_results.pl?file=..%2Fdata%2F20160418%2FF080015.dat#Hit11) | ribosomal protein L7/L12 [Salmonella enterica subsp. enterica serovar Newport str. SL254] |
|  | [**gi|194402309|gb|ACF62531.1|**](http://10.200.41.11/mascot/cgi/master_results.pl?file=..%2Fdata%2F20160418%2FF080015.dat#Hit12) | ribosomal protein S10 [Salmonella enterica subsp. enterica serovar Newport str. SL254] |
|  | [**gi|194403829|gb|ACF64051.1|**](http://10.200.41.11/mascot/cgi/master_results.pl?file=..%2Fdata%2F20160418%2FF080015.dat#Hit13) | ribosomal protein L3 [Salmonella enterica subsp. enterica serovar Newport str. SL254] |
|  | [**gi|194404219|gb|ACF64441.1|**](http://10.200.41.11/mascot/cgi/master_results.pl?file=..%2Fdata%2F20160418%2FF080015.dat#Hit14) | flagellar hook-associated protein 3 [Salmonella enterica subsp. enterica serovar Newport str. SL254] |
|  | [**gi|194405415|gb|ACF65637.1|**](http://10.200.41.11/mascot/cgi/master_results.pl?file=..%2Fdata%2F20160418%2FF080015.dat#Hit15) | ribosomal protein L15 [Salmonella enterica subsp. enterica serovar Newport str. SL254] |
|  | [**gi|392807267|gb|EJA63344.1|**](http://10.200.41.11/mascot/cgi/master_results.pl?file=..%2Fdata%2F20160418%2FF080015.dat#Hit16) | hypothetical protein SEEN443_08957 [Salmonella enterica subsp. enterica serovar Newport str. CVM 19443] |
|  | [**gi|194402357|gb|ACF62579.1|**](http://10.200.41.11/mascot/cgi/master_results.pl?file=..%2Fdata%2F20160418%2FF080015.dat#Hit17) | ribosomal protein S15 [Salmonella enterica subsp. enterica serovar Newport str. SL254] |
|  | [**gi|194402029|gb|ACF62251.1|**](http://10.200.41.11/mascot/cgi/master_results.pl?file=..%2Fdata%2F20160418%2FF080015.dat#Hit18) | lipase 1 [Salmonella enterica subsp. enterica serovar Newport str. SL254] |

| **1.** | [gi|50830890|gb|AAT81610.1|](http://10.200.41.11/mascot/cgi/protein_view.pl?file=..%2Fdata%2F20160418%2FF080015.dat&hit=gi%7C50830890%7Cgb%7CAAT81610.1%7C&db_idx=1&px=1&ave_thresh=32&_ignoreionsscorebelow=0&report=0&_sigthreshold=0.05&_msresflags=1089&_msresflags2=2&percolate=-1&percolate_rt=0&_minpeplen=5&sessionID=all_secdisabledsession)    **Mass:** 52223    **Score:** 613    **Matches:** 31(14)  **Sequences:** 9(5)  **emPAI:** 0.77 |
| --- | --- |
|  | phase 1 flagellin [Salmonella enterica subsp. enterica serovar Newport] |

|  | **Query** | **Observed** | **Mr(expt)** | **Mr(calc)** | **ppm** | **Miss** | **Score** | **Expect** | **Rank** | **Unique** | **Peptide** |
| --- | --- | --- | --- | --- | --- | --- | --- | --- | --- | --- | --- |
|  | [935](http://10.200.41.11/mascot/cgi/peptide_view.pl?file=..%2Fdata%2F20160418%2FF080015.dat&query=935&hit=1&index=gi%7C50830890%7Cgb%7CAAT81610.1%7C&db_idx=1&px=1&section=5&ave_thresh=32&_ignoreionsscorebelow=0&report=0&_sigthreshold=0.05&_msresflags=1089&_msresflags2=2&percolate=-1&percolate_rt=0&_minpeplen=5&sessionID=all_secdisabledsession) | **481.2675** | **960.5205** | **960.5128** | **8.08** | **0** | **34** | **0.082** | **1** |  | **K.TEVVTIDGK.T** [933](http://10.200.41.11/mascot/cgi/peptide_view.pl?file=..%2Fdata%2F20160418%2FF080015.dat&query=933&hit=1&index=gi%7C50830890%7Cgb%7CAAT81610.1%7C&db_idx=1&px=1&section=5&ave_thresh=32&_ignoreionsscorebelow=0&report=0&_sigthreshold=0.05&_msresflags=1089&_msresflags2=2&percolate=-1&percolate_rt=0&_minpeplen=5&sessionID=all_secdisabledsession) [934](http://10.200.41.11/mascot/cgi/peptide_view.pl?file=..%2Fdata%2F20160418%2FF080015.dat&query=934&hit=1&index=gi%7C50830890%7Cgb%7CAAT81610.1%7C&db_idx=1&px=1&section=5&ave_thresh=32&_ignoreionsscorebelow=0&report=0&_sigthreshold=0.05&_msresflags=1089&_msresflags2=2&percolate=-1&percolate_rt=0&_minpeplen=5&sessionID=all_secdisabledsession) |
|  | [1655](http://10.200.41.11/mascot/cgi/peptide_view.pl?file=..%2Fdata%2F20160418%2FF080015.dat&query=1655&hit=1&index=gi%7C50830890%7Cgb%7CAAT81610.1%7C&db_idx=1&px=1&section=5&ave_thresh=32&_ignoreionsscorebelow=0&report=0&_sigthreshold=0.05&_msresflags=1089&_msresflags2=2&percolate=-1&percolate_rt=0&_minpeplen=5&sessionID=all_secdisabledsession) | **549.7971** | **1097.5797** | **1097.5716** | **7.33** | **0** | **2** | **85** | **1** |  | **K.AQPELAEAAAK.T** |
|  | [1666](http://10.200.41.11/mascot/cgi/peptide_view.pl?file=..%2Fdata%2F20160418%2FF080015.dat&query=1666&hit=1&index=gi%7C50830890%7Cgb%7CAAT81610.1%7C&db_idx=1&px=1&section=5&ave_thresh=32&_ignoreionsscorebelow=0&report=0&_sigthreshold=0.05&_msresflags=1089&_msresflags2=2&percolate=-1&percolate_rt=0&_minpeplen=5&sessionID=all_secdisabledsession) | **551.2723** | **1100.5301** | **1100.5210** | **8.27** | **0** | **68** | **1.4e-005** | **1** | **U** | **K.DDAAGQAIANR.F** [1665](http://10.200.41.11/mascot/cgi/peptide_view.pl?file=..%2Fdata%2F20160418%2FF080015.dat&query=1665&hit=1&index=gi%7C50830890%7Cgb%7CAAT81610.1%7C&db_idx=1&px=1&section=5&ave_thresh=32&_ignoreionsscorebelow=0&report=0&_sigthreshold=0.05&_msresflags=1089&_msresflags2=2&percolate=-1&percolate_rt=0&_minpeplen=5&sessionID=all_secdisabledsession) [1667](http://10.200.41.11/mascot/cgi/peptide_view.pl?file=..%2Fdata%2F20160418%2FF080015.dat&query=1667&hit=1&index=gi%7C50830890%7Cgb%7CAAT81610.1%7C&db_idx=1&px=1&section=5&ave_thresh=32&_ignoreionsscorebelow=0&report=0&_sigthreshold=0.05&_msresflags=1089&_msresflags2=2&percolate=-1&percolate_rt=0&_minpeplen=5&sessionID=all_secdisabledsession) [1668](http://10.200.41.11/mascot/cgi/peptide_view.pl?file=..%2Fdata%2F20160418%2FF080015.dat&query=1668&hit=1&index=gi%7C50830890%7Cgb%7CAAT81610.1%7C&db_idx=1&px=1&section=5&ave_thresh=32&_ignoreionsscorebelow=0&report=0&_sigthreshold=0.05&_msresflags=1089&_msresflags2=2&percolate=-1&percolate_rt=0&_minpeplen=5&sessionID=all_secdisabledsession) |
|  | [1731](http://10.200.41.11/mascot/cgi/peptide_view.pl?file=..%2Fdata%2F20160418%2FF080015.dat&query=1731&hit=1&index=gi%7C50830890%7Cgb%7CAAT81610.1%7C&db_idx=1&px=1&section=5&ave_thresh=32&_ignoreionsscorebelow=0&report=0&_sigthreshold=0.05&_msresflags=1089&_msresflags2=2&percolate=-1&percolate_rt=0&_minpeplen=5&sessionID=all_secdisabledsession) | **565.7976** | **1129.5807** | **1129.5728** | **7.00** | **0** | **51** | **0.0015** | **1** |  | **K.TAANQLGGVDGK.T** |
|  | [1738](http://10.200.41.11/mascot/cgi/peptide_view.pl?file=..%2Fdata%2F20160418%2FF080015.dat&query=1738&hit=1&index=gi%7C50830890%7Cgb%7CAAT81610.1%7C&db_idx=1&px=1&section=5&ave_thresh=32&_ignoreionsscorebelow=0&report=0&_sigthreshold=0.05&_msresflags=1089&_msresflags2=2&percolate=-1&percolate_rt=0&_minpeplen=5&sessionID=all_secdisabledsession) | **566.8062** | **1131.5978** | **1131.5884** | **8.28** | **0** | **57** | **0.0004** | **1** | **U** | **K.SQSALGTAIER.L** [1733](http://10.200.41.11/mascot/cgi/peptide_view.pl?file=..%2Fdata%2F20160418%2FF080015.dat&query=1733&hit=1&index=gi%7C50830890%7Cgb%7CAAT81610.1%7C&db_idx=1&px=1&section=5&ave_thresh=32&_ignoreionsscorebelow=0&report=0&_sigthreshold=0.05&_msresflags=1089&_msresflags2=2&percolate=-1&percolate_rt=0&_minpeplen=5&sessionID=all_secdisabledsession) [1734](http://10.200.41.11/mascot/cgi/peptide_view.pl?file=..%2Fdata%2F20160418%2FF080015.dat&query=1734&hit=1&index=gi%7C50830890%7Cgb%7CAAT81610.1%7C&db_idx=1&px=1&section=5&ave_thresh=32&_ignoreionsscorebelow=0&report=0&_sigthreshold=0.05&_msresflags=1089&_msresflags2=2&percolate=-1&percolate_rt=0&_minpeplen=5&sessionID=all_secdisabledsession) [1735](http://10.200.41.11/mascot/cgi/peptide_view.pl?file=..%2Fdata%2F20160418%2FF080015.dat&query=1735&hit=1&index=gi%7C50830890%7Cgb%7CAAT81610.1%7C&db_idx=1&px=1&section=5&ave_thresh=32&_ignoreionsscorebelow=0&report=0&_sigthreshold=0.05&_msresflags=1089&_msresflags2=2&percolate=-1&percolate_rt=0&_minpeplen=5&sessionID=all_secdisabledsession) [1736](http://10.200.41.11/mascot/cgi/peptide_view.pl?file=..%2Fdata%2F20160418%2FF080015.dat&query=1736&hit=1&index=gi%7C50830890%7Cgb%7CAAT81610.1%7C&db_idx=1&px=1&section=5&ave_thresh=32&_ignoreionsscorebelow=0&report=0&_sigthreshold=0.05&_msresflags=1089&_msresflags2=2&percolate=-1&percolate_rt=0&_minpeplen=5&sessionID=all_secdisabledsession) [1737](http://10.200.41.11/mascot/cgi/peptide_view.pl?file=..%2Fdata%2F20160418%2FF080015.dat&query=1737&hit=1&index=gi%7C50830890%7Cgb%7CAAT81610.1%7C&db_idx=1&px=1&section=5&ave_thresh=32&_ignoreionsscorebelow=0&report=0&_sigthreshold=0.05&_msresflags=1089&_msresflags2=2&percolate=-1&percolate_rt=0&_minpeplen=5&sessionID=all_secdisabledsession) |
|  | [1871](http://10.200.41.11/mascot/cgi/peptide_view.pl?file=..%2Fdata%2F20160418%2FF080015.dat&query=1871&hit=1&index=gi%7C50830890%7Cgb%7CAAT81610.1%7C&db_idx=1&px=1&section=5&ave_thresh=32&_ignoreionsscorebelow=0&report=0&_sigthreshold=0.05&_msresflags=1089&_msresflags2=2&percolate=-1&percolate_rt=0&_minpeplen=5&sessionID=all_secdisabledsession) | **582.8084** | **1163.6023** | **1163.5935** | **7.53** | **0** | **62** | **0.00012** | **1** | **U** | **R.VSGQTQFNGVK.V** [1872](http://10.200.41.11/mascot/cgi/peptide_view.pl?file=..%2Fdata%2F20160418%2FF080015.dat&query=1872&hit=1&index=gi%7C50830890%7Cgb%7CAAT81610.1%7C&db_idx=1&px=1&section=5&ave_thresh=32&_ignoreionsscorebelow=0&report=0&_sigthreshold=0.05&_msresflags=1089&_msresflags2=2&percolate=-1&percolate_rt=0&_minpeplen=5&sessionID=all_secdisabledsession) |
|  | [3059](http://10.200.41.11/mascot/cgi/peptide_view.pl?file=..%2Fdata%2F20160418%2FF080015.dat&query=3059&hit=1&index=gi%7C50830890%7Cgb%7CAAT81610.1%7C&db_idx=1&px=1&section=5&ave_thresh=32&_ignoreionsscorebelow=0&report=0&_sigthreshold=0.05&_msresflags=1089&_msresflags2=2&percolate=-1&percolate_rt=0&_minpeplen=5&sessionID=all_secdisabledsession) | **538.9484** | **1613.8234** | **1613.8121** | **7.03** | **1** | **34** | **0.078** | **1** | **U** | **R.INSAKDDAAGQAIANR.F** [3058](http://10.200.41.11/mascot/cgi/peptide_view.pl?file=..%2Fdata%2F20160418%2FF080015.dat&query=3058&hit=1&index=gi%7C50830890%7Cgb%7CAAT81610.1%7C&db_idx=1&px=1&section=5&ave_thresh=32&_ignoreionsscorebelow=0&report=0&_sigthreshold=0.05&_msresflags=1089&_msresflags2=2&percolate=-1&percolate_rt=0&_minpeplen=5&sessionID=all_secdisabledsession) [3060](http://10.200.41.11/mascot/cgi/peptide_view.pl?file=..%2Fdata%2F20160418%2FF080015.dat&query=3060&hit=1&index=gi%7C50830890%7Cgb%7CAAT81610.1%7C&db_idx=1&px=1&section=5&ave_thresh=32&_ignoreionsscorebelow=0&report=0&_sigthreshold=0.05&_msresflags=1089&_msresflags2=2&percolate=-1&percolate_rt=0&_minpeplen=5&sessionID=all_secdisabledsession) [3061](http://10.200.41.11/mascot/cgi/peptide_view.pl?file=..%2Fdata%2F20160418%2FF080015.dat&query=3061&hit=1&index=gi%7C50830890%7Cgb%7CAAT81610.1%7C&db_idx=1&px=1&section=5&ave_thresh=32&_ignoreionsscorebelow=0&report=0&_sigthreshold=0.05&_msresflags=1089&_msresflags2=2&percolate=-1&percolate_rt=0&_minpeplen=5&sessionID=all_secdisabledsession) [3062](http://10.200.41.11/mascot/cgi/peptide_view.pl?file=..%2Fdata%2F20160418%2FF080015.dat&query=3062&hit=1&index=gi%7C50830890%7Cgb%7CAAT81610.1%7C&db_idx=1&px=1&section=5&ave_thresh=32&_ignoreionsscorebelow=0&report=0&_sigthreshold=0.05&_msresflags=1089&_msresflags2=2&percolate=-1&percolate_rt=0&_minpeplen=5&sessionID=all_secdisabledsession) [3063](http://10.200.41.11/mascot/cgi/peptide_view.pl?file=..%2Fdata%2F20160418%2FF080015.dat&query=3063&hit=1&index=gi%7C50830890%7Cgb%7CAAT81610.1%7C&db_idx=1&px=1&section=5&ave_thresh=32&_ignoreionsscorebelow=0&report=0&_sigthreshold=0.05&_msresflags=1089&_msresflags2=2&percolate=-1&percolate_rt=0&_minpeplen=5&sessionID=all_secdisabledsession) |
|  | [3097](http://10.200.41.11/mascot/cgi/peptide_view.pl?file=..%2Fdata%2F20160418%2FF080015.dat&query=3097&hit=1&index=gi%7C50830890%7Cgb%7CAAT81610.1%7C&db_idx=1&px=1&section=5&ave_thresh=32&_ignoreionsscorebelow=0&report=0&_sigthreshold=0.05&_msresflags=1089&_msresflags2=2&percolate=-1&percolate_rt=0&_minpeplen=5&sessionID=all_secdisabledsession) | **539.9377** | **1616.7912** | **1616.8006** | **-5.77** | **1** | **1** | **1e+002** | **1** | **U** | **K.KALEDGGVSNADATAAK.L** [3094](http://10.200.41.11/mascot/cgi/peptide_view.pl?file=..%2Fdata%2F20160418%2FF080015.dat&query=3094&hit=1&index=gi%7C50830890%7Cgb%7CAAT81610.1%7C&db_idx=1&px=1&section=5&ave_thresh=32&_ignoreionsscorebelow=0&report=0&_sigthreshold=0.05&_msresflags=1089&_msresflags2=2&percolate=-1&percolate_rt=0&_minpeplen=5&sessionID=all_secdisabledsession) [3095](http://10.200.41.11/mascot/cgi/peptide_view.pl?file=..%2Fdata%2F20160418%2FF080015.dat&query=3095&hit=1&index=gi%7C50830890%7Cgb%7CAAT81610.1%7C&db_idx=1&px=1&section=5&ave_thresh=32&_ignoreionsscorebelow=0&report=0&_sigthreshold=0.05&_msresflags=1089&_msresflags2=2&percolate=-1&percolate_rt=0&_minpeplen=5&sessionID=all_secdisabledsession) [3096](http://10.200.41.11/mascot/cgi/peptide_view.pl?file=..%2Fdata%2F20160418%2FF080015.dat&query=3096&hit=1&index=gi%7C50830890%7Cgb%7CAAT81610.1%7C&db_idx=1&px=1&section=5&ave_thresh=32&_ignoreionsscorebelow=0&report=0&_sigthreshold=0.05&_msresflags=1089&_msresflags2=2&percolate=-1&percolate_rt=0&_minpeplen=5&sessionID=all_secdisabledsession) [3098](http://10.200.41.11/mascot/cgi/peptide_view.pl?file=..%2Fdata%2F20160418%2FF080015.dat&query=3098&hit=1&index=gi%7C50830890%7Cgb%7CAAT81610.1%7C&db_idx=1&px=1&section=5&ave_thresh=32&_ignoreionsscorebelow=0&report=0&_sigthreshold=0.05&_msresflags=1089&_msresflags2=2&percolate=-1&percolate_rt=0&_minpeplen=5&sessionID=all_secdisabledsession) [3099](http://10.200.41.11/mascot/cgi/peptide_view.pl?file=..%2Fdata%2F20160418%2FF080015.dat&query=3099&hit=1&index=gi%7C50830890%7Cgb%7CAAT81610.1%7C&db_idx=1&px=1&section=5&ave_thresh=32&_ignoreionsscorebelow=0&report=0&_sigthreshold=0.05&_msresflags=1089&_msresflags2=2&percolate=-1&percolate_rt=0&_minpeplen=5&sessionID=all_secdisabledsession) [3100](http://10.200.41.11/mascot/cgi/peptide_view.pl?file=..%2Fdata%2F20160418%2FF080015.dat&query=3100&hit=1&index=gi%7C50830890%7Cgb%7CAAT81610.1%7C&db_idx=1&px=1&section=5&ave_thresh=32&_ignoreionsscorebelow=0&report=0&_sigthreshold=0.05&_msresflags=1089&_msresflags2=2&percolate=-1&percolate_rt=0&_minpeplen=5&sessionID=all_secdisabledsession) |
|  | [3216](http://10.200.41.11/mascot/cgi/peptide_view.pl?file=..%2Fdata%2F20160418%2FF080015.dat&query=3216&hit=1&index=gi%7C50830890%7Cgb%7CAAT81610.1%7C&db_idx=1&px=1&section=5&ave_thresh=32&_ignoreionsscorebelow=0&report=0&_sigthreshold=0.05&_msresflags=1089&_msresflags2=2&percolate=-1&percolate_rt=0&_minpeplen=5&sessionID=all_secdisabledsession) | **659.2984** | **1974.8734** | **1974.8589** | **7.34** | **1** | **38** | **0.0064** | **1** |  | **R.SRIEDSDYATEVSNMSR.A** |

### Protein sequence coverage: 20%

Matched peptides shown in ***bold red***.

| **1** | MAQVINTNSL | SLLTQNNLNK | **SQSALGTAIE** | **R**LSSGLR**INS** | **AKDDAAGQAI** |
| --- | --- | --- | --- | --- | --- |
| **51** | **ANR**FTANIKG | LTQASRNAND | GISIAQTTEG | ALNEINNNLQ | RVRELAVQSA |
| **101** | NSTNSQSDLD | SIQAEITQRL | NEIDR**VSGQT** | **QFNGVK**VLAQ | DNTLTIQVGA |
| **151** | NDGETIDIDL | KQINSQTLGL | DTLNVQKAYD | VSATAAMDPK | SFTDGTKNLT |
| **201** | APDATAIKAA | LGNPAATGDS | LSATLSFKDG | KYYATVAGYT | NAADTSKNGK |
| **251** | YEVNVDSATG | AVTFNAAPTK | ATVTGDTTVT | KVQVNAPVAV | STDVK**KALED** |
| **301** | **GGVSNADATA** | **AK**LVKMSYTD | KNGKSIDGGY | ALEAGGKYYA | ATYDEGTGKI |
| **351** | TANVTTYTDS | TGVTK**TAANQ** | **LGGVDGKTEV** | **VTIDGK**TYNA | SKAAGHDFK**A** |
| **401** | **QPELAEAAAK** | TTENPLAKID | AALAQVDALR | SDLGAVQNRF | NSAITNLGNT |
| **451** | VNNLSEAR**SR** | **IEDSDYATEV** | **SNMSR**AQILQ | QAGTSVLAQA | NQVPQNVLSL |
| **501** | LR |  |  |  |  |

| **2.** | [gi|392616945|gb|EIW99373.1|](http://10.200.41.11/mascot/cgi/protein_view.pl?file=..%2Fdata%2F20160418%2FF080015.dat&hit=gi%7C392616945%7Cgb%7CEIW99373.1%7C&db_idx=1&px=1&ave_thresh=32&_ignoreionsscorebelow=0&report=0&_sigthreshold=0.05&_msresflags=1089&_msresflags2=2&percolate=-1&percolate_rt=0&_minpeplen=5&sessionID=all_secdisabledsession)    **Mass:** 42957    **Score:** 587    **Matches:** 23(18)  **Sequences:** 6(6)  **emPAI:** 1.29 |
| --- | --- |
|  | pathogenicity island 1 effector protein SipC [Salmonella enterica subsp. enterica serovar Newport str. Levine 15] |

|  | **Query** | **Observed** | **Mr(expt)** | **Mr(calc)** | **ppm** | **Miss** | **Score** | **Expect** | **Rank** | **Unique** | **Peptide** |
| --- | --- | --- | --- | --- | --- | --- | --- | --- | --- | --- | --- |
|  | [1186](http://10.200.41.11/mascot/cgi/peptide_view.pl?file=..%2Fdata%2F20160418%2FF080015.dat&query=1186&hit=1&index=gi%7C392616945%7Cgb%7CEIW99373.1%7C&db_idx=1&px=1&section=5&ave_thresh=32&_ignoreionsscorebelow=0&report=0&_sigthreshold=0.05&_msresflags=1089&_msresflags2=2&percolate=-1&percolate_rt=0&_minpeplen=5&sessionID=all_secdisabledsession) | **494.7731** | **987.5316** | **987.5237** | **8.02** | **0** | **51** | **0.0021** | **1** | **U** | **K.LGAEGVDSLK.S** [1180](http://10.200.41.11/mascot/cgi/peptide_view.pl?file=..%2Fdata%2F20160418%2FF080015.dat&query=1180&hit=1&index=gi%7C392616945%7Cgb%7CEIW99373.1%7C&db_idx=1&px=1&section=5&ave_thresh=32&_ignoreionsscorebelow=0&report=0&_sigthreshold=0.05&_msresflags=1089&_msresflags2=2&percolate=-1&percolate_rt=0&_minpeplen=5&sessionID=all_secdisabledsession) [1181](http://10.200.41.11/mascot/cgi/peptide_view.pl?file=..%2Fdata%2F20160418%2FF080015.dat&query=1181&hit=1&index=gi%7C392616945%7Cgb%7CEIW99373.1%7C&db_idx=1&px=1&section=5&ave_thresh=32&_ignoreionsscorebelow=0&report=0&_sigthreshold=0.05&_msresflags=1089&_msresflags2=2&percolate=-1&percolate_rt=0&_minpeplen=5&sessionID=all_secdisabledsession) [1182](http://10.200.41.11/mascot/cgi/peptide_view.pl?file=..%2Fdata%2F20160418%2FF080015.dat&query=1182&hit=1&index=gi%7C392616945%7Cgb%7CEIW99373.1%7C&db_idx=1&px=1&section=5&ave_thresh=32&_ignoreionsscorebelow=0&report=0&_sigthreshold=0.05&_msresflags=1089&_msresflags2=2&percolate=-1&percolate_rt=0&_minpeplen=5&sessionID=all_secdisabledsession) [1185](http://10.200.41.11/mascot/cgi/peptide_view.pl?file=..%2Fdata%2F20160418%2FF080015.dat&query=1185&hit=1&index=gi%7C392616945%7Cgb%7CEIW99373.1%7C&db_idx=1&px=1&section=5&ave_thresh=32&_ignoreionsscorebelow=0&report=0&_sigthreshold=0.05&_msresflags=1089&_msresflags2=2&percolate=-1&percolate_rt=0&_minpeplen=5&sessionID=all_secdisabledsession) [1187](http://10.200.41.11/mascot/cgi/peptide_view.pl?file=..%2Fdata%2F20160418%2FF080015.dat&query=1187&hit=1&index=gi%7C392616945%7Cgb%7CEIW99373.1%7C&db_idx=1&px=1&section=5&ave_thresh=32&_ignoreionsscorebelow=0&report=0&_sigthreshold=0.05&_msresflags=1089&_msresflags2=2&percolate=-1&percolate_rt=0&_minpeplen=5&sessionID=all_secdisabledsession) |
|  | [1609](http://10.200.41.11/mascot/cgi/peptide_view.pl?file=..%2Fdata%2F20160418%2FF080015.dat&query=1609&hit=1&index=gi%7C392616945%7Cgb%7CEIW99373.1%7C&db_idx=1&px=1&section=5&ave_thresh=32&_ignoreionsscorebelow=0&report=0&_sigthreshold=0.05&_msresflags=1089&_msresflags2=2&percolate=-1&percolate_rt=0&_minpeplen=5&sessionID=all_secdisabledsession) | **536.7949** | **1071.5752** | **1071.5673** | **7.38** | **0** | **35** | **0.055** | **1** | **U** | **K.NVLNGQNSVK.L** [1607](http://10.200.41.11/mascot/cgi/peptide_view.pl?file=..%2Fdata%2F20160418%2FF080015.dat&query=1607&hit=1&index=gi%7C392616945%7Cgb%7CEIW99373.1%7C&db_idx=1&px=1&section=5&ave_thresh=32&_ignoreionsscorebelow=0&report=0&_sigthreshold=0.05&_msresflags=1089&_msresflags2=2&percolate=-1&percolate_rt=0&_minpeplen=5&sessionID=all_secdisabledsession) [1608](http://10.200.41.11/mascot/cgi/peptide_view.pl?file=..%2Fdata%2F20160418%2FF080015.dat&query=1608&hit=1&index=gi%7C392616945%7Cgb%7CEIW99373.1%7C&db_idx=1&px=1&section=5&ave_thresh=32&_ignoreionsscorebelow=0&report=0&_sigthreshold=0.05&_msresflags=1089&_msresflags2=2&percolate=-1&percolate_rt=0&_minpeplen=5&sessionID=all_secdisabledsession) [1610](http://10.200.41.11/mascot/cgi/peptide_view.pl?file=..%2Fdata%2F20160418%2FF080015.dat&query=1610&hit=1&index=gi%7C392616945%7Cgb%7CEIW99373.1%7C&db_idx=1&px=1&section=5&ave_thresh=32&_ignoreionsscorebelow=0&report=0&_sigthreshold=0.05&_msresflags=1089&_msresflags2=2&percolate=-1&percolate_rt=0&_minpeplen=5&sessionID=all_secdisabledsession) [1611](http://10.200.41.11/mascot/cgi/peptide_view.pl?file=..%2Fdata%2F20160418%2FF080015.dat&query=1611&hit=1&index=gi%7C392616945%7Cgb%7CEIW99373.1%7C&db_idx=1&px=1&section=5&ave_thresh=32&_ignoreionsscorebelow=0&report=0&_sigthreshold=0.05&_msresflags=1089&_msresflags2=2&percolate=-1&percolate_rt=0&_minpeplen=5&sessionID=all_secdisabledsession) |
|  | [1862](http://10.200.41.11/mascot/cgi/peptide_view.pl?file=..%2Fdata%2F20160418%2FF080015.dat&query=1862&hit=1&index=gi%7C392616945%7Cgb%7CEIW99373.1%7C&db_idx=1&px=1&section=5&ave_thresh=32&_ignoreionsscorebelow=0&report=0&_sigthreshold=0.05&_msresflags=1089&_msresflags2=2&percolate=-1&percolate_rt=0&_minpeplen=5&sessionID=all_secdisabledsession) | **579.3213** | **1156.6280** | **1156.6200** | **6.92** | **0** | **59** | **0.00023** | **1** | **U** | **K.ANEVVQTQLR.E** [1861](http://10.200.41.11/mascot/cgi/peptide_view.pl?file=..%2Fdata%2F20160418%2FF080015.dat&query=1861&hit=1&index=gi%7C392616945%7Cgb%7CEIW99373.1%7C&db_idx=1&px=1&section=5&ave_thresh=32&_ignoreionsscorebelow=0&report=0&_sigthreshold=0.05&_msresflags=1089&_msresflags2=2&percolate=-1&percolate_rt=0&_minpeplen=5&sessionID=all_secdisabledsession) |
|  | [2293](http://10.200.41.11/mascot/cgi/peptide_view.pl?file=..%2Fdata%2F20160418%2FF080015.dat&query=2293&hit=1&index=gi%7C392616945%7Cgb%7CEIW99373.1%7C&db_idx=1&px=1&section=5&ave_thresh=32&_ignoreionsscorebelow=0&report=0&_sigthreshold=0.05&_msresflags=1089&_msresflags2=2&percolate=-1&percolate_rt=0&_minpeplen=5&sessionID=all_secdisabledsession) | **635.7786** | **1269.5426** | **1269.5329** | **7.60** | **0** | **42** | **0.0019** | **1** | **U** | **R.LEQNTMDMTR.I** [2292](http://10.200.41.11/mascot/cgi/peptide_view.pl?file=..%2Fdata%2F20160418%2FF080015.dat&query=2292&hit=1&index=gi%7C392616945%7Cgb%7CEIW99373.1%7C&db_idx=1&px=1&section=5&ave_thresh=32&_ignoreionsscorebelow=0&report=0&_sigthreshold=0.05&_msresflags=1089&_msresflags2=2&percolate=-1&percolate_rt=0&_minpeplen=5&sessionID=all_secdisabledsession) |
|  | [2469](http://10.200.41.11/mascot/cgi/peptide_view.pl?file=..%2Fdata%2F20160418%2FF080015.dat&query=2469&hit=1&index=gi%7C392616945%7Cgb%7CEIW99373.1%7C&db_idx=1&px=1&section=5&ave_thresh=32&_ignoreionsscorebelow=0&report=0&_sigthreshold=0.05&_msresflags=1089&_msresflags2=2&percolate=-1&percolate_rt=0&_minpeplen=5&sessionID=all_secdisabledsession) | **651.8282** | **1301.6418** | **1301.6324** | **7.26** | **0** | **62** | **8.8e-005** | **1** | **U** | **R.SEQQISQVNNR.V** [2470](http://10.200.41.11/mascot/cgi/peptide_view.pl?file=..%2Fdata%2F20160418%2FF080015.dat&query=2470&hit=1&index=gi%7C392616945%7Cgb%7CEIW99373.1%7C&db_idx=1&px=1&section=5&ave_thresh=32&_ignoreionsscorebelow=0&report=0&_sigthreshold=0.05&_msresflags=1089&_msresflags2=2&percolate=-1&percolate_rt=0&_minpeplen=5&sessionID=all_secdisabledsession) [2471](http://10.200.41.11/mascot/cgi/peptide_view.pl?file=..%2Fdata%2F20160418%2FF080015.dat&query=2471&hit=1&index=gi%7C392616945%7Cgb%7CEIW99373.1%7C&db_idx=1&px=1&section=5&ave_thresh=32&_ignoreionsscorebelow=0&report=0&_sigthreshold=0.05&_msresflags=1089&_msresflags2=2&percolate=-1&percolate_rt=0&_minpeplen=5&sessionID=all_secdisabledsession) [2472](http://10.200.41.11/mascot/cgi/peptide_view.pl?file=..%2Fdata%2F20160418%2FF080015.dat&query=2472&hit=1&index=gi%7C392616945%7Cgb%7CEIW99373.1%7C&db_idx=1&px=1&section=5&ave_thresh=32&_ignoreionsscorebelow=0&report=0&_sigthreshold=0.05&_msresflags=1089&_msresflags2=2&percolate=-1&percolate_rt=0&_minpeplen=5&sessionID=all_secdisabledsession) [2473](http://10.200.41.11/mascot/cgi/peptide_view.pl?file=..%2Fdata%2F20160418%2FF080015.dat&query=2473&hit=1&index=gi%7C392616945%7Cgb%7CEIW99373.1%7C&db_idx=1&px=1&section=5&ave_thresh=32&_ignoreionsscorebelow=0&report=0&_sigthreshold=0.05&_msresflags=1089&_msresflags2=2&percolate=-1&percolate_rt=0&_minpeplen=5&sessionID=all_secdisabledsession) [2474](http://10.200.41.11/mascot/cgi/peptide_view.pl?file=..%2Fdata%2F20160418%2FF080015.dat&query=2474&hit=1&index=gi%7C392616945%7Cgb%7CEIW99373.1%7C&db_idx=1&px=1&section=5&ave_thresh=32&_ignoreionsscorebelow=0&report=0&_sigthreshold=0.05&_msresflags=1089&_msresflags2=2&percolate=-1&percolate_rt=0&_minpeplen=5&sessionID=all_secdisabledsession) |
|  | [2571](http://10.200.41.11/mascot/cgi/peptide_view.pl?file=..%2Fdata%2F20160418%2FF080015.dat&query=2571&hit=1&index=gi%7C392616945%7Cgb%7CEIW99373.1%7C&db_idx=1&px=1&section=5&ave_thresh=32&_ignoreionsscorebelow=0&report=0&_sigthreshold=0.05&_msresflags=1089&_msresflags2=2&percolate=-1&percolate_rt=0&_minpeplen=5&sessionID=all_secdisabledsession) | **668.3459** | **1334.6773** | **1334.6678** | **7.18** | **0** | **53** | **0.00083** | **1** | **U** | **K.SNAGTSATESLGIK.D** [2570](http://10.200.41.11/mascot/cgi/peptide_view.pl?file=..%2Fdata%2F20160418%2FF080015.dat&query=2570&hit=1&index=gi%7C392616945%7Cgb%7CEIW99373.1%7C&db_idx=1&px=1&section=5&ave_thresh=32&_ignoreionsscorebelow=0&report=0&_sigthreshold=0.05&_msresflags=1089&_msresflags2=2&percolate=-1&percolate_rt=0&_minpeplen=5&sessionID=all_secdisabledsession) |

### Protein sequence coverage: 15%

Matched peptides shown in ***bold red***.

| **1** | MLISNVGINP | AAYLNNHSVE | NSSQTASQSV | SAKDILNSIG | ISSSKVSDLG |
| --- | --- | --- | --- | --- | --- |
| **51** | LSPTLSAPAP | GVLTQTPGTI | TSFLKASIQN | TDMNQDLNAL | ANNVTTK**ANE** |
| **101** | **VVQTQLR**EQQ | AEVGKFFDIS | GMSSSAVALL | AAANTLMLTL | NQADSKLSGK |
| **151** | LSLVSFDAAK | TTASSMMREG | MNALSGSISQ | SALQLGITGV | GAKLEYKGLQ |
| **201** | NERGALKHNA | AKIDKLTTES | HSIK**NVLNGQ** | **NSVKLGAEGV** | **DSLK**SLNMKK |
| **251** | TGTDATKNLN | DATLK**SNAGT** | **SATESLGIK**D | SNKQISPEHQ | AILSKRLESV |
| **301** | ESDIR**LEQNT** | **MDMTR**IDARK | MQMTGDLIMK | NSVTVGGIAG | ASGQYAATQE |
| **351** | R**SEQQISQVN** | **NR**VASTASDE | ARESSRKSTS | LIQEMLKTME | SINQSKASAL |
| **401** | AAIAGNIRA |  |  |  |  |

| **4.** | [gi|194402702|gb|ACF62924.1|](http://10.200.41.11/mascot/cgi/protein_view.pl?file=..%2Fdata%2F20160418%2FF080015.dat&hit=gi%7C194402702%7Cgb%7CACF62924.1%7C&db_idx=1&px=1&ave_thresh=32&_ignoreionsscorebelow=0&report=0&_sigthreshold=0.05&_msresflags=1089&_msresflags2=2&percolate=-1&percolate_rt=0&_minpeplen=5&sessionID=all_secdisabledsession)    **Mass:** 72333    **Score:** 221    **Matches:** 11(7)  **Sequences:** 4(4)  **emPAI:** 0.39 |
| --- | --- |
|  | cell invasion protein SipA [Salmonella enterica subsp. enterica serovar Newport str. SL254] |

|  | **Query** | **Observed** | **Mr(expt)** | **Mr(calc)** | **ppm** | **Miss** | **Score** | **Expect** | **Rank** | **Unique** | **Peptide** |
| --- | --- | --- | --- | --- | --- | --- | --- | --- | --- | --- | --- |
|  | [1110](http://10.200.41.11/mascot/cgi/peptide_view.pl?file=..%2Fdata%2F20160418%2FF080015.dat&query=1110&hit=1&index=gi%7C194402702%7Cgb%7CACF62924.1%7C&db_idx=1&px=1&section=5&ave_thresh=32&_ignoreionsscorebelow=0&report=0&_sigthreshold=0.05&_msresflags=1089&_msresflags2=2&percolate=-1&percolate_rt=0&_minpeplen=5&sessionID=all_secdisabledsession) | **490.7472** | **979.4798** | **979.4723** | **7.68** | **0** | **39** | **0.024** | **1** | **U** | **R.TFIDNSQR.N** [1109](http://10.200.41.11/mascot/cgi/peptide_view.pl?file=..%2Fdata%2F20160418%2FF080015.dat&query=1109&hit=1&index=gi%7C194402702%7Cgb%7CACF62924.1%7C&db_idx=1&px=1&section=5&ave_thresh=32&_ignoreionsscorebelow=0&report=0&_sigthreshold=0.05&_msresflags=1089&_msresflags2=2&percolate=-1&percolate_rt=0&_minpeplen=5&sessionID=all_secdisabledsession) [1111](http://10.200.41.11/mascot/cgi/peptide_view.pl?file=..%2Fdata%2F20160418%2FF080015.dat&query=1111&hit=1&index=gi%7C194402702%7Cgb%7CACF62924.1%7C&db_idx=1&px=1&section=5&ave_thresh=32&_ignoreionsscorebelow=0&report=0&_sigthreshold=0.05&_msresflags=1089&_msresflags2=2&percolate=-1&percolate_rt=0&_minpeplen=5&sessionID=all_secdisabledsession) [1112](http://10.200.41.11/mascot/cgi/peptide_view.pl?file=..%2Fdata%2F20160418%2FF080015.dat&query=1112&hit=1&index=gi%7C194402702%7Cgb%7CACF62924.1%7C&db_idx=1&px=1&section=5&ave_thresh=32&_ignoreionsscorebelow=0&report=0&_sigthreshold=0.05&_msresflags=1089&_msresflags2=2&percolate=-1&percolate_rt=0&_minpeplen=5&sessionID=all_secdisabledsession) [1113](http://10.200.41.11/mascot/cgi/peptide_view.pl?file=..%2Fdata%2F20160418%2FF080015.dat&query=1113&hit=1&index=gi%7C194402702%7Cgb%7CACF62924.1%7C&db_idx=1&px=1&section=5&ave_thresh=32&_ignoreionsscorebelow=0&report=0&_sigthreshold=0.05&_msresflags=1089&_msresflags2=2&percolate=-1&percolate_rt=0&_minpeplen=5&sessionID=all_secdisabledsession) [1114](http://10.200.41.11/mascot/cgi/peptide_view.pl?file=..%2Fdata%2F20160418%2FF080015.dat&query=1114&hit=1&index=gi%7C194402702%7Cgb%7CACF62924.1%7C&db_idx=1&px=1&section=5&ave_thresh=32&_ignoreionsscorebelow=0&report=0&_sigthreshold=0.05&_msresflags=1089&_msresflags2=2&percolate=-1&percolate_rt=0&_minpeplen=5&sessionID=all_secdisabledsession) |
|  | [1956](http://10.200.41.11/mascot/cgi/peptide_view.pl?file=..%2Fdata%2F20160418%2FF080015.dat&query=1956&hit=1&index=gi%7C194402702%7Cgb%7CACF62924.1%7C&db_idx=1&px=1&section=5&ave_thresh=32&_ignoreionsscorebelow=0&report=0&_sigthreshold=0.05&_msresflags=1089&_msresflags2=2&percolate=-1&percolate_rt=0&_minpeplen=5&sessionID=all_secdisabledsession) | **588.3092** | **1174.6039** | **1174.5942** | **8.20** | **0** | **57** | **0.00037** | **1** | **U** | **K.LTQEQGTSVGR.E** [1955](http://10.200.41.11/mascot/cgi/peptide_view.pl?file=..%2Fdata%2F20160418%2FF080015.dat&query=1955&hit=1&index=gi%7C194402702%7Cgb%7CACF62924.1%7C&db_idx=1&px=1&section=5&ave_thresh=32&_ignoreionsscorebelow=0&report=0&_sigthreshold=0.05&_msresflags=1089&_msresflags2=2&percolate=-1&percolate_rt=0&_minpeplen=5&sessionID=all_secdisabledsession) [1957](http://10.200.41.11/mascot/cgi/peptide_view.pl?file=..%2Fdata%2F20160418%2FF080015.dat&query=1957&hit=1&index=gi%7C194402702%7Cgb%7CACF62924.1%7C&db_idx=1&px=1&section=5&ave_thresh=32&_ignoreionsscorebelow=0&report=0&_sigthreshold=0.05&_msresflags=1089&_msresflags2=2&percolate=-1&percolate_rt=0&_minpeplen=5&sessionID=all_secdisabledsession) |
|  | [2403](http://10.200.41.11/mascot/cgi/peptide_view.pl?file=..%2Fdata%2F20160418%2FF080015.dat&query=2403&hit=1&index=gi%7C194402702%7Cgb%7CACF62924.1%7C&db_idx=1&px=1&section=5&ave_thresh=32&_ignoreionsscorebelow=0&report=0&_sigthreshold=0.05&_msresflags=1089&_msresflags2=2&percolate=-1&percolate_rt=0&_minpeplen=5&sessionID=all_secdisabledsession) | **645.8172** | **1289.6198** | **1289.6099** | **7.72** | **0** | **49** | **0.0014** | **1** | **U** | **R.SALNATSDSPEAK.T** |
|  | [3196](http://10.200.41.11/mascot/cgi/peptide_view.pl?file=..%2Fdata%2F20160418%2FF080015.dat&query=3196&hit=1&index=gi%7C194402702%7Cgb%7CACF62924.1%7C&db_idx=1&px=1&section=5&ave_thresh=32&_ignoreionsscorebelow=0&report=0&_sigthreshold=0.05&_msresflags=1089&_msresflags2=2&percolate=-1&percolate_rt=0&_minpeplen=5&sessionID=all_secdisabledsession) | **915.9219** | **1829.8292** | **1829.8167** | **6.82** | **0** | **69** | **8.8e-006** | **1** | **U** | **K.GTTGETTSFDEVDGVTSK.S** |

### Protein sequence coverage: 7%

Matched peptides shown in ***bold red***.

| **1** | MQTEIKTQAT | NLAANLSAVR | ESATTTLSGE | IKGPQLEDFP | ALIKQASLDA |
| --- | --- | --- | --- | --- | --- |
| **51** | LFKCGKDAEA | LKEVFTNSNN | VAGKKAIMEF | AGLFR**SALNA** | **TSDSPEAK**TL |
| **101** | LMKVGAEYTA | QIIKDGLKEK | SAFGPWLPET | KKAEAKLENL | EKQLLDIIKN |
| **151** | NTGGELSKLS | TNLVMQEVMP | YIASCIEHNF | GCTLDPLTRS | NLTHLVDKAA |
| **201** | AKAVEALDMC | HQK**LTQEQGT** | **SVGR**EARHLE | MQTLIPLLLR | NVFAQIPADK |
| **251** | LPDPKIPEPA | AGPVPDGGKK | AEPTGININI | NIDSSNHSVD | NSKHINNSRS |
| **301** | HVDNSQRHID | NSNHDNSRKT | IDNSR**TFIDN** | **SQR**NGESHHS | TNSSNVSHSH |
| **351** | SRVDSTTHQT | ETAHSASTGA | IDHGIAGKID | VTAHATAEAV | TNASSESKDG |
| **401** | KVVTSEK**GTT** | **GETTSFDEVD** | **GVTSK**SIIGK | PVQATVHGVD | DNKQQSQTAE |
| **451** | IVNVKPLASQ | LAGVENVKTD | TLQSDTTVIT | GNKAGTTDND | NSQTDKTGPF |
| **501** | SGLKFKQNSF | LSTVPSVTNM | HSMHFDARET | FLGVIRKALE | PDTSTPFPVR |
| **551** | RAFDGLRAEI | LPNDTIKSAA | LKAQCSDIDK | HPELKAKMET | LKEVITHHPQ |
| **601** | KEKLAEIALQ | FAREAGLTRL | KGETDYVLSN | VLDGLIGDGS | WRAGPAYESY |
| **651** | LNKPGVDRVI | TTVDGLHMQR |  |  |  |

| **5.** | [gi|194403331|gb|ACF63553.1|](http://10.200.41.11/mascot/cgi/protein_view.pl?file=..%2Fdata%2F20160418%2FF080015.dat&hit=gi%7C194403331%7Cgb%7CACF63553.1%7C&db_idx=1&px=1&ave_thresh=32&_ignoreionsscorebelow=0&report=0&_sigthreshold=0.05&_msresflags=1089&_msresflags2=2&percolate=-1&percolate_rt=0&_minpeplen=5&sessionID=all_secdisabledsession)    **Mass:** 10561    **Score:** 198    **Matches:** 4(4)  **Sequences:** 1(1)  **emPAI:** 0.71 |
| --- | --- |
|  | negative regulator of flagellin synthesis [Salmonella enterica subsp. enterica serovar Newport str. SL254] |

|  | **Query** | **Observed** | **Mr(expt)** | **Mr(calc)** | **ppm** | **Miss** | **Score** | **Expect** | **Rank** | **Unique** | **Peptide** |
| --- | --- | --- | --- | --- | --- | --- | --- | --- | --- | --- | --- |
|  | [2998](http://10.200.41.11/mascot/cgi/peptide_view.pl?file=..%2Fdata%2F20160418%2FF080015.dat&query=2998&hit=1&index=gi%7C194403331%7Cgb%7CACF63553.1%7C&db_idx=1&px=1&section=5&ave_thresh=32&_ignoreionsscorebelow=0&report=0&_sigthreshold=0.05&_msresflags=1089&_msresflags2=2&percolate=-1&percolate_rt=0&_minpeplen=5&sessionID=all_secdisabledsession) | **761.3605** | **1520.7064** | **1520.6963** | **6.64** | **0** | **66** | **2.8e-005** | **1** | **U** | **K.LMQPGVSDINMER.V** [2995](http://10.200.41.11/mascot/cgi/peptide_view.pl?file=..%2Fdata%2F20160418%2FF080015.dat&query=2995&hit=1&index=gi%7C194403331%7Cgb%7CACF63553.1%7C&db_idx=1&px=1&section=5&ave_thresh=32&_ignoreionsscorebelow=0&report=0&_sigthreshold=0.05&_msresflags=1089&_msresflags2=2&percolate=-1&percolate_rt=0&_minpeplen=5&sessionID=all_secdisabledsession) [2996](http://10.200.41.11/mascot/cgi/peptide_view.pl?file=..%2Fdata%2F20160418%2FF080015.dat&query=2996&hit=1&index=gi%7C194403331%7Cgb%7CACF63553.1%7C&db_idx=1&px=1&section=5&ave_thresh=32&_ignoreionsscorebelow=0&report=0&_sigthreshold=0.05&_msresflags=1089&_msresflags2=2&percolate=-1&percolate_rt=0&_minpeplen=5&sessionID=all_secdisabledsession) [2997](http://10.200.41.11/mascot/cgi/peptide_view.pl?file=..%2Fdata%2F20160418%2FF080015.dat&query=2997&hit=1&index=gi%7C194403331%7Cgb%7CACF63553.1%7C&db_idx=1&px=1&section=5&ave_thresh=32&_ignoreionsscorebelow=0&report=0&_sigthreshold=0.05&_msresflags=1089&_msresflags2=2&percolate=-1&percolate_rt=0&_minpeplen=5&sessionID=all_secdisabledsession) |

### Protein sequence coverage: 13%

Matched peptides shown in ***bold red***.

| **1** | MSIDRTSPLK | PVSTVQTRET | SDTPVQKTRQ | EKTSAATSAS | VTLSDAQAK**L** |
| --- | --- | --- | --- | --- | --- |
| **51** | **MQPGVSDINM** | **ER**VEALKTAI | RNGELKMDTG | KIADSLIREA | QSYLQSK |

| **7.** | [gi|874404664|gb|KMU13862.1|](http://10.200.41.11/mascot/cgi/protein_view.pl?file=..%2Fdata%2F20160418%2FF080015.dat&hit=gi%7C874404664%7Cgb%7CKMU13862.1%7C&db_idx=1&px=1&ave_thresh=32&_ignoreionsscorebelow=0&report=0&_sigthreshold=0.05&_msresflags=1089&_msresflags2=2&percolate=-1&percolate_rt=0&_minpeplen=5&sessionID=all_secdisabledsession)    **Mass:** 34557    **Score:** 117    **Matches:** 8(2)  **Sequences:** 5(2)  **emPAI:** 0.41 |
| --- | --- |
|  | flagellin [Salmonella enterica subsp. enterica serovar Newport str. DC_10-446] |

|  | **Query** | **Observed** | **Mr(expt)** | **Mr(calc)** | **ppm** | **Miss** | **Score** | **Expect** | **Rank** | **Unique** | **Peptide** |
| --- | --- | --- | --- | --- | --- | --- | --- | --- | --- | --- | --- |
|  | [8](http://10.200.41.11/mascot/cgi/peptide_view.pl?file=..%2Fdata%2F20160418%2FF080015.dat&query=8&hit=3&index=gi%7C874404664%7Cgb%7CKMU13862.1%7C&db_idx=1&px=1&section=5&ave_thresh=32&_ignoreionsscorebelow=0&report=0&_sigthreshold=0.05&_msresflags=1089&_msresflags2=2&percolate=-1&percolate_rt=0&_minpeplen=5&sessionID=all_secdisabledsession) | **402.2209** | **802.4273** | **802.4185** | **11.1** | **0** | **1** | **1.5e+002** | **3** | **U** | **M.DSLNVQK.A** [26](http://10.200.41.11/mascot/cgi/peptide_view.pl?file=..%2Fdata%2F20160418%2FF080015.dat&query=26&hit=1&index=gi%7C874404664%7Cgb%7CKMU13862.1%7C&db_idx=1&px=1&section=5&ave_thresh=32&_ignoreionsscorebelow=0&report=0&_sigthreshold=0.05&_msresflags=1089&_msresflags2=2&percolate=-1&percolate_rt=0&_minpeplen=5&sessionID=all_secdisabledsession) |
|  | [935](http://10.200.41.11/mascot/cgi/peptide_view.pl?file=..%2Fdata%2F20160418%2FF080015.dat&query=935&hit=1&index=gi%7C874404664%7Cgb%7CKMU13862.1%7C&db_idx=1&px=1&section=5&ave_thresh=32&_ignoreionsscorebelow=0&report=0&_sigthreshold=0.05&_msresflags=1089&_msresflags2=2&percolate=-1&percolate_rt=0&_minpeplen=5&sessionID=all_secdisabledsession) | 481.2675 | 960.5205 | 960.5128 | 8.08 | 0 | 34 | 0.082 | 1 |  | K.TEVVTIDGK.T [933](http://10.200.41.11/mascot/cgi/peptide_view.pl?file=..%2Fdata%2F20160418%2FF080015.dat&query=933&hit=1&index=gi%7C874404664%7Cgb%7CKMU13862.1%7C&db_idx=1&px=1&section=5&ave_thresh=32&_ignoreionsscorebelow=0&report=0&_sigthreshold=0.05&_msresflags=1089&_msresflags2=2&percolate=-1&percolate_rt=0&_minpeplen=5&sessionID=all_secdisabledsession) [934](http://10.200.41.11/mascot/cgi/peptide_view.pl?file=..%2Fdata%2F20160418%2FF080015.dat&query=934&hit=1&index=gi%7C874404664%7Cgb%7CKMU13862.1%7C&db_idx=1&px=1&section=5&ave_thresh=32&_ignoreionsscorebelow=0&report=0&_sigthreshold=0.05&_msresflags=1089&_msresflags2=2&percolate=-1&percolate_rt=0&_minpeplen=5&sessionID=all_secdisabledsession) |
|  | [1655](http://10.200.41.11/mascot/cgi/peptide_view.pl?file=..%2Fdata%2F20160418%2FF080015.dat&query=1655&hit=1&index=gi%7C874404664%7Cgb%7CKMU13862.1%7C&db_idx=1&px=1&section=5&ave_thresh=32&_ignoreionsscorebelow=0&report=0&_sigthreshold=0.05&_msresflags=1089&_msresflags2=2&percolate=-1&percolate_rt=0&_minpeplen=5&sessionID=all_secdisabledsession) | 549.7971 | 1097.5797 | 1097.5716 | 7.33 | 0 | 2 | 85 | 1 |  | K.AQPELAEAAAK.T |
|  | [1731](http://10.200.41.11/mascot/cgi/peptide_view.pl?file=..%2Fdata%2F20160418%2FF080015.dat&query=1731&hit=1&index=gi%7C874404664%7Cgb%7CKMU13862.1%7C&db_idx=1&px=1&section=5&ave_thresh=32&_ignoreionsscorebelow=0&report=0&_sigthreshold=0.05&_msresflags=1089&_msresflags2=2&percolate=-1&percolate_rt=0&_minpeplen=5&sessionID=all_secdisabledsession) | 565.7976 | 1129.5807 | 1129.5728 | 7.00 | 0 | 51 | 0.0015 | 1 |  | K.TAANQLGGVDGK.T |
|  | [3216](http://10.200.41.11/mascot/cgi/peptide_view.pl?file=..%2Fdata%2F20160418%2FF080015.dat&query=3216&hit=1&index=gi%7C874404664%7Cgb%7CKMU13862.1%7C&db_idx=1&px=1&section=5&ave_thresh=32&_ignoreionsscorebelow=0&report=0&_sigthreshold=0.05&_msresflags=1089&_msresflags2=2&percolate=-1&percolate_rt=0&_minpeplen=5&sessionID=all_secdisabledsession) | 659.2984 | 1974.8734 | 1974.8589 | 7.34 | 1 | 38 | 0.0064 | 1 |  | R.SRIEDSDYATEVSNMSR.A |

### Protein sequence coverage: 16%

Matched peptides shown in ***bold red***.

| **1** | M**DSLNVQK**AY | DVKDTAVTTK | AYANNGTTLD | VSGLDDAAIK | AATGGTNGTA |
| --- | --- | --- | --- | --- | --- |
| **51** | SVTGGAVKFD | ADNNKYFVTI | GGFTGADAAK | NGDYEVNVAT | DGTVTLAAGA |
| **101** | TKTTMPAGAT | TKTEVQELKD | TPAVVSADAK | NALIAGGVDA | TDANGAELVK |
| **151** | MSYTDKNGKT | IEGGYALKAG | DKYYAADYDE | ATGAIKAKTT | SYTAADGTTK |
| **201** | **TAANQLGGVD** | **GKTEVVTIDG** | **K**TYNASKAAG | HDFK**AQPELA** | **EAAAK**TTENP |
| **251** | LQKIDAALAQ | VDALRSDLGA | VQNRFNSAIT | NLGNTVNNLS | EAR**SRIEDSD** |
| **301** | **YATEVSNMSR** | AQILQQAGTS | VLAQANQVPQ | NVLSLLR |  |

| **9.** | [gi|194403640|gb|ACF63862.1|](http://10.200.41.11/mascot/cgi/protein_view.pl?file=..%2Fdata%2F20160418%2FF080015.dat&hit=gi%7C194403640%7Cgb%7CACF63862.1%7C&db_idx=1&px=1&ave_thresh=32&_ignoreionsscorebelow=0&report=0&_sigthreshold=0.05&_msresflags=1089&_msresflags2=2&percolate=-1&percolate_rt=0&_minpeplen=5&sessionID=all_secdisabledsession)    **Mass:** 62382    **Score:** 101    **Matches:** 6(3)  **Sequences:** 3(2)  **emPAI:** 0.21 |
| --- | --- |
|  | cell invasion protein SipB [Salmonella enterica subsp. enterica serovar Newport str. SL254] |

|  | **Query** | **Observed** | **Mr(expt)** | **Mr(calc)** | **ppm** | **Miss** | **Score** | **Expect** | **Rank** | **Unique** | **Peptide** |
| --- | --- | --- | --- | --- | --- | --- | --- | --- | --- | --- | --- |
|  | [735](http://10.200.41.11/mascot/cgi/peptide_view.pl?file=..%2Fdata%2F20160418%2FF080015.dat&query=735&hit=1&index=gi%7C194403640%7Cgb%7CACF63862.1%7C&db_idx=1&px=1&section=5&ave_thresh=32&_ignoreionsscorebelow=0&report=0&_sigthreshold=0.05&_msresflags=1089&_msresflags2=2&percolate=-1&percolate_rt=0&_minpeplen=5&sessionID=all_secdisabledsession) | **461.7263** | **921.4380** | **921.4304** | **8.17** | **0** | **41** | **0.0089** | **1** | **U** | **R.SGYTQNPR.L** [734](http://10.200.41.11/mascot/cgi/peptide_view.pl?file=..%2Fdata%2F20160418%2FF080015.dat&query=734&hit=1&index=gi%7C194403640%7Cgb%7CACF63862.1%7C&db_idx=1&px=1&section=5&ave_thresh=32&_ignoreionsscorebelow=0&report=0&_sigthreshold=0.05&_msresflags=1089&_msresflags2=2&percolate=-1&percolate_rt=0&_minpeplen=5&sessionID=all_secdisabledsession) |
|  | [1111](http://10.200.41.11/mascot/cgi/peptide_view.pl?file=..%2Fdata%2F20160418%2FF080015.dat&query=1111&hit=3&index=gi%7C194403640%7Cgb%7CACF63862.1%7C&db_idx=1&px=1&section=5&ave_thresh=32&_ignoreionsscorebelow=0&report=0&_sigthreshold=0.05&_msresflags=1089&_msresflags2=2&percolate=-1&percolate_rt=0&_minpeplen=5&sessionID=all_secdisabledsession) | 490.7473 | 979.4801 | 979.4909 | -11.09 | 0 | 3 | 87 | 3 | U | K.LFTQGMQR.I [1109](http://10.200.41.11/mascot/cgi/peptide_view.pl?file=..%2Fdata%2F20160418%2FF080015.dat&query=1109&hit=9&index=gi%7C194403640%7Cgb%7CACF63862.1%7C&db_idx=1&px=1&section=5&ave_thresh=32&_ignoreionsscorebelow=0&report=0&_sigthreshold=0.05&_msresflags=1089&_msresflags2=2&percolate=-1&percolate_rt=0&_minpeplen=5&sessionID=all_secdisabledsession) [1110](http://10.200.41.11/mascot/cgi/peptide_view.pl?file=..%2Fdata%2F20160418%2FF080015.dat&query=1110&hit=6&index=gi%7C194403640%7Cgb%7CACF63862.1%7C&db_idx=1&px=1&section=5&ave_thresh=32&_ignoreionsscorebelow=0&report=0&_sigthreshold=0.05&_msresflags=1089&_msresflags2=2&percolate=-1&percolate_rt=0&_minpeplen=5&sessionID=all_secdisabledsession) |
|  | [1582](http://10.200.41.11/mascot/cgi/peptide_view.pl?file=..%2Fdata%2F20160418%2FF080015.dat&query=1582&hit=1&index=gi%7C194403640%7Cgb%7CACF63862.1%7C&db_idx=1&px=1&section=5&ave_thresh=32&_ignoreionsscorebelow=0&report=0&_sigthreshold=0.05&_msresflags=1089&_msresflags2=2&percolate=-1&percolate_rt=0&_minpeplen=5&sessionID=all_secdisabledsession) | **531.7869** | **1061.5592** | **1061.5505** | **8.14** | **0** | **58** | **0.00031** | **1** | **U** | **R.LAEAAFEGVR.K** |

### Protein sequence coverage: 4%

Matched peptides shown in ***bold red***.

| **1** | MVNDASSISR | **SGYTQNPRLA** | **EAAFEGVR**KN | TDFLKAADKA | FKDVVATKAG |
| --- | --- | --- | --- | --- | --- |
| **51** | DLKAGTKSGE | SAINTVGLKP | PTDAAREKLS | SEGQLTLLLG | KLMTLLGDVS |
| **101** | LSQLESRLAV | WQAMIESQKE | MGIQVSKEFQ | TALGEAQEAT | DLYEASIKKT |
| **151** | DTAKSVYDAA | AKKLTQAQNK | LQSLDPADPG | YAQAEAAVEQ | AGKEATEAKE |
| **201** | ALDKATDATV | KAGTDAKAKA | EKADNILTKF | QGTANAASQN | QVSQGEQDNL |
| **251** | SNVARLTMLM | AMFIEIVGKN | TEESLQNDLA | LFNALQEGRQ | AEMEKKSAEF |
| **301** | QEETRKAEET | NRIMGCIGKV | LGALLTIVSV | VAAVFTGGAS | LALAAVGLAV |
| **351** | MVADEIVKAA | TGVSFIQQAL | NPIMEHVLKP | LMELIGKAIT | KALEGLGVDK |
| **401** | KTAEMAGSIV | GAIVAAIAMV | AVIVVVAVVG | KGAAAKLGNA | LSKMMGETIK |
| **451** | KLVPNVLKQL | AQNGSK**LFTQ** | **GMQR**ITSGLG | NVGSKMGLQT | NALSKELVGN |
| **501** | TLNKVALGME | VTNTAAQSAG | GVAEGVFIKN | ASEALADFML | ARFAMDQIQQ |
| **551** | WLKQSVEIFG | ENQKVTAELQ | KAMSSAVQQN | ADASRFILRQ | SRA |

| **14.** | [gi|194404219|gb|ACF64441.1|](http://10.200.41.11/mascot/cgi/protein_view.pl?file=..%2Fdata%2F20160418%2FF080015.dat&hit=gi%7C194404219%7Cgb%7CACF64441.1%7C&db_idx=1&px=1&ave_thresh=32&_ignoreionsscorebelow=0&report=0&_sigthreshold=0.05&_msresflags=1089&_msresflags2=2&percolate=-1&percolate_rt=0&_minpeplen=5&sessionID=all_secdisabledsession)    **Mass:** 34155    **Score:** 48     **Matches:** 3(2)  **Sequences:** 1(1)  **emPAI:** 0.19 |
| --- | --- |
|  | flagellar hook-associated protein 3 [Salmonella enterica subsp. enterica serovar Newport str. SL254] |

|  | **Query** | **Observed** | **Mr(expt)** | **Mr(calc)** | **ppm** | **Miss** | **Score** | **Expect** | **Rank** | **Unique** | **Peptide** |
| --- | --- | --- | --- | --- | --- | --- | --- | --- | --- | --- | --- |
|  | [1617](http://10.200.41.11/mascot/cgi/peptide_view.pl?file=..%2Fdata%2F20160418%2FF080015.dat&query=1617&hit=1&index=gi%7C194404219%7Cgb%7CACF64441.1%7C&db_idx=1&px=1&section=5&ave_thresh=32&_ignoreionsscorebelow=0&report=0&_sigthreshold=0.05&_msresflags=1089&_msresflags2=2&percolate=-1&percolate_rt=0&_minpeplen=5&sessionID=all_secdisabledsession) | **545.7822** | **1089.5499** | **1089.5415** | **7.75** | **0** | **37** | **0.04** | **1** | **U** | **K.SVTQQVDSAR.T** [1615](http://10.200.41.11/mascot/cgi/peptide_view.pl?file=..%2Fdata%2F20160418%2FF080015.dat&query=1615&hit=1&index=gi%7C194404219%7Cgb%7CACF64441.1%7C&db_idx=1&px=1&section=5&ave_thresh=32&_ignoreionsscorebelow=0&report=0&_sigthreshold=0.05&_msresflags=1089&_msresflags2=2&percolate=-1&percolate_rt=0&_minpeplen=5&sessionID=all_secdisabledsession) [1616](http://10.200.41.11/mascot/cgi/peptide_view.pl?file=..%2Fdata%2F20160418%2FF080015.dat&query=1616&hit=1&index=gi%7C194404219%7Cgb%7CACF64441.1%7C&db_idx=1&px=1&section=5&ave_thresh=32&_ignoreionsscorebelow=0&report=0&_sigthreshold=0.05&_msresflags=1089&_msresflags2=2&percolate=-1&percolate_rt=0&_minpeplen=5&sessionID=all_secdisabledsession) |

### Protein sequence coverage: 3%

Matched peptides shown in ***bold red***.

| **1** | MRISTQMMYE | QNMSGITNSQ | AEWMKLGEQM | STGKRVTNPS | DDPIAASQAV |
| --- | --- | --- | --- | --- | --- |
| **51** | VLSQAQAQNS | QYALARTFAT | QKVSLEESVL | SQVTTAIQTA | QEKIVYAGNG |
| **101** | TLSDDDRASL | ATDLQGIRDQ | LMNLANSTDG | NGRYIFAGYK | TEAAPFDQAT |
| **151** | GGYHGGEK**SV** | **TQQVDSAR**TM | VIGHTGAQIF | NSITSNAVPE | PDGSDSEKNL |
| **201** | FVMLDTAIAA | LKTPVEGNDV | EKEKAAAAID | KTNRGLKNSL | NNVLTVRAEL |
| **251** | GTQLSELSTL | DSLGSDRALG | QKLQMSNLVD | VDWNSVISSY | VMQQAALQAS |
| **301** | YKTFTDMQGM | SLFQLNR |  |  |  |

*******************************************************************************************************************************************************

**3^rd^ technical replicate**

**Enzyme : Trypsin**

**Variable modifications :** [**Oxidation (M)**](http://10.200.41.11/mascot/cgi/client.pl?modification&mod_name=Oxidation%20%28M%29&file=..%2Fdata%2F20160418%2FF080018.dat)

**Mass values : Monoisotopic**

**Protein Mass : Unrestricted**

**Peptide Mass Tolerance : ± 20 ppm**

**Fragment Mass Tolerance: ± 0.4 Da**

**Max Missed Cleavages : 3**

**Instrument type : Default**

**Number of queries : 3357**

| **Protein hits           :** | [**gi|392616945|gb|EIW99373.1|**](http://10.200.41.11/mascot/cgi/master_results.pl?file=..%2Fdata%2F20160418%2FF080018.dat#Hit1) | pathogenicity island 1 effector protein SipC [Salmonella enterica subsp. enterica serovar Newport str. Levine 15] |
| --- | --- | --- |
|  | [**gi|194403331|gb|ACF63553.1|**](http://10.200.41.11/mascot/cgi/master_results.pl?file=..%2Fdata%2F20160418%2FF080018.dat#Hit2) | negative regulator of flagellin synthesis [Salmonella enterica subsp. enterica serovar Newport str. SL254] |
|  | [**gi|50830890|gb|AAT81610.1|**](http://10.200.41.11/mascot/cgi/master_results.pl?file=..%2Fdata%2F20160418%2FF080018.dat#Hit3) | phase 1 flagellin [Salmonella enterica subsp. enterica serovar Newport] |
|  | [**gi|194401698|gb|ACF61920.1|**](http://10.200.41.11/mascot/cgi/master_results.pl?file=..%2Fdata%2F20160418%2FF080018.dat#Hit4) | translation elongation factor Tu [Salmonella enterica subsp. enterica serovar Newport str. SL254] |
|  | [**gi|194402702|gb|ACF62924.1|**](http://10.200.41.11/mascot/cgi/master_results.pl?file=..%2Fdata%2F20160418%2FF080018.dat#Hit5) | cell invasion protein SipA [Salmonella enterica subsp. enterica serovar Newport str. SL254] |
|  | [**gi|692117421|ref|WP_032074328.1|**](http://10.200.41.11/mascot/cgi/master_results.pl?file=..%2Fdata%2F20160418%2FF080018.dat#Hit6) | hypothetical protein [Salmonella enterica] |
|  | [**gi|194401176|gb|ACF61398.1|**](http://10.200.41.11/mascot/cgi/master_results.pl?file=..%2Fdata%2F20160418%2FF080018.dat#Hit7) | ribosomal protein L7/L12 [Salmonella enterica subsp. enterica serovar Newport str. SL254] |
|  | [**gi|194402309|gb|ACF62531.1|**](http://10.200.41.11/mascot/cgi/master_results.pl?file=..%2Fdata%2F20160418%2FF080018.dat#Hit8) | ribosomal protein S10 [Salmonella enterica subsp. enterica serovar Newport str. SL254] |
|  | [**gi|194403640|gb|ACF63862.1|**](http://10.200.41.11/mascot/cgi/master_results.pl?file=..%2Fdata%2F20160418%2FF080018.dat#Hit9) | cell invasion protein SipB [Salmonella enterica subsp. enterica serovar Newport str. SL254] |
|  | [**gi|194401878|gb|ACF62100.1|**](http://10.200.41.11/mascot/cgi/master_results.pl?file=..%2Fdata%2F20160418%2FF080018.dat#Hit10) | ribosomal protein L11 [Salmonella enterica subsp. enterica serovar Newport str. SL254] |
|  | [**gi|194401103|gb|ACF61325.1|**](http://10.200.41.11/mascot/cgi/master_results.pl?file=..%2Fdata%2F20160418%2FF080018.dat#Hit11) | ribosomal protein L6 [Salmonella enterica subsp. enterica serovar Newport str. SL254] |
|  | [**gi|194401633|gb|ACF61855.1|**](http://10.200.41.11/mascot/cgi/master_results.pl?file=..%2Fdata%2F20160418%2FF080018.dat#Hit12) | ribosomal protein S8 [Salmonella enterica subsp. enterica serovar Newport str. SL254] |
|  | [**gi|194403829|gb|ACF64051.1|**](http://10.200.41.11/mascot/cgi/master_results.pl?file=..%2Fdata%2F20160418%2FF080018.dat#Hit13) | ribosomal protein L3 [Salmonella enterica subsp. enterica serovar Newport str. SL254] |
|  | [**gi|392807267|gb|EJA63344.1|**](http://10.200.41.11/mascot/cgi/master_results.pl?file=..%2Fdata%2F20160418%2FF080018.dat#Hit14) | hypothetical protein SEEN443_08957 [Salmonella enterica subsp. enterica serovar Newport str. CVM 19443] |
|  | [**gi|194405130|gb|ACF65352.1|**](http://10.200.41.11/mascot/cgi/master_results.pl?file=..%2Fdata%2F20160418%2FF080018.dat#Hit15) | inositol phosphate phosphatase SopB [Salmonella enterica subsp. enterica serovar Newport str. SL254] |

| **1.** | [gi|392616945|gb|EIW99373.1|](http://10.200.41.11/mascot/cgi/protein_view.pl?file=..%2Fdata%2F20160418%2FF080018.dat&hit=gi%7C392616945%7Cgb%7CEIW99373.1%7C&db_idx=1&px=1&ave_thresh=32&_ignoreionsscorebelow=0&report=0&_sigthreshold=0.05&_msresflags=1089&_msresflags2=2&percolate=-1&percolate_rt=0&_minpeplen=5&sessionID=all_secdisabledsession)    **Mass:** 42957    **Score:** 354    **Matches:** 14(10)  **Sequences:** 5(4)  **emPAI:** 0.67 |
| --- | --- |
|  | pathogenicity island 1 effector protein SipC [Salmonella enterica subsp. enterica serovar Newport str. Levine 15] |

|  | **Query** | **Observed** | **Mr(expt)** | **Mr(calc)** | **ppm** | **Miss** | **Score** | **Expect** | **Rank** | **Unique** | **Peptide** |
| --- | --- | --- | --- | --- | --- | --- | --- | --- | --- | --- | --- |
|  | [837](http://10.200.41.11/mascot/cgi/peptide_view.pl?file=..%2Fdata%2F20160418%2FF080018.dat&query=837&hit=1&index=gi%7C392616945%7Cgb%7CEIW99373.1%7C&db_idx=1&px=1&section=5&ave_thresh=32&_ignoreionsscorebelow=0&report=0&_sigthreshold=0.05&_msresflags=1089&_msresflags2=2&percolate=-1&percolate_rt=0&_minpeplen=5&sessionID=all_secdisabledsession) | **494.7729** | **987.5313** | **987.5237** | **7.78** | **0** | **44** | **0.01** | **1** | **U** | **K.LGAEGVDSLK.S** |
|  | [1115](http://10.200.41.11/mascot/cgi/peptide_view.pl?file=..%2Fdata%2F20160418%2FF080018.dat&query=1115&hit=1&index=gi%7C392616945%7Cgb%7CEIW99373.1%7C&db_idx=1&px=1&section=5&ave_thresh=32&_ignoreionsscorebelow=0&report=0&_sigthreshold=0.05&_msresflags=1089&_msresflags2=2&percolate=-1&percolate_rt=0&_minpeplen=5&sessionID=all_secdisabledsession) | **527.2516** | **1052.4886** | **1052.4808** | **7.43** | **0** | **36** | **0.018** | **1** | **U** | **K.TMESINQSK.A** [1112](http://10.200.41.11/mascot/cgi/peptide_view.pl?file=..%2Fdata%2F20160418%2FF080018.dat&query=1112&hit=1&index=gi%7C392616945%7Cgb%7CEIW99373.1%7C&db_idx=1&px=1&section=5&ave_thresh=32&_ignoreionsscorebelow=0&report=0&_sigthreshold=0.05&_msresflags=1089&_msresflags2=2&percolate=-1&percolate_rt=0&_minpeplen=5&sessionID=all_secdisabledsession) [1113](http://10.200.41.11/mascot/cgi/peptide_view.pl?file=..%2Fdata%2F20160418%2FF080018.dat&query=1113&hit=1&index=gi%7C392616945%7Cgb%7CEIW99373.1%7C&db_idx=1&px=1&section=5&ave_thresh=32&_ignoreionsscorebelow=0&report=0&_sigthreshold=0.05&_msresflags=1089&_msresflags2=2&percolate=-1&percolate_rt=0&_minpeplen=5&sessionID=all_secdisabledsession) [1114](http://10.200.41.11/mascot/cgi/peptide_view.pl?file=..%2Fdata%2F20160418%2FF080018.dat&query=1114&hit=1&index=gi%7C392616945%7Cgb%7CEIW99373.1%7C&db_idx=1&px=1&section=5&ave_thresh=32&_ignoreionsscorebelow=0&report=0&_sigthreshold=0.05&_msresflags=1089&_msresflags2=2&percolate=-1&percolate_rt=0&_minpeplen=5&sessionID=all_secdisabledsession) |
|  | [1240](http://10.200.41.11/mascot/cgi/peptide_view.pl?file=..%2Fdata%2F20160418%2FF080018.dat&query=1240&hit=1&index=gi%7C392616945%7Cgb%7CEIW99373.1%7C&db_idx=1&px=1&section=5&ave_thresh=32&_ignoreionsscorebelow=0&report=0&_sigthreshold=0.05&_msresflags=1089&_msresflags2=2&percolate=-1&percolate_rt=0&_minpeplen=5&sessionID=all_secdisabledsession) | **536.7948** | **1071.5750** | **1071.5673** | **7.27** | **0** | **28** | **0.3** | **1** | **U** | **K.NVLNGQNSVK.L** [1241](http://10.200.41.11/mascot/cgi/peptide_view.pl?file=..%2Fdata%2F20160418%2FF080018.dat&query=1241&hit=1&index=gi%7C392616945%7Cgb%7CEIW99373.1%7C&db_idx=1&px=1&section=5&ave_thresh=32&_ignoreionsscorebelow=0&report=0&_sigthreshold=0.05&_msresflags=1089&_msresflags2=2&percolate=-1&percolate_rt=0&_minpeplen=5&sessionID=all_secdisabledsession) [1242](http://10.200.41.11/mascot/cgi/peptide_view.pl?file=..%2Fdata%2F20160418%2FF080018.dat&query=1242&hit=1&index=gi%7C392616945%7Cgb%7CEIW99373.1%7C&db_idx=1&px=1&section=5&ave_thresh=32&_ignoreionsscorebelow=0&report=0&_sigthreshold=0.05&_msresflags=1089&_msresflags2=2&percolate=-1&percolate_rt=0&_minpeplen=5&sessionID=all_secdisabledsession) |
|  | [2060](http://10.200.41.11/mascot/cgi/peptide_view.pl?file=..%2Fdata%2F20160418%2FF080018.dat&query=2060&hit=1&index=gi%7C392616945%7Cgb%7CEIW99373.1%7C&db_idx=1&px=1&section=5&ave_thresh=32&_ignoreionsscorebelow=0&report=0&_sigthreshold=0.05&_msresflags=1089&_msresflags2=2&percolate=-1&percolate_rt=0&_minpeplen=5&sessionID=all_secdisabledsession) | **651.8282** | **1301.6419** | **1301.6324** | **7.35** | **0** | **64** | **6.2e-005** | **1** | **U** | **R.SEQQISQVNNR.V** [2059](http://10.200.41.11/mascot/cgi/peptide_view.pl?file=..%2Fdata%2F20160418%2FF080018.dat&query=2059&hit=1&index=gi%7C392616945%7Cgb%7CEIW99373.1%7C&db_idx=1&px=1&section=5&ave_thresh=32&_ignoreionsscorebelow=0&report=0&_sigthreshold=0.05&_msresflags=1089&_msresflags2=2&percolate=-1&percolate_rt=0&_minpeplen=5&sessionID=all_secdisabledsession) [2061](http://10.200.41.11/mascot/cgi/peptide_view.pl?file=..%2Fdata%2F20160418%2FF080018.dat&query=2061&hit=1&index=gi%7C392616945%7Cgb%7CEIW99373.1%7C&db_idx=1&px=1&section=5&ave_thresh=32&_ignoreionsscorebelow=0&report=0&_sigthreshold=0.05&_msresflags=1089&_msresflags2=2&percolate=-1&percolate_rt=0&_minpeplen=5&sessionID=all_secdisabledsession) [2062](http://10.200.41.11/mascot/cgi/peptide_view.pl?file=..%2Fdata%2F20160418%2FF080018.dat&query=2062&hit=1&index=gi%7C392616945%7Cgb%7CEIW99373.1%7C&db_idx=1&px=1&section=5&ave_thresh=32&_ignoreionsscorebelow=0&report=0&_sigthreshold=0.05&_msresflags=1089&_msresflags2=2&percolate=-1&percolate_rt=0&_minpeplen=5&sessionID=all_secdisabledsession) |
|  | [2159](http://10.200.41.11/mascot/cgi/peptide_view.pl?file=..%2Fdata%2F20160418%2FF080018.dat&query=2159&hit=1&index=gi%7C392616945%7Cgb%7CEIW99373.1%7C&db_idx=1&px=1&section=5&ave_thresh=32&_ignoreionsscorebelow=0&report=0&_sigthreshold=0.05&_msresflags=1089&_msresflags2=2&percolate=-1&percolate_rt=0&_minpeplen=5&sessionID=all_secdisabledsession) | **668.3455** | **1334.6764** | **1334.6678** | **6.45** | **0** | **60** | **0.00021** | **1** | **U** | **K.SNAGTSATESLGIK.D** [2160](http://10.200.41.11/mascot/cgi/peptide_view.pl?file=..%2Fdata%2F20160418%2FF080018.dat&query=2160&hit=1&index=gi%7C392616945%7Cgb%7CEIW99373.1%7C&db_idx=1&px=1&section=5&ave_thresh=32&_ignoreionsscorebelow=0&report=0&_sigthreshold=0.05&_msresflags=1089&_msresflags2=2&percolate=-1&percolate_rt=0&_minpeplen=5&sessionID=all_secdisabledsession) |

### Protein sequence coverage: 13%

Matched peptides shown in ***bold red***.

| **1** | MLISNVGINP | AAYLNNHSVE | NSSQTASQSV | SAKDILNSIG | ISSSKVSDLG |
| --- | --- | --- | --- | --- | --- |
| **51** | LSPTLSAPAP | GVLTQTPGTI | TSFLKASIQN | TDMNQDLNAL | ANNVTTKANE |
| **101** | VVQTQLREQQ | AEVGKFFDIS | GMSSSAVALL | AAANTLMLTL | NQADSKLSGK |
| **151** | LSLVSFDAAK | TTASSMMREG | MNALSGSISQ | SALQLGITGV | GAKLEYKGLQ |
| **201** | NERGALKHNA | AKIDKLTTES | HSIK**NVLNGQ** | **NSVKLGAEGV** | **DSLK**SLNMKK |
| **251** | TGTDATKNLN | DATLK**SNAGT** | **SATESLGIK**D | SNKQISPEHQ | AILSKRLESV |
| **301** | ESDIRLEQNT | MDMTRIDARK | MQMTGDLIMK | NSVTVGGIAG | ASGQYAATQE |
| **351** | R**SEQQISQVN** | **NR**VASTASDE | ARESSRKSTS | LIQEMLK**TME** | **SINQSK**ASAL |
| **401** | AAIAGNIRA |  |  |  |  |

| **2.** | [gi|194403331|gb|ACF63553.1|](http://10.200.41.11/mascot/cgi/protein_view.pl?file=..%2Fdata%2F20160418%2FF080018.dat&hit=gi%7C194403331%7Cgb%7CACF63553.1%7C&db_idx=1&px=1&ave_thresh=32&_ignoreionsscorebelow=0&report=0&_sigthreshold=0.05&_msresflags=1089&_msresflags2=2&percolate=-1&percolate_rt=0&_minpeplen=5&sessionID=all_secdisabledsession)    **Mass:** 10561    **Score:** 305    **Matches:** 12(5)  **Sequences:** 4(2)  **emPAI:** 1.72 |
| --- | --- |
|  | negative regulator of flagellin synthesis [Salmonella enterica subsp. enterica serovar Newport str. SL254] |

|  | **Query** | **Observed** | **Mr(expt)** | **Mr(calc)** | **ppm** | **Miss** | **Score** | **Expect** | **Rank** | **Unique** | **Peptide** |
| --- | --- | --- | --- | --- | --- | --- | --- | --- | --- | --- | --- |
|  | [912](http://10.200.41.11/mascot/cgi/peptide_view.pl?file=..%2Fdata%2F20160418%2FF080018.dat&query=912&hit=1&index=gi%7C194403331%7Cgb%7CACF63553.1%7C&db_idx=1&px=1&section=5&ave_thresh=32&_ignoreionsscorebelow=0&report=0&_sigthreshold=0.05&_msresflags=1089&_msresflags2=2&percolate=-1&percolate_rt=0&_minpeplen=5&sessionID=all_secdisabledsession) | **502.7523** | **1003.4901** | **1003.4822** | **7.85** | **0** | **34** | **0.055** | **1** | **U** | **R.ETSDTPVQK.T** [910](http://10.200.41.11/mascot/cgi/peptide_view.pl?file=..%2Fdata%2F20160418%2FF080018.dat&query=910&hit=1&index=gi%7C194403331%7Cgb%7CACF63553.1%7C&db_idx=1&px=1&section=5&ave_thresh=32&_ignoreionsscorebelow=0&report=0&_sigthreshold=0.05&_msresflags=1089&_msresflags2=2&percolate=-1&percolate_rt=0&_minpeplen=5&sessionID=all_secdisabledsession) [911](http://10.200.41.11/mascot/cgi/peptide_view.pl?file=..%2Fdata%2F20160418%2FF080018.dat&query=911&hit=1&index=gi%7C194403331%7Cgb%7CACF63553.1%7C&db_idx=1&px=1&section=5&ave_thresh=32&_ignoreionsscorebelow=0&report=0&_sigthreshold=0.05&_msresflags=1089&_msresflags2=2&percolate=-1&percolate_rt=0&_minpeplen=5&sessionID=all_secdisabledsession) [913](http://10.200.41.11/mascot/cgi/peptide_view.pl?file=..%2Fdata%2F20160418%2FF080018.dat&query=913&hit=1&index=gi%7C194403331%7Cgb%7CACF63553.1%7C&db_idx=1&px=1&section=5&ave_thresh=32&_ignoreionsscorebelow=0&report=0&_sigthreshold=0.05&_msresflags=1089&_msresflags2=2&percolate=-1&percolate_rt=0&_minpeplen=5&sessionID=all_secdisabledsession) [914](http://10.200.41.11/mascot/cgi/peptide_view.pl?file=..%2Fdata%2F20160418%2FF080018.dat&query=914&hit=1&index=gi%7C194403331%7Cgb%7CACF63553.1%7C&db_idx=1&px=1&section=5&ave_thresh=32&_ignoreionsscorebelow=0&report=0&_sigthreshold=0.05&_msresflags=1089&_msresflags2=2&percolate=-1&percolate_rt=0&_minpeplen=5&sessionID=all_secdisabledsession) |
|  | [1117](http://10.200.41.11/mascot/cgi/peptide_view.pl?file=..%2Fdata%2F20160418%2FF080018.dat&query=1117&hit=1&index=gi%7C194403331%7Cgb%7CACF63553.1%7C&db_idx=1&px=1&section=5&ave_thresh=32&_ignoreionsscorebelow=0&report=0&_sigthreshold=0.05&_msresflags=1089&_msresflags2=2&percolate=-1&percolate_rt=0&_minpeplen=5&sessionID=all_secdisabledsession) | **527.2683** | **1052.5221** | **1052.5138** | **7.85** | **0** | **30** | **0.1** | **1** | **U** | **R.EAQSYLQSK.-** [1116](http://10.200.41.11/mascot/cgi/peptide_view.pl?file=..%2Fdata%2F20160418%2FF080018.dat&query=1116&hit=1&index=gi%7C194403331%7Cgb%7CACF63553.1%7C&db_idx=1&px=1&section=5&ave_thresh=32&_ignoreionsscorebelow=0&report=0&_sigthreshold=0.05&_msresflags=1089&_msresflags2=2&percolate=-1&percolate_rt=0&_minpeplen=5&sessionID=all_secdisabledsession) |
|  | [2475](http://10.200.41.11/mascot/cgi/peptide_view.pl?file=..%2Fdata%2F20160418%2FF080018.dat&query=2475&hit=1&index=gi%7C194403331%7Cgb%7CACF63553.1%7C&db_idx=1&px=1&section=5&ave_thresh=32&_ignoreionsscorebelow=0&report=0&_sigthreshold=0.05&_msresflags=1089&_msresflags2=2&percolate=-1&percolate_rt=0&_minpeplen=5&sessionID=all_secdisabledsession) | **761.3605** | **1520.7064** | **1520.6963** | **6.64** | **0** | **62** | **7.4e-005** | **1** | **U** | **K.LMQPGVSDINMER.V** [2474](http://10.200.41.11/mascot/cgi/peptide_view.pl?file=..%2Fdata%2F20160418%2FF080018.dat&query=2474&hit=1&index=gi%7C194403331%7Cgb%7CACF63553.1%7C&db_idx=1&px=1&section=5&ave_thresh=32&_ignoreionsscorebelow=0&report=0&_sigthreshold=0.05&_msresflags=1089&_msresflags2=2&percolate=-1&percolate_rt=0&_minpeplen=5&sessionID=all_secdisabledsession) [2476](http://10.200.41.11/mascot/cgi/peptide_view.pl?file=..%2Fdata%2F20160418%2FF080018.dat&query=2476&hit=1&index=gi%7C194403331%7Cgb%7CACF63553.1%7C&db_idx=1&px=1&section=5&ave_thresh=32&_ignoreionsscorebelow=0&report=0&_sigthreshold=0.05&_msresflags=1089&_msresflags2=2&percolate=-1&percolate_rt=0&_minpeplen=5&sessionID=all_secdisabledsession) |
|  | [2583](http://10.200.41.11/mascot/cgi/peptide_view.pl?file=..%2Fdata%2F20160418%2FF080018.dat&query=2583&hit=1&index=gi%7C194403331%7Cgb%7CACF63553.1%7C&db_idx=1&px=1&section=5&ave_thresh=32&_ignoreionsscorebelow=0&report=0&_sigthreshold=0.05&_msresflags=1089&_msresflags2=2&percolate=-1&percolate_rt=0&_minpeplen=5&sessionID=all_secdisabledsession) | **804.9128** | **1607.8110** | **1607.8002** | **6.70** | **0** | **98** | **2.8e-008** | **1** | **U** | **K.TSAATSASVTLSDAQAK.L** [2584](http://10.200.41.11/mascot/cgi/peptide_view.pl?file=..%2Fdata%2F20160418%2FF080018.dat&query=2584&hit=1&index=gi%7C194403331%7Cgb%7CACF63553.1%7C&db_idx=1&px=1&section=5&ave_thresh=32&_ignoreionsscorebelow=0&report=0&_sigthreshold=0.05&_msresflags=1089&_msresflags2=2&percolate=-1&percolate_rt=0&_minpeplen=5&sessionID=all_secdisabledsession) |

### Protein sequence coverage: 49%

Matched peptides shown in ***bold red***.

| **1** | MSIDRTSPLK | PVSTVQTR**ET** | **SDTPVQK**TRQ | EK**TSAATSAS** | **VTLSDAQAKL** |
| --- | --- | --- | --- | --- | --- |
| **51** | **MQPGVSDINM** | **ER**VEALKTAI | RNGELKMDTG | KIADSLIR**EA** | **QSYLQSK** |

| **3.** | [gi|50830890|gb|AAT81610.1|](http://10.200.41.11/mascot/cgi/protein_view.pl?file=..%2Fdata%2F20160418%2FF080018.dat&hit=gi%7C50830890%7Cgb%7CAAT81610.1%7C&db_idx=1&px=1&ave_thresh=32&_ignoreionsscorebelow=0&report=0&_sigthreshold=0.05&_msresflags=1089&_msresflags2=2&percolate=-1&percolate_rt=0&_minpeplen=5&sessionID=all_secdisabledsession)    **Mass:** 52223    **Score:** 222    **Matches:** 12(7)  **Sequences:** 5(3)  **emPAI:** 0.37 |
| --- | --- |
|  | phase 1 flagellin [Salmonella enterica subsp. enterica serovar Newport] |

|  | **Query** | **Observed** | **Mr(expt)** | **Mr(calc)** | **ppm** | **Miss** | **Score** | **Expect** | **Rank** | **Unique** | **Peptide** |
| --- | --- | --- | --- | --- | --- | --- | --- | --- | --- | --- | --- |
|  | [1379](http://10.200.41.11/mascot/cgi/peptide_view.pl?file=..%2Fdata%2F20160418%2FF080018.dat&query=1379&hit=1&index=gi%7C50830890%7Cgb%7CAAT81610.1%7C&db_idx=1&px=1&section=5&ave_thresh=32&_ignoreionsscorebelow=0&report=0&_sigthreshold=0.05&_msresflags=1089&_msresflags2=2&percolate=-1&percolate_rt=0&_minpeplen=5&sessionID=all_secdisabledsession) | **551.2722** | **1100.5299** | **1100.5210** | **8.05** | **0** | **67** | **1.5e-005** | **1** | **U** | **K.DDAAGQAIANR.F** [1378](http://10.200.41.11/mascot/cgi/peptide_view.pl?file=..%2Fdata%2F20160418%2FF080018.dat&query=1378&hit=1&index=gi%7C50830890%7Cgb%7CAAT81610.1%7C&db_idx=1&px=1&section=5&ave_thresh=32&_ignoreionsscorebelow=0&report=0&_sigthreshold=0.05&_msresflags=1089&_msresflags2=2&percolate=-1&percolate_rt=0&_minpeplen=5&sessionID=all_secdisabledsession) |
|  | [1491](http://10.200.41.11/mascot/cgi/peptide_view.pl?file=..%2Fdata%2F20160418%2FF080018.dat&query=1491&hit=1&index=gi%7C50830890%7Cgb%7CAAT81610.1%7C&db_idx=1&px=1&section=5&ave_thresh=32&_ignoreionsscorebelow=0&report=0&_sigthreshold=0.05&_msresflags=1089&_msresflags2=2&percolate=-1&percolate_rt=0&_minpeplen=5&sessionID=all_secdisabledsession) | **566.8058** | **1131.5970** | **1131.5884** | **7.64** | **0** | **46** | **0.0053** | **1** | **U** | **K.SQSALGTAIER.L** [1489](http://10.200.41.11/mascot/cgi/peptide_view.pl?file=..%2Fdata%2F20160418%2FF080018.dat&query=1489&hit=1&index=gi%7C50830890%7Cgb%7CAAT81610.1%7C&db_idx=1&px=1&section=5&ave_thresh=32&_ignoreionsscorebelow=0&report=0&_sigthreshold=0.05&_msresflags=1089&_msresflags2=2&percolate=-1&percolate_rt=0&_minpeplen=5&sessionID=all_secdisabledsession) [1490](http://10.200.41.11/mascot/cgi/peptide_view.pl?file=..%2Fdata%2F20160418%2FF080018.dat&query=1490&hit=1&index=gi%7C50830890%7Cgb%7CAAT81610.1%7C&db_idx=1&px=1&section=5&ave_thresh=32&_ignoreionsscorebelow=0&report=0&_sigthreshold=0.05&_msresflags=1089&_msresflags2=2&percolate=-1&percolate_rt=0&_minpeplen=5&sessionID=all_secdisabledsession) |
|  | [1592](http://10.200.41.11/mascot/cgi/peptide_view.pl?file=..%2Fdata%2F20160418%2FF080018.dat&query=1592&hit=1&index=gi%7C50830890%7Cgb%7CAAT81610.1%7C&db_idx=1&px=1&section=5&ave_thresh=32&_ignoreionsscorebelow=0&report=0&_sigthreshold=0.05&_msresflags=1089&_msresflags2=2&percolate=-1&percolate_rt=0&_minpeplen=5&sessionID=all_secdisabledsession) | **582.8082** | **1163.6018** | **1163.5935** | **7.11** | **0** | **47** | **0.0041** | **1** | **U** | **R.VSGQTQFNGVK.V** [1591](http://10.200.41.11/mascot/cgi/peptide_view.pl?file=..%2Fdata%2F20160418%2FF080018.dat&query=1591&hit=1&index=gi%7C50830890%7Cgb%7CAAT81610.1%7C&db_idx=1&px=1&section=5&ave_thresh=32&_ignoreionsscorebelow=0&report=0&_sigthreshold=0.05&_msresflags=1089&_msresflags2=2&percolate=-1&percolate_rt=0&_minpeplen=5&sessionID=all_secdisabledsession) |
|  | [2585](http://10.200.41.11/mascot/cgi/peptide_view.pl?file=..%2Fdata%2F20160418%2FF080018.dat&query=2585&hit=1&index=gi%7C50830890%7Cgb%7CAAT81610.1%7C&db_idx=1&px=1&section=5&ave_thresh=32&_ignoreionsscorebelow=0&report=0&_sigthreshold=0.05&_msresflags=1089&_msresflags2=2&percolate=-1&percolate_rt=0&_minpeplen=5&sessionID=all_secdisabledsession) | **538.9485** | **1613.8236** | **1613.8121** | **7.14** | **1** | **23** | **0.86** | **1** | **U** | **R.INSAKDDAAGQAIANR.F** |
|  | [2590](http://10.200.41.11/mascot/cgi/peptide_view.pl?file=..%2Fdata%2F20160418%2FF080018.dat&query=2590&hit=1&index=gi%7C50830890%7Cgb%7CAAT81610.1%7C&db_idx=1&px=1&section=5&ave_thresh=32&_ignoreionsscorebelow=0&report=0&_sigthreshold=0.05&_msresflags=1089&_msresflags2=2&percolate=-1&percolate_rt=0&_minpeplen=5&sessionID=all_secdisabledsession) | **539.9374** | **1616.7905** | **1616.8006** | **-6.22** | **1** | **1** | **1.2e+002** | **1** | **U** | **K.KALEDGGVSNADATAAK.L** [2587](http://10.200.41.11/mascot/cgi/peptide_view.pl?file=..%2Fdata%2F20160418%2FF080018.dat&query=2587&hit=1&index=gi%7C50830890%7Cgb%7CAAT81610.1%7C&db_idx=1&px=1&section=5&ave_thresh=32&_ignoreionsscorebelow=0&report=0&_sigthreshold=0.05&_msresflags=1089&_msresflags2=2&percolate=-1&percolate_rt=0&_minpeplen=5&sessionID=all_secdisabledsession) [2588](http://10.200.41.11/mascot/cgi/peptide_view.pl?file=..%2Fdata%2F20160418%2FF080018.dat&query=2588&hit=1&index=gi%7C50830890%7Cgb%7CAAT81610.1%7C&db_idx=1&px=1&section=5&ave_thresh=32&_ignoreionsscorebelow=0&report=0&_sigthreshold=0.05&_msresflags=1089&_msresflags2=2&percolate=-1&percolate_rt=0&_minpeplen=5&sessionID=all_secdisabledsession) [2589](http://10.200.41.11/mascot/cgi/peptide_view.pl?file=..%2Fdata%2F20160418%2FF080018.dat&query=2589&hit=2&index=gi%7C50830890%7Cgb%7CAAT81610.1%7C&db_idx=1&px=1&section=5&ave_thresh=32&_ignoreionsscorebelow=0&report=0&_sigthreshold=0.05&_msresflags=1089&_msresflags2=2&percolate=-1&percolate_rt=0&_minpeplen=5&sessionID=all_secdisabledsession) |

### Protein sequence coverage: 10%

Matched peptides shown in ***bold red***.

| **1** | MAQVINTNSL | SLLTQNNLNK | **SQSALGTAIE** | **R**LSSGLR**INS** | **AKDDAAGQAI** |
| --- | --- | --- | --- | --- | --- |
| **51** | **ANR**FTANIKG | LTQASRNAND | GISIAQTTEG | ALNEINNNLQ | RVRELAVQSA |
| **101** | NSTNSQSDLD | SIQAEITQRL | NEIDR**VSGQT** | **QFNGVK**VLAQ | DNTLTIQVGA |
| **151** | NDGETIDIDL | KQINSQTLGL | DTLNVQKAYD | VSATAAMDPK | SFTDGTKNLT |
| **201** | APDATAIKAA | LGNPAATGDS | LSATLSFKDG | KYYATVAGYT | NAADTSKNGK |
| **251** | YEVNVDSATG | AVTFNAAPTK | ATVTGDTTVT | KVQVNAPVAV | STDVK**KALED** |
| **301** | **GGVSNADATA** | **AK**LVKMSYTD | KNGKSIDGGY | ALEAGGKYYA | ATYDEGTGKI |
| **351** | TANVTTYTDS | TGVTKTAANQ | LGGVDGKTEV | VTIDGKTYNA | SKAAGHDFKA |
| **401** | QPELAEAAAK | TTENPLAKID | AALAQVDALR | SDLGAVQNRF | NSAITNLGNT |
| **451** | VNNLSEARSR | IEDSDYATEV | SNMSRAQILQ | QAGTSVLAQA | NQVPQNVLSL |
| **501** | LR |  |  |  |  |

| **5.** | [gi|194402702|gb|ACF62924.1|](http://10.200.41.11/mascot/cgi/protein_view.pl?file=..%2Fdata%2F20160418%2FF080018.dat&hit=gi%7C194402702%7Cgb%7CACF62924.1%7C&db_idx=1&px=1&ave_thresh=32&_ignoreionsscorebelow=0&report=0&_sigthreshold=0.05&_msresflags=1089&_msresflags2=2&percolate=-1&percolate_rt=0&_minpeplen=5&sessionID=all_secdisabledsession)    **Mass:** 72333    **Score:** 123    **Matches:** 7(3)  **Sequences:** 3(1)  **emPAI:** 0.08 |
| --- | --- |
|  | cell invasion protein SipA [Salmonella enterica subsp. enterica serovar Newport str. SL254] |

|  | **Query** | **Observed** | **Mr(expt)** | **Mr(calc)** | **ppm** | **Miss** | **Score** | **Expect** | **Rank** | **Unique** | **Peptide** |
| --- | --- | --- | --- | --- | --- | --- | --- | --- | --- | --- | --- |
|  | [769](http://10.200.41.11/mascot/cgi/peptide_view.pl?file=..%2Fdata%2F20160418%2FF080018.dat&query=769&hit=1&index=gi%7C194402702%7Cgb%7CACF62924.1%7C&db_idx=1&px=1&section=5&ave_thresh=32&_ignoreionsscorebelow=0&report=0&_sigthreshold=0.05&_msresflags=1089&_msresflags2=2&percolate=-1&percolate_rt=0&_minpeplen=5&sessionID=all_secdisabledsession) | **490.7472** | **979.4798** | **979.4723** | **7.62** | **0** | **35** | **0.061** | **1** | **U** | **R.TFIDNSQR.N** [768](http://10.200.41.11/mascot/cgi/peptide_view.pl?file=..%2Fdata%2F20160418%2FF080018.dat&query=768&hit=1&index=gi%7C194402702%7Cgb%7CACF62924.1%7C&db_idx=1&px=1&section=5&ave_thresh=32&_ignoreionsscorebelow=0&report=0&_sigthreshold=0.05&_msresflags=1089&_msresflags2=2&percolate=-1&percolate_rt=0&_minpeplen=5&sessionID=all_secdisabledsession) |
|  | [1579](http://10.200.41.11/mascot/cgi/peptide_view.pl?file=..%2Fdata%2F20160418%2FF080018.dat&query=1579&hit=2&index=gi%7C194402702%7Cgb%7CACF62924.1%7C&db_idx=1&px=1&section=5&ave_thresh=32&_ignoreionsscorebelow=0&report=0&_sigthreshold=0.05&_msresflags=1089&_msresflags2=2&percolate=-1&percolate_rt=0&_minpeplen=5&sessionID=all_secdisabledsession) | **577.7966** | **1153.5787** | **1153.5954** | **-14.45** | **0** | **5** | **32** | **2** | **U** | **K.AIMEFAGLFR.S** [1580](http://10.200.41.11/mascot/cgi/peptide_view.pl?file=..%2Fdata%2F20160418%2FF080018.dat&query=1580&hit=2&index=gi%7C194402702%7Cgb%7CACF62924.1%7C&db_idx=1&px=1&section=5&ave_thresh=32&_ignoreionsscorebelow=0&report=0&_sigthreshold=0.05&_msresflags=1089&_msresflags2=2&percolate=-1&percolate_rt=0&_minpeplen=5&sessionID=all_secdisabledsession) |
|  | [1658](http://10.200.41.11/mascot/cgi/peptide_view.pl?file=..%2Fdata%2F20160418%2FF080018.dat&query=1658&hit=1&index=gi%7C194402702%7Cgb%7CACF62924.1%7C&db_idx=1&px=1&section=5&ave_thresh=32&_ignoreionsscorebelow=0&report=0&_sigthreshold=0.05&_msresflags=1089&_msresflags2=2&percolate=-1&percolate_rt=0&_minpeplen=5&sessionID=all_secdisabledsession) | **588.3088** | **1174.6030** | **1174.5942** | **7.47** | **0** | **58** | **0.00032** | **1** | **U** | **K.LTQEQGTSVGR.E** [1657](http://10.200.41.11/mascot/cgi/peptide_view.pl?file=..%2Fdata%2F20160418%2FF080018.dat&query=1657&hit=1&index=gi%7C194402702%7Cgb%7CACF62924.1%7C&db_idx=1&px=1&section=5&ave_thresh=32&_ignoreionsscorebelow=0&report=0&_sigthreshold=0.05&_msresflags=1089&_msresflags2=2&percolate=-1&percolate_rt=0&_minpeplen=5&sessionID=all_secdisabledsession) [1659](http://10.200.41.11/mascot/cgi/peptide_view.pl?file=..%2Fdata%2F20160418%2FF080018.dat&query=1659&hit=1&index=gi%7C194402702%7Cgb%7CACF62924.1%7C&db_idx=1&px=1&section=5&ave_thresh=32&_ignoreionsscorebelow=0&report=0&_sigthreshold=0.05&_msresflags=1089&_msresflags2=2&percolate=-1&percolate_rt=0&_minpeplen=5&sessionID=all_secdisabledsession) |

### Protein sequence coverage: 4%

Matched peptides shown in ***bold red***.

| **1** | MQTEIKTQAT | NLAANLSAVR | ESATTTLSGE | IKGPQLEDFP | ALIKQASLDA |
| --- | --- | --- | --- | --- | --- |
| **51** | LFKCGKDAEA | LKEVFTNSNN | VAGKK**AIMEF** | **AGLFR**SALNA | TSDSPEAKTL |
| **101** | LMKVGAEYTA | QIIKDGLKEK | SAFGPWLPET | KKAEAKLENL | EKQLLDIIKN |
| **151** | NTGGELSKLS | TNLVMQEVMP | YIASCIEHNF | GCTLDPLTRS | NLTHLVDKAA |
| **201** | AKAVEALDMC | HQK**LTQEQGT** | **SVGR**EARHLE | MQTLIPLLLR | NVFAQIPADK |
| **251** | LPDPKIPEPA | AGPVPDGGKK | AEPTGININI | NIDSSNHSVD | NSKHINNSRS |
| **301** | HVDNSQRHID | NSNHDNSRKT | IDNSR**TFIDN** | **SQR**NGESHHS | TNSSNVSHSH |
| **351** | SRVDSTTHQT | ETAHSASTGA | IDHGIAGKID | VTAHATAEAV | TNASSESKDG |
| **401** | KVVTSEKGTT | GETTSFDEVD | GVTSKSIIGK | PVQATVHGVD | DNKQQSQTAE |
| **451** | IVNVKPLASQ | LAGVENVKTD | TLQSDTTVIT | GNKAGTTDND | NSQTDKTGPF |
| **501** | SGLKFKQNSF | LSTVPSVTNM | HSMHFDARET | FLGVIRKALE | PDTSTPFPVR |
| **551** | RAFDGLRAEI | LPNDTIKSAA | LKAQCSDIDK | HPELKAKMET | LKEVITHHPQ |
| **601** | KEKLAEIALQ | FAREAGLTRL | KGETDYVLSN | VLDGLIGDGS | WRAGPAYESY |
| **651** | LNKPGVDRVI | TTVDGLHMQR |  |  |  |

| **9.** | [gi|194403640|gb|ACF63862.1|](http://10.200.41.11/mascot/cgi/protein_view.pl?file=..%2Fdata%2F20160418%2FF080018.dat&hit=gi%7C194403640%7Cgb%7CACF63862.1%7C&db_idx=1&px=1&ave_thresh=32&_ignoreionsscorebelow=0&report=0&_sigthreshold=0.05&_msresflags=1089&_msresflags2=2&percolate=-1&percolate_rt=0&_minpeplen=5&sessionID=all_secdisabledsession)    **Mass:** 62382    **Score:** 53     **Matches:** 4(1)  **Sequences:** 2(1)  **emPAI:** 0.09 |
| --- | --- |
|  | cell invasion protein SipB [Salmonella enterica subsp. enterica serovar Newport str. SL254] |

|  | **Query** | **Observed** | **Mr(expt)** | **Mr(calc)** | **ppm** | **Miss** | **Score** | **Expect** | **Rank** | **Unique** | **Peptide** |
| --- | --- | --- | --- | --- | --- | --- | --- | --- | --- | --- | --- |
|  | [513](http://10.200.41.11/mascot/cgi/peptide_view.pl?file=..%2Fdata%2F20160418%2FF080018.dat&query=513&hit=1&index=gi%7C194403640%7Cgb%7CACF63862.1%7C&db_idx=1&px=1&section=5&ave_thresh=32&_ignoreionsscorebelow=0&report=0&_sigthreshold=0.05&_msresflags=1089&_msresflags2=2&percolate=-1&percolate_rt=0&_minpeplen=5&sessionID=all_secdisabledsession) | **461.7263** | **921.4381** | **921.4304** | **8.30** | **0** | **45** | **0.0032** | **1** | **U** | **R.SGYTQNPR.L** [512](http://10.200.41.11/mascot/cgi/peptide_view.pl?file=..%2Fdata%2F20160418%2FF080018.dat&query=512&hit=1&index=gi%7C194403640%7Cgb%7CACF63862.1%7C&db_idx=1&px=1&section=5&ave_thresh=32&_ignoreionsscorebelow=0&report=0&_sigthreshold=0.05&_msresflags=1089&_msresflags2=2&percolate=-1&percolate_rt=0&_minpeplen=5&sessionID=all_secdisabledsession) |
|  | [768](http://10.200.41.11/mascot/cgi/peptide_view.pl?file=..%2Fdata%2F20160418%2FF080018.dat&query=768&hit=5&index=gi%7C194403640%7Cgb%7CACF63862.1%7C&db_idx=1&px=1&section=5&ave_thresh=32&_ignoreionsscorebelow=0&report=0&_sigthreshold=0.05&_msresflags=1089&_msresflags2=2&percolate=-1&percolate_rt=0&_minpeplen=5&sessionID=all_secdisabledsession) | 490.7469 | 979.4792 | 979.4909 | -11.96 | 0 | 2 | 1.3e+002 | 5 | U | K.LFTQGMQR.I [769](http://10.200.41.11/mascot/cgi/peptide_view.pl?file=..%2Fdata%2F20160418%2FF080018.dat&query=769&hit=5&index=gi%7C194403640%7Cgb%7CACF63862.1%7C&db_idx=1&px=1&section=5&ave_thresh=32&_ignoreionsscorebelow=0&report=0&_sigthreshold=0.05&_msresflags=1089&_msresflags2=2&percolate=-1&percolate_rt=0&_minpeplen=5&sessionID=all_secdisabledsession) |

### Protein sequence coverage: 2%

Matched peptides shown in ***bold red***.

| **1** | MVNDASSISR | **SGYTQNPR**LA | EAAFEGVRKN | TDFLKAADKA | FKDVVATKAG |
| --- | --- | --- | --- | --- | --- |
| **51** | DLKAGTKSGE | SAINTVGLKP | PTDAAREKLS | SEGQLTLLLG | KLMTLLGDVS |
| **101** | LSQLESRLAV | WQAMIESQKE | MGIQVSKEFQ | TALGEAQEAT | DLYEASIKKT |
| **151** | DTAKSVYDAA | AKKLTQAQNK | LQSLDPADPG | YAQAEAAVEQ | AGKEATEAKE |
| **201** | ALDKATDATV | KAGTDAKAKA | EKADNILTKF | QGTANAASQN | QVSQGEQDNL |
| **251** | SNVARLTMLM | AMFIEIVGKN | TEESLQNDLA | LFNALQEGRQ | AEMEKKSAEF |
| **301** | QEETRKAEET | NRIMGCIGKV | LGALLTIVSV | VAAVFTGGAS | LALAAVGLAV |
| **351** | MVADEIVKAA | TGVSFIQQAL | NPIMEHVLKP | LMELIGKAIT | KALEGLGVDK |
| **401** | KTAEMAGSIV | GAIVAAIAMV | AVIVVVAVVG | KGAAAKLGNA | LSKMMGETIK |
| **451** | KLVPNVLKQL | AQNGSK**LFTQ** | **GMQR**ITSGLG | NVGSKMGLQT | NALSKELVGN |
| **501** | TLNKVALGME | VTNTAAQSAG | GVAEGVFIKN | ASEALADFML | ARFAMDQIQQ |
| **551** | WLKQSVEIFG | ENQKVTAELQ | KAMSSAVQQN | ADASRFILRQ | SRA |

**********************************************20 Hour Re-Digest Control***********************************************

**1^st^ technical replicate**

**Enzyme : Trypsin**

**Variable modifications :** [**Oxidation (M)**](http://10.200.41.11/mascot/cgi/client.pl?modification&mod_name=Oxidation%20%28M%29&file=..%2Fdata%2F20160414%2FF079944.dat)

**Mass values : Monoisotopic**

**Protein Mass : Unrestricted**

**Peptide Mass Tolerance : ± 20 ppm**

**Fragment Mass Tolerance: ± 0.4 Da**

**Max Missed Cleavages : 3**

**Instrument type : Default**

**Number of queries : 3187**

**No identfications.**

*******************************************************************************************************************************************************

**2^nd^ technical replicate**

**Enzyme : Trypsin**

**Variable modifications :** [**Oxidation (M)**](http://10.200.41.11/mascot/cgi/client.pl?modification&mod_name=Oxidation%20%28M%29&file=..%2Fdata%2F20160414%2FF079946.dat)

**Mass values : Monoisotopic**

**Protein Mass : Unrestricted**

**Peptide Mass Tolerance : ± 20 ppm**

**Fragment Mass Tolerance: ± 0.4 Da**

**Max Missed Cleavages : 3**

**Instrument type : Default**

**Number of queries : 3065**

| Protein hits           : | [**gi|392765192|gb|EJA21981.1|**](http://10.200.41.11/mascot/cgi/master_results.pl?file=..%2Fdata%2F20160414%2FF079946.dat#Hit1) | phage immunity repressor protein [Salmonella enterica subsp. enterica serovar Newport str. CVM 19449] |
| --- | --- | --- |

| 1. | [gi|392765192|gb|EJA21981.1|](http://10.200.41.11/mascot/cgi/protein_view.pl?file=..%2Fdata%2F20160414%2FF079946.dat&hit=gi%7C392765192%7Cgb%7CEJA21981.1%7C&db_idx=1&px=1&ave_thresh=32&_ignoreionsscorebelow=0&report=0&_sigthreshold=0.05&_msresflags=1089&_msresflags2=2&percolate=-1&percolate_rt=0&_minpeplen=5&sessionID=all_secdisabledsession)    Mass: 21776    Score: 21     Matches: 10(0)  Sequences: 1(0) |
| --- | --- |
|  | phage immunity repressor protein [Salmonella enterica subsp. enterica serovar Newport str. CVM 19449] |

|  | Query | Observed | Mr(expt) | Mr(calc) | ppm | Miss | Score | Expect | Rank | Unique | Peptide |
| --- | --- | --- | --- | --- | --- | --- | --- | --- | --- | --- | --- |
|  | [95](http://10.200.41.11/mascot/cgi/peptide_view.pl?file=..%2Fdata%2F20160414%2FF079946.dat&query=95&hit=1&index=gi%7C392765192%7Cgb%7CEJA21981.1%7C&db_idx=1&px=1&section=5&ave_thresh=32&_ignoreionsscorebelow=0&report=0&_sigthreshold=0.05&_msresflags=1089&_msresflags2=2&percolate=-1&percolate_rt=0&_minpeplen=5&sessionID=all_secdisabledsession) | 415.7460 | 829.4775 | 829.4770 | 0.65 | 1 | 21 | 3.1 | 1 | U | K.VAAKSAAGR.R [87](http://10.200.41.11/mascot/cgi/peptide_view.pl?file=..%2Fdata%2F20160414%2FF079946.dat&query=87&hit=8&index=gi%7C392765192%7Cgb%7CEJA21981.1%7C&db_idx=1&px=1&section=5&ave_thresh=32&_ignoreionsscorebelow=0&report=0&_sigthreshold=0.05&_msresflags=1089&_msresflags2=2&percolate=-1&percolate_rt=0&_minpeplen=5&sessionID=all_secdisabledsession) [88](http://10.200.41.11/mascot/cgi/peptide_view.pl?file=..%2Fdata%2F20160414%2FF079946.dat&query=88&hit=5&index=gi%7C392765192%7Cgb%7CEJA21981.1%7C&db_idx=1&px=1&section=5&ave_thresh=32&_ignoreionsscorebelow=0&report=0&_sigthreshold=0.05&_msresflags=1089&_msresflags2=2&percolate=-1&percolate_rt=0&_minpeplen=5&sessionID=all_secdisabledsession) [89](http://10.200.41.11/mascot/cgi/peptide_view.pl?file=..%2Fdata%2F20160414%2FF079946.dat&query=89&hit=9&index=gi%7C392765192%7Cgb%7CEJA21981.1%7C&db_idx=1&px=1&section=5&ave_thresh=32&_ignoreionsscorebelow=0&report=0&_sigthreshold=0.05&_msresflags=1089&_msresflags2=2&percolate=-1&percolate_rt=0&_minpeplen=5&sessionID=all_secdisabledsession) [90](http://10.200.41.11/mascot/cgi/peptide_view.pl?file=..%2Fdata%2F20160414%2FF079946.dat&query=90&hit=4&index=gi%7C392765192%7Cgb%7CEJA21981.1%7C&db_idx=1&px=1&section=5&ave_thresh=32&_ignoreionsscorebelow=0&report=0&_sigthreshold=0.05&_msresflags=1089&_msresflags2=2&percolate=-1&percolate_rt=0&_minpeplen=5&sessionID=all_secdisabledsession) [91](http://10.200.41.11/mascot/cgi/peptide_view.pl?file=..%2Fdata%2F20160414%2FF079946.dat&query=91&hit=4&index=gi%7C392765192%7Cgb%7CEJA21981.1%7C&db_idx=1&px=1&section=5&ave_thresh=32&_ignoreionsscorebelow=0&report=0&_sigthreshold=0.05&_msresflags=1089&_msresflags2=2&percolate=-1&percolate_rt=0&_minpeplen=5&sessionID=all_secdisabledsession) [92](http://10.200.41.11/mascot/cgi/peptide_view.pl?file=..%2Fdata%2F20160414%2FF079946.dat&query=92&hit=4&index=gi%7C392765192%7Cgb%7CEJA21981.1%7C&db_idx=1&px=1&section=5&ave_thresh=32&_ignoreionsscorebelow=0&report=0&_sigthreshold=0.05&_msresflags=1089&_msresflags2=2&percolate=-1&percolate_rt=0&_minpeplen=5&sessionID=all_secdisabledsession) [93](http://10.200.41.11/mascot/cgi/peptide_view.pl?file=..%2Fdata%2F20160414%2FF079946.dat&query=93&hit=2&index=gi%7C392765192%7Cgb%7CEJA21981.1%7C&db_idx=1&px=1&section=5&ave_thresh=32&_ignoreionsscorebelow=0&report=0&_sigthreshold=0.05&_msresflags=1089&_msresflags2=2&percolate=-1&percolate_rt=0&_minpeplen=5&sessionID=all_secdisabledsession) [94](http://10.200.41.11/mascot/cgi/peptide_view.pl?file=..%2Fdata%2F20160414%2FF079946.dat&query=94&hit=2&index=gi%7C392765192%7Cgb%7CEJA21981.1%7C&db_idx=1&px=1&section=5&ave_thresh=32&_ignoreionsscorebelow=0&report=0&_sigthreshold=0.05&_msresflags=1089&_msresflags2=2&percolate=-1&percolate_rt=0&_minpeplen=5&sessionID=all_secdisabledsession) [96](http://10.200.41.11/mascot/cgi/peptide_view.pl?file=..%2Fdata%2F20160414%2FF079946.dat&query=96&hit=1&index=gi%7C392765192%7Cgb%7CEJA21981.1%7C&db_idx=1&px=1&section=5&ave_thresh=32&_ignoreionsscorebelow=0&report=0&_sigthreshold=0.05&_msresflags=1089&_msresflags2=2&percolate=-1&percolate_rt=0&_minpeplen=5&sessionID=all_secdisabledsession) |

*******************************************************************************************************************************************************

**3^rd^ technical replicate**

**Enzyme : Trypsin**

**Variable modifications :** [**Oxidation (M)**](http://10.200.41.11/mascot/cgi/client.pl?modification&mod_name=Oxidation%20%28M%29&file=..%2Fdata%2F20160414%2FF079948.dat)

**Mass values : Monoisotopic**

**Protein Mass : Unrestricted**

**Peptide Mass Tolerance : ± 20 ppm**

**Fragment Mass Tolerance: ± 0.4 Da**

**Max Missed Cleavages : 3**

**Instrument type : Default**

**Number of queries : 3251**

| Protein hits           : | [**gi|195630380|gb|EDX49006.1|**](http://10.200.41.11/mascot/cgi/master_results.pl?file=..%2Fdata%2F20160414%2FF079948.dat#Hit1) | protein RecT [Salmonella enterica subsp. enterica serovar Newport str. SL317] |
| --- | --- | --- |

******************************************************************DAY 2***********************************************************************

**********************************************************15 minute SS****************************************************************

**1^st^ technical replicate**

**Enzyme : Trypsin**

**Variable modifications :** [**Oxidation (M)**](http://10.200.41.11/mascot/cgi/client.pl?modification&mod_name=Oxidation%20%28M%29&file=..%2Fdata%2F20160609%2FF081283.dat)

**Mass values : Monoisotopic**

**Protein Mass : Unrestricted**

**Peptide Mass Tolerance : ± 20 ppm**

**Fragment Mass Tolerance: ± 0.4 Da**

**Max Missed Cleavages : 3**

**Instrument type : Default**

**Number of queries : 2828**

| **Protein hits           :** | [**gi|50830890|gb|AAT81610.1|**](http://10.200.41.11/mascot/cgi/master_results.pl?file=..%2Fdata%2F20160609%2FF081283.dat#Hit1) | phase 1 flagellin [Salmonella enterica subsp. enterica serovar Newport] |
| --- | --- | --- |
|  | [**gi|194402702|gb|ACF62924.1|**](http://10.200.41.11/mascot/cgi/master_results.pl?file=..%2Fdata%2F20160609%2FF081283.dat#Hit2) | cell invasion protein SipA [Salmonella enterica subsp. enterica serovar Newport str. SL254] |
|  | [**gi|392616945|gb|EIW99373.1|**](http://10.200.41.11/mascot/cgi/master_results.pl?file=..%2Fdata%2F20160609%2FF081283.dat#Hit3) | pathogenicity island 1 effector protein SipC [Salmonella enterica subsp. enterica serovar Newport str. Levine 15] |
|  | [**gi|392616944|gb|EIW99372.1|**](http://10.200.41.11/mascot/cgi/master_results.pl?file=..%2Fdata%2F20160609%2FF081283.dat#Hit4) | cell invasion protein SipD [Salmonella enterica subsp. enterica serovar Newport str. Levine 15] |
|  | [**gi|194403331|gb|ACF63553.1|**](http://10.200.41.11/mascot/cgi/master_results.pl?file=..%2Fdata%2F20160609%2FF081283.dat#Hit5) | negative regulator of flagellin synthesis [Salmonella enterica subsp. enterica serovar Newport str. SL254] |
|  | [**gi|194401173|gb|ACF61395.1|**](http://10.200.41.11/mascot/cgi/master_results.pl?file=..%2Fdata%2F20160609%2FF081283.dat#Hit6) | flagellar hook-associated protein 2 [Salmonella enterica subsp. enterica serovar Newport str. SL254] |
|  | [**gi|194403640|gb|ACF63862.1|**](http://10.200.41.11/mascot/cgi/master_results.pl?file=..%2Fdata%2F20160609%2FF081283.dat#Hit7) | cell invasion protein SipB [Salmonella enterica subsp. enterica serovar Newport str. SL254] |
|  | [**gi|194404381|gb|ACF64603.1|**](http://10.200.41.11/mascot/cgi/master_results.pl?file=..%2Fdata%2F20160609%2FF081283.dat#Hit8) | DNA-binding protein HU-alpha [Salmonella enterica subsp. enterica serovar Newport str. SL254] |
|  | [**gi|194401698|gb|ACF61920.1|**](http://10.200.41.11/mascot/cgi/master_results.pl?file=..%2Fdata%2F20160609%2FF081283.dat#Hit9) | translation elongation factor Tu [Salmonella enterica subsp. enterica serovar Newport str. SL254] |
|  | [**gi|194404227|gb|ACF64449.1|**](http://10.200.41.11/mascot/cgi/master_results.pl?file=..%2Fdata%2F20160609%2FF081283.dat#Hit10) | ribosomal protein S5 [Salmonella enterica subsp. enterica serovar Newport str. SL254] |
|  | [**gi|194401878|gb|ACF62100.1|**](http://10.200.41.11/mascot/cgi/master_results.pl?file=..%2Fdata%2F20160609%2FF081283.dat#Hit11) | ribosomal protein L11 [Salmonella enterica subsp. enterica serovar Newport str. SL254] |
|  | [**gi|194401176|gb|ACF61398.1|**](http://10.200.41.11/mascot/cgi/master_results.pl?file=..%2Fdata%2F20160609%2FF081283.dat#Hit12) | ribosomal protein L7/L12 [Salmonella enterica subsp. enterica serovar Newport str. SL254] |
|  | [**gi|194401103|gb|ACF61325.1|**](http://10.200.41.11/mascot/cgi/master_results.pl?file=..%2Fdata%2F20160609%2FF081283.dat#Hit13) | ribosomal protein L6 [Salmonella enterica subsp. enterica serovar Newport str. SL254] |
|  | [**gi|194405415|gb|ACF65637.1|**](http://10.200.41.11/mascot/cgi/master_results.pl?file=..%2Fdata%2F20160609%2FF081283.dat#Hit14) | ribosomal protein L15 [Salmonella enterica subsp. enterica serovar Newport str. SL254] |
|  | [**gi|194401633|gb|ACF61855.1|**](http://10.200.41.11/mascot/cgi/master_results.pl?file=..%2Fdata%2F20160609%2FF081283.dat#Hit15) | ribosomal protein S8 [Salmonella enterica subsp. enterica serovar Newport str. SL254] |
|  | [**gi|194404279|gb|ACF64501.1|**](http://10.200.41.11/mascot/cgi/master_results.pl?file=..%2Fdata%2F20160609%2FF081283.dat#Hit16) | ribosomal protein S12 [Salmonella enterica subsp. enterica serovar Newport str. SL254] |
|  | [**gi|194404219|gb|ACF64441.1|**](http://10.200.41.11/mascot/cgi/master_results.pl?file=..%2Fdata%2F20160609%2FF081283.dat#Hit17) | flagellar hook-associated protein 3 [Salmonella enterica subsp. enterica serovar Newport str. SL254] |
|  | [**gi|446662362|ref|WP_000739708.1|**](http://10.200.41.11/mascot/cgi/master_results.pl?file=..%2Fdata%2F20160609%2FF081283.dat#Hit18) | hypothetical protein [Salmonella enterica] |
|  | [**gi|194402866|gb|ACF63088.1|**](http://10.200.41.11/mascot/cgi/master_results.pl?file=..%2Fdata%2F20160609%2FF081283.dat#Hit19) | ribosomal protein L31 [Salmonella enterica subsp. enterica serovar Newport str. SL254] |
|  | [**gi|195630380|gb|EDX49006.1|**](http://10.200.41.11/mascot/cgi/master_results.pl?file=..%2Fdata%2F20160609%2FF081283.dat#Hit20) | protein RecT [Salmonella enterica subsp. enterica serovar Newport str. SL317] |
|  | [**gi|194402721|gb|ACF62943.1|**](http://10.200.41.11/mascot/cgi/master_results.pl?file=..%2Fdata%2F20160609%2FF081283.dat#Hit21) | autonomous glycyl radical cofactor [Salmonella enterica subsp. enterica serovar Newport str. SL254] |
|  | [**gi|194404877|gb|ACF65099.1|**](http://10.200.41.11/mascot/cgi/master_results.pl?file=..%2Fdata%2F20160609%2FF081283.dat#Hit22) | ABC transporter domain protein [Salmonella enterica subsp. enterica serovar Newport str. SL254] |

| **1.** | [gi|50830890|gb|AAT81610.1|](http://10.200.41.11/mascot/cgi/protein_view.pl?file=..%2Fdata%2F20160609%2FF081283.dat&hit=gi%7C50830890%7Cgb%7CAAT81610.1%7C&db_idx=1&px=1&ave_thresh=32&_ignoreionsscorebelow=0&report=0&_sigthreshold=0.05&_msresflags=1089&_msresflags2=2&percolate=-1&percolate_rt=0&_minpeplen=5&sessionID=all_secdisabledsession)    **Mass:** 52223    **Score:** 388    **Matches:** 11(10)  **Sequences:** 3(3)  **emPAI:** 0.37 |
| --- | --- |
|  | phase 1 flagellin [Salmonella enterica subsp. enterica serovar Newport] |

|  | **Query** | **Observed** | **Mr(expt)** | **Mr(calc)** | **ppm** | **Miss** | **Score** | **Expect** | **Rank** | **Unique** | **Peptide** |
| --- | --- | --- | --- | --- | --- | --- | --- | --- | --- | --- | --- |
|  | [1297](http://10.200.41.11/mascot/cgi/peptide_view.pl?file=..%2Fdata%2F20160609%2FF081283.dat&query=1297&hit=1&index=gi%7C50830890%7Cgb%7CAAT81610.1%7C&db_idx=1&px=1&section=5&ave_thresh=32&_ignoreionsscorebelow=0&report=0&_sigthreshold=0.05&_msresflags=1089&_msresflags2=2&percolate=-1&percolate_rt=0&_minpeplen=5&sessionID=all_secdisabledsession) | **566.8033** | **1131.5920** | **1131.5884** | **3.21** | **0** | **68** | **3.2e-005** | **1** | **U** | **K.SQSALGTAIER.L** [1298](http://10.200.41.11/mascot/cgi/peptide_view.pl?file=..%2Fdata%2F20160609%2FF081283.dat&query=1298&hit=1&index=gi%7C50830890%7Cgb%7CAAT81610.1%7C&db_idx=1&px=1&section=5&ave_thresh=32&_ignoreionsscorebelow=0&report=0&_sigthreshold=0.05&_msresflags=1089&_msresflags2=2&percolate=-1&percolate_rt=0&_minpeplen=5&sessionID=all_secdisabledsession) [1299](http://10.200.41.11/mascot/cgi/peptide_view.pl?file=..%2Fdata%2F20160609%2FF081283.dat&query=1299&hit=1&index=gi%7C50830890%7Cgb%7CAAT81610.1%7C&db_idx=1&px=1&section=5&ave_thresh=32&_ignoreionsscorebelow=0&report=0&_sigthreshold=0.05&_msresflags=1089&_msresflags2=2&percolate=-1&percolate_rt=0&_minpeplen=5&sessionID=all_secdisabledsession) |
|  | [2380](http://10.200.41.11/mascot/cgi/peptide_view.pl?file=..%2Fdata%2F20160609%2FF081283.dat&query=2380&hit=1&index=gi%7C50830890%7Cgb%7CAAT81610.1%7C&db_idx=1&px=1&section=5&ave_thresh=32&_ignoreionsscorebelow=0&report=0&_sigthreshold=0.05&_msresflags=1089&_msresflags2=2&percolate=-1&percolate_rt=0&_minpeplen=5&sessionID=all_secdisabledsession) | **807.9163** | **1613.8180** | **1613.8121** | **3.63** | **1** | **76** | **4.5e-006** | **1** | **U** | **R.INSAKDDAAGQAIANR.F** |
|  | [2382](http://10.200.41.11/mascot/cgi/peptide_view.pl?file=..%2Fdata%2F20160609%2FF081283.dat&query=2382&hit=1&index=gi%7C50830890%7Cgb%7CAAT81610.1%7C&db_idx=1&px=1&section=5&ave_thresh=32&_ignoreionsscorebelow=0&report=0&_sigthreshold=0.05&_msresflags=1089&_msresflags2=2&percolate=-1&percolate_rt=0&_minpeplen=5&sessionID=all_secdisabledsession) | **538.9467** | **1613.8181** | **1613.8121** | **3.74** | **1** | **(51)** | **0.0015** | **1** | **U** | **R.INSAKDDAAGQAIANR.F** [2381](http://10.200.41.11/mascot/cgi/peptide_view.pl?file=..%2Fdata%2F20160609%2FF081283.dat&query=2381&hit=1&index=gi%7C50830890%7Cgb%7CAAT81610.1%7C&db_idx=1&px=1&section=5&ave_thresh=32&_ignoreionsscorebelow=0&report=0&_sigthreshold=0.05&_msresflags=1089&_msresflags2=2&percolate=-1&percolate_rt=0&_minpeplen=5&sessionID=all_secdisabledsession) [2383](http://10.200.41.11/mascot/cgi/peptide_view.pl?file=..%2Fdata%2F20160609%2FF081283.dat&query=2383&hit=1&index=gi%7C50830890%7Cgb%7CAAT81610.1%7C&db_idx=1&px=1&section=5&ave_thresh=32&_ignoreionsscorebelow=0&report=0&_sigthreshold=0.05&_msresflags=1089&_msresflags2=2&percolate=-1&percolate_rt=0&_minpeplen=5&sessionID=all_secdisabledsession) |
|  | [2733](http://10.200.41.11/mascot/cgi/peptide_view.pl?file=..%2Fdata%2F20160609%2FF081283.dat&query=2733&hit=1&index=gi%7C50830890%7Cgb%7CAAT81610.1%7C&db_idx=1&px=1&section=5&ave_thresh=32&_ignoreionsscorebelow=0&report=0&_sigthreshold=0.05&_msresflags=1089&_msresflags2=2&percolate=-1&percolate_rt=0&_minpeplen=5&sessionID=all_secdisabledsession) | **659.2963** | **1974.8670** | **1974.8589** | **4.10** | **1** | **56** | **9.7e-005** | **1** | **U** | **R.SRIEDSDYATEVSNMSR.A** [2732](http://10.200.41.11/mascot/cgi/peptide_view.pl?file=..%2Fdata%2F20160609%2FF081283.dat&query=2732&hit=1&index=gi%7C50830890%7Cgb%7CAAT81610.1%7C&db_idx=1&px=1&section=5&ave_thresh=32&_ignoreionsscorebelow=0&report=0&_sigthreshold=0.05&_msresflags=1089&_msresflags2=2&percolate=-1&percolate_rt=0&_minpeplen=5&sessionID=all_secdisabledsession) [2734](http://10.200.41.11/mascot/cgi/peptide_view.pl?file=..%2Fdata%2F20160609%2FF081283.dat&query=2734&hit=1&index=gi%7C50830890%7Cgb%7CAAT81610.1%7C&db_idx=1&px=1&section=5&ave_thresh=32&_ignoreionsscorebelow=0&report=0&_sigthreshold=0.05&_msresflags=1089&_msresflags2=2&percolate=-1&percolate_rt=0&_minpeplen=5&sessionID=all_secdisabledsession) [2735](http://10.200.41.11/mascot/cgi/peptide_view.pl?file=..%2Fdata%2F20160609%2FF081283.dat&query=2735&hit=1&index=gi%7C50830890%7Cgb%7CAAT81610.1%7C&db_idx=1&px=1&section=5&ave_thresh=32&_ignoreionsscorebelow=0&report=0&_sigthreshold=0.05&_msresflags=1089&_msresflags2=2&percolate=-1&percolate_rt=0&_minpeplen=5&sessionID=all_secdisabledsession) |

### Protein sequence coverage: 8%

Matched peptides shown in ***bold red***.

| **1** | MAQVINTNSL | SLLTQNNLNK | **SQSALGTAIE** | **R**LSSGLR**INS** | **AKDDAAGQAI** |
| --- | --- | --- | --- | --- | --- |
| **51** | **ANR**FTANIKG | LTQASRNAND | GISIAQTTEG | ALNEINNNLQ | RVRELAVQSA |
| **101** | NSTNSQSDLD | SIQAEITQRL | NEIDRVSGQT | QFNGVKVLAQ | DNTLTIQVGA |
| **151** | NDGETIDIDL | KQINSQTLGL | DTLNVQKAYD | VSATAAMDPK | SFTDGTKNLT |
| **201** | APDATAIKAA | LGNPAATGDS | LSATLSFKDG | KYYATVAGYT | NAADTSKNGK |
| **251** | YEVNVDSATG | AVTFNAAPTK | ATVTGDTTVT | KVQVNAPVAV | STDVKKALED |
| **301** | GGVSNADATA | AKLVKMSYTD | KNGKSIDGGY | ALEAGGKYYA | ATYDEGTGKI |
| **351** | TANVTTYTDS | TGVTKTAANQ | LGGVDGKTEV | VTIDGKTYNA | SKAAGHDFKA |
| **401** | QPELAEAAAK | TTENPLAKID | AALAQVDALR | SDLGAVQNRF | NSAITNLGNT |
| **451** | VNNLSEAR**SR** | **IEDSDYATEV** | **SNMSR**AQILQ | QAGTSVLAQA | NQVPQNVLSL |
| **501** | LR |  |  |  |  |

| **2.** | [gi|194402702|gb|ACF62924.1|](http://10.200.41.11/mascot/cgi/protein_view.pl?file=..%2Fdata%2F20160609%2FF081283.dat&hit=gi%7C194402702%7Cgb%7CACF62924.1%7C&db_idx=1&px=1&ave_thresh=32&_ignoreionsscorebelow=0&report=0&_sigthreshold=0.05&_msresflags=1089&_msresflags2=2&percolate=-1&percolate_rt=0&_minpeplen=5&sessionID=all_secdisabledsession)    **Mass:** 72333    **Score:** 299    **Matches:** 11(8)  **Sequences:** 5(4)  **emPAI:** 0.35 |
| --- | --- |
|  | cell invasion protein SipA [Salmonella enterica subsp. enterica serovar Newport str. SL254] |

|  | **Query** | **Observed** | **Mr(expt)** | **Mr(calc)** | **ppm** | **Miss** | **Score** | **Expect** | **Rank** | **Unique** | **Peptide** |
| --- | --- | --- | --- | --- | --- | --- | --- | --- | --- | --- | --- |
|  | [720](http://10.200.41.11/mascot/cgi/peptide_view.pl?file=..%2Fdata%2F20160609%2FF081283.dat&query=720&hit=1&index=gi%7C194402702%7Cgb%7CACF62924.1%7C&db_idx=1&px=1&section=5&ave_thresh=32&_ignoreionsscorebelow=0&report=0&_sigthreshold=0.05&_msresflags=1089&_msresflags2=2&percolate=-1&percolate_rt=0&_minpeplen=5&sessionID=all_secdisabledsession) | **490.7454** | **979.4763** | **979.4723** | **4.07** | **0** | **50** | **0.0013** | **1** | **U** | **R.TFIDNSQR.N** [719](http://10.200.41.11/mascot/cgi/peptide_view.pl?file=..%2Fdata%2F20160609%2FF081283.dat&query=719&hit=1&index=gi%7C194402702%7Cgb%7CACF62924.1%7C&db_idx=1&px=1&section=5&ave_thresh=32&_ignoreionsscorebelow=0&report=0&_sigthreshold=0.05&_msresflags=1089&_msresflags2=2&percolate=-1&percolate_rt=0&_minpeplen=5&sessionID=all_secdisabledsession) [721](http://10.200.41.11/mascot/cgi/peptide_view.pl?file=..%2Fdata%2F20160609%2FF081283.dat&query=721&hit=1&index=gi%7C194402702%7Cgb%7CACF62924.1%7C&db_idx=1&px=1&section=5&ave_thresh=32&_ignoreionsscorebelow=0&report=0&_sigthreshold=0.05&_msresflags=1089&_msresflags2=2&percolate=-1&percolate_rt=0&_minpeplen=5&sessionID=all_secdisabledsession) [722](http://10.200.41.11/mascot/cgi/peptide_view.pl?file=..%2Fdata%2F20160609%2FF081283.dat&query=722&hit=1&index=gi%7C194402702%7Cgb%7CACF62924.1%7C&db_idx=1&px=1&section=5&ave_thresh=32&_ignoreionsscorebelow=0&report=0&_sigthreshold=0.05&_msresflags=1089&_msresflags2=2&percolate=-1&percolate_rt=0&_minpeplen=5&sessionID=all_secdisabledsession) |
|  | [1504](http://10.200.41.11/mascot/cgi/peptide_view.pl?file=..%2Fdata%2F20160609%2FF081283.dat&query=1504&hit=1&index=gi%7C194402702%7Cgb%7CACF62924.1%7C&db_idx=1&px=1&section=5&ave_thresh=32&_ignoreionsscorebelow=0&report=0&_sigthreshold=0.05&_msresflags=1089&_msresflags2=2&percolate=-1&percolate_rt=0&_minpeplen=5&sessionID=all_secdisabledsession) | **588.3064** | **1174.5982** | **1174.5942** | **3.42** | **0** | **60** | **0.00021** | **1** | **U** | **K.LTQEQGTSVGR.E** [1503](http://10.200.41.11/mascot/cgi/peptide_view.pl?file=..%2Fdata%2F20160609%2FF081283.dat&query=1503&hit=1&index=gi%7C194402702%7Cgb%7CACF62924.1%7C&db_idx=1&px=1&section=5&ave_thresh=32&_ignoreionsscorebelow=0&report=0&_sigthreshold=0.05&_msresflags=1089&_msresflags2=2&percolate=-1&percolate_rt=0&_minpeplen=5&sessionID=all_secdisabledsession) [1505](http://10.200.41.11/mascot/cgi/peptide_view.pl?file=..%2Fdata%2F20160609%2FF081283.dat&query=1505&hit=1&index=gi%7C194402702%7Cgb%7CACF62924.1%7C&db_idx=1&px=1&section=5&ave_thresh=32&_ignoreionsscorebelow=0&report=0&_sigthreshold=0.05&_msresflags=1089&_msresflags2=2&percolate=-1&percolate_rt=0&_minpeplen=5&sessionID=all_secdisabledsession) |
|  | [1835](http://10.200.41.11/mascot/cgi/peptide_view.pl?file=..%2Fdata%2F20160609%2FF081283.dat&query=1835&hit=1&index=gi%7C194402702%7Cgb%7CACF62924.1%7C&db_idx=1&px=1&section=5&ave_thresh=32&_ignoreionsscorebelow=0&report=0&_sigthreshold=0.05&_msresflags=1089&_msresflags2=2&percolate=-1&percolate_rt=0&_minpeplen=5&sessionID=all_secdisabledsession) | **645.8151** | **1289.6156** | **1289.6099** | **4.40** | **0** | **71** | **7.9e-006** | **1** | **U** | **R.SALNATSDSPEAK.T** |
|  | [2066](http://10.200.41.11/mascot/cgi/peptide_view.pl?file=..%2Fdata%2F20160609%2FF081283.dat&query=2066&hit=1&index=gi%7C194402702%7Cgb%7CACF62924.1%7C&db_idx=1&px=1&section=5&ave_thresh=32&_ignoreionsscorebelow=0&report=0&_sigthreshold=0.05&_msresflags=1089&_msresflags2=2&percolate=-1&percolate_rt=0&_minpeplen=5&sessionID=all_secdisabledsession) | **462.5804** | **1384.7192** | **1384.7133** | **4.28** | **0** | **3** | **71** | **1** | **U** | **R.VITTVDGLHMQR.-** |
|  | [2813](http://10.200.41.11/mascot/cgi/peptide_view.pl?file=..%2Fdata%2F20160609%2FF081283.dat&query=2813&hit=1&index=gi%7C194402702%7Cgb%7CACF62924.1%7C&db_idx=1&px=1&section=5&ave_thresh=32&_ignoreionsscorebelow=0&report=0&_sigthreshold=0.05&_msresflags=1089&_msresflags2=2&percolate=-1&percolate_rt=0&_minpeplen=5&sessionID=all_secdisabledsession) | **825.3999** | **2473.1779** | **2473.1708** | **2.85** | **1** | **68** | **2.2e-005** | **1** | **U** | **K.VVTSEKGTTGETTSFDEVDGVTSK.S** [2814](http://10.200.41.11/mascot/cgi/peptide_view.pl?file=..%2Fdata%2F20160609%2FF081283.dat&query=2814&hit=1&index=gi%7C194402702%7Cgb%7CACF62924.1%7C&db_idx=1&px=1&section=5&ave_thresh=32&_ignoreionsscorebelow=0&report=0&_sigthreshold=0.05&_msresflags=1089&_msresflags2=2&percolate=-1&percolate_rt=0&_minpeplen=5&sessionID=all_secdisabledsession) |

### Protein sequence coverage: 10%

Matched peptides shown in ***bold red***.

| **1** | MQTEIKTQAT | NLAANLSAVR | ESATTTLSGE | IKGPQLEDFP | ALIKQASLDA |
| --- | --- | --- | --- | --- | --- |
| **51** | LFKCGKDAEA | LKEVFTNSNN | VAGKKAIMEF | AGLFR**SALNA** | **TSDSPEAK**TL |
| **101** | LMKVGAEYTA | QIIKDGLKEK | SAFGPWLPET | KKAEAKLENL | EKQLLDIIKN |
| **151** | NTGGELSKLS | TNLVMQEVMP | YIASCIEHNF | GCTLDPLTRS | NLTHLVDKAA |
| **201** | AKAVEALDMC | HQK**LTQEQGT** | **SVGR**EARHLE | MQTLIPLLLR | NVFAQIPADK |
| **251** | LPDPKIPEPA | AGPVPDGGKK | AEPTGININI | NIDSSNHSVD | NSKHINNSRS |
| **301** | HVDNSQRHID | NSNHDNSRKT | IDNSR**TFIDN** | **SQR**NGESHHS | TNSSNVSHSH |
| **351** | SRVDSTTHQT | ETAHSASTGA | IDHGIAGKID | VTAHATAEAV | TNASSESKDG |
| **401** | K**VVTSEKGTT** | **GETTSFDEVD** | **GVTSK**SIIGK | PVQATVHGVD | DNKQQSQTAE |
| **451** | IVNVKPLASQ | LAGVENVKTD | TLQSDTTVIT | GNKAGTTDND | NSQTDKTGPF |
| **501** | SGLKFKQNSF | LSTVPSVTNM | HSMHFDARET | FLGVIRKALE | PDTSTPFPVR |
| **551** | RAFDGLRAEI | LPNDTIKSAA | LKAQCSDIDK | HPELKAKMET | LKEVITHHPQ |
| **601** | KEKLAEIALQ | FAREAGLTRL | KGETDYVLSN | VLDGLIGDGS | WRAGPAYESY |
| **651** | LNKPGVDR**VI** | **TTVDGLHMQR** |  |  |  |

| **3.** | [gi|392616945|gb|EIW99373.1|](http://10.200.41.11/mascot/cgi/protein_view.pl?file=..%2Fdata%2F20160609%2FF081283.dat&hit=gi%7C392616945%7Cgb%7CEIW99373.1%7C&db_idx=1&px=1&ave_thresh=32&_ignoreionsscorebelow=0&report=0&_sigthreshold=0.05&_msresflags=1089&_msresflags2=2&percolate=-1&percolate_rt=0&_minpeplen=5&sessionID=all_secdisabledsession)    **Mass:** 42957    **Score:** 289    **Matches:** 12(9)  **Sequences:** 5(4)  **emPAI:** 0.66 |
| --- | --- |
|  | pathogenicity island 1 effector protein SipC [Salmonella enterica subsp. enterica serovar Newport str. Levine 15] |

|  | **Query** | **Observed** | **Mr(expt)** | **Mr(calc)** | **ppm** | **Miss** | **Score** | **Expect** | **Rank** | **Unique** | **Peptide** |
| --- | --- | --- | --- | --- | --- | --- | --- | --- | --- | --- | --- |
|  | [765](http://10.200.41.11/mascot/cgi/peptide_view.pl?file=..%2Fdata%2F20160609%2FF081283.dat&query=765&hit=1&index=gi%7C392616945%7Cgb%7CEIW99373.1%7C&db_idx=1&px=1&section=5&ave_thresh=32&_ignoreionsscorebelow=0&report=0&_sigthreshold=0.05&_msresflags=1089&_msresflags2=2&percolate=-1&percolate_rt=0&_minpeplen=5&sessionID=all_secdisabledsession) | **494.7715** | **987.5285** | **987.5237** | **4.87** | **0** | **58** | **0.0004** | **1** | **U** | **K.LGAEGVDSLK.S** [764](http://10.200.41.11/mascot/cgi/peptide_view.pl?file=..%2Fdata%2F20160609%2FF081283.dat&query=764&hit=1&index=gi%7C392616945%7Cgb%7CEIW99373.1%7C&db_idx=1&px=1&section=5&ave_thresh=32&_ignoreionsscorebelow=0&report=0&_sigthreshold=0.05&_msresflags=1089&_msresflags2=2&percolate=-1&percolate_rt=0&_minpeplen=5&sessionID=all_secdisabledsession) |
|  | [956](http://10.200.41.11/mascot/cgi/peptide_view.pl?file=..%2Fdata%2F20160609%2FF081283.dat&query=956&hit=1&index=gi%7C392616945%7Cgb%7CEIW99373.1%7C&db_idx=1&px=1&section=5&ave_thresh=32&_ignoreionsscorebelow=0&report=0&_sigthreshold=0.05&_msresflags=1089&_msresflags2=2&percolate=-1&percolate_rt=0&_minpeplen=5&sessionID=all_secdisabledsession) | **519.2522** | **1036.4898** | **1036.4859** | **3.82** | **0** | **38** | **0.016** | **1** | **U** | **K.TMESINQSK.A** [957](http://10.200.41.11/mascot/cgi/peptide_view.pl?file=..%2Fdata%2F20160609%2FF081283.dat&query=957&hit=1&index=gi%7C392616945%7Cgb%7CEIW99373.1%7C&db_idx=1&px=1&section=5&ave_thresh=32&_ignoreionsscorebelow=0&report=0&_sigthreshold=0.05&_msresflags=1089&_msresflags2=2&percolate=-1&percolate_rt=0&_minpeplen=5&sessionID=all_secdisabledsession) |
|  | [980](http://10.200.41.11/mascot/cgi/peptide_view.pl?file=..%2Fdata%2F20160609%2FF081283.dat&query=980&hit=1&index=gi%7C392616945%7Cgb%7CEIW99373.1%7C&db_idx=1&px=1&section=5&ave_thresh=32&_ignoreionsscorebelow=0&report=0&_sigthreshold=0.05&_msresflags=1089&_msresflags2=2&percolate=-1&percolate_rt=0&_minpeplen=5&sessionID=all_secdisabledsession) | **525.7973** | **1049.5801** | **1049.5757** | **4.15** | **0** | **42** | **0.0072** | **1** | **U** | **K.LSLVSFDAAK.T** [979](http://10.200.41.11/mascot/cgi/peptide_view.pl?file=..%2Fdata%2F20160609%2FF081283.dat&query=979&hit=1&index=gi%7C392616945%7Cgb%7CEIW99373.1%7C&db_idx=1&px=1&section=5&ave_thresh=32&_ignoreionsscorebelow=0&report=0&_sigthreshold=0.05&_msresflags=1089&_msresflags2=2&percolate=-1&percolate_rt=0&_minpeplen=5&sessionID=all_secdisabledsession) |
|  | [1914](http://10.200.41.11/mascot/cgi/peptide_view.pl?file=..%2Fdata%2F20160609%2FF081283.dat&query=1914&hit=1&index=gi%7C392616945%7Cgb%7CEIW99373.1%7C&db_idx=1&px=1&section=5&ave_thresh=32&_ignoreionsscorebelow=0&report=0&_sigthreshold=0.05&_msresflags=1089&_msresflags2=2&percolate=-1&percolate_rt=0&_minpeplen=5&sessionID=all_secdisabledsession) | **651.8254** | **1301.6363** | **1301.6324** | **3.04** | **0** | **72** | **7e-006** | **1** | **U** | **R.SEQQISQVNNR.V** [1915](http://10.200.41.11/mascot/cgi/peptide_view.pl?file=..%2Fdata%2F20160609%2FF081283.dat&query=1915&hit=1&index=gi%7C392616945%7Cgb%7CEIW99373.1%7C&db_idx=1&px=1&section=5&ave_thresh=32&_ignoreionsscorebelow=0&report=0&_sigthreshold=0.05&_msresflags=1089&_msresflags2=2&percolate=-1&percolate_rt=0&_minpeplen=5&sessionID=all_secdisabledsession) [1916](http://10.200.41.11/mascot/cgi/peptide_view.pl?file=..%2Fdata%2F20160609%2FF081283.dat&query=1916&hit=1&index=gi%7C392616945%7Cgb%7CEIW99373.1%7C&db_idx=1&px=1&section=5&ave_thresh=32&_ignoreionsscorebelow=0&report=0&_sigthreshold=0.05&_msresflags=1089&_msresflags2=2&percolate=-1&percolate_rt=0&_minpeplen=5&sessionID=all_secdisabledsession) |
|  | [2222](http://10.200.41.11/mascot/cgi/peptide_view.pl?file=..%2Fdata%2F20160609%2FF081283.dat&query=2222&hit=1&index=gi%7C392616945%7Cgb%7CEIW99373.1%7C&db_idx=1&px=1&section=5&ave_thresh=32&_ignoreionsscorebelow=0&report=0&_sigthreshold=0.05&_msresflags=1089&_msresflags2=2&percolate=-1&percolate_rt=0&_minpeplen=5&sessionID=all_secdisabledsession) | **733.3495** | **1464.6844** | **1464.6804** | **2.72** | **1** | **20** | **1.1** | **1** | **U** | **R.VASTASDEARESSR.K** |
|  | [2223](http://10.200.41.11/mascot/cgi/peptide_view.pl?file=..%2Fdata%2F20160609%2FF081283.dat&query=2223&hit=1&index=gi%7C392616945%7Cgb%7CEIW99373.1%7C&db_idx=1&px=1&section=5&ave_thresh=32&_ignoreionsscorebelow=0&report=0&_sigthreshold=0.05&_msresflags=1089&_msresflags2=2&percolate=-1&percolate_rt=0&_minpeplen=5&sessionID=all_secdisabledsession) | **489.2358** | **1464.6856** | **1464.6804** | **3.53** | **1** | **(6)** | **29** | **1** | **U** | **R.VASTASDEARESSR.K** [2225](http://10.200.41.11/mascot/cgi/peptide_view.pl?file=..%2Fdata%2F20160609%2FF081283.dat&query=2225&hit=1&index=gi%7C392616945%7Cgb%7CEIW99373.1%7C&db_idx=1&px=1&section=5&ave_thresh=32&_ignoreionsscorebelow=0&report=0&_sigthreshold=0.05&_msresflags=1089&_msresflags2=2&percolate=-1&percolate_rt=0&_minpeplen=5&sessionID=all_secdisabledsession) |

### Protein sequence coverage: 13%

Matched peptides shown in ***bold red***.

| **1** | MLISNVGINP | AAYLNNHSVE | NSSQTASQSV | SAKDILNSIG | ISSSKVSDLG |
| --- | --- | --- | --- | --- | --- |
| **51** | LSPTLSAPAP | GVLTQTPGTI | TSFLKASIQN | TDMNQDLNAL | ANNVTTKANE |
| **101** | VVQTQLREQQ | AEVGKFFDIS | GMSSSAVALL | AAANTLMLTL | NQADSKLSGK |
| **151** | **LSLVSFDAAK** | TTASSMMREG | MNALSGSISQ | SALQLGITGV | GAKLEYKGLQ |
| **201** | NERGALKHNA | AKIDKLTTES | HSIKNVLNGQ | NSVK**LGAEGV** | **DSLK**SLNMKK |
| **251** | TGTDATKNLN | DATLKSNAGT | SATESLGIKD | SNKQISPEHQ | AILSKRLESV |
| **301** | ESDIRLEQNT | MDMTRIDARK | MQMTGDLIMK | NSVTVGGIAG | ASGQYAATQE |
| **351** | R**SEQQISQVN** | **NRVASTASDE** | **ARESSR**KSTS | LIQEMLK**TME** | **SINQSK**ASAL |
| **401** | AAIAGNIRA |  |  |  |  |

| **4.** | [gi|392616944|gb|EIW99372.1|](http://10.200.41.11/mascot/cgi/protein_view.pl?file=..%2Fdata%2F20160609%2FF081283.dat&hit=gi%7C392616944%7Cgb%7CEIW99372.1%7C&db_idx=1&px=1&ave_thresh=32&_ignoreionsscorebelow=0&report=0&_sigthreshold=0.05&_msresflags=1089&_msresflags2=2&percolate=-1&percolate_rt=0&_minpeplen=5&sessionID=all_secdisabledsession)    **Mass:** 37081    **Score:** 225    **Matches:** 4(4)  **Sequences:** 2(2)  **emPAI:** 0.34 |
| --- | --- |
|  | cell invasion protein SipD [Salmonella enterica subsp. enterica serovar Newport str. Levine 15] |

|  | **Query** | **Observed** | **Mr(expt)** | **Mr(calc)** | **ppm** | **Miss** | **Score** | **Expect** | **Rank** | **Unique** | **Peptide** |
| --- | --- | --- | --- | --- | --- | --- | --- | --- | --- | --- | --- |
|  | [1766](http://10.200.41.11/mascot/cgi/peptide_view.pl?file=..%2Fdata%2F20160609%2FF081283.dat&query=1766&hit=1&index=gi%7C392616944%7Cgb%7CEIW99372.1%7C&db_idx=1&px=1&section=5&ave_thresh=32&_ignoreionsscorebelow=0&report=0&_sigthreshold=0.05&_msresflags=1089&_msresflags2=2&percolate=-1&percolate_rt=0&_minpeplen=5&sessionID=all_secdisabledsession) | **637.3610** | **1272.7075** | **1272.7037** | **2.95** | **0** | **46** | **0.0043** | **1** | **U** | **R.QQLTSSLNALAK.S** |
|  | [2261](http://10.200.41.11/mascot/cgi/peptide_view.pl?file=..%2Fdata%2F20160609%2FF081283.dat&query=2261&hit=1&index=gi%7C392616944%7Cgb%7CEIW99372.1%7C&db_idx=1&px=1&section=5&ave_thresh=32&_ignoreionsscorebelow=0&report=0&_sigthreshold=0.05&_msresflags=1089&_msresflags2=2&percolate=-1&percolate_rt=0&_minpeplen=5&sessionID=all_secdisabledsession) | **752.3761** | **1502.7376** | **1502.7325** | **3.45** | **0** | **98** | **2.2e-008** | **1** | **U** | **K.SGVSLSAEQNENLR.S** [2260](http://10.200.41.11/mascot/cgi/peptide_view.pl?file=..%2Fdata%2F20160609%2FF081283.dat&query=2260&hit=1&index=gi%7C392616944%7Cgb%7CEIW99372.1%7C&db_idx=1&px=1&section=5&ave_thresh=32&_ignoreionsscorebelow=0&report=0&_sigthreshold=0.05&_msresflags=1089&_msresflags2=2&percolate=-1&percolate_rt=0&_minpeplen=5&sessionID=all_secdisabledsession) [2262](http://10.200.41.11/mascot/cgi/peptide_view.pl?file=..%2Fdata%2F20160609%2FF081283.dat&query=2262&hit=1&index=gi%7C392616944%7Cgb%7CEIW99372.1%7C&db_idx=1&px=1&section=5&ave_thresh=32&_ignoreionsscorebelow=0&report=0&_sigthreshold=0.05&_msresflags=1089&_msresflags2=2&percolate=-1&percolate_rt=0&_minpeplen=5&sessionID=all_secdisabledsession) |

### Protein sequence coverage: 7%

Matched peptides shown in ***bold red***.

| **1** | MLNIQNYSAS | PHPGIVAERP | QTPSASEHVE | TAVVPSTTEH | RGTDIISLSQ |
| --- | --- | --- | --- | --- | --- |
| **51** | AATKIQQAQQ | TLQSTPPISE | ENNDERTLAR | **QQLTSSLNAL** | **AKSGVSLSAE** |
| **101** | **QNENLR**SAFS | APTSALFSAS | PMAQPRTTIS | DAEIWDMVSQ | NISAIGDSYL |
| **151** | GVYENVVAVY | TDFYQAFSDI | LSKMGGWLLP | GKDGNTVKLD | VTSLKNDLNS |
| **201** | LVNKYNQINS | NTVLFPAQSG | SGVKVATEAE | ARQWLSELNL | PNSCLKSYGS |
| **251** | GYVVTVDLTP | LQKMVQDIDG | LGAPGKDSKL | EMDNAKYQAW | QSGFKAQEEN |
| **301** | MKTTLQTLTQ | KYSNANSLYD | NLVKVLSSTI | SSSLETAKSF | LQG |

| **5.** | [gi|194403331|gb|ACF63553.1|](http://10.200.41.11/mascot/cgi/protein_view.pl?file=..%2Fdata%2F20160609%2FF081283.dat&hit=gi%7C194403331%7Cgb%7CACF63553.1%7C&db_idx=1&px=1&ave_thresh=32&_ignoreionsscorebelow=0&report=0&_sigthreshold=0.05&_msresflags=1089&_msresflags2=2&percolate=-1&percolate_rt=0&_minpeplen=5&sessionID=all_secdisabledsession)    **Mass:** 10561    **Score:** 209    **Matches:** 10(6)  **Sequences:** 3(2)  **emPAI:** 1.68 |
| --- | --- |
|  | negative regulator of flagellin synthesis [Salmonella enterica subsp. enterica serovar Newport str. SL254] |

|  | **Query** | **Observed** | **Mr(expt)** | **Mr(calc)** | **ppm** | **Miss** | **Score** | **Expect** | **Rank** | **Unique** | **Peptide** |
| --- | --- | --- | --- | --- | --- | --- | --- | --- | --- | --- | --- |
|  | [983](http://10.200.41.11/mascot/cgi/peptide_view.pl?file=..%2Fdata%2F20160609%2FF081283.dat&query=983&hit=1&index=gi%7C194403331%7Cgb%7CACF63553.1%7C&db_idx=1&px=1&section=5&ave_thresh=32&_ignoreionsscorebelow=0&report=0&_sigthreshold=0.05&_msresflags=1089&_msresflags2=2&percolate=-1&percolate_rt=0&_minpeplen=5&sessionID=all_secdisabledsession) | **527.2660** | **1052.5174** | **1052.5138** | **3.44** | **0** | **57** | **0.00021** | **1** | **U** | **R.EAQSYLQSK.-** [981](http://10.200.41.11/mascot/cgi/peptide_view.pl?file=..%2Fdata%2F20160609%2FF081283.dat&query=981&hit=1&index=gi%7C194403331%7Cgb%7CACF63553.1%7C&db_idx=1&px=1&section=5&ave_thresh=32&_ignoreionsscorebelow=0&report=0&_sigthreshold=0.05&_msresflags=1089&_msresflags2=2&percolate=-1&percolate_rt=0&_minpeplen=5&sessionID=all_secdisabledsession) [982](http://10.200.41.11/mascot/cgi/peptide_view.pl?file=..%2Fdata%2F20160609%2FF081283.dat&query=982&hit=1&index=gi%7C194403331%7Cgb%7CACF63553.1%7C&db_idx=1&px=1&section=5&ave_thresh=32&_ignoreionsscorebelow=0&report=0&_sigthreshold=0.05&_msresflags=1089&_msresflags2=2&percolate=-1&percolate_rt=0&_minpeplen=5&sessionID=all_secdisabledsession) [984](http://10.200.41.11/mascot/cgi/peptide_view.pl?file=..%2Fdata%2F20160609%2FF081283.dat&query=984&hit=1&index=gi%7C194403331%7Cgb%7CACF63553.1%7C&db_idx=1&px=1&section=5&ave_thresh=32&_ignoreionsscorebelow=0&report=0&_sigthreshold=0.05&_msresflags=1089&_msresflags2=2&percolate=-1&percolate_rt=0&_minpeplen=5&sessionID=all_secdisabledsession) |
|  | [2156](http://10.200.41.11/mascot/cgi/peptide_view.pl?file=..%2Fdata%2F20160609%2FF081283.dat&query=2156&hit=1&index=gi%7C194403331%7Cgb%7CACF63553.1%7C&db_idx=1&px=1&section=5&ave_thresh=32&_ignoreionsscorebelow=0&report=0&_sigthreshold=0.05&_msresflags=1089&_msresflags2=2&percolate=-1&percolate_rt=0&_minpeplen=5&sessionID=all_secdisabledsession) | **707.4097** | **1412.8049** | **1412.7987** | **4.37** | **0** | **(5)** | **38** | **1** | **U** | **R.TSPLKPVSTVQTR.E** |
|  | [2159](http://10.200.41.11/mascot/cgi/peptide_view.pl?file=..%2Fdata%2F20160609%2FF081283.dat&query=2159&hit=1&index=gi%7C194403331%7Cgb%7CACF63553.1%7C&db_idx=1&px=1&section=5&ave_thresh=32&_ignoreionsscorebelow=0&report=0&_sigthreshold=0.05&_msresflags=1089&_msresflags2=2&percolate=-1&percolate_rt=0&_minpeplen=5&sessionID=all_secdisabledsession) | **471.9426** | **1412.8059** | **1412.7987** | **5.05** | **0** | **20** | **1.2** | **1** | **U** | **R.TSPLKPVSTVQTR.E** [2157](http://10.200.41.11/mascot/cgi/peptide_view.pl?file=..%2Fdata%2F20160609%2FF081283.dat&query=2157&hit=1&index=gi%7C194403331%7Cgb%7CACF63553.1%7C&db_idx=1&px=1&section=5&ave_thresh=32&_ignoreionsscorebelow=0&report=0&_sigthreshold=0.05&_msresflags=1089&_msresflags2=2&percolate=-1&percolate_rt=0&_minpeplen=5&sessionID=all_secdisabledsession) [2158](http://10.200.41.11/mascot/cgi/peptide_view.pl?file=..%2Fdata%2F20160609%2FF081283.dat&query=2158&hit=1&index=gi%7C194403331%7Cgb%7CACF63553.1%7C&db_idx=1&px=1&section=5&ave_thresh=32&_ignoreionsscorebelow=0&report=0&_sigthreshold=0.05&_msresflags=1089&_msresflags2=2&percolate=-1&percolate_rt=0&_minpeplen=5&sessionID=all_secdisabledsession) |
|  | [2378](http://10.200.41.11/mascot/cgi/peptide_view.pl?file=..%2Fdata%2F20160609%2FF081283.dat&query=2378&hit=1&index=gi%7C194403331%7Cgb%7CACF63553.1%7C&db_idx=1&px=1&section=5&ave_thresh=32&_ignoreionsscorebelow=0&report=0&_sigthreshold=0.05&_msresflags=1089&_msresflags2=2&percolate=-1&percolate_rt=0&_minpeplen=5&sessionID=all_secdisabledsession) | **804.9105** | **1607.8065** | **1607.8002** | **3.89** | **0** | **75** | **4.7e-006** | **1** | **U** | **K.TSAATSASVTLSDAQAK.L** [2379](http://10.200.41.11/mascot/cgi/peptide_view.pl?file=..%2Fdata%2F20160609%2FF081283.dat&query=2379&hit=1&index=gi%7C194403331%7Cgb%7CACF63553.1%7C&db_idx=1&px=1&section=5&ave_thresh=32&_ignoreionsscorebelow=0&report=0&_sigthreshold=0.05&_msresflags=1089&_msresflags2=2&percolate=-1&percolate_rt=0&_minpeplen=5&sessionID=all_secdisabledsession) |

### Protein sequence coverage: 40%

Matched peptides shown in ***bold red***.

| **1** | MSIDR**TSPLK** | **PVSTVQTR**ET | SDTPVQKTRQ | EK**TSAATSAS** | **VTLSDAQAK**L |
| --- | --- | --- | --- | --- | --- |
| **51** | MQPGVSDINM | ERVEALKTAI | RNGELKMDTG | KIADSLIR**EA** | **QSYLQSK** |

| **6.** | [gi|194401173|gb|ACF61395.1|](http://10.200.41.11/mascot/cgi/protein_view.pl?file=..%2Fdata%2F20160609%2FF081283.dat&hit=gi%7C194401173%7Cgb%7CACF61395.1%7C&db_idx=1&px=1&ave_thresh=32&_ignoreionsscorebelow=0&report=0&_sigthreshold=0.05&_msresflags=1089&_msresflags2=2&percolate=-1&percolate_rt=0&_minpeplen=5&sessionID=all_secdisabledsession)    **Mass:** 49778    **Score:** 159    **Matches:** 2(2)  **Sequences:** 1(1)  **emPAI:** 0.12 |
| --- | --- |
|  | flagellar hook-associated protein 2 [Salmonella enterica subsp. enterica serovar Newport str. SL254] |

|  | **Query** | **Observed** | **Mr(expt)** | **Mr(calc)** | **ppm** | **Miss** | **Score** | **Expect** | **Rank** | **Unique** | **Peptide** |
| --- | --- | --- | --- | --- | --- | --- | --- | --- | --- | --- | --- |
|  | [2507](http://10.200.41.11/mascot/cgi/peptide_view.pl?file=..%2Fdata%2F20160609%2FF081283.dat&query=2507&hit=1&index=gi%7C194401173%7Cgb%7CACF61395.1%7C&db_idx=1&px=1&section=5&ave_thresh=32&_ignoreionsscorebelow=0&report=0&_sigthreshold=0.05&_msresflags=1089&_msresflags2=2&percolate=-1&percolate_rt=0&_minpeplen=5&sessionID=all_secdisabledsession) | **841.4265** | **1680.8385** | **1680.8319** | **3.93** | **0** | **103** | **8.4e-009** | **1** | **U** | **K.QYLSVSNSIDETVAR.Y** [2508](http://10.200.41.11/mascot/cgi/peptide_view.pl?file=..%2Fdata%2F20160609%2FF081283.dat&query=2508&hit=1&index=gi%7C194401173%7Cgb%7CACF61395.1%7C&db_idx=1&px=1&section=5&ave_thresh=32&_ignoreionsscorebelow=0&report=0&_sigthreshold=0.05&_msresflags=1089&_msresflags2=2&percolate=-1&percolate_rt=0&_minpeplen=5&sessionID=all_secdisabledsession) |

### Protein sequence coverage: 3%

Matched peptides shown in ***bold red***.

| **1** | MASISSLGVG | SNLPLDQLLT | DLTKNEKGRL | TPITKQQSAN | SAKLTAYGTL |
| --- | --- | --- | --- | --- | --- |
| **51** | KSALEKFQTA | NTALNKADLF | KSTVASSTTE | DLKVSTTAGA | AAGTYKISVT |
| **101** | QLAAAQSLAT | KTTFATTKEQ | LGDTSVTSRT | IKIEQPGRKE | PLEIKLDKGD |
| **151** | TSMEAIRDAI | NDADSGIAAS | IVKVKENEFQ | LVLTANSGTD | NTMKITVEGD |
| **201** | TKLNDLLAYD | STTNTGNMQE | LVKAENAKLN | VNGIDIERQS | NTVTDAPQGI |
| **251** | TLTLTKKVTD | ATVTVTKDDT | KAKEAIKSWV | DAYNSLVDTF | SSLTKYTAVE |
| **301** | PGEEASDKNG | ALLGDSVVRT | IQTGIRAQFA | NSGSNSAFKT | MAEIGITQDG |
| **351** | TSGKLKIDDD | KLTKVLKDNT | AAARELLVGD | GKETGITTKI | ATEVKSYLAD |
| **401** | DGIIDNAQDN | VNATLKSLTK | **QYLSVSNSID** | **ETVAR**YKAQF | TQLDTMMSKL |
| **451** | NNTSSYLTQQ | FTAMNKS |  |  |  |

| **7.** | [gi|194403640|gb|ACF63862.1|](http://10.200.41.11/mascot/cgi/protein_view.pl?file=..%2Fdata%2F20160609%2FF081283.dat&hit=gi%7C194403640%7Cgb%7CACF63862.1%7C&db_idx=1&px=1&ave_thresh=32&_ignoreionsscorebelow=0&report=0&_sigthreshold=0.05&_msresflags=1089&_msresflags2=2&percolate=-1&percolate_rt=0&_minpeplen=5&sessionID=all_secdisabledsession)    **Mass:** 62382    **Score:** 112    **Matches:** 5(2)  **Sequences:** 3(1)  **emPAI:** 0.09 |
| --- | --- |
|  | cell invasion protein SipB [Salmonella enterica subsp. enterica serovar Newport str. SL254] |

|  | **Query** | **Observed** | **Mr(expt)** | **Mr(calc)** | **ppm** | **Miss** | **Score** | **Expect** | **Rank** | **Unique** | **Peptide** |
| --- | --- | --- | --- | --- | --- | --- | --- | --- | --- | --- | --- |
|  | [720](http://10.200.41.11/mascot/cgi/peptide_view.pl?file=..%2Fdata%2F20160609%2FF081283.dat&query=720&hit=4&index=gi%7C194403640%7Cgb%7CACF63862.1%7C&db_idx=1&px=1&section=5&ave_thresh=32&_ignoreionsscorebelow=0&report=0&_sigthreshold=0.05&_msresflags=1089&_msresflags2=2&percolate=-1&percolate_rt=0&_minpeplen=5&sessionID=all_secdisabledsession) | 490.7454 | 979.4763 | 979.4909 | -14.95 | 0 | 4 | 52 | 4 | U | K.LFTQGMQR.I [719](http://10.200.41.11/mascot/cgi/peptide_view.pl?file=..%2Fdata%2F20160609%2FF081283.dat&query=719&hit=6&index=gi%7C194403640%7Cgb%7CACF63862.1%7C&db_idx=1&px=1&section=5&ave_thresh=32&_ignoreionsscorebelow=0&report=0&_sigthreshold=0.05&_msresflags=1089&_msresflags2=2&percolate=-1&percolate_rt=0&_minpeplen=5&sessionID=all_secdisabledsession) |
|  | [1058](http://10.200.41.11/mascot/cgi/peptide_view.pl?file=..%2Fdata%2F20160609%2FF081283.dat&query=1058&hit=1&index=gi%7C194403640%7Cgb%7CACF63862.1%7C&db_idx=1&px=1&section=5&ave_thresh=32&_ignoreionsscorebelow=0&report=0&_sigthreshold=0.05&_msresflags=1089&_msresflags2=2&percolate=-1&percolate_rt=0&_minpeplen=5&sessionID=all_secdisabledsession) | **531.7852** | **1061.5558** | **1061.5505** | **4.92** | **0** | **69** | **2.7e-005** | **1** | **U** | **R.LAEAAFEGVR.K** [1059](http://10.200.41.11/mascot/cgi/peptide_view.pl?file=..%2Fdata%2F20160609%2FF081283.dat&query=1059&hit=1&index=gi%7C194403640%7Cgb%7CACF63862.1%7C&db_idx=1&px=1&section=5&ave_thresh=32&_ignoreionsscorebelow=0&report=0&_sigthreshold=0.05&_msresflags=1089&_msresflags2=2&percolate=-1&percolate_rt=0&_minpeplen=5&sessionID=all_secdisabledsession) |
|  | [1228](http://10.200.41.11/mascot/cgi/peptide_view.pl?file=..%2Fdata%2F20160609%2FF081283.dat&query=1228&hit=5&index=gi%7C194403640%7Cgb%7CACF63862.1%7C&db_idx=1&px=1&section=5&ave_thresh=32&_ignoreionsscorebelow=0&report=0&_sigthreshold=0.05&_msresflags=1089&_msresflags2=2&percolate=-1&percolate_rt=0&_minpeplen=5&sessionID=all_secdisabledsession) | **558.8058** | **1115.5970** | **1115.6186** | **-19.38** | **1** | **0** | **2.6e+002** | **5** | **U** | **K.DVVATKAGDLK.A** |

### Protein sequence coverage: 4%

Matched peptides shown in ***bold red***.

| **1** | MVNDASSISR | SGYTQNPR**LA** | **EAAFEGVR**KN | TDFLKAADKA | FK**DVVATKAG** |
| --- | --- | --- | --- | --- | --- |
| **51** | **DLK**AGTKSGE | SAINTVGLKP | PTDAAREKLS | SEGQLTLLLG | KLMTLLGDVS |
| **101** | LSQLESRLAV | WQAMIESQKE | MGIQVSKEFQ | TALGEAQEAT | DLYEASIKKT |
| **151** | DTAKSVYDAA | AKKLTQAQNK | LQSLDPADPG | YAQAEAAVEQ | AGKEATEAKE |
| **201** | ALDKATDATV | KAGTDAKAKA | EKADNILTKF | QGTANAASQN | QVSQGEQDNL |
| **251** | SNVARLTMLM | AMFIEIVGKN | TEESLQNDLA | LFNALQEGRQ | AEMEKKSAEF |
| **301** | QEETRKAEET | NRIMGCIGKV | LGALLTIVSV | VAAVFTGGAS | LALAAVGLAV |
| **351** | MVADEIVKAA | TGVSFIQQAL | NPIMEHVLKP | LMELIGKAIT | KALEGLGVDK |
| **401** | KTAEMAGSIV | GAIVAAIAMV | AVIVVVAVVG | KGAAAKLGNA | LSKMMGETIK |
| **451** | KLVPNVLKQL | AQNGSK**LFTQ** | **GMQR**ITSGLG | NVGSKMGLQT | NALSKELVGN |
| **501** | TLNKVALGME | VTNTAAQSAG | GVAEGVFIKN | ASEALADFML | ARFAMDQIQQ |
| **551** | WLKQSVEIFG | ENQKVTAELQ | KAMSSAVQQN | ADASRFILRQ | SRA |

| **17.** | [gi|194404219|gb|ACF64441.1|](http://10.200.41.11/mascot/cgi/protein_view.pl?file=..%2Fdata%2F20160609%2FF081283.dat&hit=gi%7C194404219%7Cgb%7CACF64441.1%7C&db_idx=1&px=1&ave_thresh=32&_ignoreionsscorebelow=0&report=0&_sigthreshold=0.05&_msresflags=1089&_msresflags2=2&percolate=-1&percolate_rt=0&_minpeplen=5&sessionID=all_secdisabledsession)    **Mass:** 34155    **Score:** 47     **Matches:** 2(1)  **Sequences:** 1(1)  **emPAI:** 0.17 |
| --- | --- |
|  | flagellar hook-associated protein 3 [Salmonella enterica subsp. enterica serovar Newport str. SL254] |

|  | **Query** | **Observed** | **Mr(expt)** | **Mr(calc)** | **ppm** | **Miss** | **Score** | **Expect** | **Rank** | **Unique** | **Peptide** |
| --- | --- | --- | --- | --- | --- | --- | --- | --- | --- | --- | --- |
|  | [1168](http://10.200.41.11/mascot/cgi/peptide_view.pl?file=..%2Fdata%2F20160609%2FF081283.dat&query=1168&hit=1&index=gi%7C194404219%7Cgb%7CACF64441.1%7C&db_idx=1&px=1&section=5&ave_thresh=32&_ignoreionsscorebelow=0&report=0&_sigthreshold=0.05&_msresflags=1089&_msresflags2=2&percolate=-1&percolate_rt=0&_minpeplen=5&sessionID=all_secdisabledsession) | **545.7802** | **1089.5458** | **1089.5415** | **3.94** | **0** | **40** | **0.017** | **1** | **U** | **K.SVTQQVDSAR.T** [1167](http://10.200.41.11/mascot/cgi/peptide_view.pl?file=..%2Fdata%2F20160609%2FF081283.dat&query=1167&hit=1&index=gi%7C194404219%7Cgb%7CACF64441.1%7C&db_idx=1&px=1&section=5&ave_thresh=32&_ignoreionsscorebelow=0&report=0&_sigthreshold=0.05&_msresflags=1089&_msresflags2=2&percolate=-1&percolate_rt=0&_minpeplen=5&sessionID=all_secdisabledsession) |

### Protein sequence coverage: 3%

Matched peptides shown in ***bold red***.

| **1** | MRISTQMMYE | QNMSGITNSQ | AEWMKLGEQM | STGKRVTNPS | DDPIAASQAV |
| --- | --- | --- | --- | --- | --- |
| **51** | VLSQAQAQNS | QYALARTFAT | QKVSLEESVL | SQVTTAIQTA | QEKIVYAGNG |
| **101** | TLSDDDRASL | ATDLQGIRDQ | LMNLANSTDG | NGRYIFAGYK | TEAAPFDQAT |
| **151** | GGYHGGEK**SV** | **TQQVDSAR**TM | VIGHTGAQIF | NSITSNAVPE | PDGSDSEKNL |
| **201** | FVMLDTAIAA | LKTPVEGNDV | EKEKAAAAID | KTNRGLKNSL | NNVLTVRAEL |
| **251** | GTQLSELSTL | DSLGSDRALG | QKLQMSNLVD | VDWNSVISSY | VMQQAALQAS |
| **301** | YKTFTDMQGM | SLFQLNR |  |  |  |

*******************************************************************************************************************************************************

**2^nd^ technical replicate**

**Enzyme : Trypsin**

**Variable modifications :** [**Oxidation (M)**](http://10.200.41.11/mascot/cgi/client.pl?modification&mod_name=Oxidation%20%28M%29&file=..%2Fdata%2F20160609%2FF081284.dat)

**Mass values : Monoisotopic**

**Protein Mass : Unrestricted**

**Peptide Mass Tolerance : ± 20 ppm**

**Fragment Mass Tolerance: ± 0.4 Da**

**Max Missed Cleavages : 3**

**Instrument type : Default**

**Number of queries : 2813**

| **Protein hits           :** | [**gi|194401173|gb|ACF61395.1|**](http://10.200.41.11/mascot/cgi/master_results.pl?file=..%2Fdata%2F20160609%2FF081284.dat#Hit1) | flagellar hook-associated protein 2 [Salmonella enterica subsp. enterica serovar Newport str. SL254] |
| --- | --- | --- |
|  | [**gi|50830890|gb|AAT81610.1|**](http://10.200.41.11/mascot/cgi/master_results.pl?file=..%2Fdata%2F20160609%2FF081284.dat#Hit2) | phase 1 flagellin [Salmonella enterica subsp. enterica serovar Newport] |
|  | [**gi|392616945|gb|EIW99373.1|**](http://10.200.41.11/mascot/cgi/master_results.pl?file=..%2Fdata%2F20160609%2FF081284.dat#Hit3) | pathogenicity island 1 effector protein SipC [Salmonella enterica subsp. enterica serovar Newport str. Levine 15] |
|  | [**gi|194403331|gb|ACF63553.1|**](http://10.200.41.11/mascot/cgi/master_results.pl?file=..%2Fdata%2F20160609%2FF081284.dat#Hit4) | negative regulator of flagellin synthesis [Salmonella enterica subsp. enterica serovar Newport str. SL254] |
|  | [**gi|194402702|gb|ACF62924.1|**](http://10.200.41.11/mascot/cgi/master_results.pl?file=..%2Fdata%2F20160609%2FF081284.dat#Hit5) | cell invasion protein SipA [Salmonella enterica subsp. enterica serovar Newport str. SL254] |
|  | [**gi|392616944|gb|EIW99372.1|**](http://10.200.41.11/mascot/cgi/master_results.pl?file=..%2Fdata%2F20160609%2FF081284.dat#Hit6) | cell invasion protein SipD [Salmonella enterica subsp. enterica serovar Newport str. Levine 15] |
|  | [**gi|194404381|gb|ACF64603.1|**](http://10.200.41.11/mascot/cgi/master_results.pl?file=..%2Fdata%2F20160609%2FF081284.dat#Hit7) | DNA-binding protein HU-alpha [Salmonella enterica subsp. enterica serovar Newport str. SL254] |
|  | [**gi|194401878|gb|ACF62100.1|**](http://10.200.41.11/mascot/cgi/master_results.pl?file=..%2Fdata%2F20160609%2FF081284.dat#Hit8) | ribosomal protein L11 [Salmonella enterica subsp. enterica serovar Newport str. SL254] |
|  | [**gi|194404227|gb|ACF64449.1|**](http://10.200.41.11/mascot/cgi/master_results.pl?file=..%2Fdata%2F20160609%2FF081284.dat#Hit9) | ribosomal protein S5 [Salmonella enterica subsp. enterica serovar Newport str. SL254] |
|  | [**gi|194401698|gb|ACF61920.1|**](http://10.200.41.11/mascot/cgi/master_results.pl?file=..%2Fdata%2F20160609%2FF081284.dat#Hit10) | translation elongation factor Tu [Salmonella enterica subsp. enterica serovar Newport str. SL254] |
|  | [**gi|194403640|gb|ACF63862.1|**](http://10.200.41.11/mascot/cgi/master_results.pl?file=..%2Fdata%2F20160609%2FF081284.dat#Hit11) | cell invasion protein SipB [Salmonella enterica subsp. enterica serovar Newport str. SL254] |
|  | [**gi|194405415|gb|ACF65637.1|**](http://10.200.41.11/mascot/cgi/master_results.pl?file=..%2Fdata%2F20160609%2FF081284.dat#Hit12) | ribosomal protein L15 [Salmonella enterica subsp. enterica serovar Newport str. SL254] |
|  | [**gi|194401103|gb|ACF61325.1|**](http://10.200.41.11/mascot/cgi/master_results.pl?file=..%2Fdata%2F20160609%2FF081284.dat#Hit13) | ribosomal protein L6 [Salmonella enterica subsp. enterica serovar Newport str. SL254] |
|  | [**gi|446662362|ref|WP_000739708.1|**](http://10.200.41.11/mascot/cgi/master_results.pl?file=..%2Fdata%2F20160609%2FF081284.dat#Hit14) | hypothetical protein [Salmonella enterica] |
|  | [**gi|194402721|gb|ACF62943.1|**](http://10.200.41.11/mascot/cgi/master_results.pl?file=..%2Fdata%2F20160609%2FF081284.dat#Hit15) | autonomous glycyl radical cofactor [Salmonella enterica subsp. enterica serovar Newport str. SL254] |

| **1.** | [gi|194401173|gb|ACF61395.1|](http://10.200.41.11/mascot/cgi/protein_view.pl?file=..%2Fdata%2F20160609%2FF081284.dat&hit=gi%7C194401173%7Cgb%7CACF61395.1%7C&db_idx=1&px=1&ave_thresh=32&_ignoreionsscorebelow=0&report=0&_sigthreshold=0.05&_msresflags=1089&_msresflags2=2&percolate=-1&percolate_rt=0&_minpeplen=5&sessionID=all_secdisabledsession)    **Mass:** 49778    **Score:** 280    **Matches:** 5(5)  **Sequences:** 2(2)  **emPAI:** 0.23 |
| --- | --- |
|  | flagellar hook-associated protein 2 [Salmonella enterica subsp. enterica serovar Newport str. SL254] |

|  | **Query** | **Observed** | **Mr(expt)** | **Mr(calc)** | **ppm** | **Miss** | **Score** | **Expect** | **Rank** | **Unique** | **Peptide** |
| --- | --- | --- | --- | --- | --- | --- | --- | --- | --- | --- | --- |
|  | [1970](http://10.200.41.11/mascot/cgi/peptide_view.pl?file=..%2Fdata%2F20160609%2FF081284.dat&query=1970&hit=1&index=gi%7C194401173%7Cgb%7CACF61395.1%7C&db_idx=1&px=1&section=5&ave_thresh=32&_ignoreionsscorebelow=0&report=0&_sigthreshold=0.05&_msresflags=1089&_msresflags2=2&percolate=-1&percolate_rt=0&_minpeplen=5&sessionID=all_secdisabledsession) | **716.8272** | **1431.6399** | **1431.6374** | **1.72** | **0** | **59** | **4.2e-005** | **1** | **U** | **K.AQFTQLDTMMSK.L** [1969](http://10.200.41.11/mascot/cgi/peptide_view.pl?file=..%2Fdata%2F20160609%2FF081284.dat&query=1969&hit=1&index=gi%7C194401173%7Cgb%7CACF61395.1%7C&db_idx=1&px=1&section=5&ave_thresh=32&_ignoreionsscorebelow=0&report=0&_sigthreshold=0.05&_msresflags=1089&_msresflags2=2&percolate=-1&percolate_rt=0&_minpeplen=5&sessionID=all_secdisabledsession) |
|  | [2237](http://10.200.41.11/mascot/cgi/peptide_view.pl?file=..%2Fdata%2F20160609%2FF081284.dat&query=2237&hit=1&index=gi%7C194401173%7Cgb%7CACF61395.1%7C&db_idx=1&px=1&section=5&ave_thresh=32&_ignoreionsscorebelow=0&report=0&_sigthreshold=0.05&_msresflags=1089&_msresflags2=2&percolate=-1&percolate_rt=0&_minpeplen=5&sessionID=all_secdisabledsession) | **841.4247** | **1680.8349** | **1680.8319** | **1.83** | **0** | **85** | **4.8e-007** | **1** | **U** | **K.QYLSVSNSIDETVAR.Y** [2238](http://10.200.41.11/mascot/cgi/peptide_view.pl?file=..%2Fdata%2F20160609%2FF081284.dat&query=2238&hit=1&index=gi%7C194401173%7Cgb%7CACF61395.1%7C&db_idx=1&px=1&section=5&ave_thresh=32&_ignoreionsscorebelow=0&report=0&_sigthreshold=0.05&_msresflags=1089&_msresflags2=2&percolate=-1&percolate_rt=0&_minpeplen=5&sessionID=all_secdisabledsession) [2239](http://10.200.41.11/mascot/cgi/peptide_view.pl?file=..%2Fdata%2F20160609%2FF081284.dat&query=2239&hit=1&index=gi%7C194401173%7Cgb%7CACF61395.1%7C&db_idx=1&px=1&section=5&ave_thresh=32&_ignoreionsscorebelow=0&report=0&_sigthreshold=0.05&_msresflags=1089&_msresflags2=2&percolate=-1&percolate_rt=0&_minpeplen=5&sessionID=all_secdisabledsession) |

### Protein sequence coverage: 5%

Matched peptides shown in ***bold red***.

| **1** | MASISSLGVG | SNLPLDQLLT | DLTKNEKGRL | TPITKQQSAN | SAKLTAYGTL |
| --- | --- | --- | --- | --- | --- |
| **51** | KSALEKFQTA | NTALNKADLF | KSTVASSTTE | DLKVSTTAGA | AAGTYKISVT |
| **101** | QLAAAQSLAT | KTTFATTKEQ | LGDTSVTSRT | IKIEQPGRKE | PLEIKLDKGD |
| **151** | TSMEAIRDAI | NDADSGIAAS | IVKVKENEFQ | LVLTANSGTD | NTMKITVEGD |
| **201** | TKLNDLLAYD | STTNTGNMQE | LVKAENAKLN | VNGIDIERQS | NTVTDAPQGI |
| **251** | TLTLTKKVTD | ATVTVTKDDT | KAKEAIKSWV | DAYNSLVDTF | SSLTKYTAVE |
| **301** | PGEEASDKNG | ALLGDSVVRT | IQTGIRAQFA | NSGSNSAFKT | MAEIGITQDG |
| **351** | TSGKLKIDDD | KLTKVLKDNT | AAARELLVGD | GKETGITTKI | ATEVKSYLAD |
| **401** | DGIIDNAQDN | VNATLKSLTK | **QYLSVSNSID** | **ETVAR**YK**AQF** | **TQLDTMMSK**L |
| **451** | NNTSSYLTQQ | FTAMNKS |  |  |  |

| **2.** | [gi|50830890|gb|AAT81610.1|](http://10.200.41.11/mascot/cgi/protein_view.pl?file=..%2Fdata%2F20160609%2FF081284.dat&hit=gi%7C50830890%7Cgb%7CAAT81610.1%7C&db_idx=1&px=1&ave_thresh=32&_ignoreionsscorebelow=0&report=0&_sigthreshold=0.05&_msresflags=1089&_msresflags2=2&percolate=-1&percolate_rt=0&_minpeplen=5&sessionID=all_secdisabledsession)    **Mass:** 52223    **Score:** 267    **Matches:** 11(9)  **Sequences:** 3(3)  **emPAI:** 0.34 |
| --- | --- |
|  | phase 1 flagellin [Salmonella enterica subsp. enterica serovar Newport] |

|  | **Query** | **Observed** | **Mr(expt)** | **Mr(calc)** | **ppm** | **Miss** | **Score** | **Expect** | **Rank** | **Unique** | **Peptide** |
| --- | --- | --- | --- | --- | --- | --- | --- | --- | --- | --- | --- |
|  | [1168](http://10.200.41.11/mascot/cgi/peptide_view.pl?file=..%2Fdata%2F20160609%2FF081284.dat&query=1168&hit=1&index=gi%7C50830890%7Cgb%7CAAT81610.1%7C&db_idx=1&px=1&section=5&ave_thresh=32&_ignoreionsscorebelow=0&report=0&_sigthreshold=0.05&_msresflags=1089&_msresflags2=2&percolate=-1&percolate_rt=0&_minpeplen=5&sessionID=all_secdisabledsession) | **566.8026** | **1131.5907** | **1131.5884** | **2.03** | **0** | **66** | **4.6e-005** | **1** | **U** | **K.SQSALGTAIER.L** [1167](http://10.200.41.11/mascot/cgi/peptide_view.pl?file=..%2Fdata%2F20160609%2FF081284.dat&query=1167&hit=1&index=gi%7C50830890%7Cgb%7CAAT81610.1%7C&db_idx=1&px=1&section=5&ave_thresh=32&_ignoreionsscorebelow=0&report=0&_sigthreshold=0.05&_msresflags=1089&_msresflags2=2&percolate=-1&percolate_rt=0&_minpeplen=5&sessionID=all_secdisabledsession) [1169](http://10.200.41.11/mascot/cgi/peptide_view.pl?file=..%2Fdata%2F20160609%2FF081284.dat&query=1169&hit=1&index=gi%7C50830890%7Cgb%7CAAT81610.1%7C&db_idx=1&px=1&section=5&ave_thresh=32&_ignoreionsscorebelow=0&report=0&_sigthreshold=0.05&_msresflags=1089&_msresflags2=2&percolate=-1&percolate_rt=0&_minpeplen=5&sessionID=all_secdisabledsession) |
|  | [2156](http://10.200.41.11/mascot/cgi/peptide_view.pl?file=..%2Fdata%2F20160609%2FF081284.dat&query=2156&hit=1&index=gi%7C50830890%7Cgb%7CAAT81610.1%7C&db_idx=1&px=1&section=5&ave_thresh=32&_ignoreionsscorebelow=0&report=0&_sigthreshold=0.05&_msresflags=1089&_msresflags2=2&percolate=-1&percolate_rt=0&_minpeplen=5&sessionID=all_secdisabledsession) | **538.9462** | **1613.8169** | **1613.8121** | **2.95** | **1** | **36** | **0.044** | **1** | **U** | **R.INSAKDDAAGQAIANR.F** [2157](http://10.200.41.11/mascot/cgi/peptide_view.pl?file=..%2Fdata%2F20160609%2FF081284.dat&query=2157&hit=1&index=gi%7C50830890%7Cgb%7CAAT81610.1%7C&db_idx=1&px=1&section=5&ave_thresh=32&_ignoreionsscorebelow=0&report=0&_sigthreshold=0.05&_msresflags=1089&_msresflags2=2&percolate=-1&percolate_rt=0&_minpeplen=5&sessionID=all_secdisabledsession) [2158](http://10.200.41.11/mascot/cgi/peptide_view.pl?file=..%2Fdata%2F20160609%2FF081284.dat&query=2158&hit=1&index=gi%7C50830890%7Cgb%7CAAT81610.1%7C&db_idx=1&px=1&section=5&ave_thresh=32&_ignoreionsscorebelow=0&report=0&_sigthreshold=0.05&_msresflags=1089&_msresflags2=2&percolate=-1&percolate_rt=0&_minpeplen=5&sessionID=all_secdisabledsession) |
|  | [2562](http://10.200.41.11/mascot/cgi/peptide_view.pl?file=..%2Fdata%2F20160609%2FF081284.dat&query=2562&hit=1&index=gi%7C50830890%7Cgb%7CAAT81610.1%7C&db_idx=1&px=1&section=5&ave_thresh=32&_ignoreionsscorebelow=0&report=0&_sigthreshold=0.05&_msresflags=1089&_msresflags2=2&percolate=-1&percolate_rt=0&_minpeplen=5&sessionID=all_secdisabledsession) | **659.2957** | **1974.8653** | **1974.8589** | **3.26** | **1** | **40** | **0.0039** | **1** | **U** | **R.SRIEDSDYATEVSNMSR.A** [2559](http://10.200.41.11/mascot/cgi/peptide_view.pl?file=..%2Fdata%2F20160609%2FF081284.dat&query=2559&hit=1&index=gi%7C50830890%7Cgb%7CAAT81610.1%7C&db_idx=1&px=1&section=5&ave_thresh=32&_ignoreionsscorebelow=0&report=0&_sigthreshold=0.05&_msresflags=1089&_msresflags2=2&percolate=-1&percolate_rt=0&_minpeplen=5&sessionID=all_secdisabledsession) [2560](http://10.200.41.11/mascot/cgi/peptide_view.pl?file=..%2Fdata%2F20160609%2FF081284.dat&query=2560&hit=1&index=gi%7C50830890%7Cgb%7CAAT81610.1%7C&db_idx=1&px=1&section=5&ave_thresh=32&_ignoreionsscorebelow=0&report=0&_sigthreshold=0.05&_msresflags=1089&_msresflags2=2&percolate=-1&percolate_rt=0&_minpeplen=5&sessionID=all_secdisabledsession) [2561](http://10.200.41.11/mascot/cgi/peptide_view.pl?file=..%2Fdata%2F20160609%2FF081284.dat&query=2561&hit=1&index=gi%7C50830890%7Cgb%7CAAT81610.1%7C&db_idx=1&px=1&section=5&ave_thresh=32&_ignoreionsscorebelow=0&report=0&_sigthreshold=0.05&_msresflags=1089&_msresflags2=2&percolate=-1&percolate_rt=0&_minpeplen=5&sessionID=all_secdisabledsession) [2563](http://10.200.41.11/mascot/cgi/peptide_view.pl?file=..%2Fdata%2F20160609%2FF081284.dat&query=2563&hit=1&index=gi%7C50830890%7Cgb%7CAAT81610.1%7C&db_idx=1&px=1&section=5&ave_thresh=32&_ignoreionsscorebelow=0&report=0&_sigthreshold=0.05&_msresflags=1089&_msresflags2=2&percolate=-1&percolate_rt=0&_minpeplen=5&sessionID=all_secdisabledsession) |

### Protein sequence coverage: 8%

Matched peptides shown in ***bold red***.

| **1** | MAQVINTNSL | SLLTQNNLNK | **SQSALGTAIE** | **R**LSSGLR**INS** | **AKDDAAGQAI** |
| --- | --- | --- | --- | --- | --- |
| **51** | **ANR**FTANIKG | LTQASRNAND | GISIAQTTEG | ALNEINNNLQ | RVRELAVQSA |
| **101** | NSTNSQSDLD | SIQAEITQRL | NEIDRVSGQT | QFNGVKVLAQ | DNTLTIQVGA |
| **151** | NDGETIDIDL | KQINSQTLGL | DTLNVQKAYD | VSATAAMDPK | SFTDGTKNLT |
| **201** | APDATAIKAA | LGNPAATGDS | LSATLSFKDG | KYYATVAGYT | NAADTSKNGK |
| **251** | YEVNVDSATG | AVTFNAAPTK | ATVTGDTTVT | KVQVNAPVAV | STDVKKALED |
| **301** | GGVSNADATA | AKLVKMSYTD | KNGKSIDGGY | ALEAGGKYYA | ATYDEGTGKI |
| **351** | TANVTTYTDS | TGVTKTAANQ | LGGVDGKTEV | VTIDGKTYNA | SKAAGHDFKA |
| **401** | QPELAEAAAK | TTENPLAKID | AALAQVDALR | SDLGAVQNRF | NSAITNLGNT |
| **451** | VNNLSEAR**SR** | **IEDSDYATEV** | **SNMSR**AQILQ | QAGTSVLAQA | NQVPQNVLSL |
| **501** | LR |  |  |  |  |

| **3.** | [gi|392616945|gb|EIW99373.1|](http://10.200.41.11/mascot/cgi/protein_view.pl?file=..%2Fdata%2F20160609%2FF081284.dat&hit=gi%7C392616945%7Cgb%7CEIW99373.1%7C&db_idx=1&px=1&ave_thresh=32&_ignoreionsscorebelow=0&report=0&_sigthreshold=0.05&_msresflags=1089&_msresflags2=2&percolate=-1&percolate_rt=0&_minpeplen=5&sessionID=all_secdisabledsession)    **Mass:** 42957    **Score:** 256    **Matches:** 7(7)  **Sequences:** 2(2)  **emPAI:** 0.27 |
| --- | --- |
|  | pathogenicity island 1 effector protein SipC [Salmonella enterica subsp. enterica serovar Newport str. Levine 15] |

|  | **Query** | **Observed** | **Mr(expt)** | **Mr(calc)** | **ppm** | **Miss** | **Score** | **Expect** | **Rank** | **Unique** | **Peptide** |
| --- | --- | --- | --- | --- | --- | --- | --- | --- | --- | --- | --- |
|  | [738](http://10.200.41.11/mascot/cgi/peptide_view.pl?file=..%2Fdata%2F20160609%2FF081284.dat&query=738&hit=1&index=gi%7C392616945%7Cgb%7CEIW99373.1%7C&db_idx=1&px=1&section=5&ave_thresh=32&_ignoreionsscorebelow=0&report=0&_sigthreshold=0.05&_msresflags=1089&_msresflags2=2&percolate=-1&percolate_rt=0&_minpeplen=5&sessionID=all_secdisabledsession) | **494.7702** | **987.5258** | **987.5237** | **2.15** | **0** | **56** | **0.00061** | **1** | **U** | **K.LGAEGVDSLK.S** [739](http://10.200.41.11/mascot/cgi/peptide_view.pl?file=..%2Fdata%2F20160609%2FF081284.dat&query=739&hit=1&index=gi%7C392616945%7Cgb%7CEIW99373.1%7C&db_idx=1&px=1&section=5&ave_thresh=32&_ignoreionsscorebelow=0&report=0&_sigthreshold=0.05&_msresflags=1089&_msresflags2=2&percolate=-1&percolate_rt=0&_minpeplen=5&sessionID=all_secdisabledsession) [740](http://10.200.41.11/mascot/cgi/peptide_view.pl?file=..%2Fdata%2F20160609%2FF081284.dat&query=740&hit=1&index=gi%7C392616945%7Cgb%7CEIW99373.1%7C&db_idx=1&px=1&section=5&ave_thresh=32&_ignoreionsscorebelow=0&report=0&_sigthreshold=0.05&_msresflags=1089&_msresflags2=2&percolate=-1&percolate_rt=0&_minpeplen=5&sessionID=all_secdisabledsession) |
|  | [1750](http://10.200.41.11/mascot/cgi/peptide_view.pl?file=..%2Fdata%2F20160609%2FF081284.dat&query=1750&hit=1&index=gi%7C392616945%7Cgb%7CEIW99373.1%7C&db_idx=1&px=1&section=5&ave_thresh=32&_ignoreionsscorebelow=0&report=0&_sigthreshold=0.05&_msresflags=1089&_msresflags2=2&percolate=-1&percolate_rt=0&_minpeplen=5&sessionID=all_secdisabledsession) | **651.8255** | **1301.6364** | **1301.6324** | **3.13** | **0** | **82** | **7.9e-007** | **1** | **U** | **R.SEQQISQVNNR.V** [1748](http://10.200.41.11/mascot/cgi/peptide_view.pl?file=..%2Fdata%2F20160609%2FF081284.dat&query=1748&hit=1&index=gi%7C392616945%7Cgb%7CEIW99373.1%7C&db_idx=1&px=1&section=5&ave_thresh=32&_ignoreionsscorebelow=0&report=0&_sigthreshold=0.05&_msresflags=1089&_msresflags2=2&percolate=-1&percolate_rt=0&_minpeplen=5&sessionID=all_secdisabledsession) [1749](http://10.200.41.11/mascot/cgi/peptide_view.pl?file=..%2Fdata%2F20160609%2FF081284.dat&query=1749&hit=1&index=gi%7C392616945%7Cgb%7CEIW99373.1%7C&db_idx=1&px=1&section=5&ave_thresh=32&_ignoreionsscorebelow=0&report=0&_sigthreshold=0.05&_msresflags=1089&_msresflags2=2&percolate=-1&percolate_rt=0&_minpeplen=5&sessionID=all_secdisabledsession) [1751](http://10.200.41.11/mascot/cgi/peptide_view.pl?file=..%2Fdata%2F20160609%2FF081284.dat&query=1751&hit=1&index=gi%7C392616945%7Cgb%7CEIW99373.1%7C&db_idx=1&px=1&section=5&ave_thresh=32&_ignoreionsscorebelow=0&report=0&_sigthreshold=0.05&_msresflags=1089&_msresflags2=2&percolate=-1&percolate_rt=0&_minpeplen=5&sessionID=all_secdisabledsession) |

### Protein sequence coverage: 5%

Matched peptides shown in ***bold red***.

| **1** | MLISNVGINP | AAYLNNHSVE | NSSQTASQSV | SAKDILNSIG | ISSSKVSDLG |
| --- | --- | --- | --- | --- | --- |
| **51** | LSPTLSAPAP | GVLTQTPGTI | TSFLKASIQN | TDMNQDLNAL | ANNVTTKANE |
| **101** | VVQTQLREQQ | AEVGKFFDIS | GMSSSAVALL | AAANTLMLTL | NQADSKLSGK |
| **151** | LSLVSFDAAK | TTASSMMREG | MNALSGSISQ | SALQLGITGV | GAKLEYKGLQ |
| **201** | NERGALKHNA | AKIDKLTTES | HSIKNVLNGQ | NSVK**LGAEGV** | **DSLK**SLNMKK |
| **251** | TGTDATKNLN | DATLKSNAGT | SATESLGIKD | SNKQISPEHQ | AILSKRLESV |
| **301** | ESDIRLEQNT | MDMTRIDARK | MQMTGDLIMK | NSVTVGGIAG | ASGQYAATQE |
| **351** | R**SEQQISQVN** | **NR**VASTASDE | ARESSRKSTS | LIQEMLKTME | SINQSKASAL |
| **401** | AAIAGNIRA |  |  |  |  |

| **4.** | [gi|194403331|gb|ACF63553.1|](http://10.200.41.11/mascot/cgi/protein_view.pl?file=..%2Fdata%2F20160609%2FF081284.dat&hit=gi%7C194403331%7Cgb%7CACF63553.1%7C&db_idx=1&px=1&ave_thresh=32&_ignoreionsscorebelow=0&report=0&_sigthreshold=0.05&_msresflags=1089&_msresflags2=2&percolate=-1&percolate_rt=0&_minpeplen=5&sessionID=all_secdisabledsession)    **Mass:** 10561    **Score:** 244    **Matches:** 7(7)  **Sequences:** 2(2)  **emPAI:** 1.54 |
| --- | --- |
|  | negative regulator of flagellin synthesis [Salmonella enterica subsp. enterica serovar Newport str. SL254] |

|  | **Query** | **Observed** | **Mr(expt)** | **Mr(calc)** | **ppm** | **Miss** | **Score** | **Expect** | **Rank** | **Unique** | **Peptide** |
| --- | --- | --- | --- | --- | --- | --- | --- | --- | --- | --- | --- |
|  | [924](http://10.200.41.11/mascot/cgi/peptide_view.pl?file=..%2Fdata%2F20160609%2FF081284.dat&query=924&hit=1&index=gi%7C194403331%7Cgb%7CACF63553.1%7C&db_idx=1&px=1&section=5&ave_thresh=32&_ignoreionsscorebelow=0&report=0&_sigthreshold=0.05&_msresflags=1089&_msresflags2=2&percolate=-1&percolate_rt=0&_minpeplen=5&sessionID=all_secdisabledsession) | **527.2657** | **1052.5169** | **1052.5138** | **2.98** | **0** | **53** | **0.00061** | **1** | **U** | **R.EAQSYLQSK.-** [922](http://10.200.41.11/mascot/cgi/peptide_view.pl?file=..%2Fdata%2F20160609%2FF081284.dat&query=922&hit=1&index=gi%7C194403331%7Cgb%7CACF63553.1%7C&db_idx=1&px=1&section=5&ave_thresh=32&_ignoreionsscorebelow=0&report=0&_sigthreshold=0.05&_msresflags=1089&_msresflags2=2&percolate=-1&percolate_rt=0&_minpeplen=5&sessionID=all_secdisabledsession) [923](http://10.200.41.11/mascot/cgi/peptide_view.pl?file=..%2Fdata%2F20160609%2FF081284.dat&query=923&hit=1&index=gi%7C194403331%7Cgb%7CACF63553.1%7C&db_idx=1&px=1&section=5&ave_thresh=32&_ignoreionsscorebelow=0&report=0&_sigthreshold=0.05&_msresflags=1089&_msresflags2=2&percolate=-1&percolate_rt=0&_minpeplen=5&sessionID=all_secdisabledsession) [925](http://10.200.41.11/mascot/cgi/peptide_view.pl?file=..%2Fdata%2F20160609%2FF081284.dat&query=925&hit=1&index=gi%7C194403331%7Cgb%7CACF63553.1%7C&db_idx=1&px=1&section=5&ave_thresh=32&_ignoreionsscorebelow=0&report=0&_sigthreshold=0.05&_msresflags=1089&_msresflags2=2&percolate=-1&percolate_rt=0&_minpeplen=5&sessionID=all_secdisabledsession) |
|  | [2155](http://10.200.41.11/mascot/cgi/peptide_view.pl?file=..%2Fdata%2F20160609%2FF081284.dat&query=2155&hit=1&index=gi%7C194403331%7Cgb%7CACF63553.1%7C&db_idx=1&px=1&section=5&ave_thresh=32&_ignoreionsscorebelow=0&report=0&_sigthreshold=0.05&_msresflags=1089&_msresflags2=2&percolate=-1&percolate_rt=0&_minpeplen=5&sessionID=all_secdisabledsession) | **804.9086** | **1607.8026** | **1607.8002** | **1.46** | **0** | **76** | **4.3e-006** | **1** | **U** | **K.TSAATSASVTLSDAQAK.L** [2153](http://10.200.41.11/mascot/cgi/peptide_view.pl?file=..%2Fdata%2F20160609%2FF081284.dat&query=2153&hit=1&index=gi%7C194403331%7Cgb%7CACF63553.1%7C&db_idx=1&px=1&section=5&ave_thresh=32&_ignoreionsscorebelow=0&report=0&_sigthreshold=0.05&_msresflags=1089&_msresflags2=2&percolate=-1&percolate_rt=0&_minpeplen=5&sessionID=all_secdisabledsession) [2154](http://10.200.41.11/mascot/cgi/peptide_view.pl?file=..%2Fdata%2F20160609%2FF081284.dat&query=2154&hit=1&index=gi%7C194403331%7Cgb%7CACF63553.1%7C&db_idx=1&px=1&section=5&ave_thresh=32&_ignoreionsscorebelow=0&report=0&_sigthreshold=0.05&_msresflags=1089&_msresflags2=2&percolate=-1&percolate_rt=0&_minpeplen=5&sessionID=all_secdisabledsession) |

### Protein sequence coverage: 26%

Matched peptides shown in ***bold red***.

| **1** | MSIDRTSPLK | PVSTVQTRET | SDTPVQKTRQ | EK**TSAATSAS** | **VTLSDAQAK**L |
| --- | --- | --- | --- | --- | --- |
| **51** | MQPGVSDINM | ERVEALKTAI | RNGELKMDTG | KIADSLIR**EA** | **QSYLQSK** |

| **5.** | [gi|194402702|gb|ACF62924.1|](http://10.200.41.11/mascot/cgi/protein_view.pl?file=..%2Fdata%2F20160609%2FF081284.dat&hit=gi%7C194402702%7Cgb%7CACF62924.1%7C&db_idx=1&px=1&ave_thresh=32&_ignoreionsscorebelow=0&report=0&_sigthreshold=0.05&_msresflags=1089&_msresflags2=2&percolate=-1&percolate_rt=0&_minpeplen=5&sessionID=all_secdisabledsession)    **Mass:** 72333    **Score:** 242    **Matches:** 11(7)  **Sequences:** 3(3)  **emPAI:** 0.24 |
| --- | --- |
|  | cell invasion protein SipA [Salmonella enterica subsp. enterica serovar Newport str. SL254] |

|  | **Query** | **Observed** | **Mr(expt)** | **Mr(calc)** | **ppm** | **Miss** | **Score** | **Expect** | **Rank** | **Unique** | **Peptide** |
| --- | --- | --- | --- | --- | --- | --- | --- | --- | --- | --- | --- |
|  | [698](http://10.200.41.11/mascot/cgi/peptide_view.pl?file=..%2Fdata%2F20160609%2FF081284.dat&query=698&hit=1&index=gi%7C194402702%7Cgb%7CACF62924.1%7C&db_idx=1&px=1&section=5&ave_thresh=32&_ignoreionsscorebelow=0&report=0&_sigthreshold=0.05&_msresflags=1089&_msresflags2=2&percolate=-1&percolate_rt=0&_minpeplen=5&sessionID=all_secdisabledsession) | **490.7446** | **979.4747** | **979.4723** | **2.45** | **0** | **48** | **0.002** | **1** | **U** | **R.TFIDNSQR.N** [696](http://10.200.41.11/mascot/cgi/peptide_view.pl?file=..%2Fdata%2F20160609%2FF081284.dat&query=696&hit=1&index=gi%7C194402702%7Cgb%7CACF62924.1%7C&db_idx=1&px=1&section=5&ave_thresh=32&_ignoreionsscorebelow=0&report=0&_sigthreshold=0.05&_msresflags=1089&_msresflags2=2&percolate=-1&percolate_rt=0&_minpeplen=5&sessionID=all_secdisabledsession) [697](http://10.200.41.11/mascot/cgi/peptide_view.pl?file=..%2Fdata%2F20160609%2FF081284.dat&query=697&hit=1&index=gi%7C194402702%7Cgb%7CACF62924.1%7C&db_idx=1&px=1&section=5&ave_thresh=32&_ignoreionsscorebelow=0&report=0&_sigthreshold=0.05&_msresflags=1089&_msresflags2=2&percolate=-1&percolate_rt=0&_minpeplen=5&sessionID=all_secdisabledsession) [699](http://10.200.41.11/mascot/cgi/peptide_view.pl?file=..%2Fdata%2F20160609%2FF081284.dat&query=699&hit=1&index=gi%7C194402702%7Cgb%7CACF62924.1%7C&db_idx=1&px=1&section=5&ave_thresh=32&_ignoreionsscorebelow=0&report=0&_sigthreshold=0.05&_msresflags=1089&_msresflags2=2&percolate=-1&percolate_rt=0&_minpeplen=5&sessionID=all_secdisabledsession) [700](http://10.200.41.11/mascot/cgi/peptide_view.pl?file=..%2Fdata%2F20160609%2FF081284.dat&query=700&hit=1&index=gi%7C194402702%7Cgb%7CACF62924.1%7C&db_idx=1&px=1&section=5&ave_thresh=32&_ignoreionsscorebelow=0&report=0&_sigthreshold=0.05&_msresflags=1089&_msresflags2=2&percolate=-1&percolate_rt=0&_minpeplen=5&sessionID=all_secdisabledsession) |
|  | [1362](http://10.200.41.11/mascot/cgi/peptide_view.pl?file=..%2Fdata%2F20160609%2FF081284.dat&query=1362&hit=1&index=gi%7C194402702%7Cgb%7CACF62924.1%7C&db_idx=1&px=1&section=5&ave_thresh=32&_ignoreionsscorebelow=0&report=0&_sigthreshold=0.05&_msresflags=1089&_msresflags2=2&percolate=-1&percolate_rt=0&_minpeplen=5&sessionID=all_secdisabledsession) | **588.3061** | **1174.5976** | **1174.5942** | **2.90** | **0** | **64** | **9e-005** | **1** | **U** | **K.LTQEQGTSVGR.E** [1363](http://10.200.41.11/mascot/cgi/peptide_view.pl?file=..%2Fdata%2F20160609%2FF081284.dat&query=1363&hit=1&index=gi%7C194402702%7Cgb%7CACF62924.1%7C&db_idx=1&px=1&section=5&ave_thresh=32&_ignoreionsscorebelow=0&report=0&_sigthreshold=0.05&_msresflags=1089&_msresflags2=2&percolate=-1&percolate_rt=0&_minpeplen=5&sessionID=all_secdisabledsession) [1364](http://10.200.41.11/mascot/cgi/peptide_view.pl?file=..%2Fdata%2F20160609%2FF081284.dat&query=1364&hit=1&index=gi%7C194402702%7Cgb%7CACF62924.1%7C&db_idx=1&px=1&section=5&ave_thresh=32&_ignoreionsscorebelow=0&report=0&_sigthreshold=0.05&_msresflags=1089&_msresflags2=2&percolate=-1&percolate_rt=0&_minpeplen=5&sessionID=all_secdisabledsession) |
|  | [2792](http://10.200.41.11/mascot/cgi/peptide_view.pl?file=..%2Fdata%2F20160609%2FF081284.dat&query=2792&hit=1&index=gi%7C194402702%7Cgb%7CACF62924.1%7C&db_idx=1&px=1&section=5&ave_thresh=32&_ignoreionsscorebelow=0&report=0&_sigthreshold=0.05&_msresflags=1089&_msresflags2=2&percolate=-1&percolate_rt=0&_minpeplen=5&sessionID=all_secdisabledsession) | **825.3995** | **2473.1768** | **2473.1708** | **2.41** | **1** | **43** | **0.0054** | **1** | **U** | **K.VVTSEKGTTGETTSFDEVDGVTSK.S** [2790](http://10.200.41.11/mascot/cgi/peptide_view.pl?file=..%2Fdata%2F20160609%2FF081284.dat&query=2790&hit=1&index=gi%7C194402702%7Cgb%7CACF62924.1%7C&db_idx=1&px=1&section=5&ave_thresh=32&_ignoreionsscorebelow=0&report=0&_sigthreshold=0.05&_msresflags=1089&_msresflags2=2&percolate=-1&percolate_rt=0&_minpeplen=5&sessionID=all_secdisabledsession) [2791](http://10.200.41.11/mascot/cgi/peptide_view.pl?file=..%2Fdata%2F20160609%2FF081284.dat&query=2791&hit=1&index=gi%7C194402702%7Cgb%7CACF62924.1%7C&db_idx=1&px=1&section=5&ave_thresh=32&_ignoreionsscorebelow=0&report=0&_sigthreshold=0.05&_msresflags=1089&_msresflags2=2&percolate=-1&percolate_rt=0&_minpeplen=5&sessionID=all_secdisabledsession) |

### Protein sequence coverage: 6%

Matched peptides shown in ***bold red***.

| **1** | MQTEIKTQAT | NLAANLSAVR | ESATTTLSGE | IKGPQLEDFP | ALIKQASLDA |
| --- | --- | --- | --- | --- | --- |
| **51** | LFKCGKDAEA | LKEVFTNSNN | VAGKKAIMEF | AGLFRSALNA | TSDSPEAKTL |
| **101** | LMKVGAEYTA | QIIKDGLKEK | SAFGPWLPET | KKAEAKLENL | EKQLLDIIKN |
| **151** | NTGGELSKLS | TNLVMQEVMP | YIASCIEHNF | GCTLDPLTRS | NLTHLVDKAA |
| **201** | AKAVEALDMC | HQK**LTQEQGT** | **SVGR**EARHLE | MQTLIPLLLR | NVFAQIPADK |
| **251** | LPDPKIPEPA | AGPVPDGGKK | AEPTGININI | NIDSSNHSVD | NSKHINNSRS |
| **301** | HVDNSQRHID | NSNHDNSRKT | IDNSR**TFIDN** | **SQR**NGESHHS | TNSSNVSHSH |
| **351** | SRVDSTTHQT | ETAHSASTGA | IDHGIAGKID | VTAHATAEAV | TNASSESKDG |
| **401** | K**VVTSEKGTT** | **GETTSFDEVD** | **GVTSK**SIIGK | PVQATVHGVD | DNKQQSQTAE |
| **451** | IVNVKPLASQ | LAGVENVKTD | TLQSDTTVIT | GNKAGTTDND | NSQTDKTGPF |
| **501** | SGLKFKQNSF | LSTVPSVTNM | HSMHFDARET | FLGVIRKALE | PDTSTPFPVR |
| **551** | RAFDGLRAEI | LPNDTIKSAA | LKAQCSDIDK | HPELKAKMET | LKEVITHHPQ |
| **601** | KEKLAEIALQ | FAREAGLTRL | KGETDYVLSN | VLDGLIGDGS | WRAGPAYESY |
| **651** | LNKPGVDRVI | TTVDGLHMQR |  |  |  |

| **6.** | [gi|392616944|gb|EIW99372.1|](http://10.200.41.11/mascot/cgi/protein_view.pl?file=..%2Fdata%2F20160609%2FF081284.dat&hit=gi%7C392616944%7Cgb%7CEIW99372.1%7C&db_idx=1&px=1&ave_thresh=32&_ignoreionsscorebelow=0&report=0&_sigthreshold=0.05&_msresflags=1089&_msresflags2=2&percolate=-1&percolate_rt=0&_minpeplen=5&sessionID=all_secdisabledsession)    **Mass:** 37081    **Score:** 195    **Matches:** 5(4)  **Sequences:** 2(2)  **emPAI:** 0.32 |
| --- | --- |
|  | cell invasion protein SipD [Salmonella enterica subsp. enterica serovar Newport str. Levine 15] |

|  | **Query** | **Observed** | **Mr(expt)** | **Mr(calc)** | **ppm** | **Miss** | **Score** | **Expect** | **Rank** | **Unique** | **Peptide** |
| --- | --- | --- | --- | --- | --- | --- | --- | --- | --- | --- | --- |
|  | [1598](http://10.200.41.11/mascot/cgi/peptide_view.pl?file=..%2Fdata%2F20160609%2FF081284.dat&query=1598&hit=1&index=gi%7C392616944%7Cgb%7CEIW99372.1%7C&db_idx=1&px=1&section=5&ave_thresh=32&_ignoreionsscorebelow=0&report=0&_sigthreshold=0.05&_msresflags=1089&_msresflags2=2&percolate=-1&percolate_rt=0&_minpeplen=5&sessionID=all_secdisabledsession) | **637.3606** | **1272.7066** | **1272.7037** | **2.28** | **0** | **39** | **0.019** | **1** | **U** | **R.QQLTSSLNALAK.S** [1599](http://10.200.41.11/mascot/cgi/peptide_view.pl?file=..%2Fdata%2F20160609%2FF081284.dat&query=1599&hit=1&index=gi%7C392616944%7Cgb%7CEIW99372.1%7C&db_idx=1&px=1&section=5&ave_thresh=32&_ignoreionsscorebelow=0&report=0&_sigthreshold=0.05&_msresflags=1089&_msresflags2=2&percolate=-1&percolate_rt=0&_minpeplen=5&sessionID=all_secdisabledsession) |
|  | [2045](http://10.200.41.11/mascot/cgi/peptide_view.pl?file=..%2Fdata%2F20160609%2FF081284.dat&query=2045&hit=1&index=gi%7C392616944%7Cgb%7CEIW99372.1%7C&db_idx=1&px=1&section=5&ave_thresh=32&_ignoreionsscorebelow=0&report=0&_sigthreshold=0.05&_msresflags=1089&_msresflags2=2&percolate=-1&percolate_rt=0&_minpeplen=5&sessionID=all_secdisabledsession) | **752.3750** | **1502.7354** | **1502.7325** | **1.98** | **0** | **67** | **2.7e-005** | **1** | **U** | **K.SGVSLSAEQNENLR.S** [2044](http://10.200.41.11/mascot/cgi/peptide_view.pl?file=..%2Fdata%2F20160609%2FF081284.dat&query=2044&hit=1&index=gi%7C392616944%7Cgb%7CEIW99372.1%7C&db_idx=1&px=1&section=5&ave_thresh=32&_ignoreionsscorebelow=0&report=0&_sigthreshold=0.05&_msresflags=1089&_msresflags2=2&percolate=-1&percolate_rt=0&_minpeplen=5&sessionID=all_secdisabledsession) [2046](http://10.200.41.11/mascot/cgi/peptide_view.pl?file=..%2Fdata%2F20160609%2FF081284.dat&query=2046&hit=1&index=gi%7C392616944%7Cgb%7CEIW99372.1%7C&db_idx=1&px=1&section=5&ave_thresh=32&_ignoreionsscorebelow=0&report=0&_sigthreshold=0.05&_msresflags=1089&_msresflags2=2&percolate=-1&percolate_rt=0&_minpeplen=5&sessionID=all_secdisabledsession) |

### Protein sequence coverage: 7%

Matched peptides shown in ***bold red***.

| **1** | MLNIQNYSAS | PHPGIVAERP | QTPSASEHVE | TAVVPSTTEH | RGTDIISLSQ |
| --- | --- | --- | --- | --- | --- |
| **51** | AATKIQQAQQ | TLQSTPPISE | ENNDERTLAR | **QQLTSSLNAL** | **AKSGVSLSAE** |
| **101** | **QNENLR**SAFS | APTSALFSAS | PMAQPRTTIS | DAEIWDMVSQ | NISAIGDSYL |
| **151** | GVYENVVAVY | TDFYQAFSDI | LSKMGGWLLP | GKDGNTVKLD | VTSLKNDLNS |
| **201** | LVNKYNQINS | NTVLFPAQSG | SGVKVATEAE | ARQWLSELNL | PNSCLKSYGS |
| **251** | GYVVTVDLTP | LQKMVQDIDG | LGAPGKDSKL | EMDNAKYQAW | QSGFKAQEEN |
| **301** | MKTTLQTLTQ | KYSNANSLYD | NLVKVLSSTI | SSSLETAKSF | LQG |

| **11.** | [gi|194403640|gb|ACF63862.1|](http://10.200.41.11/mascot/cgi/protein_view.pl?file=..%2Fdata%2F20160609%2FF081284.dat&hit=gi%7C194403640%7Cgb%7CACF63862.1%7C&db_idx=1&px=1&ave_thresh=32&_ignoreionsscorebelow=0&report=0&_sigthreshold=0.05&_msresflags=1089&_msresflags2=2&percolate=-1&percolate_rt=0&_minpeplen=5&sessionID=all_secdisabledsession)    **Mass:** 62382    **Score:** 59     **Matches:** 4(1)  **Sequences:** 2(1)  **emPAI:** 0.09 |
| --- | --- |
|  | cell invasion protein SipB [Salmonella enterica subsp. enterica serovar Newport str. SL254] |

|  | **Query** | **Observed** | **Mr(expt)** | **Mr(calc)** | **ppm** | **Miss** | **Score** | **Expect** | **Rank** | **Unique** | **Peptide** |
| --- | --- | --- | --- | --- | --- | --- | --- | --- | --- | --- | --- |
|  | [699](http://10.200.41.11/mascot/cgi/peptide_view.pl?file=..%2Fdata%2F20160609%2FF081284.dat&query=699&hit=4&index=gi%7C194403640%7Cgb%7CACF63862.1%7C&db_idx=1&px=1&section=5&ave_thresh=32&_ignoreionsscorebelow=0&report=0&_sigthreshold=0.05&_msresflags=1089&_msresflags2=2&percolate=-1&percolate_rt=0&_minpeplen=5&sessionID=all_secdisabledsession) | 490.7447 | 979.4749 | 979.4909 | -16.38 | 0 | 2 | 91 | 4 | U | K.LFTQGMQR.I [696](http://10.200.41.11/mascot/cgi/peptide_view.pl?file=..%2Fdata%2F20160609%2FF081284.dat&query=696&hit=4&index=gi%7C194403640%7Cgb%7CACF63862.1%7C&db_idx=1&px=1&section=5&ave_thresh=32&_ignoreionsscorebelow=0&report=0&_sigthreshold=0.05&_msresflags=1089&_msresflags2=2&percolate=-1&percolate_rt=0&_minpeplen=5&sessionID=all_secdisabledsession) [698](http://10.200.41.11/mascot/cgi/peptide_view.pl?file=..%2Fdata%2F20160609%2FF081284.dat&query=698&hit=5&index=gi%7C194403640%7Cgb%7CACF63862.1%7C&db_idx=1&px=1&section=5&ave_thresh=32&_ignoreionsscorebelow=0&report=0&_sigthreshold=0.05&_msresflags=1089&_msresflags2=2&percolate=-1&percolate_rt=0&_minpeplen=5&sessionID=all_secdisabledsession) |
|  | [997](http://10.200.41.11/mascot/cgi/peptide_view.pl?file=..%2Fdata%2F20160609%2FF081284.dat&query=997&hit=1&index=gi%7C194403640%7Cgb%7CACF63862.1%7C&db_idx=1&px=1&section=5&ave_thresh=32&_ignoreionsscorebelow=0&report=0&_sigthreshold=0.05&_msresflags=1089&_msresflags2=2&percolate=-1&percolate_rt=0&_minpeplen=5&sessionID=all_secdisabledsession) | **531.7836** | **1061.5527** | **1061.5505** | **2.05** | **0** | **59** | **0.00027** | **1** | **U** | **R.LAEAAFEGVR.K** |

**Protein sequence coverage: 3%**

Matched peptides shown in ***bold red***.

| **1** | MVNDASSISR | SGYTQNPR**LA** | **EAAFEGVR**KN | TDFLKAADKA | FKDVVATKAG |
| --- | --- | --- | --- | --- | --- |
| **51** | DLKAGTKSGE | SAINTVGLKP | PTDAAREKLS | SEGQLTLLLG | KLMTLLGDVS |
| **101** | LSQLESRLAV | WQAMIESQKE | MGIQVSKEFQ | TALGEAQEAT | DLYEASIKKT |
| **151** | DTAKSVYDAA | AKKLTQAQNK | LQSLDPADPG | YAQAEAAVEQ | AGKEATEAKE |
| **201** | ALDKATDATV | KAGTDAKAKA | EKADNILTKF | QGTANAASQN | QVSQGEQDNL |
| **251** | SNVARLTMLM | AMFIEIVGKN | TEESLQNDLA | LFNALQEGRQ | AEMEKKSAEF |
| **301** | QEETRKAEET | NRIMGCIGKV | LGALLTIVSV | VAAVFTGGAS | LALAAVGLAV |
| **351** | MVADEIVKAA | TGVSFIQQAL | NPIMEHVLKP | LMELIGKAIT | KALEGLGVDK |
| **401** | KTAEMAGSIV | GAIVAAIAMV | AVIVVVAVVG | KGAAAKLGNA | LSKMMGETIK |
| **451** | KLVPNVLKQL | AQNGSK**LFTQ** | **GMQR**ITSGLG | NVGSKMGLQT | NALSKELVGN |
| **501** | TLNKVALGME | VTNTAAQSAG | GVAEGVFIKN | ASEALADFML | ARFAMDQIQQ |
| **551** | WLKQSVEIFG | ENQKVTAELQ | KAMSSAVQQN | ADASRFILRQ | SRA |

*******************************************************************************************************************************************************

**3^rd^ technical replicate**

**Enzyme : Trypsin**

**Variable modifications :** [**Oxidation (M)**](http://10.200.41.11/mascot/cgi/client.pl?modification&mod_name=Oxidation%20%28M%29&file=..%2Fdata%2F20160609%2FF081285.dat)

**Mass values : Monoisotopic**

**Protein Mass : Unrestricted**

**Peptide Mass Tolerance : ± 20 ppm**

**Fragment Mass Tolerance: ± 0.4 Da**

**Max Missed Cleavages : 3**

**Instrument type : Default**

**Number of queries : 2562**

| **Protein hits           :** | [**gi|50830890|gb|AAT81610.1|**](http://10.200.41.11/mascot/cgi/master_results.pl?file=..%2Fdata%2F20160609%2FF081285.dat#Hit1) | phase 1 flagellin [Salmonella enterica subsp. enterica serovar Newport] |
| --- | --- | --- |
|  | [**gi|194401173|gb|ACF61395.1|**](http://10.200.41.11/mascot/cgi/master_results.pl?file=..%2Fdata%2F20160609%2FF081285.dat#Hit2) | flagellar hook-associated protein 2 [Salmonella enterica subsp. enterica serovar Newport str. SL254] |
|  | [**gi|194403331|gb|ACF63553.1|**](http://10.200.41.11/mascot/cgi/master_results.pl?file=..%2Fdata%2F20160609%2FF081285.dat#Hit3) | negative regulator of flagellin synthesis [Salmonella enterica subsp. enterica serovar Newport str. SL254] |
|  | [**gi|392616945|gb|EIW99373.1|**](http://10.200.41.11/mascot/cgi/master_results.pl?file=..%2Fdata%2F20160609%2FF081285.dat#Hit4) | pathogenicity island 1 effector protein SipC [Salmonella enterica subsp. enterica serovar Newport str. Levine 15] |
|  | [**gi|194402702|gb|ACF62924.1|**](http://10.200.41.11/mascot/cgi/master_results.pl?file=..%2Fdata%2F20160609%2FF081285.dat#Hit5) | cell invasion protein SipA [Salmonella enterica subsp. enterica serovar Newport str. SL254] |
|  | [**gi|392616944|gb|EIW99372.1|**](http://10.200.41.11/mascot/cgi/master_results.pl?file=..%2Fdata%2F20160609%2FF081285.dat#Hit6) | cell invasion protein SipD [Salmonella enterica subsp. enterica serovar Newport str. Levine 15] |
|  | [**gi|194403640|gb|ACF63862.1|**](http://10.200.41.11/mascot/cgi/master_results.pl?file=..%2Fdata%2F20160609%2FF081285.dat#Hit7) | cell invasion protein SipB [Salmonella enterica subsp. enterica serovar Newport str. SL254] |
|  | [**gi|194401878|gb|ACF62100.1|**](http://10.200.41.11/mascot/cgi/master_results.pl?file=..%2Fdata%2F20160609%2FF081285.dat#Hit8) | ribosomal protein L11 [Salmonella enterica subsp. enterica serovar Newport str. SL254] |
|  | [**gi|194404381|gb|ACF64603.1|**](http://10.200.41.11/mascot/cgi/master_results.pl?file=..%2Fdata%2F20160609%2FF081285.dat#Hit9) | DNA-binding protein HU-alpha [Salmonella enterica subsp. enterica serovar Newport str. SL254] |
|  | [**gi|194401698|gb|ACF61920.1|**](http://10.200.41.11/mascot/cgi/master_results.pl?file=..%2Fdata%2F20160609%2FF081285.dat#Hit10) | translation elongation factor Tu [Salmonella enterica subsp. enterica serovar Newport str. SL254] |
|  | [**gi|194404227|gb|ACF64449.1|**](http://10.200.41.11/mascot/cgi/master_results.pl?file=..%2Fdata%2F20160609%2FF081285.dat#Hit11) | ribosomal protein S5 [Salmonella enterica subsp. enterica serovar Newport str. SL254] |
|  | [**gi|194405415|gb|ACF65637.1|**](http://10.200.41.11/mascot/cgi/master_results.pl?file=..%2Fdata%2F20160609%2FF081285.dat#Hit12) | ribosomal protein L15 [Salmonella enterica subsp. enterica serovar Newport str. SL254] |
|  | [**gi|194401633|gb|ACF61855.1|**](http://10.200.41.11/mascot/cgi/master_results.pl?file=..%2Fdata%2F20160609%2FF081285.dat#Hit13) | ribosomal protein S8 [Salmonella enterica subsp. enterica serovar Newport str. SL254] |
|  | [**gi|194402721|gb|ACF62943.1|**](http://10.200.41.11/mascot/cgi/master_results.pl?file=..%2Fdata%2F20160609%2FF081285.dat#Hit14) | autonomous glycyl radical cofactor [Salmonella enterica subsp. enterica serovar Newport str. SL254] |
|  | [**gi|195630380|gb|EDX49006.1|**](http://10.200.41.11/mascot/cgi/master_results.pl?file=..%2Fdata%2F20160609%2FF081285.dat#Hit15) | protein RecT [Salmonella enterica subsp. enterica serovar Newport str. SL317] |

| **1.** | [gi|50830890|gb|AAT81610.1|](http://10.200.41.11/mascot/cgi/protein_view.pl?file=..%2Fdata%2F20160609%2FF081285.dat&hit=gi%7C50830890%7Cgb%7CAAT81610.1%7C&db_idx=1&px=1&ave_thresh=32&_ignoreionsscorebelow=0&report=0&_sigthreshold=0.05&_msresflags=1089&_msresflags2=2&percolate=-1&percolate_rt=0&_minpeplen=5&sessionID=all_secdisabledsession)    **Mass:** 52223    **Score:** 429    **Matches:** 14(13)  **Sequences:** 3(3)  **emPAI:** 0.34 |
| --- | --- |
|  | phase 1 flagellin [Salmonella enterica subsp. enterica serovar Newport] |

|  | **Query** | **Observed** | **Mr(expt)** | **Mr(calc)** | **ppm** | **Miss** | **Score** | **Expect** | **Rank** | **Unique** | **Peptide** |
| --- | --- | --- | --- | --- | --- | --- | --- | --- | --- | --- | --- |
|  | [1327](http://10.200.41.11/mascot/cgi/peptide_view.pl?file=..%2Fdata%2F20160609%2FF081285.dat&query=1327&hit=1&index=gi%7C50830890%7Cgb%7CAAT81610.1%7C&db_idx=1&px=1&section=5&ave_thresh=32&_ignoreionsscorebelow=0&report=0&_sigthreshold=0.05&_msresflags=1089&_msresflags2=2&percolate=-1&percolate_rt=0&_minpeplen=5&sessionID=all_secdisabledsession) | **566.8027** | **1131.5909** | **1131.5884** | **2.24** | **0** | **58** | **0.00033** | **1** | **U** | **K.SQSALGTAIER.L** [1328](http://10.200.41.11/mascot/cgi/peptide_view.pl?file=..%2Fdata%2F20160609%2FF081285.dat&query=1328&hit=1&index=gi%7C50830890%7Cgb%7CAAT81610.1%7C&db_idx=1&px=1&section=5&ave_thresh=32&_ignoreionsscorebelow=0&report=0&_sigthreshold=0.05&_msresflags=1089&_msresflags2=2&percolate=-1&percolate_rt=0&_minpeplen=5&sessionID=all_secdisabledsession) [1329](http://10.200.41.11/mascot/cgi/peptide_view.pl?file=..%2Fdata%2F20160609%2FF081285.dat&query=1329&hit=1&index=gi%7C50830890%7Cgb%7CAAT81610.1%7C&db_idx=1&px=1&section=5&ave_thresh=32&_ignoreionsscorebelow=0&report=0&_sigthreshold=0.05&_msresflags=1089&_msresflags2=2&percolate=-1&percolate_rt=0&_minpeplen=5&sessionID=all_secdisabledsession) [1330](http://10.200.41.11/mascot/cgi/peptide_view.pl?file=..%2Fdata%2F20160609%2FF081285.dat&query=1330&hit=1&index=gi%7C50830890%7Cgb%7CAAT81610.1%7C&db_idx=1&px=1&section=5&ave_thresh=32&_ignoreionsscorebelow=0&report=0&_sigthreshold=0.05&_msresflags=1089&_msresflags2=2&percolate=-1&percolate_rt=0&_minpeplen=5&sessionID=all_secdisabledsession) |
|  | [2485](http://10.200.41.11/mascot/cgi/peptide_view.pl?file=..%2Fdata%2F20160609%2FF081285.dat&query=2485&hit=1&index=gi%7C50830890%7Cgb%7CAAT81610.1%7C&db_idx=1&px=1&section=5&ave_thresh=32&_ignoreionsscorebelow=0&report=0&_sigthreshold=0.05&_msresflags=1089&_msresflags2=2&percolate=-1&percolate_rt=0&_minpeplen=5&sessionID=all_secdisabledsession) | **538.9458** | **1613.8156** | **1613.8121** | **2.15** | **1** | **46** | **0.0044** | **1** | **U** | **R.INSAKDDAAGQAIANR.F** [2484](http://10.200.41.11/mascot/cgi/peptide_view.pl?file=..%2Fdata%2F20160609%2FF081285.dat&query=2484&hit=1&index=gi%7C50830890%7Cgb%7CAAT81610.1%7C&db_idx=1&px=1&section=5&ave_thresh=32&_ignoreionsscorebelow=0&report=0&_sigthreshold=0.05&_msresflags=1089&_msresflags2=2&percolate=-1&percolate_rt=0&_minpeplen=5&sessionID=all_secdisabledsession) [2486](http://10.200.41.11/mascot/cgi/peptide_view.pl?file=..%2Fdata%2F20160609%2FF081285.dat&query=2486&hit=1&index=gi%7C50830890%7Cgb%7CAAT81610.1%7C&db_idx=1&px=1&section=5&ave_thresh=32&_ignoreionsscorebelow=0&report=0&_sigthreshold=0.05&_msresflags=1089&_msresflags2=2&percolate=-1&percolate_rt=0&_minpeplen=5&sessionID=all_secdisabledsession) [2487](http://10.200.41.11/mascot/cgi/peptide_view.pl?file=..%2Fdata%2F20160609%2FF081285.dat&query=2487&hit=1&index=gi%7C50830890%7Cgb%7CAAT81610.1%7C&db_idx=1&px=1&section=5&ave_thresh=32&_ignoreionsscorebelow=0&report=0&_sigthreshold=0.05&_msresflags=1089&_msresflags2=2&percolate=-1&percolate_rt=0&_minpeplen=5&sessionID=all_secdisabledsession) |
|  | [2508](http://10.200.41.11/mascot/cgi/peptide_view.pl?file=..%2Fdata%2F20160609%2FF081285.dat&query=2508&hit=1&index=gi%7C50830890%7Cgb%7CAAT81610.1%7C&db_idx=1&px=1&section=5&ave_thresh=32&_ignoreionsscorebelow=0&report=0&_sigthreshold=0.05&_msresflags=1089&_msresflags2=2&percolate=-1&percolate_rt=0&_minpeplen=5&sessionID=all_secdisabledsession) | **659.2960** | **1974.8660** | **1974.8589** | **3.63** | **1** | **61** | **3.1e-005** | **1** | **U** | **R.SRIEDSDYATEVSNMSR.A** [2503](http://10.200.41.11/mascot/cgi/peptide_view.pl?file=..%2Fdata%2F20160609%2FF081285.dat&query=2503&hit=1&index=gi%7C50830890%7Cgb%7CAAT81610.1%7C&db_idx=1&px=1&section=5&ave_thresh=32&_ignoreionsscorebelow=0&report=0&_sigthreshold=0.05&_msresflags=1089&_msresflags2=2&percolate=-1&percolate_rt=0&_minpeplen=5&sessionID=all_secdisabledsession) [2504](http://10.200.41.11/mascot/cgi/peptide_view.pl?file=..%2Fdata%2F20160609%2FF081285.dat&query=2504&hit=1&index=gi%7C50830890%7Cgb%7CAAT81610.1%7C&db_idx=1&px=1&section=5&ave_thresh=32&_ignoreionsscorebelow=0&report=0&_sigthreshold=0.05&_msresflags=1089&_msresflags2=2&percolate=-1&percolate_rt=0&_minpeplen=5&sessionID=all_secdisabledsession) [2505](http://10.200.41.11/mascot/cgi/peptide_view.pl?file=..%2Fdata%2F20160609%2FF081285.dat&query=2505&hit=1&index=gi%7C50830890%7Cgb%7CAAT81610.1%7C&db_idx=1&px=1&section=5&ave_thresh=32&_ignoreionsscorebelow=0&report=0&_sigthreshold=0.05&_msresflags=1089&_msresflags2=2&percolate=-1&percolate_rt=0&_minpeplen=5&sessionID=all_secdisabledsession) [2506](http://10.200.41.11/mascot/cgi/peptide_view.pl?file=..%2Fdata%2F20160609%2FF081285.dat&query=2506&hit=1&index=gi%7C50830890%7Cgb%7CAAT81610.1%7C&db_idx=1&px=1&section=5&ave_thresh=32&_ignoreionsscorebelow=0&report=0&_sigthreshold=0.05&_msresflags=1089&_msresflags2=2&percolate=-1&percolate_rt=0&_minpeplen=5&sessionID=all_secdisabledsession) [2507](http://10.200.41.11/mascot/cgi/peptide_view.pl?file=..%2Fdata%2F20160609%2FF081285.dat&query=2507&hit=1&index=gi%7C50830890%7Cgb%7CAAT81610.1%7C&db_idx=1&px=1&section=5&ave_thresh=32&_ignoreionsscorebelow=0&report=0&_sigthreshold=0.05&_msresflags=1089&_msresflags2=2&percolate=-1&percolate_rt=0&_minpeplen=5&sessionID=all_secdisabledsession) |

### Protein sequence coverage: 8%

Matched peptides shown in ***bold red***.

| **1** | MAQVINTNSL | SLLTQNNLNK | **SQSALGTAIE** | **R**LSSGLR**INS** | **AKDDAAGQAI** |
| --- | --- | --- | --- | --- | --- |
| **51** | **ANR**FTANIKG | LTQASRNAND | GISIAQTTEG | ALNEINNNLQ | RVRELAVQSA |
| **101** | NSTNSQSDLD | SIQAEITQRL | NEIDRVSGQT | QFNGVKVLAQ | DNTLTIQVGA |
| **151** | NDGETIDIDL | KQINSQTLGL | DTLNVQKAYD | VSATAAMDPK | SFTDGTKNLT |
| **201** | APDATAIKAA | LGNPAATGDS | LSATLSFKDG | KYYATVAGYT | NAADTSKNGK |
| **251** | YEVNVDSATG | AVTFNAAPTK | ATVTGDTTVT | KVQVNAPVAV | STDVKKALED |
| **301** | GGVSNADATA | AKLVKMSYTD | KNGKSIDGGY | ALEAGGKYYA | ATYDEGTGKI |
| **351** | TANVTTYTDS | TGVTKTAANQ | LGGVDGKTEV | VTIDGKTYNA | SKAAGHDFKA |
| **401** | QPELAEAAAK | TTENPLAKID | AALAQVDALR | SDLGAVQNRF | NSAITNLGNT |
| **451** | VNNLSEAR**SR** | **IEDSDYATEV** | **SNMSR**AQILQ | QAGTSVLAQA | NQVPQNVLSL |
| **501** | LR |  |  |  |  |

| **2.** | [gi|194401173|gb|ACF61395.1|](http://10.200.41.11/mascot/cgi/protein_view.pl?file=..%2Fdata%2F20160609%2FF081285.dat&hit=gi%7C194401173%7Cgb%7CACF61395.1%7C&db_idx=1&px=1&ave_thresh=32&_ignoreionsscorebelow=0&report=0&_sigthreshold=0.05&_msresflags=1089&_msresflags2=2&percolate=-1&percolate_rt=0&_minpeplen=5&sessionID=all_secdisabledsession)    **Mass:** 49778    **Score:** 358    **Matches:** 6(6)  **Sequences:** 2(2)  **emPAI:** 0.23 |
| --- | --- |
|  | flagellar hook-associated protein 2 [Salmonella enterica subsp. enterica serovar Newport str. SL254] |

|  | **Query** | **Observed** | **Mr(expt)** | **Mr(calc)** | **ppm** | **Miss** | **Score** | **Expect** | **Rank** | **Unique** | **Peptide** |
| --- | --- | --- | --- | --- | --- | --- | --- | --- | --- | --- | --- |
|  | [2304](http://10.200.41.11/mascot/cgi/peptide_view.pl?file=..%2Fdata%2F20160609%2FF081285.dat&query=2304&hit=1&index=gi%7C194401173%7Cgb%7CACF61395.1%7C&db_idx=1&px=1&section=5&ave_thresh=32&_ignoreionsscorebelow=0&report=0&_sigthreshold=0.05&_msresflags=1089&_msresflags2=2&percolate=-1&percolate_rt=0&_minpeplen=5&sessionID=all_secdisabledsession) | **716.8280** | **1431.6415** | **1431.6374** | **2.83** | **0** | **45** | **0.0012** | **1** | **U** | **K.AQFTQLDTMMSK.L** [2303](http://10.200.41.11/mascot/cgi/peptide_view.pl?file=..%2Fdata%2F20160609%2FF081285.dat&query=2303&hit=1&index=gi%7C194401173%7Cgb%7CACF61395.1%7C&db_idx=1&px=1&section=5&ave_thresh=32&_ignoreionsscorebelow=0&report=0&_sigthreshold=0.05&_msresflags=1089&_msresflags2=2&percolate=-1&percolate_rt=0&_minpeplen=5&sessionID=all_secdisabledsession) |
|  | [2502](http://10.200.41.11/mascot/cgi/peptide_view.pl?file=..%2Fdata%2F20160609%2FF081285.dat&query=2502&hit=1&index=gi%7C194401173%7Cgb%7CACF61395.1%7C&db_idx=1&px=1&section=5&ave_thresh=32&_ignoreionsscorebelow=0&report=0&_sigthreshold=0.05&_msresflags=1089&_msresflags2=2&percolate=-1&percolate_rt=0&_minpeplen=5&sessionID=all_secdisabledsession) | **841.4256** | **1680.8366** | **1680.8319** | **2.84** | **0** | **108** | **2.6e-009** | **1** | **U** | **K.QYLSVSNSIDETVAR.Y** [2499](http://10.200.41.11/mascot/cgi/peptide_view.pl?file=..%2Fdata%2F20160609%2FF081285.dat&query=2499&hit=1&index=gi%7C194401173%7Cgb%7CACF61395.1%7C&db_idx=1&px=1&section=5&ave_thresh=32&_ignoreionsscorebelow=0&report=0&_sigthreshold=0.05&_msresflags=1089&_msresflags2=2&percolate=-1&percolate_rt=0&_minpeplen=5&sessionID=all_secdisabledsession) [2500](http://10.200.41.11/mascot/cgi/peptide_view.pl?file=..%2Fdata%2F20160609%2FF081285.dat&query=2500&hit=1&index=gi%7C194401173%7Cgb%7CACF61395.1%7C&db_idx=1&px=1&section=5&ave_thresh=32&_ignoreionsscorebelow=0&report=0&_sigthreshold=0.05&_msresflags=1089&_msresflags2=2&percolate=-1&percolate_rt=0&_minpeplen=5&sessionID=all_secdisabledsession) [2501](http://10.200.41.11/mascot/cgi/peptide_view.pl?file=..%2Fdata%2F20160609%2FF081285.dat&query=2501&hit=1&index=gi%7C194401173%7Cgb%7CACF61395.1%7C&db_idx=1&px=1&section=5&ave_thresh=32&_ignoreionsscorebelow=0&report=0&_sigthreshold=0.05&_msresflags=1089&_msresflags2=2&percolate=-1&percolate_rt=0&_minpeplen=5&sessionID=all_secdisabledsession) |

### Protein sequence coverage: 5%

Matched peptides shown in ***bold red***.

| **1** | MASISSLGVG | SNLPLDQLLT | DLTKNEKGRL | TPITKQQSAN | SAKLTAYGTL |
| --- | --- | --- | --- | --- | --- |
| **51** | KSALEKFQTA | NTALNKADLF | KSTVASSTTE | DLKVSTTAGA | AAGTYKISVT |
| **101** | QLAAAQSLAT | KTTFATTKEQ | LGDTSVTSRT | IKIEQPGRKE | PLEIKLDKGD |
| **151** | TSMEAIRDAI | NDADSGIAAS | IVKVKENEFQ | LVLTANSGTD | NTMKITVEGD |
| **201** | TKLNDLLAYD | STTNTGNMQE | LVKAENAKLN | VNGIDIERQS | NTVTDAPQGI |
| **251** | TLTLTKKVTD | ATVTVTKDDT | KAKEAIKSWV | DAYNSLVDTF | SSLTKYTAVE |
| **301** | PGEEASDKNG | ALLGDSVVRT | IQTGIRAQFA | NSGSNSAFKT | MAEIGITQDG |
| **351** | TSGKLKIDDD | KLTKVLKDNT | AAARELLVGD | GKETGITTKI | ATEVKSYLAD |
| **401** | DGIIDNAQDN | VNATLKSLTK | **QYLSVSNSID** | **ETVAR**YK**AQF** | **TQLDTMMSK**L |
| **451** | NNTSSYLTQQ | FTAMNKS |  |  |  |

| **3.** | [gi|194403331|gb|ACF63553.1|](http://10.200.41.11/mascot/cgi/protein_view.pl?file=..%2Fdata%2F20160609%2FF081285.dat&hit=gi%7C194403331%7Cgb%7CACF63553.1%7C&db_idx=1&px=1&ave_thresh=32&_ignoreionsscorebelow=0&report=0&_sigthreshold=0.05&_msresflags=1089&_msresflags2=2&percolate=-1&percolate_rt=0&_minpeplen=5&sessionID=all_secdisabledsession)    **Mass:** 10561    **Score:** 337    **Matches:** 13(9)  **Sequences:** 3(2)  **emPAI:** 1.54 |
| --- | --- |
|  | negative regulator of flagellin synthesis [Salmonella enterica subsp. enterica serovar Newport str. SL254] |

|  | **Query** | **Observed** | **Mr(expt)** | **Mr(calc)** | **ppm** | **Miss** | **Score** | **Expect** | **Rank** | **Unique** | **Peptide** |
| --- | --- | --- | --- | --- | --- | --- | --- | --- | --- | --- | --- |
|  | [988](http://10.200.41.11/mascot/cgi/peptide_view.pl?file=..%2Fdata%2F20160609%2FF081285.dat&query=988&hit=1&index=gi%7C194403331%7Cgb%7CACF63553.1%7C&db_idx=1&px=1&section=5&ave_thresh=32&_ignoreionsscorebelow=0&report=0&_sigthreshold=0.05&_msresflags=1089&_msresflags2=2&percolate=-1&percolate_rt=0&_minpeplen=5&sessionID=all_secdisabledsession) | **527.2664** | **1052.5183** | **1052.5138** | **4.25** | **0** | **39** | **0.014** | **1** | **U** | **R.EAQSYLQSK.-** [983](http://10.200.41.11/mascot/cgi/peptide_view.pl?file=..%2Fdata%2F20160609%2FF081285.dat&query=983&hit=1&index=gi%7C194403331%7Cgb%7CACF63553.1%7C&db_idx=1&px=1&section=5&ave_thresh=32&_ignoreionsscorebelow=0&report=0&_sigthreshold=0.05&_msresflags=1089&_msresflags2=2&percolate=-1&percolate_rt=0&_minpeplen=5&sessionID=all_secdisabledsession) [984](http://10.200.41.11/mascot/cgi/peptide_view.pl?file=..%2Fdata%2F20160609%2FF081285.dat&query=984&hit=1&index=gi%7C194403331%7Cgb%7CACF63553.1%7C&db_idx=1&px=1&section=5&ave_thresh=32&_ignoreionsscorebelow=0&report=0&_sigthreshold=0.05&_msresflags=1089&_msresflags2=2&percolate=-1&percolate_rt=0&_minpeplen=5&sessionID=all_secdisabledsession) [985](http://10.200.41.11/mascot/cgi/peptide_view.pl?file=..%2Fdata%2F20160609%2FF081285.dat&query=985&hit=1&index=gi%7C194403331%7Cgb%7CACF63553.1%7C&db_idx=1&px=1&section=5&ave_thresh=32&_ignoreionsscorebelow=0&report=0&_sigthreshold=0.05&_msresflags=1089&_msresflags2=2&percolate=-1&percolate_rt=0&_minpeplen=5&sessionID=all_secdisabledsession) [986](http://10.200.41.11/mascot/cgi/peptide_view.pl?file=..%2Fdata%2F20160609%2FF081285.dat&query=986&hit=1&index=gi%7C194403331%7Cgb%7CACF63553.1%7C&db_idx=1&px=1&section=5&ave_thresh=32&_ignoreionsscorebelow=0&report=0&_sigthreshold=0.05&_msresflags=1089&_msresflags2=2&percolate=-1&percolate_rt=0&_minpeplen=5&sessionID=all_secdisabledsession) [987](http://10.200.41.11/mascot/cgi/peptide_view.pl?file=..%2Fdata%2F20160609%2FF081285.dat&query=987&hit=1&index=gi%7C194403331%7Cgb%7CACF63553.1%7C&db_idx=1&px=1&section=5&ave_thresh=32&_ignoreionsscorebelow=0&report=0&_sigthreshold=0.05&_msresflags=1089&_msresflags2=2&percolate=-1&percolate_rt=0&_minpeplen=5&sessionID=all_secdisabledsession) |
|  | [2264](http://10.200.41.11/mascot/cgi/peptide_view.pl?file=..%2Fdata%2F20160609%2FF081285.dat&query=2264&hit=1&index=gi%7C194403331%7Cgb%7CACF63553.1%7C&db_idx=1&px=1&section=5&ave_thresh=32&_ignoreionsscorebelow=0&report=0&_sigthreshold=0.05&_msresflags=1089&_msresflags2=2&percolate=-1&percolate_rt=0&_minpeplen=5&sessionID=all_secdisabledsession) | **471.9418** | **1412.8035** | **1412.7987** | **3.36** | **0** | **25** | **0.41** | **1** | **U** | **R.TSPLKPVSTVQTR.E** [2265](http://10.200.41.11/mascot/cgi/peptide_view.pl?file=..%2Fdata%2F20160609%2FF081285.dat&query=2265&hit=1&index=gi%7C194403331%7Cgb%7CACF63553.1%7C&db_idx=1&px=1&section=5&ave_thresh=32&_ignoreionsscorebelow=0&report=0&_sigthreshold=0.05&_msresflags=1089&_msresflags2=2&percolate=-1&percolate_rt=0&_minpeplen=5&sessionID=all_secdisabledsession) [2266](http://10.200.41.11/mascot/cgi/peptide_view.pl?file=..%2Fdata%2F20160609%2FF081285.dat&query=2266&hit=1&index=gi%7C194403331%7Cgb%7CACF63553.1%7C&db_idx=1&px=1&section=5&ave_thresh=32&_ignoreionsscorebelow=0&report=0&_sigthreshold=0.05&_msresflags=1089&_msresflags2=2&percolate=-1&percolate_rt=0&_minpeplen=5&sessionID=all_secdisabledsession) |
|  | [2481](http://10.200.41.11/mascot/cgi/peptide_view.pl?file=..%2Fdata%2F20160609%2FF081285.dat&query=2481&hit=1&index=gi%7C194403331%7Cgb%7CACF63553.1%7C&db_idx=1&px=1&section=5&ave_thresh=32&_ignoreionsscorebelow=0&report=0&_sigthreshold=0.05&_msresflags=1089&_msresflags2=2&percolate=-1&percolate_rt=0&_minpeplen=5&sessionID=all_secdisabledsession) | **804.9088** | **1607.8031** | **1607.8002** | **1.77** | **0** | **99** | **2.4e-008** | **1** | **U** | **K.TSAATSASVTLSDAQAK.L** [2480](http://10.200.41.11/mascot/cgi/peptide_view.pl?file=..%2Fdata%2F20160609%2FF081285.dat&query=2480&hit=1&index=gi%7C194403331%7Cgb%7CACF63553.1%7C&db_idx=1&px=1&section=5&ave_thresh=32&_ignoreionsscorebelow=0&report=0&_sigthreshold=0.05&_msresflags=1089&_msresflags2=2&percolate=-1&percolate_rt=0&_minpeplen=5&sessionID=all_secdisabledsession) [2482](http://10.200.41.11/mascot/cgi/peptide_view.pl?file=..%2Fdata%2F20160609%2FF081285.dat&query=2482&hit=1&index=gi%7C194403331%7Cgb%7CACF63553.1%7C&db_idx=1&px=1&section=5&ave_thresh=32&_ignoreionsscorebelow=0&report=0&_sigthreshold=0.05&_msresflags=1089&_msresflags2=2&percolate=-1&percolate_rt=0&_minpeplen=5&sessionID=all_secdisabledsession) [2483](http://10.200.41.11/mascot/cgi/peptide_view.pl?file=..%2Fdata%2F20160609%2FF081285.dat&query=2483&hit=1&index=gi%7C194403331%7Cgb%7CACF63553.1%7C&db_idx=1&px=1&section=5&ave_thresh=32&_ignoreionsscorebelow=0&report=0&_sigthreshold=0.05&_msresflags=1089&_msresflags2=2&percolate=-1&percolate_rt=0&_minpeplen=5&sessionID=all_secdisabledsession) |

### Protein sequence coverage: 40%

Matched peptides shown in ***bold red***.

| **1** | MSIDR**TSPLK** | **PVSTVQTR**ET | SDTPVQKTRQ | EK**TSAATSAS** | **VTLSDAQAK**L |
| --- | --- | --- | --- | --- | --- |
| **51** | MQPGVSDINM | ERVEALKTAI | RNGELKMDTG | KIADSLIR**EA** | **QSYLQSK** |

| **4.** | [gi|392616945|gb|EIW99373.1|](http://10.200.41.11/mascot/cgi/protein_view.pl?file=..%2Fdata%2F20160609%2FF081285.dat&hit=gi%7C392616945%7Cgb%7CEIW99373.1%7C&db_idx=1&px=1&ave_thresh=32&_ignoreionsscorebelow=0&report=0&_sigthreshold=0.05&_msresflags=1089&_msresflags2=2&percolate=-1&percolate_rt=0&_minpeplen=5&sessionID=all_secdisabledsession)    **Mass:** 42957    **Score:** 308    **Matches:** 14(8)  **Sequences:** 4(2)  **emPAI:** 0.27 |
| --- | --- |
|  | pathogenicity island 1 effector protein SipC [Salmonella enterica subsp. enterica serovar Newport str. Levine 15] |

|  | **Query** | **Observed** | **Mr(expt)** | **Mr(calc)** | **ppm** | **Miss** | **Score** | **Expect** | **Rank** | **Unique** | **Peptide** |
| --- | --- | --- | --- | --- | --- | --- | --- | --- | --- | --- | --- |
|  | [762](http://10.200.41.11/mascot/cgi/peptide_view.pl?file=..%2Fdata%2F20160609%2FF081285.dat&query=762&hit=1&index=gi%7C392616945%7Cgb%7CEIW99373.1%7C&db_idx=1&px=1&section=5&ave_thresh=32&_ignoreionsscorebelow=0&report=0&_sigthreshold=0.05&_msresflags=1089&_msresflags2=2&percolate=-1&percolate_rt=0&_minpeplen=5&sessionID=all_secdisabledsession) | **494.7708** | **987.5271** | **987.5237** | **3.51** | **0** | **47** | **0.0048** | **1** | **U** | **K.LGAEGVDSLK.S** [763](http://10.200.41.11/mascot/cgi/peptide_view.pl?file=..%2Fdata%2F20160609%2FF081285.dat&query=763&hit=1&index=gi%7C392616945%7Cgb%7CEIW99373.1%7C&db_idx=1&px=1&section=5&ave_thresh=32&_ignoreionsscorebelow=0&report=0&_sigthreshold=0.05&_msresflags=1089&_msresflags2=2&percolate=-1&percolate_rt=0&_minpeplen=5&sessionID=all_secdisabledsession) |
|  | [1999](http://10.200.41.11/mascot/cgi/peptide_view.pl?file=..%2Fdata%2F20160609%2FF081285.dat&query=1999&hit=1&index=gi%7C392616945%7Cgb%7CEIW99373.1%7C&db_idx=1&px=1&section=5&ave_thresh=32&_ignoreionsscorebelow=0&report=0&_sigthreshold=0.05&_msresflags=1089&_msresflags2=2&percolate=-1&percolate_rt=0&_minpeplen=5&sessionID=all_secdisabledsession) | **651.8248** | **1301.6351** | **1301.6324** | **2.10** | **0** | **93** | **5.5e-008** | **1** | **U** | **R.SEQQISQVNNR.V** [2000](http://10.200.41.11/mascot/cgi/peptide_view.pl?file=..%2Fdata%2F20160609%2FF081285.dat&query=2000&hit=1&index=gi%7C392616945%7Cgb%7CEIW99373.1%7C&db_idx=1&px=1&section=5&ave_thresh=32&_ignoreionsscorebelow=0&report=0&_sigthreshold=0.05&_msresflags=1089&_msresflags2=2&percolate=-1&percolate_rt=0&_minpeplen=5&sessionID=all_secdisabledsession) [2001](http://10.200.41.11/mascot/cgi/peptide_view.pl?file=..%2Fdata%2F20160609%2FF081285.dat&query=2001&hit=1&index=gi%7C392616945%7Cgb%7CEIW99373.1%7C&db_idx=1&px=1&section=5&ave_thresh=32&_ignoreionsscorebelow=0&report=0&_sigthreshold=0.05&_msresflags=1089&_msresflags2=2&percolate=-1&percolate_rt=0&_minpeplen=5&sessionID=all_secdisabledsession) [2002](http://10.200.41.11/mascot/cgi/peptide_view.pl?file=..%2Fdata%2F20160609%2FF081285.dat&query=2002&hit=1&index=gi%7C392616945%7Cgb%7CEIW99373.1%7C&db_idx=1&px=1&section=5&ave_thresh=32&_ignoreionsscorebelow=0&report=0&_sigthreshold=0.05&_msresflags=1089&_msresflags2=2&percolate=-1&percolate_rt=0&_minpeplen=5&sessionID=all_secdisabledsession) [2003](http://10.200.41.11/mascot/cgi/peptide_view.pl?file=..%2Fdata%2F20160609%2FF081285.dat&query=2003&hit=1&index=gi%7C392616945%7Cgb%7CEIW99373.1%7C&db_idx=1&px=1&section=5&ave_thresh=32&_ignoreionsscorebelow=0&report=0&_sigthreshold=0.05&_msresflags=1089&_msresflags2=2&percolate=-1&percolate_rt=0&_minpeplen=5&sessionID=all_secdisabledsession) [2004](http://10.200.41.11/mascot/cgi/peptide_view.pl?file=..%2Fdata%2F20160609%2FF081285.dat&query=2004&hit=1&index=gi%7C392616945%7Cgb%7CEIW99373.1%7C&db_idx=1&px=1&section=5&ave_thresh=32&_ignoreionsscorebelow=0&report=0&_sigthreshold=0.05&_msresflags=1089&_msresflags2=2&percolate=-1&percolate_rt=0&_minpeplen=5&sessionID=all_secdisabledsession) |
|  | [2085](http://10.200.41.11/mascot/cgi/peptide_view.pl?file=..%2Fdata%2F20160609%2FF081285.dat&query=2085&hit=1&index=gi%7C392616945%7Cgb%7CEIW99373.1%7C&db_idx=1&px=1&section=5&ave_thresh=32&_ignoreionsscorebelow=0&report=0&_sigthreshold=0.05&_msresflags=1089&_msresflags2=2&percolate=-1&percolate_rt=0&_minpeplen=5&sessionID=all_secdisabledsession) | **448.5522** | **1342.6349** | **1342.6295** | **4.05** | **1** | **26** | **0.23** | **1** | **U** | **R.KMQMTGDLIMK.N** |
|  | [2538](http://10.200.41.11/mascot/cgi/peptide_view.pl?file=..%2Fdata%2F20160609%2FF081285.dat&query=2538&hit=1&index=gi%7C392616945%7Cgb%7CEIW99373.1%7C&db_idx=1&px=1&section=5&ave_thresh=32&_ignoreionsscorebelow=0&report=0&_sigthreshold=0.05&_msresflags=1089&_msresflags2=2&percolate=-1&percolate_rt=0&_minpeplen=5&sessionID=all_secdisabledsession) | **819.0585** | **2454.1536** | **2454.1479** | **2.33** | **2** | **16** | **3.7** | **1** | **U** | **K.RLESVESDIRLEQNTMDMTR.I** |
|  | [2540](http://10.200.41.11/mascot/cgi/peptide_view.pl?file=..%2Fdata%2F20160609%2FF081285.dat&query=2540&hit=1&index=gi%7C392616945%7Cgb%7CEIW99373.1%7C&db_idx=1&px=1&section=5&ave_thresh=32&_ignoreionsscorebelow=0&report=0&_sigthreshold=0.05&_msresflags=1089&_msresflags2=2&percolate=-1&percolate_rt=0&_minpeplen=5&sessionID=all_secdisabledsession) | **614.5458** | **2454.1540** | **2454.1479** | **2.50** | **2** | **(15)** | **4.2** | **1** | **U** | **K.RLESVESDIRLEQNTMDMTR.I** [2536](http://10.200.41.11/mascot/cgi/peptide_view.pl?file=..%2Fdata%2F20160609%2FF081285.dat&query=2536&hit=1&index=gi%7C392616945%7Cgb%7CEIW99373.1%7C&db_idx=1&px=1&section=5&ave_thresh=32&_ignoreionsscorebelow=0&report=0&_sigthreshold=0.05&_msresflags=1089&_msresflags2=2&percolate=-1&percolate_rt=0&_minpeplen=5&sessionID=all_secdisabledsession) [2537](http://10.200.41.11/mascot/cgi/peptide_view.pl?file=..%2Fdata%2F20160609%2FF081285.dat&query=2537&hit=1&index=gi%7C392616945%7Cgb%7CEIW99373.1%7C&db_idx=1&px=1&section=5&ave_thresh=32&_ignoreionsscorebelow=0&report=0&_sigthreshold=0.05&_msresflags=1089&_msresflags2=2&percolate=-1&percolate_rt=0&_minpeplen=5&sessionID=all_secdisabledsession) [2539](http://10.200.41.11/mascot/cgi/peptide_view.pl?file=..%2Fdata%2F20160609%2FF081285.dat&query=2539&hit=1&index=gi%7C392616945%7Cgb%7CEIW99373.1%7C&db_idx=1&px=1&section=5&ave_thresh=32&_ignoreionsscorebelow=0&report=0&_sigthreshold=0.05&_msresflags=1089&_msresflags2=2&percolate=-1&percolate_rt=0&_minpeplen=5&sessionID=all_secdisabledsession) |

### Protein sequence coverage: 12%

Matched peptides shown in ***bold red***.

| **1** | MLISNVGINP | AAYLNNHSVE | NSSQTASQSV | SAKDILNSIG | ISSSKVSDLG |
| --- | --- | --- | --- | --- | --- |
| **51** | LSPTLSAPAP | GVLTQTPGTI | TSFLKASIQN | TDMNQDLNAL | ANNVTTKANE |
| **101** | VVQTQLREQQ | AEVGKFFDIS | GMSSSAVALL | AAANTLMLTL | NQADSKLSGK |
| **151** | LSLVSFDAAK | TTASSMMREG | MNALSGSISQ | SALQLGITGV | GAKLEYKGLQ |
| **201** | NERGALKHNA | AKIDKLTTES | HSIKNVLNGQ | NSVK**LGAEGV** | **DSLK**SLNMKK |
| **251** | TGTDATKNLN | DATLKSNAGT | SATESLGIKD | SNKQISPEHQ | AILSK**RLESV** |
| **301** | **ESDIRLEQNT** | **MDMTR**IDAR**K** | **MQMTGDLIMK** | NSVTVGGIAG | ASGQYAATQE |
| **351** | R**SEQQISQVN** | **NR**VASTASDE | ARESSRKSTS | LIQEMLKTME | SINQSKASAL |
| **401** | AAIAGNIRA |  |  |  |  |

| **5.** | [gi|194402702|gb|ACF62924.1|](http://10.200.41.11/mascot/cgi/protein_view.pl?file=..%2Fdata%2F20160609%2FF081285.dat&hit=gi%7C194402702%7Cgb%7CACF62924.1%7C&db_idx=1&px=1&ave_thresh=32&_ignoreionsscorebelow=0&report=0&_sigthreshold=0.05&_msresflags=1089&_msresflags2=2&percolate=-1&percolate_rt=0&_minpeplen=5&sessionID=all_secdisabledsession)    **Mass:** 72333    **Score:** 254    **Matches:** 15(9)  **Sequences:** 4(2)  **emPAI:** 0.15 |
| --- | --- |
|  | cell invasion protein SipA [Salmonella enterica subsp. enterica serovar Newport str. SL254] |

|  | **Query** | **Observed** | **Mr(expt)** | **Mr(calc)** | **ppm** | **Miss** | **Score** | **Expect** | **Rank** | **Unique** | **Peptide** |
| --- | --- | --- | --- | --- | --- | --- | --- | --- | --- | --- | --- |
|  | [720](http://10.200.41.11/mascot/cgi/peptide_view.pl?file=..%2Fdata%2F20160609%2FF081285.dat&query=720&hit=1&index=gi%7C194402702%7Cgb%7CACF62924.1%7C&db_idx=1&px=1&section=5&ave_thresh=32&_ignoreionsscorebelow=0&report=0&_sigthreshold=0.05&_msresflags=1089&_msresflags2=2&percolate=-1&percolate_rt=0&_minpeplen=5&sessionID=all_secdisabledsession) | **490.7449** | **979.4752** | **979.4723** | **2.95** | **0** | **46** | **0.0032** | **1** | **U** | **R.TFIDNSQR.N** [718](http://10.200.41.11/mascot/cgi/peptide_view.pl?file=..%2Fdata%2F20160609%2FF081285.dat&query=718&hit=1&index=gi%7C194402702%7Cgb%7CACF62924.1%7C&db_idx=1&px=1&section=5&ave_thresh=32&_ignoreionsscorebelow=0&report=0&_sigthreshold=0.05&_msresflags=1089&_msresflags2=2&percolate=-1&percolate_rt=0&_minpeplen=5&sessionID=all_secdisabledsession) [719](http://10.200.41.11/mascot/cgi/peptide_view.pl?file=..%2Fdata%2F20160609%2FF081285.dat&query=719&hit=1&index=gi%7C194402702%7Cgb%7CACF62924.1%7C&db_idx=1&px=1&section=5&ave_thresh=32&_ignoreionsscorebelow=0&report=0&_sigthreshold=0.05&_msresflags=1089&_msresflags2=2&percolate=-1&percolate_rt=0&_minpeplen=5&sessionID=all_secdisabledsession) [721](http://10.200.41.11/mascot/cgi/peptide_view.pl?file=..%2Fdata%2F20160609%2FF081285.dat&query=721&hit=1&index=gi%7C194402702%7Cgb%7CACF62924.1%7C&db_idx=1&px=1&section=5&ave_thresh=32&_ignoreionsscorebelow=0&report=0&_sigthreshold=0.05&_msresflags=1089&_msresflags2=2&percolate=-1&percolate_rt=0&_minpeplen=5&sessionID=all_secdisabledsession) [722](http://10.200.41.11/mascot/cgi/peptide_view.pl?file=..%2Fdata%2F20160609%2FF081285.dat&query=722&hit=1&index=gi%7C194402702%7Cgb%7CACF62924.1%7C&db_idx=1&px=1&section=5&ave_thresh=32&_ignoreionsscorebelow=0&report=0&_sigthreshold=0.05&_msresflags=1089&_msresflags2=2&percolate=-1&percolate_rt=0&_minpeplen=5&sessionID=all_secdisabledsession) [723](http://10.200.41.11/mascot/cgi/peptide_view.pl?file=..%2Fdata%2F20160609%2FF081285.dat&query=723&hit=1&index=gi%7C194402702%7Cgb%7CACF62924.1%7C&db_idx=1&px=1&section=5&ave_thresh=32&_ignoreionsscorebelow=0&report=0&_sigthreshold=0.05&_msresflags=1089&_msresflags2=2&percolate=-1&percolate_rt=0&_minpeplen=5&sessionID=all_secdisabledsession) [724](http://10.200.41.11/mascot/cgi/peptide_view.pl?file=..%2Fdata%2F20160609%2FF081285.dat&query=724&hit=1&index=gi%7C194402702%7Cgb%7CACF62924.1%7C&db_idx=1&px=1&section=5&ave_thresh=32&_ignoreionsscorebelow=0&report=0&_sigthreshold=0.05&_msresflags=1089&_msresflags2=2&percolate=-1&percolate_rt=0&_minpeplen=5&sessionID=all_secdisabledsession) |
|  | [2148](http://10.200.41.11/mascot/cgi/peptide_view.pl?file=..%2Fdata%2F20160609%2FF081285.dat&query=2148&hit=2&index=gi%7C194402702%7Cgb%7CACF62924.1%7C&db_idx=1&px=1&section=5&ave_thresh=32&_ignoreionsscorebelow=0&report=0&_sigthreshold=0.05&_msresflags=1089&_msresflags2=2&percolate=-1&percolate_rt=0&_minpeplen=5&sessionID=all_secdisabledsession) | **462.5805** | **1384.7196** | **1384.7133** | **4.55** | **0** | **2** | **1e+002** | **2** | **U** | **R.VITTVDGLHMQR.-** |
|  | [2545](http://10.200.41.11/mascot/cgi/peptide_view.pl?file=..%2Fdata%2F20160609%2FF081285.dat&query=2545&hit=1&index=gi%7C194402702%7Cgb%7CACF62924.1%7C&db_idx=1&px=1&section=5&ave_thresh=32&_ignoreionsscorebelow=0&report=0&_sigthreshold=0.05&_msresflags=1089&_msresflags2=2&percolate=-1&percolate_rt=0&_minpeplen=5&sessionID=all_secdisabledsession) | **825.3999** | **2473.1779** | **2473.1708** | **2.85** | **1** | **58** | **0.00021** | **1** | **U** | **K.VVTSEKGTTGETTSFDEVDGVTSK.S** [2543](http://10.200.41.11/mascot/cgi/peptide_view.pl?file=..%2Fdata%2F20160609%2FF081285.dat&query=2543&hit=1&index=gi%7C194402702%7Cgb%7CACF62924.1%7C&db_idx=1&px=1&section=5&ave_thresh=32&_ignoreionsscorebelow=0&report=0&_sigthreshold=0.05&_msresflags=1089&_msresflags2=2&percolate=-1&percolate_rt=0&_minpeplen=5&sessionID=all_secdisabledsession) [2544](http://10.200.41.11/mascot/cgi/peptide_view.pl?file=..%2Fdata%2F20160609%2FF081285.dat&query=2544&hit=1&index=gi%7C194402702%7Cgb%7CACF62924.1%7C&db_idx=1&px=1&section=5&ave_thresh=32&_ignoreionsscorebelow=0&report=0&_sigthreshold=0.05&_msresflags=1089&_msresflags2=2&percolate=-1&percolate_rt=0&_minpeplen=5&sessionID=all_secdisabledsession) [2546](http://10.200.41.11/mascot/cgi/peptide_view.pl?file=..%2Fdata%2F20160609%2FF081285.dat&query=2546&hit=1&index=gi%7C194402702%7Cgb%7CACF62924.1%7C&db_idx=1&px=1&section=5&ave_thresh=32&_ignoreionsscorebelow=0&report=0&_sigthreshold=0.05&_msresflags=1089&_msresflags2=2&percolate=-1&percolate_rt=0&_minpeplen=5&sessionID=all_secdisabledsession) |
|  | [2549](http://10.200.41.11/mascot/cgi/peptide_view.pl?file=..%2Fdata%2F20160609%2FF081285.dat&query=2549&hit=1&index=gi%7C194402702%7Cgb%7CACF62924.1%7C&db_idx=1&px=1&section=5&ave_thresh=32&_ignoreionsscorebelow=0&report=0&_sigthreshold=0.05&_msresflags=1089&_msresflags2=2&percolate=-1&percolate_rt=0&_minpeplen=5&sessionID=all_secdisabledsession) | **648.8158** | **2591.2341** | **2591.2212** | **4.97** | **0** | **31** | **0.14** | **1** | **U** | **R.VDSTTHQTETAHSASTGAIDHGIAGK.I** [2547](http://10.200.41.11/mascot/cgi/peptide_view.pl?file=..%2Fdata%2F20160609%2FF081285.dat&query=2547&hit=1&index=gi%7C194402702%7Cgb%7CACF62924.1%7C&db_idx=1&px=1&section=5&ave_thresh=32&_ignoreionsscorebelow=0&report=0&_sigthreshold=0.05&_msresflags=1089&_msresflags2=2&percolate=-1&percolate_rt=0&_minpeplen=5&sessionID=all_secdisabledsession) [2548](http://10.200.41.11/mascot/cgi/peptide_view.pl?file=..%2Fdata%2F20160609%2FF081285.dat&query=2548&hit=1&index=gi%7C194402702%7Cgb%7CACF62924.1%7C&db_idx=1&px=1&section=5&ave_thresh=32&_ignoreionsscorebelow=0&report=0&_sigthreshold=0.05&_msresflags=1089&_msresflags2=2&percolate=-1&percolate_rt=0&_minpeplen=5&sessionID=all_secdisabledsession) |

### Protein sequence coverage: 10%

Matched peptides shown in ***bold red***.

| **1** | MQTEIKTQAT | NLAANLSAVR | ESATTTLSGE | IKGPQLEDFP | ALIKQASLDA |
| --- | --- | --- | --- | --- | --- |
| **51** | LFKCGKDAEA | LKEVFTNSNN | VAGKKAIMEF | AGLFRSALNA | TSDSPEAKTL |
| **101** | LMKVGAEYTA | QIIKDGLKEK | SAFGPWLPET | KKAEAKLENL | EKQLLDIIKN |
| **151** | NTGGELSKLS | TNLVMQEVMP | YIASCIEHNF | GCTLDPLTRS | NLTHLVDKAA |
| **201** | AKAVEALDMC | HQKLTQEQGT | SVGREARHLE | MQTLIPLLLR | NVFAQIPADK |
| **251** | LPDPKIPEPA | AGPVPDGGKK | AEPTGININI | NIDSSNHSVD | NSKHINNSRS |
| **301** | HVDNSQRHID | NSNHDNSRKT | IDNSR**TFIDN** | **SQR**NGESHHS | TNSSNVSHSH |
| **351** | SR**VDSTTHQT** | **ETAHSASTGA** | **IDHGIAGK**ID | VTAHATAEAV | TNASSESKDG |
| **401** | K**VVTSEKGTT** | **GETTSFDEVD** | **GVTSK**SIIGK | PVQATVHGVD | DNKQQSQTAE |
| **451** | IVNVKPLASQ | LAGVENVKTD | TLQSDTTVIT | GNKAGTTDND | NSQTDKTGPF |
| **501** | SGLKFKQNSF | LSTVPSVTNM | HSMHFDARET | FLGVIRKALE | PDTSTPFPVR |
| **551** | RAFDGLRAEI | LPNDTIKSAA | LKAQCSDIDK | HPELKAKMET | LKEVITHHPQ |
| **601** | KEKLAEIALQ | FAREAGLTRL | KGETDYVLSN | VLDGLIGDGS | WRAGPAYESY |
| **651** | LNKPGVDR**VI** | **TTVDGLHMQR** |  |  |  |

| **6.** | [gi|392616944|gb|EIW99372.1|](http://10.200.41.11/mascot/cgi/protein_view.pl?file=..%2Fdata%2F20160609%2FF081285.dat&hit=gi%7C392616944%7Cgb%7CEIW99372.1%7C&db_idx=1&px=1&ave_thresh=32&_ignoreionsscorebelow=0&report=0&_sigthreshold=0.05&_msresflags=1089&_msresflags2=2&percolate=-1&percolate_rt=0&_minpeplen=5&sessionID=all_secdisabledsession)    **Mass:** 37081    **Score:** 236    **Matches:** 4(4)  **Sequences:** 1(1)  **emPAI:** 0.15 |
| --- | --- |
|  | cell invasion protein SipD [Salmonella enterica subsp. enterica serovar Newport str. Levine 15] |

|  | **Query** | **Observed** | **Mr(expt)** | **Mr(calc)** | **ppm** | **Miss** | **Score** | **Expect** | **Rank** | **Unique** | **Peptide** |
| --- | --- | --- | --- | --- | --- | --- | --- | --- | --- | --- | --- |
|  | [2348](http://10.200.41.11/mascot/cgi/peptide_view.pl?file=..%2Fdata%2F20160609%2FF081285.dat&query=2348&hit=1&index=gi%7C392616944%7Cgb%7CEIW99372.1%7C&db_idx=1&px=1&section=5&ave_thresh=32&_ignoreionsscorebelow=0&report=0&_sigthreshold=0.05&_msresflags=1089&_msresflags2=2&percolate=-1&percolate_rt=0&_minpeplen=5&sessionID=all_secdisabledsession) | **752.3762** | **1502.7379** | **1502.7325** | **3.61** | **0** | **85** | **4.6e-007** | **1** | **U** | **K.SGVSLSAEQNENLR.S** [2345](http://10.200.41.11/mascot/cgi/peptide_view.pl?file=..%2Fdata%2F20160609%2FF081285.dat&query=2345&hit=1&index=gi%7C392616944%7Cgb%7CEIW99372.1%7C&db_idx=1&px=1&section=5&ave_thresh=32&_ignoreionsscorebelow=0&report=0&_sigthreshold=0.05&_msresflags=1089&_msresflags2=2&percolate=-1&percolate_rt=0&_minpeplen=5&sessionID=all_secdisabledsession) [2346](http://10.200.41.11/mascot/cgi/peptide_view.pl?file=..%2Fdata%2F20160609%2FF081285.dat&query=2346&hit=1&index=gi%7C392616944%7Cgb%7CEIW99372.1%7C&db_idx=1&px=1&section=5&ave_thresh=32&_ignoreionsscorebelow=0&report=0&_sigthreshold=0.05&_msresflags=1089&_msresflags2=2&percolate=-1&percolate_rt=0&_minpeplen=5&sessionID=all_secdisabledsession) [2347](http://10.200.41.11/mascot/cgi/peptide_view.pl?file=..%2Fdata%2F20160609%2FF081285.dat&query=2347&hit=1&index=gi%7C392616944%7Cgb%7CEIW99372.1%7C&db_idx=1&px=1&section=5&ave_thresh=32&_ignoreionsscorebelow=0&report=0&_sigthreshold=0.05&_msresflags=1089&_msresflags2=2&percolate=-1&percolate_rt=0&_minpeplen=5&sessionID=all_secdisabledsession) |

**Protein sequence coverage: 4%**

Matched peptides shown in ***bold red***.

| **1** | MLNIQNYSAS | PHPGIVAERP | QTPSASEHVE | TAVVPSTTEH | RGTDIISLSQ |
| --- | --- | --- | --- | --- | --- |
| **51** | AATKIQQAQQ | TLQSTPPISE | ENNDERTLAR | QQLTSSLNAL | AK**SGVSLSAE** |
| **101** | **QNENLR**SAFS | APTSALFSAS | PMAQPRTTIS | DAEIWDMVSQ | NISAIGDSYL |
| **151** | GVYENVVAVY | TDFYQAFSDI | LSKMGGWLLP | GKDGNTVKLD | VTSLKNDLNS |
| **201** | LVNKYNQINS | NTVLFPAQSG | SGVKVATEAE | ARQWLSELNL | PNSCLKSYGS |
| **251** | GYVVTVDLTP | LQKMVQDIDG | LGAPGKDSKL | EMDNAKYQAW | QSGFKAQEEN |
| **301** | MKTTLQTLTQ | KYSNANSLYD | NLVKVLSSTI | SSSLETAKSF | LQG |

| **7.** | [gi|194403640|gb|ACF63862.1|](http://10.200.41.11/mascot/cgi/protein_view.pl?file=..%2Fdata%2F20160609%2FF081285.dat&hit=gi%7C194403640%7Cgb%7CACF63862.1%7C&db_idx=1&px=1&ave_thresh=32&_ignoreionsscorebelow=0&report=0&_sigthreshold=0.05&_msresflags=1089&_msresflags2=2&percolate=-1&percolate_rt=0&_minpeplen=5&sessionID=all_secdisabledsession)    **Mass:** 62382    **Score:** 105    **Matches:** 6(2)  **Sequences:** 2(1)  **emPAI:** 0.09 |
| --- | --- |
|  | cell invasion protein SipB [Salmonella enterica subsp. enterica serovar Newport str. SL254] |

|  | **Query** | **Observed** | **Mr(expt)** | **Mr(calc)** | **ppm** | **Miss** | **Score** | **Expect** | **Rank** | **Unique** | **Peptide** |
| --- | --- | --- | --- | --- | --- | --- | --- | --- | --- | --- | --- |
|  | [718](http://10.200.41.11/mascot/cgi/peptide_view.pl?file=..%2Fdata%2F20160609%2FF081285.dat&query=718&hit=5&index=gi%7C194403640%7Cgb%7CACF63862.1%7C&db_idx=1&px=1&section=5&ave_thresh=32&_ignoreionsscorebelow=0&report=0&_sigthreshold=0.05&_msresflags=1089&_msresflags2=2&percolate=-1&percolate_rt=0&_minpeplen=5&sessionID=all_secdisabledsession) | 490.7443 | 979.4740 | 979.4909 | -17.32 | 0 | 1 | 96 | 5 | U | K.LFTQGMQR.I [719](http://10.200.41.11/mascot/cgi/peptide_view.pl?file=..%2Fdata%2F20160609%2FF081285.dat&query=719&hit=5&index=gi%7C194403640%7Cgb%7CACF63862.1%7C&db_idx=1&px=1&section=5&ave_thresh=32&_ignoreionsscorebelow=0&report=0&_sigthreshold=0.05&_msresflags=1089&_msresflags2=2&percolate=-1&percolate_rt=0&_minpeplen=5&sessionID=all_secdisabledsession) [720](http://10.200.41.11/mascot/cgi/peptide_view.pl?file=..%2Fdata%2F20160609%2FF081285.dat&query=720&hit=7&index=gi%7C194403640%7Cgb%7CACF63862.1%7C&db_idx=1&px=1&section=5&ave_thresh=32&_ignoreionsscorebelow=0&report=0&_sigthreshold=0.05&_msresflags=1089&_msresflags2=2&percolate=-1&percolate_rt=0&_minpeplen=5&sessionID=all_secdisabledsession) [722](http://10.200.41.11/mascot/cgi/peptide_view.pl?file=..%2Fdata%2F20160609%2FF081285.dat&query=722&hit=4&index=gi%7C194403640%7Cgb%7CACF63862.1%7C&db_idx=1&px=1&section=5&ave_thresh=32&_ignoreionsscorebelow=0&report=0&_sigthreshold=0.05&_msresflags=1089&_msresflags2=2&percolate=-1&percolate_rt=0&_minpeplen=5&sessionID=all_secdisabledsession) |
|  | [1060](http://10.200.41.11/mascot/cgi/peptide_view.pl?file=..%2Fdata%2F20160609%2FF081285.dat&query=1060&hit=1&index=gi%7C194403640%7Cgb%7CACF63862.1%7C&db_idx=1&px=1&section=5&ave_thresh=32&_ignoreionsscorebelow=0&report=0&_sigthreshold=0.05&_msresflags=1089&_msresflags2=2&percolate=-1&percolate_rt=0&_minpeplen=5&sessionID=all_secdisabledsession) | **531.7845** | **1061.5544** | **1061.5505** | **3.66** | **0** | **71** | **1.5e-005** | **1** | **U** | **R.LAEAAFEGVR.K** [1059](http://10.200.41.11/mascot/cgi/peptide_view.pl?file=..%2Fdata%2F20160609%2FF081285.dat&query=1059&hit=1&index=gi%7C194403640%7Cgb%7CACF63862.1%7C&db_idx=1&px=1&section=5&ave_thresh=32&_ignoreionsscorebelow=0&report=0&_sigthreshold=0.05&_msresflags=1089&_msresflags2=2&percolate=-1&percolate_rt=0&_minpeplen=5&sessionID=all_secdisabledsession) |

**Protein sequence coverage: 3%**

Matched peptides shown in ***bold red***.

| **1** | MVNDASSISR | SGYTQNPR**LA** | **EAAFEGVR**KN | TDFLKAADKA | FKDVVATKAG |
| --- | --- | --- | --- | --- | --- |
| **51** | DLKAGTKSGE | SAINTVGLKP | PTDAAREKLS | SEGQLTLLLG | KLMTLLGDVS |
| **101** | LSQLESRLAV | WQAMIESQKE | MGIQVSKEFQ | TALGEAQEAT | DLYEASIKKT |
| **151** | DTAKSVYDAA | AKKLTQAQNK | LQSLDPADPG | YAQAEAAVEQ | AGKEATEAKE |
| **201** | ALDKATDATV | KAGTDAKAKA | EKADNILTKF | QGTANAASQN | QVSQGEQDNL |
| **251** | SNVARLTMLM | AMFIEIVGKN | TEESLQNDLA | LFNALQEGRQ | AEMEKKSAEF |
| **301** | QEETRKAEET | NRIMGCIGKV | LGALLTIVSV | VAAVFTGGAS | LALAAVGLAV |
| **351** | MVADEIVKAA | TGVSFIQQAL | NPIMEHVLKP | LMELIGKAIT | KALEGLGVDK |
| **401** | KTAEMAGSIV | GAIVAAIAMV | AVIVVVAVVG | KGAAAKLGNA | LSKMMGETIK |
| **451** | KLVPNVLKQL | AQNGSK**LFTQ** | **GMQR**ITSGLG | NVGSKMGLQT | NALSKELVGN |
| **501** | TLNKVALGME | VTNTAAQSAG | GVAEGVFIKN | ASEALADFML | ARFAMDQIQQ |
| **551** | WLKQSVEIFG | ENQKVTAELQ | KAMSSAVQQN | ADASRFILRQ | SRA |

****************************************************15 minute SS Control*****************************************************

**1^st^ technical replicate**

**Enzyme : Trypsin**

**Variable modifications :** [**Oxidation (M)**](http://10.200.41.11/mascot/cgi/client.pl?modification&mod_name=Oxidation%20%28M%29&file=..%2Fdata%2F20160609%2FF081286.dat)

**Mass values : Monoisotopic**

**Protein Mass : Unrestricted**

**Peptide Mass Tolerance : ± 20 ppm**

**Fragment Mass Tolerance: ± 0.4 Da**

**Max Missed Cleavages : 3**

**Instrument type : Default**

**Number of queries : 2744**

| **Protein hits           :** | [**gi|194405105|gb|ACF65327.1|**](http://10.200.41.11/mascot/cgi/master_results.pl?file=..%2Fdata%2F20160609%2FF081286.dat#Hit1) | (Di)nucleoside polyphosphate hydrolase [Salmonella enterica subsp. enterica serovar Newport str. SL254] |
| --- | --- | --- |
|  | [**gi|195630380|gb|EDX49006.1|**](http://10.200.41.11/mascot/cgi/master_results.pl?file=..%2Fdata%2F20160609%2FF081286.dat#Hit2) | protein RecT [Salmonella enterica subsp. enterica serovar Newport str. SL317] |
|  | [**gi|446662362|ref|WP_000739708.1|**](http://10.200.41.11/mascot/cgi/master_results.pl?file=..%2Fdata%2F20160609%2FF081286.dat#Hit3) | hypothetical protein [Salmonella enterica] |

*******************************************************************************************************************************************************

**2^nd^ technical replicate**

**Enzyme : Trypsin**

**Variable modifications :** [**Oxidation (M)**](http://10.200.41.11/mascot/cgi/client.pl?modification&mod_name=Oxidation%20%28M%29&file=..%2Fdata%2F20160609%2FF081287.dat)

**Mass values : Monoisotopic**

**Protein Mass : Unrestricted**

**Peptide Mass Tolerance : ± 20 ppm**

**Fragment Mass Tolerance: ± 0.4 Da**

**Max Missed Cleavages : 3**

**Instrument type : Default**

**Number of queries : 2588**

| **Protein hits           :** | [**gi|446662362|ref|WP_000739708.1|**](http://10.200.41.11/mascot/cgi/master_results.pl?file=..%2Fdata%2F20160609%2FF081287.dat#Hit1) | hypothetical protein [Salmonella enterica] |
| --- | --- | --- |
|  | [**gi|195630380|gb|EDX49006.1|**](http://10.200.41.11/mascot/cgi/master_results.pl?file=..%2Fdata%2F20160609%2FF081287.dat#Hit2) | protein RecT [Salmonella enterica subsp. enterica serovar Newport str. SL317] |
|  |  |  |
|  |  |  |

*******************************************************************************************************************************************************

**3^rd^ technical replicate**

**Enzyme : Trypsin**

**Variable modifications :** [**Oxidation (M)**](http://10.200.41.11/mascot/cgi/client.pl?modification&mod_name=Oxidation%20%28M%29&file=..%2Fdata%2F20160609%2FF081288.dat)

**Mass values : Monoisotopic**

**Protein Mass : Unrestricted**

**Peptide Mass Tolerance : ± 20 ppm**

**Fragment Mass Tolerance: ± 0.4 Da**

**Max Missed Cleavages : 3**

**Instrument type : Default**

**Number of queries : 2372**

| **Protein hits           :** | [**gi|195630380|gb|EDX49006.1|**](http://10.200.41.11/mascot/cgi/master_results.pl?file=..%2Fdata%2F20160609%2FF081288.dat#Hit1) | protein RecT [Salmonella enterica subsp. enterica serovar Newport str. SL317] |
| --- | --- | --- |

*********************************************************20 Hour Re-Digest*****************************************************

**1^st^ technical replicate**

**Enzyme : Trypsin**

**Variable modifications :** [**Oxidation (M)**](http://10.200.41.11/mascot/cgi/client.pl?modification&mod_name=Oxidation%20%28M%29&file=..%2Fdata%2F20160609%2FF081289.dat)

**Mass values : Monoisotopic**

**Protein Mass : Unrestricted**

**Peptide Mass Tolerance : ± 20 ppm**

**Fragment Mass Tolerance: ± 0.4 Da**

**Max Missed Cleavages : 3**

**Instrument type : Default**

**Number of queries : 2952**

| Protein hits           : | [**gi|194402702|gb|ACF62924.1|**](http://10.200.41.11/mascot/cgi/master_results.pl?file=..%2Fdata%2F20160609%2FF081289.dat#Hit1) | cell invasion protein SipA [Salmonella enterica subsp. enterica serovar Newport str. SL254] |
| --- | --- | --- |
|  | [**gi|50830890|gb|AAT81610.1|**](http://10.200.41.11/mascot/cgi/master_results.pl?file=..%2Fdata%2F20160609%2FF081289.dat#Hit2) | phase 1 flagellin [Salmonella enterica subsp. enterica serovar Newport] |
|  | [**gi|392616945|gb|EIW99373.1|**](http://10.200.41.11/mascot/cgi/master_results.pl?file=..%2Fdata%2F20160609%2FF081289.dat#Hit3) | pathogenicity island 1 effector protein SipC [Salmonella enterica subsp. enterica serovar Newport str. Levine 15] |
|  | [**gi|194401698|gb|ACF61920.1|**](http://10.200.41.11/mascot/cgi/master_results.pl?file=..%2Fdata%2F20160609%2FF081289.dat#Hit4) | translation elongation factor Tu [Salmonella enterica subsp. enterica serovar Newport str. SL254] |
|  | [**gi|194403331|gb|ACF63553.1|**](http://10.200.41.11/mascot/cgi/master_results.pl?file=..%2Fdata%2F20160609%2FF081289.dat#Hit5) | negative regulator of flagellin synthesis [Salmonella enterica subsp. enterica serovar Newport str. SL254] |
|  | [**gi|392616944|gb|EIW99372.1|**](http://10.200.41.11/mascot/cgi/master_results.pl?file=..%2Fdata%2F20160609%2FF081289.dat#Hit6) | cell invasion protein SipD [Salmonella enterica subsp. enterica serovar Newport str. Levine 15] |
|  | [**gi|194401103|gb|ACF61325.1|**](http://10.200.41.11/mascot/cgi/master_results.pl?file=..%2Fdata%2F20160609%2FF081289.dat#Hit7) | ribosomal protein L6 [Salmonella enterica subsp. enterica serovar Newport str. SL254] |
|  | [**gi|194401173|gb|ACF61395.1|**](http://10.200.41.11/mascot/cgi/master_results.pl?file=..%2Fdata%2F20160609%2FF081289.dat#Hit8) | flagellar hook-associated protein 2 [Salmonella enterica subsp. enterica serovar Newport str. SL254] |
|  | [**gi|194401878|gb|ACF62100.1|**](http://10.200.41.11/mascot/cgi/master_results.pl?file=..%2Fdata%2F20160609%2FF081289.dat#Hit9) | ribosomal protein L11 [Salmonella enterica subsp. enterica serovar Newport str. SL254] |
|  | [**gi|194401176|gb|ACF61398.1|**](http://10.200.41.11/mascot/cgi/master_results.pl?file=..%2Fdata%2F20160609%2FF081289.dat#Hit10) | ribosomal protein L7/L12 [Salmonella enterica subsp. enterica serovar Newport str. SL254] |
|  | [**gi|194401732|gb|ACF61954.1|**](http://10.200.41.11/mascot/cgi/master_results.pl?file=..%2Fdata%2F20160609%2FF081289.dat#Hit11) | chaperonin GroS [Salmonella enterica subsp. enterica serovar Newport str. SL254] |
|  | [**gi|194402721|gb|ACF62943.1|**](http://10.200.41.11/mascot/cgi/master_results.pl?file=..%2Fdata%2F20160609%2FF081289.dat#Hit12) | autonomous glycyl radical cofactor [Salmonella enterica subsp. enterica serovar Newport str. SL254] |
|  | [**gi|692117421|ref|WP_032074328.1|**](http://10.200.41.11/mascot/cgi/master_results.pl?file=..%2Fdata%2F20160609%2FF081289.dat#Hit13) | hypothetical protein [Salmonella enterica] |
|  | [**gi|194402309|gb|ACF62531.1|**](http://10.200.41.11/mascot/cgi/master_results.pl?file=..%2Fdata%2F20160609%2FF081289.dat#Hit14) | ribosomal protein S10 [Salmonella enterica subsp. enterica serovar Newport str. SL254] |
|  | [**gi|194403640|gb|ACF63862.1|**](http://10.200.41.11/mascot/cgi/master_results.pl?file=..%2Fdata%2F20160609%2FF081289.dat#Hit15) | cell invasion protein SipB [Salmonella enterica subsp. enterica serovar Newport str. SL254] |
|  | [**gi|194405415|gb|ACF65637.1|**](http://10.200.41.11/mascot/cgi/master_results.pl?file=..%2Fdata%2F20160609%2FF081289.dat#Hit16) | ribosomal protein L15 [Salmonella enterica subsp. enterica serovar Newport str. SL254] |
|  | [**gi|194404219|gb|ACF64441.1|**](http://10.200.41.11/mascot/cgi/master_results.pl?file=..%2Fdata%2F20160609%2FF081289.dat#Hit17) | flagellar hook-associated protein 3 [Salmonella enterica subsp. enterica serovar Newport str. SL254] |
|  | [**gi|194405308|gb|ACF65530.1|**](http://10.200.41.11/mascot/cgi/master_results.pl?file=..%2Fdata%2F20160609%2FF081289.dat#Hit18) | DNA-binding protein HU-beta [Salmonella enterica subsp. enterica serovar Newport str. SL254] |
|  | [**gi|194404381|gb|ACF64603.1|**](http://10.200.41.11/mascot/cgi/master_results.pl?file=..%2Fdata%2F20160609%2FF081289.dat#Hit19) | DNA-binding protein HU-alpha [Salmonella enterica subsp. enterica serovar Newport str. SL254] |
|  | [**gi|446662362|ref|WP_000739708.1|**](http://10.200.41.11/mascot/cgi/master_results.pl?file=..%2Fdata%2F20160609%2FF081289.dat#Hit20) | hypothetical protein [Salmonella enterica] |
|  | [**gi|195630380|gb|EDX49006.1|**](http://10.200.41.11/mascot/cgi/master_results.pl?file=..%2Fdata%2F20160609%2FF081289.dat#Hit21) | protein RecT [Salmonella enterica subsp. enterica serovar Newport str. SL317] |

| 1. | [gi|194402702|gb|ACF62924.1|](http://10.200.41.11/mascot/cgi/protein_view.pl?file=..%2Fdata%2F20160609%2FF081289.dat&hit=gi%7C194402702%7Cgb%7CACF62924.1%7C&db_idx=1&px=1&ave_thresh=32&_ignoreionsscorebelow=0&report=0&_sigthreshold=0.05&_msresflags=1089&_msresflags2=2&percolate=-1&percolate_rt=0&_minpeplen=5&sessionID=all_secdisabledsession)    Mass: 72333    Score: 476    Matches: 15(14)  Sequences: 7(6)  emPAI: 0.57 |
| --- | --- |
|  | cell invasion protein SipA [Salmonella enterica subsp. enterica serovar Newport str. SL254] |

|  | Query | Observed | Mr(expt) | Mr(calc) | ppm | Miss | Score | Expect | Rank | Unique | Peptide |
| --- | --- | --- | --- | --- | --- | --- | --- | --- | --- | --- | --- |
|  | [711](http://10.200.41.11/mascot/cgi/peptide_view.pl?file=..%2Fdata%2F20160609%2FF081289.dat&query=711&hit=1&index=gi%7C194402702%7Cgb%7CACF62924.1%7C&db_idx=1&px=1&section=5&ave_thresh=32&_ignoreionsscorebelow=0&report=0&_sigthreshold=0.05&_msresflags=1089&_msresflags2=2&percolate=-1&percolate_rt=0&_minpeplen=5&sessionID=all_secdisabledsession) | 490.7455 | 979.4765 | 979.4723 | 4.26 | 0 | 50 | 0.0016 | 1 | U | R.TFIDNSQR.N [709](http://10.200.41.11/mascot/cgi/peptide_view.pl?file=..%2Fdata%2F20160609%2FF081289.dat&query=709&hit=1&index=gi%7C194402702%7Cgb%7CACF62924.1%7C&db_idx=1&px=1&section=5&ave_thresh=32&_ignoreionsscorebelow=0&report=0&_sigthreshold=0.05&_msresflags=1089&_msresflags2=2&percolate=-1&percolate_rt=0&_minpeplen=5&sessionID=all_secdisabledsession) [710](http://10.200.41.11/mascot/cgi/peptide_view.pl?file=..%2Fdata%2F20160609%2FF081289.dat&query=710&hit=1&index=gi%7C194402702%7Cgb%7CACF62924.1%7C&db_idx=1&px=1&section=5&ave_thresh=32&_ignoreionsscorebelow=0&report=0&_sigthreshold=0.05&_msresflags=1089&_msresflags2=2&percolate=-1&percolate_rt=0&_minpeplen=5&sessionID=all_secdisabledsession) |
|  | [874](http://10.200.41.11/mascot/cgi/peptide_view.pl?file=..%2Fdata%2F20160609%2FF081289.dat&query=874&hit=1&index=gi%7C194402702%7Cgb%7CACF62924.1%7C&db_idx=1&px=1&section=5&ave_thresh=32&_ignoreionsscorebelow=0&report=0&_sigthreshold=0.05&_msresflags=1089&_msresflags2=2&percolate=-1&percolate_rt=0&_minpeplen=5&sessionID=all_secdisabledsession) | 513.7845 | 1025.5545 | 1025.5506 | 3.89 | 0 | 40 | 0.0073 | 1 | U | R.SNLTHLVDK.A |
|  | [1473](http://10.200.41.11/mascot/cgi/peptide_view.pl?file=..%2Fdata%2F20160609%2FF081289.dat&query=1473&hit=1&index=gi%7C194402702%7Cgb%7CACF62924.1%7C&db_idx=1&px=1&section=5&ave_thresh=32&_ignoreionsscorebelow=0&report=0&_sigthreshold=0.05&_msresflags=1089&_msresflags2=2&percolate=-1&percolate_rt=0&_minpeplen=5&sessionID=all_secdisabledsession) | 588.3065 | 1174.5984 | 1174.5942 | 3.53 | 0 | 77 | 3.8e-006 | 1 | U | K.LTQEQGTSVGR.E [1472](http://10.200.41.11/mascot/cgi/peptide_view.pl?file=..%2Fdata%2F20160609%2FF081289.dat&query=1472&hit=1&index=gi%7C194402702%7Cgb%7CACF62924.1%7C&db_idx=1&px=1&section=5&ave_thresh=32&_ignoreionsscorebelow=0&report=0&_sigthreshold=0.05&_msresflags=1089&_msresflags2=2&percolate=-1&percolate_rt=0&_minpeplen=5&sessionID=all_secdisabledsession) [1474](http://10.200.41.11/mascot/cgi/peptide_view.pl?file=..%2Fdata%2F20160609%2FF081289.dat&query=1474&hit=1&index=gi%7C194402702%7Cgb%7CACF62924.1%7C&db_idx=1&px=1&section=5&ave_thresh=32&_ignoreionsscorebelow=0&report=0&_sigthreshold=0.05&_msresflags=1089&_msresflags2=2&percolate=-1&percolate_rt=0&_minpeplen=5&sessionID=all_secdisabledsession) |
|  | [1814](http://10.200.41.11/mascot/cgi/peptide_view.pl?file=..%2Fdata%2F20160609%2FF081289.dat&query=1814&hit=1&index=gi%7C194402702%7Cgb%7CACF62924.1%7C&db_idx=1&px=1&section=5&ave_thresh=32&_ignoreionsscorebelow=0&report=0&_sigthreshold=0.05&_msresflags=1089&_msresflags2=2&percolate=-1&percolate_rt=0&_minpeplen=5&sessionID=all_secdisabledsession) | 645.8146 | 1289.6146 | 1289.6099 | 3.65 | 0 | 54 | 0.00035 | 1 | U | R.SALNATSDSPEAK.T [1813](http://10.200.41.11/mascot/cgi/peptide_view.pl?file=..%2Fdata%2F20160609%2FF081289.dat&query=1813&hit=1&index=gi%7C194402702%7Cgb%7CACF62924.1%7C&db_idx=1&px=1&section=5&ave_thresh=32&_ignoreionsscorebelow=0&report=0&_sigthreshold=0.05&_msresflags=1089&_msresflags2=2&percolate=-1&percolate_rt=0&_minpeplen=5&sessionID=all_secdisabledsession) |
|  | [2746](http://10.200.41.11/mascot/cgi/peptide_view.pl?file=..%2Fdata%2F20160609%2FF081289.dat&query=2746&hit=1&index=gi%7C194402702%7Cgb%7CACF62924.1%7C&db_idx=1&px=1&section=5&ave_thresh=32&_ignoreionsscorebelow=0&report=0&_sigthreshold=0.05&_msresflags=1089&_msresflags2=2&percolate=-1&percolate_rt=0&_minpeplen=5&sessionID=all_secdisabledsession) | 915.9194 | 1829.8242 | 1829.8167 | 4.08 | 0 | 91 | 5.5e-008 | 1 | U | K.GTTGETTSFDEVDGVTSK.S [2745](http://10.200.41.11/mascot/cgi/peptide_view.pl?file=..%2Fdata%2F20160609%2FF081289.dat&query=2745&hit=1&index=gi%7C194402702%7Cgb%7CACF62924.1%7C&db_idx=1&px=1&section=5&ave_thresh=32&_ignoreionsscorebelow=0&report=0&_sigthreshold=0.05&_msresflags=1089&_msresflags2=2&percolate=-1&percolate_rt=0&_minpeplen=5&sessionID=all_secdisabledsession) |
|  | [2940](http://10.200.41.11/mascot/cgi/peptide_view.pl?file=..%2Fdata%2F20160609%2FF081289.dat&query=2940&hit=1&index=gi%7C194402702%7Cgb%7CACF62924.1%7C&db_idx=1&px=1&section=5&ave_thresh=32&_ignoreionsscorebelow=0&report=0&_sigthreshold=0.05&_msresflags=1089&_msresflags2=2&percolate=-1&percolate_rt=0&_minpeplen=5&sessionID=all_secdisabledsession) | 825.4005 | 2473.1797 | 2473.1708 | 3.59 | 1 | 51 | 0.00097 | 1 | U | K.VVTSEKGTTGETTSFDEVDGVTSK.S [2939](http://10.200.41.11/mascot/cgi/peptide_view.pl?file=..%2Fdata%2F20160609%2FF081289.dat&query=2939&hit=1&index=gi%7C194402702%7Cgb%7CACF62924.1%7C&db_idx=1&px=1&section=5&ave_thresh=32&_ignoreionsscorebelow=0&report=0&_sigthreshold=0.05&_msresflags=1089&_msresflags2=2&percolate=-1&percolate_rt=0&_minpeplen=5&sessionID=all_secdisabledsession) [2941](http://10.200.41.11/mascot/cgi/peptide_view.pl?file=..%2Fdata%2F20160609%2FF081289.dat&query=2941&hit=1&index=gi%7C194402702%7Cgb%7CACF62924.1%7C&db_idx=1&px=1&section=5&ave_thresh=32&_ignoreionsscorebelow=0&report=0&_sigthreshold=0.05&_msresflags=1089&_msresflags2=2&percolate=-1&percolate_rt=0&_minpeplen=5&sessionID=all_secdisabledsession) |
|  | [2948](http://10.200.41.11/mascot/cgi/peptide_view.pl?file=..%2Fdata%2F20160609%2FF081289.dat&query=2948&hit=1&index=gi%7C194402702%7Cgb%7CACF62924.1%7C&db_idx=1&px=1&section=5&ave_thresh=32&_ignoreionsscorebelow=0&report=0&_sigthreshold=0.05&_msresflags=1089&_msresflags2=2&percolate=-1&percolate_rt=0&_minpeplen=5&sessionID=all_secdisabledsession) | 648.8152 | 2591.2319 | 2591.2212 | 4.12 | 0 | 19 | 1.8 | 1 | U | R.VDSTTHQTETAHSASTGAIDHGIAGK.I |

### Protein sequence coverage: 13%

Matched peptides shown in ***bold red***.

| **1** | MQTEIKTQAT | NLAANLSAVR | ESATTTLSGE | IKGPQLEDFP | ALIKQASLDA |
| --- | --- | --- | --- | --- | --- |
| **51** | LFKCGKDAEA | LKEVFTNSNN | VAGKKAIMEF | AGLFR**SALNA** | **TSDSPEAK**TL |
| **101** | LMKVGAEYTA | QIIKDGLKEK | SAFGPWLPET | KKAEAKLENL | EKQLLDIIKN |
| **151** | NTGGELSKLS | TNLVMQEVMP | YIASCIEHNF | GCTLDPLTR**S** | **NLTHLVDK**AA |
| **201** | AKAVEALDMC | HQK**LTQEQGT** | **SVGR**EARHLE | MQTLIPLLLR | NVFAQIPADK |
| **251** | LPDPKIPEPA | AGPVPDGGKK | AEPTGININI | NIDSSNHSVD | NSKHINNSRS |
| **301** | HVDNSQRHID | NSNHDNSRKT | IDNSR**TFIDN** | **SQR**NGESHHS | TNSSNVSHSH |
| **351** | SR**VDSTTHQT** | **ETAHSASTGA** | **IDHGIAGK**ID | VTAHATAEAV | TNASSESKDG |
| **401** | K**VVTSEKGTT** | **GETTSFDEVD** | **GVTSK**SIIGK | PVQATVHGVD | DNKQQSQTAE |
| **451** | IVNVKPLASQ | LAGVENVKTD | TLQSDTTVIT | GNKAGTTDND | NSQTDKTGPF |
| **501** | SGLKFKQNSF | LSTVPSVTNM | HSMHFDARET | FLGVIRKALE | PDTSTPFPVR |
| **551** | RAFDGLRAEI | LPNDTIKSAA | LKAQCSDIDK | HPELKAKMET | LKEVITHHPQ |
| **601** | KEKLAEIALQ | FAREAGLTRL | KGETDYVLSN | VLDGLIGDGS | WRAGPAYESY |
| **651** | LNKPGVDRVI | TTVDGLHMQR |  |  |  |

| 2. | [gi|50830890|gb|AAT81610.1|](http://10.200.41.11/mascot/cgi/protein_view.pl?file=..%2Fdata%2F20160609%2FF081289.dat&hit=gi%7C50830890%7Cgb%7CAAT81610.1%7C&db_idx=1&px=1&ave_thresh=32&_ignoreionsscorebelow=0&report=0&_sigthreshold=0.05&_msresflags=1089&_msresflags2=2&percolate=-1&percolate_rt=0&_minpeplen=5&sessionID=all_secdisabledsession)    Mass: 52223    Score: 331    Matches: 11(9)  Sequences: 3(3)  emPAI: 0.37 |
| --- | --- |
|  | phase 1 flagellin [Salmonella enterica subsp. enterica serovar Newport] |

|  | Query | Observed | Mr(expt) | Mr(calc) | ppm | Miss | Score | Expect | Rank | Unique | Peptide |
| --- | --- | --- | --- | --- | --- | --- | --- | --- | --- | --- | --- |
|  | [1280](http://10.200.41.11/mascot/cgi/peptide_view.pl?file=..%2Fdata%2F20160609%2FF081289.dat&query=1280&hit=1&index=gi%7C50830890%7Cgb%7CAAT81610.1%7C&db_idx=1&px=1&section=5&ave_thresh=32&_ignoreionsscorebelow=0&report=0&_sigthreshold=0.05&_msresflags=1089&_msresflags2=2&percolate=-1&percolate_rt=0&_minpeplen=5&sessionID=all_secdisabledsession) | 566.8037 | 1131.5929 | 1131.5884 | 3.97 | 0 | 61 | 0.00016 | 1 | U | K.SQSALGTAIER.L [1278](http://10.200.41.11/mascot/cgi/peptide_view.pl?file=..%2Fdata%2F20160609%2FF081289.dat&query=1278&hit=1&index=gi%7C50830890%7Cgb%7CAAT81610.1%7C&db_idx=1&px=1&section=5&ave_thresh=32&_ignoreionsscorebelow=0&report=0&_sigthreshold=0.05&_msresflags=1089&_msresflags2=2&percolate=-1&percolate_rt=0&_minpeplen=5&sessionID=all_secdisabledsession) [1279](http://10.200.41.11/mascot/cgi/peptide_view.pl?file=..%2Fdata%2F20160609%2FF081289.dat&query=1279&hit=1&index=gi%7C50830890%7Cgb%7CAAT81610.1%7C&db_idx=1&px=1&section=5&ave_thresh=32&_ignoreionsscorebelow=0&report=0&_sigthreshold=0.05&_msresflags=1089&_msresflags2=2&percolate=-1&percolate_rt=0&_minpeplen=5&sessionID=all_secdisabledsession) |
|  | [2433](http://10.200.41.11/mascot/cgi/peptide_view.pl?file=..%2Fdata%2F20160609%2FF081289.dat&query=2433&hit=1&index=gi%7C50830890%7Cgb%7CAAT81610.1%7C&db_idx=1&px=1&section=5&ave_thresh=32&_ignoreionsscorebelow=0&report=0&_sigthreshold=0.05&_msresflags=1089&_msresflags2=2&percolate=-1&percolate_rt=0&_minpeplen=5&sessionID=all_secdisabledsession) | 807.9158 | 1613.8170 | 1613.8121 | 3.03 | 1 | 53 | 0.00092 | 1 | U | R.INSAKDDAAGQAIANR.F |
|  | [2436](http://10.200.41.11/mascot/cgi/peptide_view.pl?file=..%2Fdata%2F20160609%2FF081289.dat&query=2436&hit=1&index=gi%7C50830890%7Cgb%7CAAT81610.1%7C&db_idx=1&px=1&section=5&ave_thresh=32&_ignoreionsscorebelow=0&report=0&_sigthreshold=0.05&_msresflags=1089&_msresflags2=2&percolate=-1&percolate_rt=0&_minpeplen=5&sessionID=all_secdisabledsession) | 538.9469 | 1613.8189 | 1613.8121 | 4.19 | 1 | (43) | 0.0095 | 1 | U | R.INSAKDDAAGQAIANR.F [2434](http://10.200.41.11/mascot/cgi/peptide_view.pl?file=..%2Fdata%2F20160609%2FF081289.dat&query=2434&hit=1&index=gi%7C50830890%7Cgb%7CAAT81610.1%7C&db_idx=1&px=1&section=5&ave_thresh=32&_ignoreionsscorebelow=0&report=0&_sigthreshold=0.05&_msresflags=1089&_msresflags2=2&percolate=-1&percolate_rt=0&_minpeplen=5&sessionID=all_secdisabledsession) [2435](http://10.200.41.11/mascot/cgi/peptide_view.pl?file=..%2Fdata%2F20160609%2FF081289.dat&query=2435&hit=1&index=gi%7C50830890%7Cgb%7CAAT81610.1%7C&db_idx=1&px=1&section=5&ave_thresh=32&_ignoreionsscorebelow=0&report=0&_sigthreshold=0.05&_msresflags=1089&_msresflags2=2&percolate=-1&percolate_rt=0&_minpeplen=5&sessionID=all_secdisabledsession) |
|  | [2871](http://10.200.41.11/mascot/cgi/peptide_view.pl?file=..%2Fdata%2F20160609%2FF081289.dat&query=2871&hit=1&index=gi%7C50830890%7Cgb%7CAAT81610.1%7C&db_idx=1&px=1&section=5&ave_thresh=32&_ignoreionsscorebelow=0&report=0&_sigthreshold=0.05&_msresflags=1089&_msresflags2=2&percolate=-1&percolate_rt=0&_minpeplen=5&sessionID=all_secdisabledsession) | 659.2954 | 1974.8644 | 1974.8589 | 2.80 | 1 | 56 | 0.0001 | 1 | U | R.SRIEDSDYATEVSNMSR.A [2872](http://10.200.41.11/mascot/cgi/peptide_view.pl?file=..%2Fdata%2F20160609%2FF081289.dat&query=2872&hit=1&index=gi%7C50830890%7Cgb%7CAAT81610.1%7C&db_idx=1&px=1&section=5&ave_thresh=32&_ignoreionsscorebelow=0&report=0&_sigthreshold=0.05&_msresflags=1089&_msresflags2=2&percolate=-1&percolate_rt=0&_minpeplen=5&sessionID=all_secdisabledsession) [2873](http://10.200.41.11/mascot/cgi/peptide_view.pl?file=..%2Fdata%2F20160609%2FF081289.dat&query=2873&hit=1&index=gi%7C50830890%7Cgb%7CAAT81610.1%7C&db_idx=1&px=1&section=5&ave_thresh=32&_ignoreionsscorebelow=0&report=0&_sigthreshold=0.05&_msresflags=1089&_msresflags2=2&percolate=-1&percolate_rt=0&_minpeplen=5&sessionID=all_secdisabledsession) [2874](http://10.200.41.11/mascot/cgi/peptide_view.pl?file=..%2Fdata%2F20160609%2FF081289.dat&query=2874&hit=1&index=gi%7C50830890%7Cgb%7CAAT81610.1%7C&db_idx=1&px=1&section=5&ave_thresh=32&_ignoreionsscorebelow=0&report=0&_sigthreshold=0.05&_msresflags=1089&_msresflags2=2&percolate=-1&percolate_rt=0&_minpeplen=5&sessionID=all_secdisabledsession) |

### Protein sequence coverage: 8%

Matched peptides shown in ***bold red***.

| **1** | MAQVINTNSL | SLLTQNNLNK | **SQSALGTAIE** | **R**LSSGLR**INS** | **AKDDAAGQAI** |
| --- | --- | --- | --- | --- | --- |
| **51** | **ANR**FTANIKG | LTQASRNAND | GISIAQTTEG | ALNEINNNLQ | RVRELAVQSA |
| **101** | NSTNSQSDLD | SIQAEITQRL | NEIDRVSGQT | QFNGVKVLAQ | DNTLTIQVGA |
| **151** | NDGETIDIDL | KQINSQTLGL | DTLNVQKAYD | VSATAAMDPK | SFTDGTKNLT |
| **201** | APDATAIKAA | LGNPAATGDS | LSATLSFKDG | KYYATVAGYT | NAADTSKNGK |
| **251** | YEVNVDSATG | AVTFNAAPTK | ATVTGDTTVT | KVQVNAPVAV | STDVKKALED |
| **301** | GGVSNADATA | AKLVKMSYTD | KNGKSIDGGY | ALEAGGKYYA | ATYDEGTGKI |
| **351** | TANVTTYTDS | TGVTKTAANQ | LGGVDGKTEV | VTIDGKTYNA | SKAAGHDFKA |
| **401** | QPELAEAAAK | TTENPLAKID | AALAQVDALR | SDLGAVQNRF | NSAITNLGNT |
| **451** | VNNLSEAR**SR** | **IEDSDYATEV** | **SNMSR**AQILQ | QAGTSVLAQA | NQVPQNVLSL |
| **501** | LR |  |  |  |  |

| 3. | [gi|392616945|gb|EIW99373.1|](http://10.200.41.11/mascot/cgi/protein_view.pl?file=..%2Fdata%2F20160609%2FF081289.dat&hit=gi%7C392616945%7Cgb%7CEIW99373.1%7C&db_idx=1&px=1&ave_thresh=32&_ignoreionsscorebelow=0&report=0&_sigthreshold=0.05&_msresflags=1089&_msresflags2=2&percolate=-1&percolate_rt=0&_minpeplen=5&sessionID=all_secdisabledsession)    Mass: 42957    Score: 262    Matches: 10(8)  Sequences: 5(4)  emPAI: 0.66 |
| --- | --- |
|  | pathogenicity island 1 effector protein SipC [Salmonella enterica subsp. enterica serovar Newport str. Levine 15] |

|  | Query | Observed | Mr(expt) | Mr(calc) | ppm | Miss | Score | Expect | Rank | Unique | Peptide |
| --- | --- | --- | --- | --- | --- | --- | --- | --- | --- | --- | --- |
|  | [754](http://10.200.41.11/mascot/cgi/peptide_view.pl?file=..%2Fdata%2F20160609%2FF081289.dat&query=754&hit=1&index=gi%7C392616945%7Cgb%7CEIW99373.1%7C&db_idx=1&px=1&section=5&ave_thresh=32&_ignoreionsscorebelow=0&report=0&_sigthreshold=0.05&_msresflags=1089&_msresflags2=2&percolate=-1&percolate_rt=0&_minpeplen=5&sessionID=all_secdisabledsession) | 494.7710 | 987.5274 | 987.5237 | 3.76 | 0 | 56 | 0.0007 | 1 | U | K.LGAEGVDSLK.S [755](http://10.200.41.11/mascot/cgi/peptide_view.pl?file=..%2Fdata%2F20160609%2FF081289.dat&query=755&hit=1&index=gi%7C392616945%7Cgb%7CEIW99373.1%7C&db_idx=1&px=1&section=5&ave_thresh=32&_ignoreionsscorebelow=0&report=0&_sigthreshold=0.05&_msresflags=1089&_msresflags2=2&percolate=-1&percolate_rt=0&_minpeplen=5&sessionID=all_secdisabledsession) [756](http://10.200.41.11/mascot/cgi/peptide_view.pl?file=..%2Fdata%2F20160609%2FF081289.dat&query=756&hit=1&index=gi%7C392616945%7Cgb%7CEIW99373.1%7C&db_idx=1&px=1&section=5&ave_thresh=32&_ignoreionsscorebelow=0&report=0&_sigthreshold=0.05&_msresflags=1089&_msresflags2=2&percolate=-1&percolate_rt=0&_minpeplen=5&sessionID=all_secdisabledsession) |
|  | [947](http://10.200.41.11/mascot/cgi/peptide_view.pl?file=..%2Fdata%2F20160609%2FF081289.dat&query=947&hit=1&index=gi%7C392616945%7Cgb%7CEIW99373.1%7C&db_idx=1&px=1&section=5&ave_thresh=32&_ignoreionsscorebelow=0&report=0&_sigthreshold=0.05&_msresflags=1089&_msresflags2=2&percolate=-1&percolate_rt=0&_minpeplen=5&sessionID=all_secdisabledsession) | 519.2524 | 1036.4902 | 1036.4859 | 4.17 | 0 | 37 | 0.018 | 1 | U | K.TMESINQSK.A |
|  | [1896](http://10.200.41.11/mascot/cgi/peptide_view.pl?file=..%2Fdata%2F20160609%2FF081289.dat&query=1896&hit=1&index=gi%7C392616945%7Cgb%7CEIW99373.1%7C&db_idx=1&px=1&section=5&ave_thresh=32&_ignoreionsscorebelow=0&report=0&_sigthreshold=0.05&_msresflags=1089&_msresflags2=2&percolate=-1&percolate_rt=0&_minpeplen=5&sessionID=all_secdisabledsession) | 651.8257 | 1301.6368 | 1301.6324 | 3.42 | 0 | 75 | 4e-006 | 1 | U | R.SEQQISQVNNR.V [1894](http://10.200.41.11/mascot/cgi/peptide_view.pl?file=..%2Fdata%2F20160609%2FF081289.dat&query=1894&hit=1&index=gi%7C392616945%7Cgb%7CEIW99373.1%7C&db_idx=1&px=1&section=5&ave_thresh=32&_ignoreionsscorebelow=0&report=0&_sigthreshold=0.05&_msresflags=1089&_msresflags2=2&percolate=-1&percolate_rt=0&_minpeplen=5&sessionID=all_secdisabledsession) [1895](http://10.200.41.11/mascot/cgi/peptide_view.pl?file=..%2Fdata%2F20160609%2FF081289.dat&query=1895&hit=1&index=gi%7C392616945%7Cgb%7CEIW99373.1%7C&db_idx=1&px=1&section=5&ave_thresh=32&_ignoreionsscorebelow=0&report=0&_sigthreshold=0.05&_msresflags=1089&_msresflags2=2&percolate=-1&percolate_rt=0&_minpeplen=5&sessionID=all_secdisabledsession) |
|  | [1942](http://10.200.41.11/mascot/cgi/peptide_view.pl?file=..%2Fdata%2F20160609%2FF081289.dat&query=1942&hit=1&index=gi%7C392616945%7Cgb%7CEIW99373.1%7C&db_idx=1&px=1&section=5&ave_thresh=32&_ignoreionsscorebelow=0&report=0&_sigthreshold=0.05&_msresflags=1089&_msresflags2=2&percolate=-1&percolate_rt=0&_minpeplen=5&sessionID=all_secdisabledsession) | 668.3446 | 1334.6747 | 1334.6678 | 5.17 | 0 | 52 | 0.0013 | 1 | U | K.SNAGTSATESLGIK.D |
|  | [2742](http://10.200.41.11/mascot/cgi/peptide_view.pl?file=..%2Fdata%2F20160609%2FF081289.dat&query=2742&hit=1&index=gi%7C392616945%7Cgb%7CEIW99373.1%7C&db_idx=1&px=1&section=5&ave_thresh=32&_ignoreionsscorebelow=0&report=0&_sigthreshold=0.05&_msresflags=1089&_msresflags2=2&percolate=-1&percolate_rt=0&_minpeplen=5&sessionID=all_secdisabledsession) | 593.9642 | 1778.8709 | 1778.8646 | 3.52 | 1 | 10 | 18 | 1 | U | K.SNAGTSATESLGIKDSNK.Q [2741](http://10.200.41.11/mascot/cgi/peptide_view.pl?file=..%2Fdata%2F20160609%2FF081289.dat&query=2741&hit=1&index=gi%7C392616945%7Cgb%7CEIW99373.1%7C&db_idx=1&px=1&section=5&ave_thresh=32&_ignoreionsscorebelow=0&report=0&_sigthreshold=0.05&_msresflags=1089&_msresflags2=2&percolate=-1&percolate_rt=0&_minpeplen=5&sessionID=all_secdisabledsession) |

### Protein sequence coverage: 11%

Matched peptides shown in ***bold red***.

| **1** | MLISNVGINP | AAYLNNHSVE | NSSQTASQSV | SAKDILNSIG | ISSSKVSDLG |
| --- | --- | --- | --- | --- | --- |
| **51** | LSPTLSAPAP | GVLTQTPGTI | TSFLKASIQN | TDMNQDLNAL | ANNVTTKANE |
| **101** | VVQTQLREQQ | AEVGKFFDIS | GMSSSAVALL | AAANTLMLTL | NQADSKLSGK |
| **151** | LSLVSFDAAK | TTASSMMREG | MNALSGSISQ | SALQLGITGV | GAKLEYKGLQ |
| **201** | NERGALKHNA | AKIDKLTTES | HSIKNVLNGQ | NSVK**LGAEGV** | **DSLK**SLNMKK |
| **251** | TGTDATKNLN | DATLK**SNAGT** | **SATESLGIKD** | **SNK**QISPEHQ | AILSKRLESV |
| **301** | ESDIRLEQNT | MDMTRIDARK | MQMTGDLIMK | NSVTVGGIAG | ASGQYAATQE |
| **351** | R**SEQQISQVN** | **NR**VASTASDE | ARESSRKSTS | LIQEMLK**TME** | **SINQSK**ASAL |
| **401** | AAIAGNIRA |  |  |  |  |

| 5. | [gi|194403331|gb|ACF63553.1|](http://10.200.41.11/mascot/cgi/protein_view.pl?file=..%2Fdata%2F20160609%2FF081289.dat&hit=gi%7C194403331%7Cgb%7CACF63553.1%7C&db_idx=1&px=1&ave_thresh=32&_ignoreionsscorebelow=0&report=0&_sigthreshold=0.05&_msresflags=1089&_msresflags2=2&percolate=-1&percolate_rt=0&_minpeplen=5&sessionID=all_secdisabledsession)    Mass: 10561    Score: 145    Matches: 6(4)  Sequences: 3(2)  emPAI: 1.68 |
| --- | --- |
|  | negative regulator of flagellin synthesis [Salmonella enterica subsp. enterica serovar Newport str. SL254] |

|  | Query | Observed | Mr(expt) | Mr(calc) | ppm | Miss | Score | Expect | Rank | Unique | Peptide |
| --- | --- | --- | --- | --- | --- | --- | --- | --- | --- | --- | --- |
|  | [963](http://10.200.41.11/mascot/cgi/peptide_view.pl?file=..%2Fdata%2F20160609%2FF081289.dat&query=963&hit=1&index=gi%7C194403331%7Cgb%7CACF63553.1%7C&db_idx=1&px=1&section=5&ave_thresh=32&_ignoreionsscorebelow=0&report=0&_sigthreshold=0.05&_msresflags=1089&_msresflags2=2&percolate=-1&percolate_rt=0&_minpeplen=5&sessionID=all_secdisabledsession) | 527.2660 | 1052.5174 | 1052.5138 | 3.44 | 0 | 52 | 0.00078 | 1 | U | R.EAQSYLQSK.- [962](http://10.200.41.11/mascot/cgi/peptide_view.pl?file=..%2Fdata%2F20160609%2FF081289.dat&query=962&hit=1&index=gi%7C194403331%7Cgb%7CACF63553.1%7C&db_idx=1&px=1&section=5&ave_thresh=32&_ignoreionsscorebelow=0&report=0&_sigthreshold=0.05&_msresflags=1089&_msresflags2=2&percolate=-1&percolate_rt=0&_minpeplen=5&sessionID=all_secdisabledsession) [964](http://10.200.41.11/mascot/cgi/peptide_view.pl?file=..%2Fdata%2F20160609%2FF081289.dat&query=964&hit=1&index=gi%7C194403331%7Cgb%7CACF63553.1%7C&db_idx=1&px=1&section=5&ave_thresh=32&_ignoreionsscorebelow=0&report=0&_sigthreshold=0.05&_msresflags=1089&_msresflags2=2&percolate=-1&percolate_rt=0&_minpeplen=5&sessionID=all_secdisabledsession) |
|  | [2179](http://10.200.41.11/mascot/cgi/peptide_view.pl?file=..%2Fdata%2F20160609%2FF081289.dat&query=2179&hit=1&index=gi%7C194403331%7Cgb%7CACF63553.1%7C&db_idx=1&px=1&section=5&ave_thresh=32&_ignoreionsscorebelow=0&report=0&_sigthreshold=0.05&_msresflags=1089&_msresflags2=2&percolate=-1&percolate_rt=0&_minpeplen=5&sessionID=all_secdisabledsession) | 707.4097 | 1412.8049 | 1412.7987 | 4.37 | 0 | 28 | 0.2 | 1 | U | R.TSPLKPVSTVQTR.E |
|  | [2180](http://10.200.41.11/mascot/cgi/peptide_view.pl?file=..%2Fdata%2F20160609%2FF081289.dat&query=2180&hit=1&index=gi%7C194403331%7Cgb%7CACF63553.1%7C&db_idx=1&px=1&section=5&ave_thresh=32&_ignoreionsscorebelow=0&report=0&_sigthreshold=0.05&_msresflags=1089&_msresflags2=2&percolate=-1&percolate_rt=0&_minpeplen=5&sessionID=all_secdisabledsession) | 471.9425 | 1412.8058 | 1412.7987 | 4.98 | 0 | (11) | 9.2 | 1 | U | R.TSPLKPVSTVQTR.E |
|  | [2432](http://10.200.41.11/mascot/cgi/peptide_view.pl?file=..%2Fdata%2F20160609%2FF081289.dat&query=2432&hit=1&index=gi%7C194403331%7Cgb%7CACF63553.1%7C&db_idx=1&px=1&section=5&ave_thresh=32&_ignoreionsscorebelow=0&report=0&_sigthreshold=0.05&_msresflags=1089&_msresflags2=2&percolate=-1&percolate_rt=0&_minpeplen=5&sessionID=all_secdisabledsession) | 804.9111 | 1607.8076 | 1607.8002 | 4.58 | 0 | 64 | 6.5e-005 | 1 | U | K.TSAATSASVTLSDAQAK.L |

### Protein sequence coverage: 40%

Matched peptides shown in ***bold red***.

| **1** | MSIDR**TSPLK** | **PVSTVQTR**ET | SDTPVQKTRQ | EK**TSAATSAS** | **VTLSDAQAK**L |
| --- | --- | --- | --- | --- | --- |
| **51** | MQPGVSDINM | ERVEALKTAI | RNGELKMDTG | KIADSLIR**EA** | **QSYLQSK** |

| 6. | [gi|392616944|gb|EIW99372.1|](http://10.200.41.11/mascot/cgi/protein_view.pl?file=..%2Fdata%2F20160609%2FF081289.dat&hit=gi%7C392616944%7Cgb%7CEIW99372.1%7C&db_idx=1&px=1&ave_thresh=32&_ignoreionsscorebelow=0&report=0&_sigthreshold=0.05&_msresflags=1089&_msresflags2=2&percolate=-1&percolate_rt=0&_minpeplen=5&sessionID=all_secdisabledsession)    Mass: 37081    Score: 139    Matches: 2(2)  Sequences: 1(1)  emPAI: 0.16 |
| --- | --- |
|  | cell invasion protein SipD [Salmonella enterica subsp. enterica serovar Newport str. Levine 15] |

|  | Query | Observed | Mr(expt) | Mr(calc) | ppm | Miss | Score | Expect | Rank | Unique | Peptide |
| --- | --- | --- | --- | --- | --- | --- | --- | --- | --- | --- | --- |
|  | [2295](http://10.200.41.11/mascot/cgi/peptide_view.pl?file=..%2Fdata%2F20160609%2FF081289.dat&query=2295&hit=1&index=gi%7C392616944%7Cgb%7CEIW99372.1%7C&db_idx=1&px=1&section=5&ave_thresh=32&_ignoreionsscorebelow=0&report=0&_sigthreshold=0.05&_msresflags=1089&_msresflags2=2&percolate=-1&percolate_rt=0&_minpeplen=5&sessionID=all_secdisabledsession) | 752.3765 | 1502.7384 | 1502.7325 | 3.93 | 0 | 90 | 1.5e-007 | 1 | U | K.SGVSLSAEQNENLR.S [2294](http://10.200.41.11/mascot/cgi/peptide_view.pl?file=..%2Fdata%2F20160609%2FF081289.dat&query=2294&hit=1&index=gi%7C392616944%7Cgb%7CEIW99372.1%7C&db_idx=1&px=1&section=5&ave_thresh=32&_ignoreionsscorebelow=0&report=0&_sigthreshold=0.05&_msresflags=1089&_msresflags2=2&percolate=-1&percolate_rt=0&_minpeplen=5&sessionID=all_secdisabledsession) |

### Protein sequence coverage: 4%

Matched peptides shown in ***bold red***.

| **1** | MLNIQNYSAS | PHPGIVAERP | QTPSASEHVE | TAVVPSTTEH | RGTDIISLSQ |
| --- | --- | --- | --- | --- | --- |
| **51** | AATKIQQAQQ | TLQSTPPISE | ENNDERTLAR | QQLTSSLNAL | AK**SGVSLSAE** |
| **101** | **QNENLR**SAFS | APTSALFSAS | PMAQPRTTIS | DAEIWDMVSQ | NISAIGDSYL |
| **151** | GVYENVVAVY | TDFYQAFSDI | LSKMGGWLLP | GKDGNTVKLD | VTSLKNDLNS |
| **201** | LVNKYNQINS | NTVLFPAQSG | SGVKVATEAE | ARQWLSELNL | PNSCLKSYGS |
| **251** | GYVVTVDLTP | LQKMVQDIDG | LGAPGKDSKL | EMDNAKYQAW | QSGFKAQEEN |
| **301** | MKTTLQTLTQ | KYSNANSLYD | NLVKVLSSTI | SSSLETAKSF | LQG |

| 8. | [gi|194401173|gb|ACF61395.1|](http://10.200.41.11/mascot/cgi/protein_view.pl?file=..%2Fdata%2F20160609%2FF081289.dat&hit=gi%7C194401173%7Cgb%7CACF61395.1%7C&db_idx=1&px=1&ave_thresh=32&_ignoreionsscorebelow=0&report=0&_sigthreshold=0.05&_msresflags=1089&_msresflags2=2&percolate=-1&percolate_rt=0&_minpeplen=5&sessionID=all_secdisabledsession)    Mass: 49778    Score: 89     Matches: 1(1)  Sequences: 1(1)  emPAI: 0.12 |
| --- | --- |
|  | flagellar hook-associated protein 2 [Salmonella enterica subsp. enterica serovar Newport str. SL254] |

|  | Query | Observed | Mr(expt) | Mr(calc) | ppm | Miss | Score | Expect | Rank | Unique | Peptide |
| --- | --- | --- | --- | --- | --- | --- | --- | --- | --- | --- | --- |
|  | [2592](http://10.200.41.11/mascot/cgi/peptide_view.pl?file=..%2Fdata%2F20160609%2FF081289.dat&query=2592&hit=1&index=gi%7C194401173%7Cgb%7CACF61395.1%7C&db_idx=1&px=1&section=5&ave_thresh=32&_ignoreionsscorebelow=0&report=0&_sigthreshold=0.05&_msresflags=1089&_msresflags2=2&percolate=-1&percolate_rt=0&_minpeplen=5&sessionID=all_secdisabledsession) | 841.4269 | 1680.8392 | 1680.8319 | 4.37 | 0 | 89 | 2.1e-007 | 1 | U | K.QYLSVSNSIDETVAR.Y |

### Protein sequence coverage: 3%

Matched peptides shown in ***bold red***.

| **1** | MASISSLGVG | SNLPLDQLLT | DLTKNEKGRL | TPITKQQSAN | SAKLTAYGTL |
| --- | --- | --- | --- | --- | --- |
| **51** | KSALEKFQTA | NTALNKADLF | KSTVASSTTE | DLKVSTTAGA | AAGTYKISVT |
| **101** | QLAAAQSLAT | KTTFATTKEQ | LGDTSVTSRT | IKIEQPGRKE | PLEIKLDKGD |
| **151** | TSMEAIRDAI | NDADSGIAAS | IVKVKENEFQ | LVLTANSGTD | NTMKITVEGD |
| **201** | TKLNDLLAYD | STTNTGNMQE | LVKAENAKLN | VNGIDIERQS | NTVTDAPQGI |
| **251** | TLTLTKKVTD | ATVTVTKDDT | KAKEAIKSWV | DAYNSLVDTF | SSLTKYTAVE |
| **301** | PGEEASDKNG | ALLGDSVVRT | IQTGIRAQFA | NSGSNSAFKT | MAEIGITQDG |
| **351** | TSGKLKIDDD | KLTKVLKDNT | AAARELLVGD | GKETGITTKI | ATEVKSYLAD |
| **401** | DGIIDNAQDN | VNATLKSLTK | **QYLSVSNSID** | **ETVAR**YKAQF | TQLDTMMSKL |
| **451** | NNTSSYLTQQ | FTAMNKS |  |  |  |

| 15. | [gi|194403640|gb|ACF63862.1|](http://10.200.41.11/mascot/cgi/protein_view.pl?file=..%2Fdata%2F20160609%2FF081289.dat&hit=gi%7C194403640%7Cgb%7CACF63862.1%7C&db_idx=1&px=1&ave_thresh=32&_ignoreionsscorebelow=0&report=0&_sigthreshold=0.05&_msresflags=1089&_msresflags2=2&percolate=-1&percolate_rt=0&_minpeplen=5&sessionID=all_secdisabledsession)    Mass: 62382    Score: 54     Matches: 4(1)  Sequences: 2(1)  emPAI: 0.09 |
| --- | --- |
|  | cell invasion protein SipB [Salmonella enterica subsp. enterica serovar Newport str. SL254] |

|  | Query | Observed | Mr(expt) | Mr(calc) | ppm | Miss | Score | Expect | Rank | Unique | Peptide |
| --- | --- | --- | --- | --- | --- | --- | --- | --- | --- | --- | --- |
|  | [710](http://10.200.41.11/mascot/cgi/peptide_view.pl?file=..%2Fdata%2F20160609%2FF081289.dat&query=710&hit=5&index=gi%7C194403640%7Cgb%7CACF63862.1%7C&db_idx=1&px=1&section=5&ave_thresh=32&_ignoreionsscorebelow=0&report=0&_sigthreshold=0.05&_msresflags=1089&_msresflags2=2&percolate=-1&percolate_rt=0&_minpeplen=5&sessionID=all_secdisabledsession) | 490.7454 | 979.4763 | 979.4909 | -14.95 | 0 | 2 | 1e+002 | 5 | U | K.LFTQGMQR.I [709](http://10.200.41.11/mascot/cgi/peptide_view.pl?file=..%2Fdata%2F20160609%2FF081289.dat&query=709&hit=5&index=gi%7C194403640%7Cgb%7CACF63862.1%7C&db_idx=1&px=1&section=5&ave_thresh=32&_ignoreionsscorebelow=0&report=0&_sigthreshold=0.05&_msresflags=1089&_msresflags2=2&percolate=-1&percolate_rt=0&_minpeplen=5&sessionID=all_secdisabledsession) [711](http://10.200.41.11/mascot/cgi/peptide_view.pl?file=..%2Fdata%2F20160609%2FF081289.dat&query=711&hit=6&index=gi%7C194403640%7Cgb%7CACF63862.1%7C&db_idx=1&px=1&section=5&ave_thresh=32&_ignoreionsscorebelow=0&report=0&_sigthreshold=0.05&_msresflags=1089&_msresflags2=2&percolate=-1&percolate_rt=0&_minpeplen=5&sessionID=all_secdisabledsession) |
|  | [1047](http://10.200.41.11/mascot/cgi/peptide_view.pl?file=..%2Fdata%2F20160609%2FF081289.dat&query=1047&hit=1&index=gi%7C194403640%7Cgb%7CACF63862.1%7C&db_idx=1&px=1&section=5&ave_thresh=32&_ignoreionsscorebelow=0&report=0&_sigthreshold=0.05&_msresflags=1089&_msresflags2=2&percolate=-1&percolate_rt=0&_minpeplen=5&sessionID=all_secdisabledsession) | 531.7850 | 1061.5554 | 1061.5505 | 4.58 | 0 | 54 | 0.00083 | 1 | U | R.LAEAAFEGVR.K |

### Protein sequence coverage: 3%

Matched peptides shown in ***bold red***.

| **1** | MVNDASSISR | SGYTQNPR**LA** | **EAAFEGVR**KN | TDFLKAADKA | FKDVVATKAG |
| --- | --- | --- | --- | --- | --- |
| **51** | DLKAGTKSGE | SAINTVGLKP | PTDAAREKLS | SEGQLTLLLG | KLMTLLGDVS |
| **101** | LSQLESRLAV | WQAMIESQKE | MGIQVSKEFQ | TALGEAQEAT | DLYEASIKKT |
| **151** | DTAKSVYDAA | AKKLTQAQNK | LQSLDPADPG | YAQAEAAVEQ | AGKEATEAKE |
| **201** | ALDKATDATV | KAGTDAKAKA | EKADNILTKF | QGTANAASQN | QVSQGEQDNL |
| **251** | SNVARLTMLM | AMFIEIVGKN | TEESLQNDLA | LFNALQEGRQ | AEMEKKSAEF |
| **301** | QEETRKAEET | NRIMGCIGKV | LGALLTIVSV | VAAVFTGGAS | LALAAVGLAV |
| **351** | MVADEIVKAA | TGVSFIQQAL | NPIMEHVLKP | LMELIGKAIT | KALEGLGVDK |
| **401** | KTAEMAGSIV | GAIVAAIAMV | AVIVVVAVVG | KGAAAKLGNA | LSKMMGETIK |
| **451** | KLVPNVLKQL | AQNGSK**LFTQ** | **GMQR**ITSGLG | NVGSKMGLQT | NALSKELVGN |
| **501** | TLNKVALGME | VTNTAAQSAG | GVAEGVFIKN | ASEALADFML | ARFAMDQIQQ |
| **551** | WLKQSVEIFG | ENQKVTAELQ | KAMSSAVQQN | ADASRFILRQ | SRA |

| 17. | [gi|194404219|gb|ACF64441.1|](http://10.200.41.11/mascot/cgi/protein_view.pl?file=..%2Fdata%2F20160609%2FF081289.dat&hit=gi%7C194404219%7Cgb%7CACF64441.1%7C&db_idx=1&px=1&ave_thresh=32&_ignoreionsscorebelow=0&report=0&_sigthreshold=0.05&_msresflags=1089&_msresflags2=2&percolate=-1&percolate_rt=0&_minpeplen=5&sessionID=all_secdisabledsession)    Mass: 34155    Score: 41     Matches: 2(0)  Sequences: 1(0) |
| --- | --- |
|  | flagellar hook-associated protein 3 [Salmonella enterica subsp. enterica serovar Newport str. SL254] |

|  | Query | Observed | Mr(expt) | Mr(calc) | ppm | Miss | Score | Expect | Rank | Unique | Peptide |
| --- | --- | --- | --- | --- | --- | --- | --- | --- | --- | --- | --- |
|  | [1155](http://10.200.41.11/mascot/cgi/peptide_view.pl?file=..%2Fdata%2F20160609%2FF081289.dat&query=1155&hit=1&index=gi%7C194404219%7Cgb%7CACF64441.1%7C&db_idx=1&px=1&section=5&ave_thresh=32&_ignoreionsscorebelow=0&report=0&_sigthreshold=0.05&_msresflags=1089&_msresflags2=2&percolate=-1&percolate_rt=0&_minpeplen=5&sessionID=all_secdisabledsession) | 545.7797 | 1089.5449 | 1089.5415 | 3.16 | 0 | 34 | 0.068 | 1 | U | K.SVTQQVDSAR.T [1156](http://10.200.41.11/mascot/cgi/peptide_view.pl?file=..%2Fdata%2F20160609%2FF081289.dat&query=1156&hit=1&index=gi%7C194404219%7Cgb%7CACF64441.1%7C&db_idx=1&px=1&section=5&ave_thresh=32&_ignoreionsscorebelow=0&report=0&_sigthreshold=0.05&_msresflags=1089&_msresflags2=2&percolate=-1&percolate_rt=0&_minpeplen=5&sessionID=all_secdisabledsession) |

### Protein sequence coverage: 3%

Matched peptides shown in ***bold red***.

| **1** | MRISTQMMYE | QNMSGITNSQ | AEWMKLGEQM | STGKRVTNPS | DDPIAASQAV |
| --- | --- | --- | --- | --- | --- |
| **51** | VLSQAQAQNS | QYALARTFAT | QKVSLEESVL | SQVTTAIQTA | QEKIVYAGNG |
| **101** | TLSDDDRASL | ATDLQGIRDQ | LMNLANSTDG | NGRYIFAGYK | TEAAPFDQAT |
| **151** | GGYHGGEK**SV** | **TQQVDSAR**TM | VIGHTGAQIF | NSITSNAVPE | PDGSDSEKNL |
| **201** | FVMLDTAIAA | LKTPVEGNDV | EKEKAAAAID | KTNRGLKNSL | NNVLTVRAEL |
| **251** | GTQLSELSTL | DSLGSDRALG | QKLQMSNLVD | VDWNSVISSY | VMQQAALQAS |
| **301** | YKTFTDMQGM | SLFQLNR |  |  |  |

*******************************************************************************************************************************************************

**2^nd^ technical replicate**

**Enzyme : Trypsin**

**Variable modifications :** [**Oxidation (M)**](http://10.200.41.11/mascot/cgi/client.pl?modification&mod_name=Oxidation%20%28M%29&file=..%2Fdata%2F20160609%2FF081290.dat)

**Mass values : Monoisotopic**

**Protein Mass : Unrestricted**

**Peptide Mass Tolerance : ± 20 ppm**

**Fragment Mass Tolerance: ± 0.4 Da**

**Max Missed Cleavages : 3**

**Instrument type : Default**

**Number of queries : 2786**

| Protein hits           : | [**gi|194402702|gb|ACF62924.1|**](http://10.200.41.11/mascot/cgi/master_results.pl?file=..%2Fdata%2F20160609%2FF081290.dat#Hit1) | cell invasion protein SipA [Salmonella enterica subsp. enterica serovar Newport str. SL254] |
| --- | --- | --- |
|  | [**gi|194403331|gb|ACF63553.1|**](http://10.200.41.11/mascot/cgi/master_results.pl?file=..%2Fdata%2F20160609%2FF081290.dat#Hit2) | negative regulator of flagellin synthesis [Salmonella enterica subsp. enterica serovar Newport str. SL254] |
|  | [**gi|392616945|gb|EIW99373.1|**](http://10.200.41.11/mascot/cgi/master_results.pl?file=..%2Fdata%2F20160609%2FF081290.dat#Hit3) | pathogenicity island 1 effector protein SipC [Salmonella enterica subsp. enterica serovar Newport str. Levine 15] |
|  | [**gi|392616944|gb|EIW99372.1|**](http://10.200.41.11/mascot/cgi/master_results.pl?file=..%2Fdata%2F20160609%2FF081290.dat#Hit4) | cell invasion protein SipD [Salmonella enterica subsp. enterica serovar Newport str. Levine 15] |
|  | [**gi|194401173|gb|ACF61395.1|**](http://10.200.41.11/mascot/cgi/master_results.pl?file=..%2Fdata%2F20160609%2FF081290.dat#Hit5) | flagellar hook-associated protein 2 [Salmonella enterica subsp. enterica serovar Newport str. SL254] |
|  | [**gi|194401698|gb|ACF61920.1|**](http://10.200.41.11/mascot/cgi/master_results.pl?file=..%2Fdata%2F20160609%2FF081290.dat#Hit6) | translation elongation factor Tu [Salmonella enterica subsp. enterica serovar Newport str. SL254] |
|  | [**gi|50830890|gb|AAT81610.1|**](http://10.200.41.11/mascot/cgi/master_results.pl?file=..%2Fdata%2F20160609%2FF081290.dat#Hit7) | phase 1 flagellin [Salmonella enterica subsp. enterica serovar Newport] |
|  | [**gi|194401732|gb|ACF61954.1|**](http://10.200.41.11/mascot/cgi/master_results.pl?file=..%2Fdata%2F20160609%2FF081290.dat#Hit8) | chaperonin GroS [Salmonella enterica subsp. enterica serovar Newport str. SL254] |
|  | [**gi|194401878|gb|ACF62100.1|**](http://10.200.41.11/mascot/cgi/master_results.pl?file=..%2Fdata%2F20160609%2FF081290.dat#Hit9) | ribosomal protein L11 [Salmonella enterica subsp. enterica serovar Newport str. SL254] |
|  | [**gi|194401103|gb|ACF61325.1|**](http://10.200.41.11/mascot/cgi/master_results.pl?file=..%2Fdata%2F20160609%2FF081290.dat#Hit10) | ribosomal protein L6 [Salmonella enterica subsp. enterica serovar Newport str. SL254] |
|  | [**gi|194404381|gb|ACF64603.1|**](http://10.200.41.11/mascot/cgi/master_results.pl?file=..%2Fdata%2F20160609%2FF081290.dat#Hit11) | DNA-binding protein HU-alpha [Salmonella enterica subsp. enterica serovar Newport str. SL254] |
|  | [**gi|194405415|gb|ACF65637.1|**](http://10.200.41.11/mascot/cgi/master_results.pl?file=..%2Fdata%2F20160609%2FF081290.dat#Hit12) | ribosomal protein L15 [Salmonella enterica subsp. enterica serovar Newport str. SL254] |
|  | [**gi|194405308|gb|ACF65530.1|**](http://10.200.41.11/mascot/cgi/master_results.pl?file=..%2Fdata%2F20160609%2FF081290.dat#Hit13) | DNA-binding protein HU-beta [Salmonella enterica subsp. enterica serovar Newport str. SL254] |
|  | [**gi|194404219|gb|ACF64441.1|**](http://10.200.41.11/mascot/cgi/master_results.pl?file=..%2Fdata%2F20160609%2FF081290.dat#Hit14) | flagellar hook-associated protein 3 [Salmonella enterica subsp. enterica serovar Newport str. SL254] |
|  | [**gi|392742504|gb|EIZ99592.1|**](http://10.200.41.11/mascot/cgi/master_results.pl?file=..%2Fdata%2F20160609%2FF081290.dat#Hit15) | phage regulatory protein [Salmonella enterica subsp. enterica serovar Newport str. CVM 35199] |
|  | [**gi|195630380|gb|EDX49006.1|**](http://10.200.41.11/mascot/cgi/master_results.pl?file=..%2Fdata%2F20160609%2FF081290.dat#Hit16) | protein RecT [Salmonella enterica subsp. enterica serovar Newport str. SL317] |
|  | [**gi|582044476|ref|WP_024132356.1|**](http://10.200.41.11/mascot/cgi/master_results.pl?file=..%2Fdata%2F20160609%2FF081290.dat#Hit17) | integrase [Salmonella enterica] |

| 1. | [gi|194402702|gb|ACF62924.1|](http://10.200.41.11/mascot/cgi/protein_view.pl?file=..%2Fdata%2F20160609%2FF081290.dat&hit=gi%7C194402702%7Cgb%7CACF62924.1%7C&db_idx=1&px=1&ave_thresh=31&_ignoreionsscorebelow=0&report=0&_sigthreshold=0.05&_msresflags=1089&_msresflags2=2&percolate=-1&percolate_rt=0&_minpeplen=5&sessionID=all_secdisabledsession)    Mass: 72333    Score: 531    Matches: 14(13)  Sequences: 5(5)  emPAI: 0.42 |
| --- | --- |
|  | cell invasion protein SipA [Salmonella enterica subsp. enterica serovar Newport str. SL254] |

|  | Query | Observed | Mr(expt) | Mr(calc) | ppm | Miss | Score | Expect | Rank | Unique | Peptide |
| --- | --- | --- | --- | --- | --- | --- | --- | --- | --- | --- | --- |
|  | [601](http://10.200.41.11/mascot/cgi/peptide_view.pl?file=..%2Fdata%2F20160609%2FF081290.dat&query=601&hit=1&index=gi%7C194402702%7Cgb%7CACF62924.1%7C&db_idx=1&px=1&section=5&ave_thresh=31&_ignoreionsscorebelow=0&report=0&_sigthreshold=0.05&_msresflags=1089&_msresflags2=2&percolate=-1&percolate_rt=0&_minpeplen=5&sessionID=all_secdisabledsession) | 490.7447 | 979.4748 | 979.4723 | 2.58 | 0 | 50 | 0.0013 | 1 | U | R.TFIDNSQR.N [600](http://10.200.41.11/mascot/cgi/peptide_view.pl?file=..%2Fdata%2F20160609%2FF081290.dat&query=600&hit=1&index=gi%7C194402702%7Cgb%7CACF62924.1%7C&db_idx=1&px=1&section=5&ave_thresh=31&_ignoreionsscorebelow=0&report=0&_sigthreshold=0.05&_msresflags=1089&_msresflags2=2&percolate=-1&percolate_rt=0&_minpeplen=5&sessionID=all_secdisabledsession) [602](http://10.200.41.11/mascot/cgi/peptide_view.pl?file=..%2Fdata%2F20160609%2FF081290.dat&query=602&hit=1&index=gi%7C194402702%7Cgb%7CACF62924.1%7C&db_idx=1&px=1&section=5&ave_thresh=31&_ignoreionsscorebelow=0&report=0&_sigthreshold=0.05&_msresflags=1089&_msresflags2=2&percolate=-1&percolate_rt=0&_minpeplen=5&sessionID=all_secdisabledsession) |
[truncated: 892,468 more chars]
